# Formal Synthesis of *Ortho*-Cyanated *N*-Heterocycles via Direct, Metal-Free Cyanation of *N*-Oxides Under Benign Conditions

**DOI:** 10.3390/molecules31020276

**Published:** 2026-01-13

**Authors:** Hua Xiao, Reziyamu Wufuer, Dong Wang

**Affiliations:** 1 State Key Laboratory of Chemistry and Utilization of Carbon Based Energy Resources, College of Chemistry, Xinjiang University, Urumqi 830017, China; 2 School of Pharmaceutical Sciences and Institute of Materia Medica, Xinjiang University, Urumqi 830017, China

**Keywords:** nitrogen heterocycles, C-H functionalization, *N*-oxides, green solvents, cyanation

## Abstract

The introduction of cyano groups into aza-heterocyclic compounds plays a pivotal role in accessing diverse derivatives that are essential for the development of natural products, pharmaceuticals, and agrochemicals. Herein, we report a unified strategy for the direct *ortho*-C-H cyanation of a broad range of heterocyclic *N*-oxides, including pyridine, quinoline, isoquinoline, and pyrimidine derivatives. This transformation proceeds under mild conditions without the need for external activating agents or catalysts, and has been successfully applied to structurally complex, biologically relevant molecules. Compared to existing methodologies, our approach offers several distinct advantages: the use of non-prefunctionalized heteroarene substrates, environmentally benign reaction solvents, operational simplicity, broad substrate scope, and high efficiency in generating diverse *ortho*-cyanated heterocyclic compounds. Moreover, the method demonstrates considerable potential for scalable synthesis.

## 1. Introduction

**Scheme 1:**
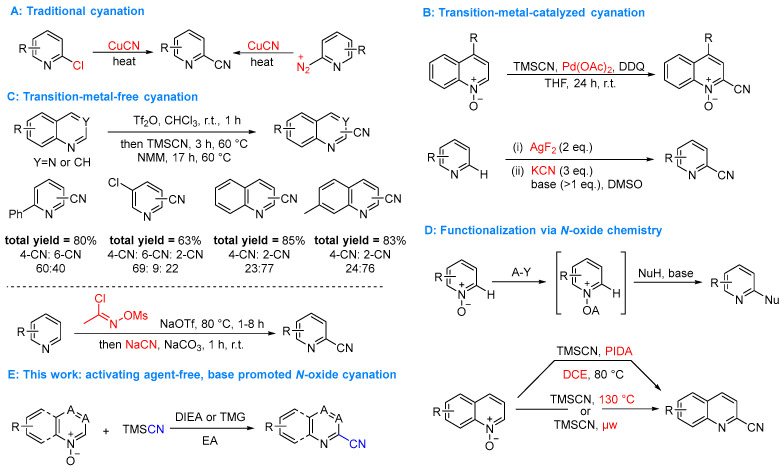
Challenges and approaches to the synthesis of 2-phenoxyisoquinolines.

Nitrile-containing aza-arene derivatives are common structural motifs in natural products [1], agrochemicals [2], and pharmaceutical molecules [3,4,5,6], exhibiting diverse biological activities and medicinal values, including antibacterial [7,8,9], anticancer [10,11,12], antioxidant [13,14,15], and antimalarial effects [16]. As a versatile functional group, the cyano moiety can be readily transformed into various functionalities such as amines, amides, and carboxylic acids. Consequently, these compounds also serve as key intermediates widely employed in synthetic chemistry. Despite the prevalence of such structures, the de novo synthesis of cyano-substituted aza-arenes via C-H functionalization remains challenging [17] (Scheme 1). Numerous methods have been reported for the *ortho*-cyanation of nitrogen-containing heterocycles. Traditional approaches rely on the Sandmeyer [18,19] and Rosenmund-von Braun reactions [20] (Scheme 1A), which involve the substitution of prefunctionalized halogenated quinolines or quinoline diazonium salts using toxic CuCN at elevated temperatures. Clearly, these methods are severely limited by their requirement for harsh reaction conditions. Alternative strategies involve transition metal-mediated nucleophilic substitution of nitrogen heterocycles with cyanation reagents (Scheme 1B). In 2004, the Tagawa group reported a palladium-catalyzed direct C2-H cyanation of quinoline *N*-oxides. However, this method was only applicable to quinoline *N*-oxides bearing a substituent at the 4-position, significantly restricting its utility [21]. In 2014, the Hartwig group described a method for pyridine C2-H cyanation utilizing a silver fluoride-mediated sequential C2-H substitution followed by nucleophilic substitution with cyanide in the presence of base [22]. The necessity for expensive transition metal reagents, the difficulty in removing metal residues from the final products, and associated environmental concerns limit the application of such methods in the pharmaceutical industry. Therefore, the development of transition metal-free cyanation methodologies is of great significance.

Transition metal-free methods for the cyanation of nitrogen-containing heterocycles primarily fall into two categories. The first approach involves the direct activation of the heterocyclic substrate using a suitable activating agent, followed by nucleophilic addition with a cyanide reagent (Scheme 1C). In 2005, Katritzky and coworkers reported a landmark study on the direct cyanation of pyridines using potassium cyanide, activated by nitric acid and trifluoroacetic anhydride. While this work constituted one of the first examples of such a direct cyanation, the requirement for highly toxic potassium cyanide as a reagent significantly limited its practical applicability [23]. In 2017, Paton group reported the activation of aza-arenes using trifluoromethanesulfonic anhydride (Tf_2_O). The activated aza-arene readily undergoes nucleophilic reaction with cyanide, and subsequent elimination of the trifluoromethanesulfonate anion mediated by *N*-methylmorpholine yields the cyano-substituted heteroaromatics [24]. However, this reaction exhibits incomplete regioselectivity, resulting in the formation of isomeric mixtures. Cyanation predominantly occurs at the 4-position for pyridine *N*-oxides. Moreover, the current substrate scope remains limited, with only four examples reported for quinolines and two for isoquinolines, indicating that more comprehensive studies are necessary to fully evaluate the generality of this method. In the same year, the team of P. S. Fier reported the *ortho*-cyanation of pyridine and isoquinoline derivatives using highly toxic NaCN as the cyanide source and α-chloro-O-mesyl aldoxime as an activating agent [25]. Nevertheless, this method requires the synthesis of the activating agent, involves a two-step operation, and is not applicable to quinoline or other nitrogen-containing heterocycles. The second method utilizes *N*-oxides of nitrogen heterocycles to achieve *ortho*-cyanation (Scheme 1D). Functionalization of the C2-H bond of heterocyclic compounds via their *N*-oxides represents the most widely employed strategy [26,27,28,29,30]. Current methods typically involve the combination of the *N*-oxide with an activating agent to enhance the electrophilicity at the C2-position, enabling subsequent reaction with a nucleophile to afford the desired product [31,32,33,34,35,36,37].

In 2018, the Sun group reported a method using a hypervalent iodine **III**) compound as an activating agent for *ortho*-cyanation of nitrogen heterocycles [38]. However, this method is primarily applicable to quinoline derivatives and exhibits very limited applicability to pyridine, isoquinoline, and other heterocyclic compounds. In 2019, the Das group reported a method for the synthesis of *ortho*-cyanated aromatic heterocycles that avoids the use of activating agents, transition metals, and solvents [39]. Although this reaction affords various *ortho*-cyanated quinolines in good yields, it requires high temperatures (130 °C) or microwave heating and exhibits a narrow substrate scope. The methodology is not applicable to heterocycles containing ester or amide functional groups. Furthermore, while primarily effective for quinoline derivatives, its applicability to pyridine and isoquinoline systems remains underexplored, with only two examples reported for each to date. Further investigation is warranted to broaden the scope of this transformation. Therefore, the development of an efficient and versatile cyanation methodology operating under mild conditions, exhibiting broad functional group tolerance, and applicable to diverse aza-heterocyclic compounds is of significant importance.

Our research group has long been committed to the development of efficient C-H functionalization methodologies for nitrogen-containing heteroarenes [40,41,42,43,44]. A significant challenge in achieving C2-H functionalization of these heterocycles arises from the presence of multiple chemically similar C-H bonds, making site selectivity a major hurdle. Additionally, side reactions between activating agents and nucleophiles must be overcome. To explore a green and practical method for *ortho*-cyanation of nitrogen heterocycles, this study reports a novel approach. Under activating agent-free and transition metal-free conditions, *N*-oxides directly react with TMSCN (trimethylsilyl cyanide) in the presence of a base. This mild and efficient method enables the *ortho*-cyanation of diverse nitrogen-containing heterocycles (Scheme 1E). The method offers significant advantages over existing approaches: excellent generality for a wide range of *N*-heterocycles, improved greenness via environmentally benign ethyl acetate (EA) solvent [45] and high atom economy, the absence of transition metals or external activating agents—reducing cost, purification, and environmental impact—as well as practical benefits including broad substrate scope, mild conditions, and operational simplicity.

## 2. Results

We initiated our study by screening optimal reaction conditions using quinoline *N*-oxide and TMSCN as the model reaction (Table 1). Under initial conditions, quinoline *N*-oxide (100 mg, 1.0 equiv) was reacted with TMSCN (3.0 equiv) in anhydrous 1,2-dichloroethane (DCE) using α-lipoic acid as a catalyst, methanesulfonic anhydride (Ms_2_O) as an activating agent, and tetramethylguanidine (TMG) as a base. The analysis indicated residual starting material, prompting the addition of extra TMSCN (3.0 equiv) and Ms_2_O (2.0 equiv). After an additional 36 h, compound **2a** was obtained in 86% yield (entry 1). Subsequently, omitting the catalyst afforded the target product in a similar yield (entry 2). Initially, we hypothesized that lipoic acid, being a thioether and a strong nucleophile, could catalyze the reaction. We proposed that it would attack the activated pyridinium salt to form a pyridylsulfonium intermediate, which would subsequently react with TMSCN to yield the product. However, experimental results showed that it had no catalytic effect. Screening of alternative activating agents (entries 3–5) resulted in decreased yields. The reaction proceeded equally well (86% yield) in the absence of base (entry 6). However, omission of both activating agent and base resulted in no product formation (entry 7). Recognizing that TMSCN serves not only as the cyanide source but also provides a trimethylsilyl group potentially capable of activating the *N*-oxide, and considering that activating agents frequently induce side reactions, we investigated the feasibility of an activating agent-free strategy [46]. Notably, conducting the reaction with base but without any activating agent afforded the target product in 75% yield (entry 8). This promising result prompted us to optimize the base under these activating agent-free conditions (entries 9–12). *N*,*N*-Diisopropylethylamine (DIEA) proved optimal, affording the product in 96% yield (entry 9). Solvent screening followed (entries 13–17). Dichloromethane (DCM) provided a comparable yield to DCE. Given the higher toxicity of DCE, DCM was selected for subsequent optimizations. Further screening of base equivalents and solvent concentration (entries 18–21) identified entry 21 as improved conditions. To enhance the green credentials, solvent optimization was revisited (entries 22–25). Gratifyingly, employing EA afforded the product in 93% yield. Consequently, the conditions described in entry 25 were established as optimal.

**Scheme 2:**
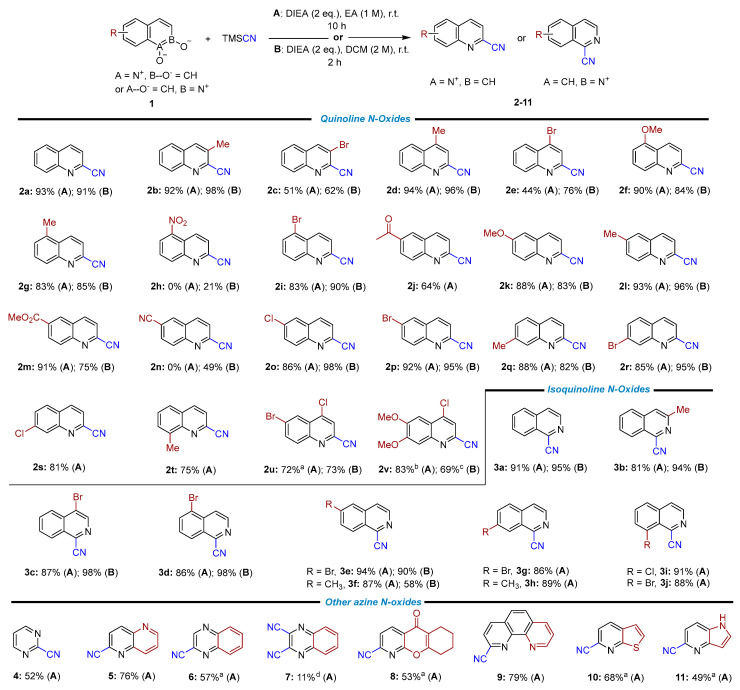
Substrate scope for the *ortho*-cyanation of azine *N*-oxides. Unless otherwise noted, all reactions were conducted at rt with azine *N*-oxide (100 mg, 1.0 equiv), TMSCN (3.0 equiv), and DIEA (2.0 equiv) in EA (1.0 M, condition A) or DCM (2.0 M, condition B). ^a^ The reaction mixture was heated to 80 °C. ^b^ The reaction mixture was heated to 100 °C. ^c^ After reacting for 2 h, extra TMSCN (3.0 equiv) was added to the reaction mixture. ^d^ TMSCN (6.0 equiv), and DIEA (4.0 equiv) were utilized, and the reaction mixture was heated to 80 °C.

Following the establishment of optimal conditions, the substrate scope for the *ortho*-cyanation of azine *N*-oxides with TMSCN was further explored (Scheme 2). The study demonstrated that various quinoline *N*-oxides readily underwent the reaction, affording the target products in moderate to excellent yields (up to 98%). The reaction proceeded efficiently regardless of substituents present at the 3-, 4-, 5-, 6-, 7-, or 8-position of the quinoline *N*-oxide, yielding the corresponding 2-cyanoquinoline derivatives (**2a**–**t**). Furthermore, the method exhibited excellent tolerance towards diverse functional groups, including methyl, methoxy, halogens, acyl, ester, cyano, and nitro groups. When substituents were present at the 3- or 4-position of the quinoline *N*-oxide, substrates bearing electron-donating groups (EDGs) generally afforded the corresponding products in higher yields than those with electron-withdrawing groups (EWGs) (**2b** > **2c**, **2d** > **2e**). For substituents at the 5-, 6-, 7-, or 8-positions (**2f**–**g**, **2i**, **2k**–**m**, **2o**–**t**), encompassing both electron-donating and weakly electron-withdrawing groups, no significant yield differences attributable to electronic effects were observed. Notably, the presence of strong EWGs on the quinoline *N*-oxide (**2h**, **2j**, **2n**) significantly impacted the reaction efficiency. Employing Method A resulted in no product formation for **2h** and **2n**. However, switching to Method B enabled the isolation of the corresponding products in moderate to low yields. The reaction remained effective for polysubstituted quinoline *N*-oxides (**2u**–**v**). Additionally, the methodology proved highly efficient for isoquinoline *N*-oxides. Substituted isoquinoline *N*-oxides, bearing substituents at the 3-, 4-, 5-, 6-, 7-, or 8-position, smoothly underwent reaction to furnish the corresponding 1-cyanoisoquinoline derivatives in excellent yields (**3a**–**j**).

Based upon these excellent results, we further investigated the substrate scope of this methodology with other nitrogen-containing heteroaromatics. The results demonstrate that the method is also effective for pyrimidine (**4**), 1,8-naphthyridine (**5**), quinoxaline (**6**–**7**), 1,10-phenanthroline (**9**), thienopyridine (**10**), and 4-azaindole (**11**). The lower yield observed for quinoxaline-1,4-dioxide (**7**) is likely attributable to steric hindrance. Notably and gratifyingly, the reaction is also applicable to azachromone derivatives (**8**) [47,48,49], which are privileged structures in medicinal chemistry, affording the corresponding product in moderate yield. This successful application represents a potentially valuable strategy for subsequent pharmaceutical development.

**Scheme 3:**
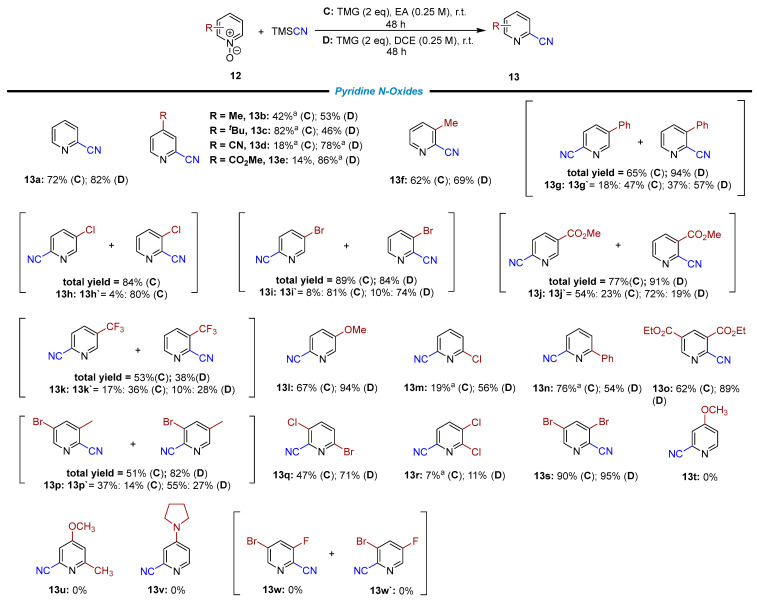
Substrate scope for the *ortho*-cyanation of pyridine *N*-oxides. Unless otherwise noted, all reactions were conducted at rt with pyridine *N*-oxide (100 mg, 1.0 equiv), TMSCN (3.0 equiv), and TMG (2.0 equiv) in EA (0.25 M, condition C) or DCE (0.25 M, condition D). After reacting for 12 h, extra TMSCN (3.0 equiv) was added to the reaction mixture. ^a^ The reaction mixture was heated to 80 °C.

Subsequently, we optimized the reaction conditions for the *ortho*-cyanation of pyridine *N*-oxides (see Table S1, 36 conditions screened) and investigated the substrate scope (Scheme 3). The optimized reaction system demonstrated good applicability to various pyridine *N*-oxides (**13a**–**s**). The reaction tolerates substituents at various positions on the parent ring and is compatible with diverse functional groups, including alkyl (**13b**–**c**, **13f**), cyano (**13d**), ester (**13e**, **13j**, **13o**), phenyl (**13g**, **13n**), halogens (**13h**–**i**, **13m**, **13q**–**s**), trifluoromethyl (**13k**), and methoxy (**13l**). The investigation revealed that for substrates bearing a substituent at the 4-position (**13b**–**e**), those with an EDG afforded the target product in higher yields compared to those with an EWG. For the 4-cyano substituted substrate (**13d**), employing Method C provided the corresponding product in low yield. However, switching the solvent from EA to DCE significantly improved the yield to 78%. This improvement is likely attributable to the poor solubility of the corresponding pyridine *N*-oxide in EA.

Furthermore, substrates bearing a substituent at the 3-position often presented regioselectivity challenges (**13f**–**l**). For 3-methyl or 3-methoxy substituents (**13f**, **13l**), no other products were observed, yielding exclusively **13f** and **13l**, respectively. In contrast, substrates with 3-phenyl, halogen, ester, or trifluoromethyl groups (**13g**–**k**) produced mixtures of *ortho*-cyanated regioisomers, differing in the relative position of the cyano group to the existing substituent (e.g., **13g** vs. **13g′**). The isomer with the cyano group *ortho* to the substituent (**13g′**–**i′**, **13k′**) was typically the major product. Interestingly, for the strong EWG (**13j**) or EDG (**13l**) group at the 3-position, the regioisomer with the cyano group *para* to the substituent became predominant (**13j** > **13j′**, **13l** as the sole product). This reversal in regioselectivity is likely governed by electronic effects. Notably, the reaction remained effective for polysubstituted pyridine *N*-oxides, yielding the corresponding target products (**13q**–**s**). Regrettably, pyridine *N*-oxides bearing a strong electron-donating group at the 4-position (**13t**–**v**) showed no reaction, presumably due to the decreased electrophilicity, which is detrimental to the nucleophilic attack of the cyanide. No reaction was observed for 3-bromo-5-fluoropyridine *N*-oxide (**13w**).

**Scheme 4:**
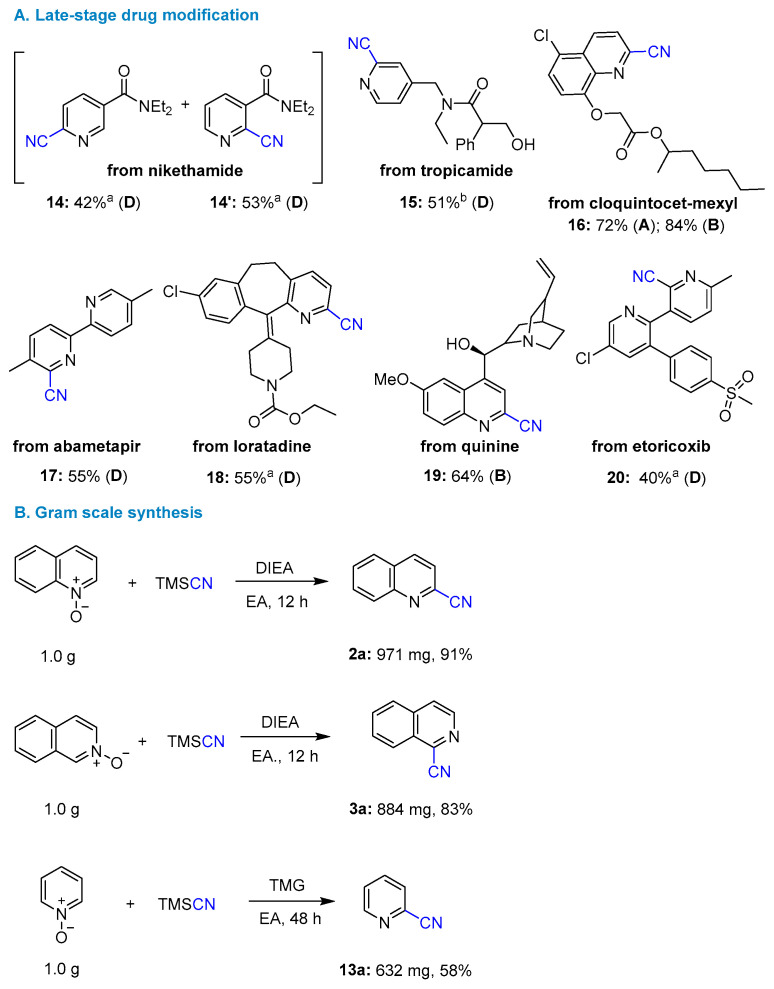
(**A**) Late-stage drug modification. In (**A**), the letters A, B, and D following the yields correspond to the General Procedures A (GPA), B (GPB), and D (GPD), respectively. ^a^ The reaction mixture was heated to 80 °C. ^b^ Following the General Procedure (GPD), but with the omission of the second addition of TMSCN (3.0 equiv). (**B**) Gram scale synthesis.

We subsequently applied this methodology to the late-stage cyanation of several pharmaceutical molecules (Scheme 4A). The reaction proceeded smoothly for pyridine-containing drugs, including nicethamide, tropicamide, abametapir, loratadine, and etoricoxib, affording the corresponding products in good yields (**14**, **15**, **17**, **18**, **20**). Similarly, the reaction proved applicable to quinoline-based drugs such as quinine and cloquintocet-mexyl, successfully yielding the cyanated derivatives (**16** and **19**). These results further demonstrate the mildness and practicality of our reaction conditions. Another remarkable feature of our developed method is scalability. We performed *ortho*-cyanation reactions using quinoline *N*-oxide, isoquinoline *N*-oxide, and pyridine *N*-oxide on a 1 g scale (Scheme 4B). Experimental results confirmed that the yields obtained in these gram-scale reactions were comparable to those achieved in milligram-scale experiments.

A comparative assessment of the green chemistry credentials was conducted between our method (Process A) and the reported literature method [38] (Process B) through established metrics calculations [50,51,52,53]. Representative transformations, including the synthesis of compounds **2a**, **2d**, and **2p** from quinoline *N*-oxide, were selected for this analysis (Table 2). The results indicate that Process A possesses significantly improved green metrics compared to Process B. This is exemplified by its notably high EcoScale score (77–78), which confirms the environmental merit of the developed protocol.

**Scheme 5:**
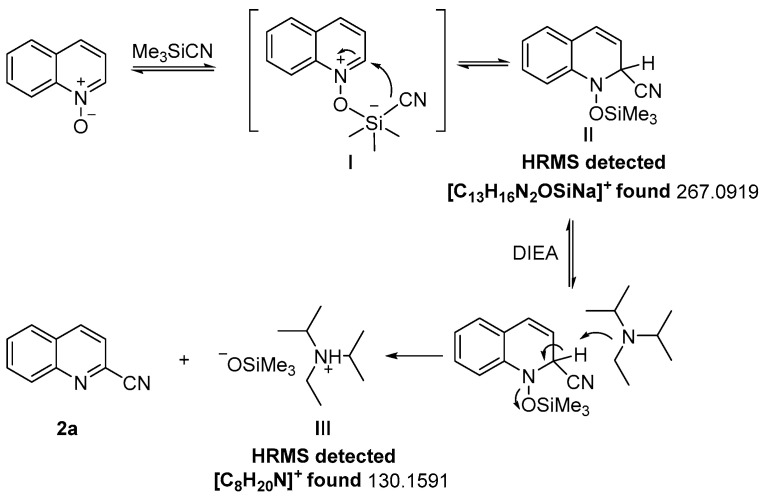
Plausible mechanism.

The proposed mechanism for this transformation is outlined in Scheme 5. First, quinoline *N*-oxide reacts with TMSCN, forming a pentacoordinated silicon complex (**I**) through oxygen anion attack of the organosilane. The enhanced nucleophilicity of the cyano group then facilitates its nucleophilic addition to the C2-position of quinoline *N*-oxide. This addition proceeds via a favorable five-membered-ring transition state to afford intermediate **II**. Subsequent deprotonation by the base DIEA yields the desired product **2a** and eliminates salt **III**. Driven by rearomatization, this step is irreversible. HRMS analysis of the reaction mixture, as well as the solid obtained after evaporating the aqueous phase, detected intermediate **II** and the cation of salt **III**, respectively. These findings provide direct evidence supporting our mechanistic hypothesis.

## 3. Materials and Methods

### 3.1. General

The preparation experiments were performed under air or an argon atmosphere in oven-dried glassware. Solvents used as reaction media were distilled immediately before use: THF was distilled from Na/benzophenone ketyl, DCM and DCE were distilled from calcium hydride, and DMF was obtained from vacuum distillation. All reagents were purchased at the highest commercial quality and used without further purification. Reactions were monitored by thin-layer chromatography (TLC) using ultraviolet light (UV) as the visualizing agent. A metallic heating mantle was used in all of the reactions carried out in this work. Nuclear magnetic resonance spectra (NMR) were recorded on Bruker Avance NEO 600 instruments (Billerica, MA, USA) and were calibrated using residual undeuterated solvent as an internal reference (^1^H NMR: CHC1_3_ 7.26 ppm, ^13^C NMR: CHC1_3_ 77.16 ppm). High-resolution mass spectra (HRMS) were recorded on a Thermo Fisher Scientific Ultimate 3000/Q-Exactive mass spectrometer (Waltham, MA, USA). Melting points were recorded on an automatic melting point meter (Shang Hai Zhuo Guang, Shanghai, China) GM50. The following abbreviations are used to indicate multiplicities: s = singlet, d = doublet, t = triplet, q = quartet, quin = quintet, sex = sextet, sep = septet, dd = doublet of doublets, dt = doublet of triplets, ddd = doublet of doublet of doublets, ddt = doublet of doublet of triplets, m = multiplet).

### 3.2. Cyanation of Quinoline and Isoquinoline N-Oxides: General Procedure A (GPA)

To a solution of quinoline *N*-oxide or isoquinoline *N*-oxide derivative (1.0 equiv) in dry ethyl acetate (EA, 1 M), TMSCN (3.0 equiv) and DIEA (2.0 equiv) were added. The reaction mixture was stirred for 2–24 h (10 h for most examples) and the progress was monitored by TLC. Upon completion, the reaction was quenched with a saturated sodium bicarbonate solution. The mixture was extracted with EA (3 × 2 mL). The combined organic layers were dried over Na_2_SO_4_, filtered, and concentrated under reduced pressure to give the crude product. Purification by flash column chromatography (eluent: PE/EA from 20:1 to 5:1) afforded the desired product.

### 3.3. Cyanation of Quinoline and Isoquinoline N-Oxides: General Procedure B (GPB)

To a solution of quinoline *N*-oxide or isoquinoline *N*-oxide derivative (1.0 equiv) in dry DCM (2 M), TMSCN (3.0 equiv) and DIEA (2.0 equiv) are added. The reaction mixture was stirred for 2 h, and the progress was monitored by TLC. Upon completion, the reaction was quenched with a saturated sodium bicarbonate solution. The mixture was extracted with DCM (3 × 2 mL). The combined organic layers were dried over Na_2_SO_4_, filtered, and concentrated under reduced pressure to give the crude product. Purification by flash column chromatography (eluent: PE/EA from 20:1 to 5:1) affords the desired product.

### 3.4. Cyanation of Pyridine N-Oxides: General Procedure C (GPC)

To a solution of pyridine *N*-oxide derivative (1.0 equiv) in dry EA (0.25 M), TMSCN (3.0 equiv) and *N*,*N*′-tetramethylguanidine (TMG, 2.0 equiv) are added. Following stirring at room temperature for 12 h, additional TMSCN (3.0 equiv) was added. After a further 36 h (reaction completion by TLC), the mixture was quenched with saturated NaHCO_3_ (aq). The mixture was extracted with EA (3 × 2 mL), and the combined organic phases were dried (Na_2_SO_4_), filtered, and concentrated in vacuo. The crude product was purified by flash chromatography (eluent: PE/EA from 10:1 to 3:1) to give the target compound.

### 3.5. Cyanation of Pyridine N-Oxides: General Procedure D (GPD)

To a solution of pyridine *N*-oxide derivative (1.0 equiv) in dry DCE (0.25 M), TMSCN (3.0 equiv) and TMG (2.0 equiv) were added. Following stirring at room temperature for 12 h, additional TMSCN (3.0 equiv) was added. After a further 36 h (reaction completion by TLC), the mixture was quenched with saturated NaHCO_3_ (aq). The mixture is extracted with DCM (3 × 2 mL), and the combined organic phases were dried (Na_2_SO_4_), filtered, and concentrated in vacuo. The crude product was purified by flash chromatography (eluent: PE/EA from 10:1 to 3:1) to give the target compound.

### 3.6. Characterization of Products

Quinoline-2-carbonitrile (**2a**). Following GPA, using quinoline *N*-oxide (100 mg, 0.69 mmol), the title compound was obtained (99 mg, 93% yield) as a white solid. Following GPB, using quinoline N-oxide (100 mg, 0.69 mmol), the title compound was obtained (97 mg, 91% yield) as a white solid. The spectroscopic data are consistent with previously reported [39]. TLC: *R_f_* = 0.50 (PE:EA = 10:1). ^1^H NMR (600 MHz, CDCl_3_) *δ* 8.31 (d, *J* = 8.4 Hz, 1H), 8.17–8.16 (m, 1H), 7.90 (d, *J* = 8.4 Hz, 1H), 7.86–7.83 (m, 1H), 7.72–7.69 (m, 2H).

3-Methylquinoline-2-carbonitrile (**2b**). Following GPA, using 3-methylquinoline *N*-oxide (100 mg, 0.63 mmol), the title compound was obtained (97 mg, 92% yield) as a white solid. Following GPB, using 3-methylquinoline *N*-oxide (100 mg, 0.63 mmol), the title compound was obtained (104 mg, 98% yield) as a white solid. The spectroscopic data are consistent with previously reported [39]. TLC: *R_f_* = 0.45 (PE:EA = 10: 1). ^1^H NMR (600 MHz, CDCl_3_) *δ* 8.10–8.08 (m, 2H), 7.79 (d, *J* = 7.8 Hz, 1H), 7.76–7.73 (m, 1H), 7.66–7.63 (m, 1H), 2.69 (s, 3H).

3-Bromoquinoline-2-carbonitrile (**2c**). Following GPA, using 3-bromoquinoline *N*-oxide (100 mg, 0.45 mmol), the title compound was obtained (54 mg, 51% yield) as a white solid. Following GPB, using 3-bromoquinoline *N*-oxide (100 mg, 0.45 mmol), the title compound was obtained (65 mg, 62% yield) as a white solid. The spectroscopic data are consistent with previously reported [39]. TLC: *R_f_* = 0.50 (PE:EA = 20:1). ^1^H NMR (600 MHz, CDCl_3_) *δ* 8.48 (s, 1H), 8.12 (d, *J* = 8.4 Hz, 1H), 7.86–7.81 (m, 2H), 7.73 (t, *J* = 7.2 Hz, 1H).

4-Methylquinoline-2-carbonitrile (**2d**). Following GPA, using 4-methylquinoline *N*-oxide (100 mg, 0.63 mmol), the title compound was obtained (100 mg, 94% yield) as a white solid. Following GPB, using 4-methylquinoline *N*-oxide (100 mg, 0.63 mmol), the title compound was obtained (102 mg, 96% yield) as a white solid. The spectroscopic data are consistent with previously reported [39]. TLC: *R_f_* = 0.41 (PE:EA = 10:1). ^1^H NMR (600 MHz, CDCl_3_) *δ* 8.12 (d, *J* = 9.0 Hz, 1H), 8.03 (d, *J* = 8.4 Hz, 1H), 7.82–7.79 (m, 1H), 7.71–7.69 (m, 1H), 7.50 (s, 1H), 2.75 (s, 3H).

4-Bromoquinoline-2-carbonitrile (**2e**). Following GPA, using 4-bromoquinoline *N*-oxide (100 mg, 0.45 mmol), the title compound was obtained (46 mg, 44% yield) as a white solid. Following GPB, using 4-bromoquinoline *N*-oxide (100 mg, 0.45 mmol), the title compound was obtained (80 mg, 76% yield) as a white solid. The spectroscopic data are consistent with previously reported [38]. TLC: *R_f_* = 0.52 (PE:EA = 15:1). ^1^H NMR (600 MHz, CDCl_3_) *δ* 8.24 (dd, *J* = 8.4, 0.6 Hz, 1H), 8.17 (d, *J* = 8.4 Hz, 1H), 7.98 (s, 1H), 7.91–7.88 (m, 1H), 7.82–7.80 (m, 1H).

5-Methoxyquinoline-2-carbonitrile (**2f**). Following GPA, using 5-methoxyquinoline *N*-oxide (100 mg, 0.57 mmol), the title compound was obtained (95 mg, 90% yield) as a white solid. Following GPB, using 5-methoxyquinoline *N*-oxide (100 mg, 0.57 mmol), the title compound was obtained (88 mg, 84% yield) as a white solid. The spectroscopic data are consistent with previously reported [54]. TLC: *R_f_* = 0.43 (PE:EA = 20:1). ^1^H NMR (600 MHz, CDCl_3_) *δ* 8.64 (d, *J* = 8.4 Hz, 1H), 7.71–7.66 (m, 2H), 7.60 (d, *J* = 8.4 Hz, 1H), 6.95 (dd, *J* = 7.2, 1.2 Hz, 1H), 4.01 (s, 3H).

5-Methylquinoline-2-carbonitrile (**2g**). Following GPA, using 5-methylquinoline *N*-oxide (100 mg, 0.63 mmol), the title compound was obtained (88 mg, 83% yield) as a white solid. Following GPB, using 5-methylquinoline *N*-oxide (100 mg, 0.63 mmol), the title compound was obtained (90 mg, 85% yield) as a white solid. TLC: *R_f_* = 0.52 (PE:EA = 10:1). Melting point: 127.2–128.0 °C. ^1^H NMR (600 MHz, CDCl_3_) *δ* 8.43 (dd, *J* = 8.4, 0.6 Hz, 1H), 7.98 (d, *J* = 8.4 Hz, 1H), 7.71–7.68 (m, 2H), 7.50 (d, *J* = 7.2 Hz, 1H), 2.70 (s, 3H). ^13^C {^1^H} NMR (150 MHz, CDCl_3_) *δ* 148.6, 135.0, 134.0, 133.2, 131.0, 129.9, 128.17, 128.15, 123.0, 117.7, 18.6. HRMS (+ESI-TOF) *m*/*z*: [M + H]^+^ Calcd for C_11_H_9_N_2_ 169.0760; found 169.0758.

5-Nitroquinoline-2-carbonitrile (**2h**). Following GPB, using 5-nitroquinoline *N*-oxide (100 mg, 0.53 mmol), the title compound was obtained (22 mg, 21% yield) as a yellow solid. The spectroscopic data are consistent with previously reported [39]. TLC: *R_f_* = 0.41 (PE:EA = 10:1). ^1^H NMR (600 MHz, CDCl_3_) *δ* 9.23 (dd, *J* = 9.0, 0.6 Hz, 1H), 8.56 (dd, *J* = 7.8, 1.2 Hz, 1H), 8.51 (d, *J* = 8.4 Hz, 1H), 7.98–7.94 (m, 2H).

5-Bromoquinoline-2-carbonitrile (**2i**). Following GPA, using 5-bromoquinoline *N*-oxide (100 mg, 0.45 mmol), the title compound was obtained (87 mg, 83% yield) as a white solid. Following GPB, using 5-bromoquinoline *N*-oxide (100 mg, 0.45 mmol), the title compound was obtained (94 mg, 90% yield) as a white solid. The spectroscopic data are consistent with previously reported [38]. TLC: *R_f_* = 0.36 (PE:EA = 20:1). ^1^H NMR (600 MHz, CDCl_3_) *δ* 8.70–8.68 (m, 1H), 8.14 (d, *J* = 9.0 Hz, 1H), 7.97 (dd, *J* = 7.2, 0.6 Hz, 1H), 7.80 (d, *J* = 8.4 Hz, 1H), 7.70 (dd, *J* = 8.4, 7.8 Hz, 1H).

6-Acetylquinoline-2-carbonitrile (**2j**). Following GPA, using 6-acetylquinoline *N*-oxide (100 mg, 0.53 mmol), the title compound was obtained (66 mg, 64% yield) as a white solid. TLC: *R_f_* = 0.51 (PE:EA = 10:1). Melting point: 176.9–177.1 °C. ^1^H NMR (600 MHz, CDCl_3_) *δ* 8.49 (d, *J* = 1.2 Hz, 1H), 8.45 (d, *J* = 8.4 Hz, 1H), 8.35 (dd, *J* = 9.0, 1.8 Hz, 1H), 8.20 (d, *J* = 9 Hz, 1H), 7.77 (d, *J* = 8.4 Hz, 1H), 2.75 (s, 3H). ^13^C {^1^H} NMR (150 MHz, CDCl_3_) *δ* 196.9, 149.9, 139.2, 137.1, 135.7, 130.7, 129.49, 129.46, 128.1, 124.2, 117.2, 27.0. HRMS (+ESI-TOF) *m*/*z*: [M + H]^+^ Calcd for C_12_H_9_N_2_O 197.0709; found 197.0711.

6-Methoxyquinoline-2-carbonitrile (**2k**). Following GPA, using 6-methoxyquinoline *N*-oxide (100 mg, 0.57 mmol), the title compound was obtained (92 mg, 88% yield) as a white solid. Following GPB, using 6-methoxyquinoline *N*-oxide (100 mg, 0.57 mmol), the title compound was obtained (87 mg, 83% yield) as a white solid. The spectroscopic data are consistent with previously reported [38]. TLC: *R_f_* = 0.49 (PE:EA = 20:1). ^1^H NMR (600 MHz, CDCl_3_) *δ* 8.15 (d, *J* = 8.4 Hz, 1H), 8.04 (d, *J* = 9.6 Hz, 1H), 7.64 (d, *J* = 8.4 Hz, 1H), 7.47 (dd, *J* = 9.6, 3.0 Hz, 1H), 7.10 (d, *J* = 2.4 Hz, 1H), 3.97 (s, 3H).

6-Methylquinoline-2-carbonitrile (**2l**). Following GPA, using 6-methylquinoline *N*-oxide (100 mg, 0.63 mmol), the title compound was obtained (99 mg, 93% yield) as a white solid. Following GPB, using 6-methylquinoline *N*-oxide (100 mg, 0.63 mmol), the title compound was obtained (102 mg, 96% yield) as a white solid. The spectroscopic data are consistent with previously reported [38]. TLC: *R_f_* = 0.43 (PE:EA= 10:1). ^1^H NMR (600 MHz, CDCl_3_) *δ* 8.18 (d, *J* = 7.8 Hz, 1H), 8.03 (d, *J* = 9.0 Hz, 1H), 7.66–7.63 (m, 3H), 2.57 (s, 3H).

Methyl 2-cyanoquinoline-6-carboxylate (**2m**). Following GPA, using 6-(methoxycarbonyl) quinoline *N*-oxide (100 mg, 0.49 mmol), the title compound was obtained (95 mg, 91% yield) as a white solid. Following GPB, using 6-(methoxycarbonyl)quinoline *N*-oxide (100 mg, 0.49 mmol), the title compound was obtained (78 mg, 75% yield) as a white solid. The spectroscopic data are consistent with previously reported [55]. TLC: *R_f_* = 0.21 (PE:EA = 20:1). ^1^H NMR (600 MHz, CDCl_3_) *δ* 8.64 (d, *J* = 1.2 Hz, 1H), 8.43–8.39 (m, 2H), 8.21 (d, *J* = 9.0 Hz, 1H), 7.76 (d, *J* = 8.4 Hz, 1H), 4.02 (s, 3H).

Quinoline-2,6-dicarbonitrile (**2n**). Following GPB, using quinoline-6-carbonitrile *N*-oxide (100 mg, 0.59 mmol), the title compound was obtained (52 mg, 49% yield) as a white solid. The spectroscopic data are consistent with previously reported [56]. TLC: *R_f_* = 0.58 (DCM). ^1^H NMR (600 MHz, CDCl_3_) *δ* 8.41 (d, *J* = 8.4 Hz, 1H), 8.32 (d, *J* = 1.8 Hz, 1H), 8.29 (d, *J* = 8.4 Hz, 1H), 7.99 (d, *J* = 9.0, 1.8 Hz, 1H), 7.84 (d, *J* = 8.4 Hz, 1H).

6-Chloroquinoline-2-carbonitrile (**2o**). Following GPA, using 6-chloroquinoline *N*-oxide (100 mg, 0.56 mmol), the title compound was obtained (90 mg, 86% yield) as a white solid. Following GPB, using 6-chloroquinoline *N*-oxide (100 mg, 0.56 mmol), the title compound was obtained (103 mg, 98% yield) as a white solid. The spectroscopic data are consistent with previously reported [39]. TLC: *R_f_* = 0.51 (PE:EA = 10:1). ^1^H NMR (600 MHz, CDCl_3_) *δ* 8.23 (d, *J* = 8.4 Hz, 1H), 8.11 (d, *J* = 9.0 Hz, 1H), 7.89 (d, *J* = 2.4 Hz, 1H),7.78 (dd, *J* = 9.0, 2.4 Hz, 1H), 7.73 (d, *J* = 8.4 Hz, 1H).

6-Bromoquinoline-2-carbonitrile (**2p**). Following GPA, using 6-bromoquinoline *N*-oxide (100 mg, 0.45 mmol), the title compound was obtained (97 mg, 92% yield) as a white solid. Following GPB, using 6-bromoquinoline *N*-oxide (100 mg, 0.45 mmol), the title compound was obtained (100 mg, 95% yield) as a white solid. The spectroscopic data are consistent with previously reported [38]. TLC: *R_f_* = 0.34 (PE:EA = 20:1). ^1^H NMR (600 MHz, CDCl_3_) *δ* 8.23 (d, *J* = 8.4 Hz, 1H), 8.08 (d, *J* = 2.4 Hz, 1H), 8.04 (d, *J* = 9.6 Hz, 1H), 7.91 (dd, *J* = 9.0, 2.4 Hz, 1H), 7.73 (d, *J* = 8.4 Hz, 1H).

7-Methylquinoline-2-carbonitrile (**2q**). Following GPA, using 7-methylquinoline *N*-oxide (100 mg, 0.63 mmol), the title compound was obtained (93 mg, 88% yield) as a white solid. Following GPB, using 7-methylquinoline *N*-oxide (100 mg, 0.63 mmol), the title compound was obtained (87 mg, 82% yield) as a white solid. The spectroscopic data are consistent with previously reported [57]. TLC: *R_f_* = 0.50 (PE:EA = 10:1). ^1^H NMR (600 MHz, CDCl_3_) *δ* 8.21 (d, *J* = 8.4 Hz, 1H), 7.87 (s, 1H), 7.75 (d, *J* = 8.4 Hz, 1H), 7.59 (d, *J* = 8.4 Hz, 1H), 7.50 (dd, *J* = 8.4, 1.2 Hz, 1H), 2.57 (s, 3H).

7-Bromoquinoline-2-carbonitrile (**2r**). Following GPA, using 7-bromoquinoline *N*-oxide (100 mg, 0.45 mmol), the title compound was obtained (89 mg, 85% yield) as a white solid. Following GPB, using 7-bromoquinoline *N*-oxide (100 mg, 0.45 mmol), the title compound was obtained (100 mg, 95% yield) as a white solid. The spectroscopic data are consistent with previously reported [38]. TLC: *R_f_* = 0.30 (PE:EA = 20:1). ^1^H NMR (600 MHz, CDCl_3_) *δ* 8.30 (t, *J* = 8.4 Hz, 2H), 7.77 (s, 2H), 7.71 (d, *J* = 8.4 Hz, 1H).

7-Chloroquinoline-2-carbonitrile (**2s**). Following GPA, using 7-chloroquinoline *N*-oxide (100 mg, 0.56 mmol), the title compound was obtained (85 mg, 81% yield) as a white solid. The spectroscopic data are consistent with previously reported [38]. TLC: *R_f_* = 0.34 (PE:EA = 10:1). ^1^H NMR (600 MHz, CDCl_3_) *δ* 8.30 (d, *J* = 8.4 Hz, 1H), 8.11 (d, *J* = 1.8 Hz, 1H), 7.84 (d, *J* = 9.0 Hz, 1H), 7.69 (d, *J* = 8.4 Hz, 1H), 7.63 (dd, *J* = 9.0, 2.4 Hz, 1H).

8-Methylquinoline-2-carbonitrile (**2t**). Following GPA, using 8-methylquinoline *N*-oxide (100 mg, 0.63 mmol), the title compound was obtained (79 mg, 75% yield) as a white solid. The spectroscopic data are consistent with previously reported [39]. TLC: *R_f_* = 0.68 (PE:EA = 5:1). ^1^H NMR (600 MHz, CDCl_3_) *δ* 8.26 (d, *J* = 8.4 Hz, 1H), 7.73–7.67 (m, 3H), 7.58 (t, *J* = 7.8 Hz, 1H), 2.82 (s, 3H).

6-Bromo-4-chloroquinoline-2-carbonitrile (**2u**). Following GPA, but with the modification that the reaction was conducted at 80 °C, the title compound was obtained. Using 6-bromo-4-chloroquinoline *N*-oxide (100 mg, 0.39 mmol), the reaction afforded the product (75 mg, 72% yield) as a white solid. Following GPB, using 6-bromo-4-chloroquinoline *N*-oxide (100 mg, 0.39 mmol), the title compound was obtained (76 mg, 73% yield) as a white solid. TLC: *R_f_* = 0.67 (PE:EA = 20:1). Melting point: 156.5–157.3 °C. ^1^H NMR (600 MHz, CDCl_3_) *δ* 8.44 (d, *J* = 2.4 Hz, 1H), 8.05 (d, *J* = 9.0 Hz, 1H), 7.96 (dd, *J* = 8.4, 1.8 Hz, 1H), 7.80 (s, 1H). ^13^C {^1^H} NMR (150 MHz, CDCl_3_) *δ* 147.4, 143.0, 135.9, 133.6, 132.1, 128.2, 126.7, 125.7, 124.2, 116.5. HRMS (+ESI-TOF) *m*/*z*: [M + H]^+^ Calcd for C_10_H_5_BrClN_2_ 266.9319; found 266.9321.

4-Chloro-6,7-dimethoxyquinoline-2-carbonitrile (**2v**). Following GPA, but with the modification that the reaction was conducted at 100 °C, the title compound was obtained. Using 4-chloro-6,7-dimethoxyquinoline *N*-oxide (100 mg, 0.42 mmol), the reaction afforded the product (87 mg, 83% yield) as a white solid. The spectroscopic data are consistent with previously reported [58]. TLC: *R_f_* = 0.30 (PE:EA = 10:1). ^1^H NMR (600 MHz, CDCl_3_) *δ* 7.65 (s, 1H), 7.44 (s, 1H), 7.40 (s, 1H), 4.09 (s, 3H), 4.06 (s, 3H).

Isoquinoline-1-carbonitrile (**3a**). Following GPA, using isoquinoline *N*-oxide (100 mg, 0.69 mmol), the title compound was obtained (96 mg, 91% yield) as a white solid. Following GPB, using isoquinoline *N*-oxide (100 mg, 0.69 mmol), the title compound was obtained (101 mg, 95% yield) as a white solid. The spectroscopic data are consistent with previously reported [39]. TLC: *R_f_* = 0.30 (PE:EA = 20:1). ^1^H NMR (600 MHz, CDCl_3_) *δ* 8.65 (d, *J* = 5.4 Hz, 1H), 8.33 (d, *J* = 8.4 Hz, 1H), 7.95 (d, *J* = 7.8 Hz, 1H), 7.90 (d, *J* = 5.4 Hz, 1H), 7.84–7.79 (m, 2H).

3-Methylisoquinoline-1-carbonitrile (**3b**). Following GPA, using 3-methyisoquinoline *N*-oxide (100 mg, 0.63 mmol), the title compound was obtained (86 mg, 81% yield) as a white solid. Following GPB, using 3-methyisoquinoline *N*-oxide (100 mg, 0.63 mmol), the title compound was obtained (100 mg, 94% yield) as a white solid. The spectroscopic data are consistent with previously reported [59]. TLC: *R_f_* = 0.47 (PE:EA = 10:1). ^1^H NMR (600 MHz, CDCl_3_) *δ* 8.25 (d, *J* =7.8 Hz, 1H), 7.83 (d, *J* =8.4 Hz, 1H), 7.75 (t, *J* =7.2 Hz, 1H), 7.71–7.68 (m, 2H), 2.73 (s, 3H).

4-Bromoisoquinoline-1-carbonitrile (**3c**). Following GPA, using 4-bromoisoquinoline *N*-oxide (100 mg, 0.45 mmol), the title compound was obtained (91 mg, 87% yield) as a white solid. Following GPB, using 4-bromoisoquinoline *N*-oxide (100 mg, 0.45 mmol), the title compound was obtained (103 mg, 98% yield) as a white solid. The spectroscopic data are consistent with previously reported [60]. TLC: *R_f_* = 0.52 (PE:EA = 20:1). ^1^H NMR (600 MHz, CDCl_3_) *δ* 8.83 (s, 1H), 8.36 (d, *J* = 8.4 Hz, 1H), 8.27 (d, *J* = 8.4 Hz, 1H), 7.96–7.94 (m, 1H), 7.88 (t, *J* = 7.8 Hz, 1H).

5-Bromoisoquinoline-1-carbonitrile (**3d**). Following GPA, using 5-bromoisoquinoline *N*-oxide (100 mg, 0.45 mmol), the title compound was obtained (90 mg, 86% yield) as a white solid. Following GPB, using 5-bromoisoquinoline *N*-oxide (100 mg, 0.45 mmol), the title compound was obtained (103 mg, 98% yield) as a white solid. The spectroscopic data are consistent with previously reported [61]. TLC: *R_f_* = 0.50 (PE:EA = 15:1). ^1^H NMR (600 MHz, CDCl_3_) *δ* 8.76 (d, *J* = 5.4 Hz, 1H), 8.35 (d, *J* = 8.4 Hz, 1H), 8.26 (d, *J* = 5.4 Hz, 1H), 8.11 (d, *J* = 7.8 Hz, 1H), 7.67 (t, *J* = 7.8 Hz, 1H).

6-Bromoisoquinoline-1-carbonitrile (**3e**). Following GPA, using 6-bromoisoquinoline *N*-oxide (100 mg, 0.45 mmol), the title compound was obtained (99 mg, 94% yield) as a white solid. Following GPB, using 6-bromoisoquinoline *N*-oxide (100 mg, 0.45 mmol), the title compound was obtained (95 mg, 90% yield) as a white solid. The spectroscopic data are consistent with previously reported [62]. TLC: *R_f_* = 0.35 (PE:EA = 20:1). ^1^H NMR (600 MHz, CDCl_3_) *δ* 8.66 (d, *J* = 5.4 Hz, 1H), 8.17 (d, *J* = 9.0 Hz, 1H), 8.11 (d, *J* = 1.2 Hz, 1H), 7.84 (dd, *J* = 9.0, 1.8 Hz, 1H), 7.80 (d, *J* = 5.4 Hz, 1H).

6-Methylisoquinoline-1-carbonitrile (**3f**). Following GPA, using 6-methyisoquinoline *N*-oxide (100 mg, 0.63 mmol), the title compound was obtained (92 mg, 87% yield) as a white solid. Following GPB, using 6-methyisoquinoline *N*-oxide (100 mg, 0.63 mmol), the title compound was obtained (61 mg, 58% yield) as a white solid. The spectroscopic data are consistent with previously reported [63]. TLC: *R_f_* = 0.34 (PE:EA = 10:1). ^1^H NMR (600 MHz, CDCl_3_) *δ* 8.55 (d, *J* = 6.0 Hz, 1H), 8.14 (d, *J* = 8.4 Hz, 1H), 7.76 (d, *J* = 5.4 Hz, 1H), 7.66 (s, 1H), 7.58 (dd, *J* = 9.0, 1.2 Hz, 1H), 2.58 (s, 3H).

7-Bromoisoquinoline-1-carbonitrile (**3g**). Following GPA, using 7-bromoisoquinoline *N*-oxide (100 mg, 0.45 mmol), the title compound was obtained (90 mg, 86% yield) as a white solid. TLC: *R_f_* = 0.39 (PE:EA = 10:1). Melting point: 175.9–176.4 °C. ^1^H NMR (600 MHz, CDCl_3_) *δ* 8.67 (d, *J* = 5.4 Hz, 1H), 8.48 (t, *J* = 1.2 Hz, 1H), 7.90–7.88 (m, 2H), 7.83 (d, *J* = 9.0 Hz, 1H). ^13^C {^1^H} NMR (150 MHz, CDCl_3_) *δ* 143.8, 135.6, 134.5, 133.8, 130.2, 129.0, 127.6, 124.41, 124.36, 115.5. HRMS (+ESI-TOF) *m*/*z*: [M + H]^+^ Calcd for C_10_H_6_BrN_2_ 232.9709; found 232.9709.

7-Methylisoquinoline-1-carbonitrile (**3h**). Following GPA, using 7-methyisoquinoline *N*-oxide (100 mg, 0.63 mmol), the title compound was obtained (94 mg, 89% yield) as a white solid. TLC: *R_f_* = 0.24 (PE:EA = 15:1). Melting point: 83.2–84.0 °C. ^1^H NMR (600 MHz, CDCl_3_) *δ* 8.52 (d, *J* = 5.4 Hz, 1H), 7.99 (s, 1H), 7.81–7.79 (m, 2H), 7.61 (dd, *J* = 8.4, 1.2 Hz, 1H), 2.58 (s, 3H). ^13^C {^1^H} NMR (150 MHz, CDCl_3_) *δ* 142.6, 140.6, 134.3, 134.1, 133.8, 129.6, 127.1, 124.3, 123.8, 116.1, 22.1. HRMS (+ESI-TOF) *m*/*z*: [M + Na]^+^ Calcd for C_11_H_8_N_2_Na 191.0580; found 191.0581.

8-Chlorolisoquinoline-2-carbonitrile (**3i**). Following GPA, using 8-chlorolisoquinoline *N*-oxide (100 mg, 0.56 mmol), the title compound was obtained (96 mg, 91% yield) as a white solid. TLC: *R_f_* = 0.29 (PE:EA = 10:1). Melting point: 168.6–169.2 °C. ^1^H NMR (600 MHz, CDCl_3_) *δ* 8.69 (d, *J* = 5.4 Hz, 1H), 7.91 (d, *J* = 5.4 Hz, 1H), 7.86 (d, *J* = 7.8 Hz, 1H), 7.78 (d, *J* = 7.2 Hz, 1H), 7.69 (t, *J* = 7.8 Hz, 1H). ^13^C {^1^H} NMR (150 MHz, CDCl_3_) *δ* 143.7, 138.4, 132.0, 131.7, 131.4, 130.3, 127.2, 126.3, 124.8, 117.8. HRMS (+ESI-TOF) *m*/*z*: [M + Na]^+^ Calcd for C_10_H_5_ClN_2_Na 211.0033; found 211.0033.

8-Bromoisoquinoline-1-carbonitrile (**3j**). Following GPA, using 8-bromoisoquinoline *N*-oxide (100 mg, 0.45 mmol), the title compound was obtained (92 mg, 88% yield) as a white solid. TLC: *R_f_* = 0.40 (PE:EA = 10:1). Melting point: 164.5–165.2 °C. ^1^H NMR (600 MHz, CDCl_3_) *δ* 8.68 (d, *J* = 5.4 Hz, 1H), 8.03 (dd, *J* = 7.8, 0.6 Hz, 1H), 7.91–7.90 (m, 2H), 7.60 (t, *J* = 7.2 Hz, 1H). ^13^C {^1^H} NMR (150 MHz, CDCl_3_) *δ* 143.6, 138.5, 136.0, 133.3, 131.7, 128.0, 127.1, 125.1, 118.9, 117.6. HRMS (+ESI-TOF) *m*/*z*: [M + H]^+^ Calcd for C_10_H_6_BrN_2_ 232.9709; found 232.9711.

Pyrimidine-2-carbonitrile (**4**). Following GPA, using pyrimidine *N*-oxide (100 mg, 1.05 mmol), the title compound was obtained (57 mg, 52% yield) as a white solid. The spectroscopic data are consistent with previously reported [64]. TLC: *R_f_* = 0.40 (PE:EA = 1:1). ^1^H NMR (600 MHz, CDCl_3_) *δ* 8.87 (d, *J* = 4.8 Hz, 2H), 7.56 (t, *J* = 4.8 Hz, 1H).

1,5-Naphthyridine-2-carbonitrile (**5**). Following GPA, using 1,5-naphthyridine 1-oxide (100 mg, 0.68 mmol), the title compound was obtained (80 mg, 76% yield) as a white solid. The spectroscopic data are consistent with previously reported [39]. TLC: *R_f_* = 0.29 (PE:EA = 5:1). ^1^H NMR (600 MHz, CDCl_3_) *δ* 9.11 (dd, *J* = 4.2, 1.8 Hz, 1H), 8.56 (dd, *J* = 8.4, 0.6 Hz, 1H), 8.48–8.46 (m, 1H), 7.94 (d, *J* = 8.4 Hz, 1H), 7.77 (dd, *J* = 8.4, 3.6 Hz, 1H).

Quinoxaline-2-carbonitrile (**6**). Following GPA, but with the modification that the reaction was conducted at 80 °C, the title compound was obtained. Using quinoxaline 1-oxide (100 mg, 0.68 mmol), the reaction afforded the product (60 mg, 57% yield) as a white solid. The spectroscopic data are consistent with previously reported [65]. TLC: *R_f_* = 0.44 (PE:EA = 5:1). ^1^H NMR (600 MHz, CDCl_3_) *δ* 9.07 (s, 1H), 8.19–8.16 (m, 2H), 7.97–7.91 (m, 2H).

Quinoxaline-2,3-dicarbonitrile (**7**). To a solution of quinoxaline 1,4-dioxide (100 mg, 0.62 mmol) in dry EA (0.6 mL), TMSCN (368 mg, 3.72 mmol) and DIEA (320 mg, 2.48 mmol) were added, and the reaction mixture was then heated to 80 °C and stirred until the reaction was complete as indicated by TLC. After cooling down to room temperature, the reaction was quenched with saturated sodium bicarbonate solution. The mixture was extracted with EA (3 × 2 mL). The combined organic phases were dried over Na_2_SO_4_ and concentrated in vacuo to give the crude product. Purification by flash column chromatography (PE:EA = 20:1) furnished the desired pure product (12 mg, 11% yield) as a yellow solid. The spectroscopic data are consistent with previously reported [66]. TLC: *R_f_* = 0.20 (PE:EA = 5:1). ^1^H NMR (600 MHz, CDCl_3_) *δ* 8.30–8.27 (m, 2H), 8.14–8.11 (m, 2H).

5-Oxo-6,7,8,9-tetrahydro-5*H*-chromeno [2,3-b]pyridine-2-carbonitrile (**8**). Following GPA, but with the modification that the reaction was conducted at 80 °C, the title compound was obtained. Using 5-oxo-6,7,8,9-tetrahydro-5*H*-chromeno[2,3-b]pyridine 1-oxide (100 mg, 0.46 mmol), the reaction afforded the product (55 mg, 53% yield) as a white solid. TLC: *R_f_* = 0.67 (PE:EA = 1:1). Melting point: 179.8–180.5 °C. ^1^H NMR (600 MHz, CDCl_3_) *δ* 8.67 (d, *J* = 7.8 Hz, 1H), 7.74 (d, *J* = 7.8 Hz, 1H), 2.75 (t, *J* = 6.0 Hz, 2H), 2.54 (t, *J* = 6.0 Hz, 2H), 1.91–1.89 (m, 2H), 1.78–1.76 (m, 2H). ^13^C NMR (150 MHz, CDCl_3_) *δ* 176.6, 165.8, 159.7, 138.3, 135.0, 125.6, 120.2, 120.1, 116.0, 28.2, 21.7, 21.2, 21.1. HRMS (+ESI-TOF) *m*/*z*: [M + H]^+^ Calcd for C_13_H_11_N_2_O_2_ 227.0815; found 227.0814.

1,10-Phenanthroline-2-carbonitrile (**9**). Following GPA, using 1,10-phenanthroline 1-oxide (100 mg, 0.51 mmol), the reaction afforded the product (83 mg, 79% yield) as a white solid. The spectroscopic data are consistent with previously reported [67]. TLC: *R_f_* = 0.59 (DCM:MeOH: NH_3_·H_2_O= 50: 1: 0.5). ^1^H NMR (600 MHz, CDCl_3_) *δ* 9.23 (dd, *J* = 4.2, 1.2 Hz, 1H), 8.36 (d, *J* = 8.4 Hz, 1H), 8.27 (dd, *J* = 8.4, 1.8 Hz, 1H), 7.93–7.91 (m, 2H), 7.81 (d, *J* = 9.0 Hz, 1H), 7.70 (dd, *J* = 7.8, 4.2 Hz, 1H).

Thieno[2,3-b]pyridine-6-carbonitrile (**10**). Following GPA, but with the modification that the reaction was conducted at 80 °C, the title compound was obtained. Using thieno[2,3-b]pyridine 7-oxide (100 mg, 0.66 mmol), the reaction afforded the product (72 mg, 68% yield) as a white solid. The spectroscopic data are consistent with previously reported [68]. TLC: *R_f_* = 0.51 (PE:EA = 5:1). ^1^H NMR (600 MHz, CDCl_3_) *δ* 8.18 (d, *J* =8.4 Hz, 1H), 7.81 (d, *J* = 6.0 Hz, 1H), 7.64 (d, *J* = 7.8 Hz, 1H), 7.37 (d, *J* = 5.4 Hz, 1H).

1H-Pyrrolo[3,2-b]pyridine-5-carbonitrile (**11**). Following GPA, but with the modification that the reaction was conducted at 80 °C, the title compound was obtained. Using 1H-pyrrolo[3,2-b]pyridine 4-oxide (100 mg, 0.75 mmol), the reaction afforded the product (53 mg, 49% yield) as a white solid. The spectroscopic data are consistent with previously reported [69]. TLC: *R_f_* = 0.42 (PE:EA = 1:1). ^1^H NMR (600 MHz, DMSO-*d_6_*) *δ* 11.88 (s, 1H), 7.97 (dd, *J* =8.4, 0.6 Hz, 1H), 7.93 (d, *J* = 3.0 Hz, 1H), 7.66 (d, *J* = 8.4 Hz, 1H), 6.71 (d, *J* = 3.0 Hz, 1H).

Picolinonitrile (**13a**). Following GPC, using pyridine *N*-oxide (100 mg, 1.05 mmol), the title compound was obtained (79 mg, 72% yield) as a yellow oil. Following GPD, using pyridine *N*-oxide (100 mg, 1.05 mmol), the title compound was obtained (90 mg, 82% yield) as a yellow oil. The spectroscopic data are consistent with previously reported [38]. TLC: *R_f_* = 0.35 (PE:EA = 10:1). ^1^H NMR (600 MHz, CDCl_3_) *δ* 8.72 (d, *J* = 4.8 Hz, 1H), 7.86–7.83 (m, 1H), 7.70 (dd, *J* = 7.8, 1.2 Hz, 1H), 7.54–7.52 (m, 1H).

4-Methylpicolinonitrile (**13b**). Following GPC, but with the modification that the reaction was conducted at 80 °C, the title compound was obtained. Using 4-methylpyridine *N*-oxide (100 mg, 0.92 mmol), the reaction afforded the product (46 mg, 42% yield) as a white solid. Following GPD, using 4-methylpyridine *N*-oxide (100 mg, 0.92 mmol), the title compound was obtained (58 mg, 53% yield) as a white solid. The spectroscopic data are consistent with previously reported [70]. TLC: *R_f_* = 0.32 (PE:EA = 5:1). ^1^H NMR (600 MHz, CDCl_3_) *δ* 8.56 (d, *J* = 4.8 Hz, 1H), 7.52 (s, 1H), 7.33 (dd, *J* = 6.0, 4.8 Hz, 1H), 2.43 (s, 3H).

4-(*Tert*-butyl)picolinonitrile (**13c**). Following GPC, but with the modification that the reaction was conducted at 80 °C, the title compound was obtained. Using 4-(*tert*-butyl)pyridine *N*-oxide (100 mg, 0.66 mmol), the reaction afforded the product (87 mg, 82% yield) as a yellow oil. Following GPD, using 4-(*tert*-butyl)pyridine *N*-oxide (100 mg, 0.66 mmol), the title compound was obtained (49 mg, 46% yield) as a yellow oil. The spectroscopic data are consistent with previously reported [24]. TLC: *R_f_* = 0.50 (PE:EA = 5:1). ^1^H NMR (600 MHz, CDCl_3_) *δ* 8.59 (d, *J* = 4.8 Hz, 1H), 7.68 (d, *J* = 1.8 Hz, 1H), 7.48 (dd, *J* = 4.8, 1.8 Hz, 1H), 1.32 (s, 9H).

Pyridine-2,4-dicarbonitrile (**13d**). Following GPC, but with the modification that the reaction was conducted at 80 °C, the title compound was obtained. Using 4-cyanopyridine *N*-oxide (100 mg, 0.83 mmol), the reaction afforded the product (19 mg, 18% yield) as a white solid. Following GPD, but with the modification that the reaction was conducted at 80 °C, the title compound was obtained. Using 4-cyanopyridine *N*-oxide (100 mg, 0.83 mmol), the reaction afforded the product (84 mg, 78% yield) as a white solid. The spectroscopic data are consistent with previously reported [24]. TLC: *R_f_* = 0.34 (PE:EA = 5:1). ^1^H NMR (600 MHz, CDCl_3_) *δ* 8.93 (d, *J* = 4.8 Hz, 1H), 7.94 (s, 1H), 7.79 (dd, *J* = 4.8, 1.2 Hz, 1H).

Methyl 2-cyanoisonicotinate (**13e**). Following GPD, using 4-(methoxycarbonyl)pyridine *N*-oxide (100 mg, 0.65 mmol), the title compound was obtained (15 mg, 14% yield) as a white solid. Following GPD, with the modification that the reaction was conducted at 80 °C, using 4-(methoxycarbonyl)pyridine *N*-oxide (100 mg, 0.65 mmol), the title compound was obtained (91 mg, 86% yield) as a white solid. The spectroscopic data are consistent with previously reported [24]. TLC: *R_f_* = 0.45 (PE:EA = 5:1). ^1^H NMR (600 MHz, CDCl_3_) *δ* 8.87 (dd, *J* = 4.8, 0.6 Hz, 1H), 8.22–8.21 (m, 1H), 8.06 (dd, *J* = 4.8, 1.8 Hz, 1H), 3.99 (s, 3H).

3-Methylpicolinonitrile (**13f**). Following GPC, using 3-methylpyridine *N*-oxide (100 mg, 0.92 mmol), the title compound was obtained (67 mg, 62% yield) as a white solid. Following GPD, using 3-methylpyridine *N*-oxide (100 mg, 0.92 mmol), the title compound was obtained (75 mg, 69% yield) as a white solid. The spectroscopic data are consistent with previously reported [24]. TLC: *R_f_* = 0.30 (PE:EA = 5:1). ^1^H NMR (600 MHz, CDCl_3_) *δ* 8.54 (d, *J* = 3.6 Hz, 1H), 7.67 (dd, *J* = 7.8, 0.6 Hz, 1H), 7.41 (dd, *J* = 8.4, 4.8 Hz, 1H), 2.57 (s, 3H).

5-Phenylpicolinonitrile (**13g**). Following GPC, using 3-phenylpyridine *N*-oxide (100 mg, 0.58 mmol), 5-phenylpicolinonitrile (**13g**) and 3-phenylpicolinonitrile (**13g′**) were obtained. The title compound was obtained (19 mg, 18% yield) as a white solid. Following GPD, using 3-phenylpyridine *N*-oxide (100 mg, 0.58 mmol), **13g** and **13g′** were obtained. The title compound was obtained (39 mg, 37% yield). The spectroscopic data are consistent with previously reported [24]. TLC: *R_f_* = 0.58 (PE:EA = 5:1). ^1^H NMR (600 MHz, CDCl_3_) *δ* 8.95 (t, *J* = 0.6 Hz, 1H), 8.01 (dd, *J* = 7.8, 1.8 Hz, 1H), 7.78 (dd, *J* = 7.8, 0.6 Hz, 1H), 7.61–7.59 (m, 2H), 7.55–7.52 (m, 2H),7.50–7.47 (m, 1H).

3-Phenylpicolinonitrile (**13g′**). Following GPC, the title compound was obtained (49 mg, 47% yield) as a white solid. Following GPD, the title compound was obtained (59 mg, 57% yield). The spectroscopic data are consistent with previously reported [24]. TLC: *R_f_* = 0.37 (PE:EA = 5:1). ^1^H NMR (600 MHz, CDCl_3_) *δ* 8.69 (dd, *J* = 4.8, 1.8 Hz, 1H), 7.86 (dd, *J* = 8.4, 1.8 Hz, 1H), 7.59–7.56 (m, 3H), 7.54–7.48 (m, 2H).

5-Chloropicolinonitrile (**13h**). Following GPC, using 3-chloropyridine *N*-oxide (100 mg, 0.77 mmol), 5-chloropicolinonitrile (**13h**) and 3-chloropicolinonitrile (**13h′**) were obtained. The title compound was obtained (4 mg, 4% yield) as a white solid. The spectroscopic data are consistent with previously reported [24]. TLC: *R_f_* = 0.50 (PE:EA = 5:1). ^1^H NMR (600 MHz, CDCl_3_) *δ* 8.68 (d, *J* = 2.4 Hz, 1H), 7.83 (dd, *J* = 8.4, 2.4 Hz, 1H), 7.66 (d, *J* = 7.8 Hz, 1H).

3-Chloropicolinonitrile (**13h′**). The title compound was obtained (86 mg, 80% yield) as a white solid. The spectroscopic data are consistent with previously reported [24]. TLC: *R_f_* = 0.34 (PE:EA = 5:1). ^1^H NMR (600 MHz, CDCl_3_) *δ* 8.62 (dd, *J* = 4.8, 1.8 Hz, 1H), 7.87 (dd, *J* = 8.4, 1.2 Hz, 1H), 7.50 (dd, *J* = 8.4, 4.8 Hz, 1H).

5-Bromopicolinonitrile (**13i**). Following GPC, using 3-bromopyridine *N*-oxide (100 mg, 0.58 mmol), 5-bromopicolinonitrile (**13i**) and 3-bromopicolinonitrile (**13i′**) were obtained. The title compound was obtained (9 mg, 8% yield) as a white solid. Following GPD, using 3-bromopyridine *N*-oxide (100 mg, 0.58 mmol), **13i** and **13i′** were obtained. The title compound was obtained (11 mg, 10% yield). The spectroscopic data are consistent with previously reported [24]. TLC: *R_f_* = 0.45 (PE:EA = 10:1). ^1^H NMR (600 MHz, CDCl_3_) *δ* 8.79 (d, *J* = 1.8 Hz, 1H), 8.00 (dd, *J* = 8.4, 2.4 Hz, 1H), 7.60 (dd, *J* = 7.8, 0.6 Hz, 1H).

3-Bromopicolinonitrile (**13i′**). Following GPC, the title compound was obtained (86 mg, 81% yield) as a white solid. Following GPD, the title compound was obtained (78 mg, 74% yield). The spectroscopic data are consistent with previously reported [24]. TLC: *R_f_* = 0.31 (PE:EA = 10:1). ^1^H NMR (600 MHz, CDCl_3_) *δ* 8.65 (dd, *J* = 4.8, 1.2 Hz, 1H), 8.03 (dd, *J* = 7.8, 1.2 Hz, 1H), 7.42 (q, *J* = 4.2 Hz,1H).

Methyl 6-cyanonicotinate (**13j**). Following GPC, using 3-(methoxycarbonyl)pyridine *N*-oxide (100 mg, 0.65 mmol), methyl 6-cyanonicotinate (**13j**) and methyl 2-cyanonicotinate (**13j′**) were obtained. The title compound was obtained (57 mg, 54% yield) as a white solid. Following GPD, using 3-(methoxycarbonyl)pyridine *N*-oxide (100 mg, 0.65 mmol), **13j** and **13j′** were obtained. The title compound was obtained (76 mg, 72% yield). The spectroscopic data are consistent with previously reported [24]. TLC: *R_f_* = 0.31 (PE:EA = 5:1). ^1^H NMR (600 MHz, CDCl_3_) *δ* 9.25 (t, *J* = 0.6 Hz, 1H), 8.42 (dd, *J* = 7.8, 1.8 Hz, 1H), 7.79 (dd, *J* = 7.8, 0.6 Hz, 1H), 3.98 (s, 3H).

Methyl 2-cyanonicotinate (**13j′**). Following GPC, using 3-(methoxycarbonyl)pyridine *N*-oxide (100 mg, 0.65 mmol), the title compound was obtained (24 mg, 23% yield) as a white solid. Following GPD, the title compound was obtained (20 mg, 19% yield). The spectroscopic data are consistent with previously reported [24]. TLC: *R_f_* = 0.28 (PE:EA = 5:1). ^1^H NMR (600 MHz, CDCl_3_) *δ* 8.87 (dd, *J* = 4.8, 1.2 Hz, 1H), 8.43 (dd, *J* = 8.4, 1.8 Hz, 1H), 7.63 (dd, *J* = 7.8, 4.8 Hz, 1H), 4.05 (s, 3H).

5-(Trifluoromethyl)picolinonitrile (**13k**). Following GPC, using 3-(trifluoromethyl)pyridine *N*-oxide (100 mg, 0.61 mmol), 5-(trifluoromethyl)picolinonitrile (**13k**) and 3-(trifluoromethyl)picolinonitrile (**13k′**) were obtained. The title compound was obtained (18 mg, 17% yield) as a yellow oil. Following GPD, using 3-phenylpyridine *N*-oxide (100 mg, 0.58 mmol), **13k** and **13k′** were obtained. The title compound was obtained (10 mg, 10% yield). The spectroscopic data are consistent with previously reported [71]. TLC: *R_f_* = 0.55 (PE:EA = 10:1). ^1^H NMR (600 MHz, CDCl_3_) *δ* 8.98 (t, *J* = 0.6 Hz, 1H), 8.12 (dd, *J* = 8.4, 1.8 Hz, 1H), 7.87 (d, *J* = 8.4 Hz, 1H).

3-(Trifluoromethyl)picolinonitrile (**13k′**). Following GPC, using 3-(trifluoromethyl)pyridine *N*-oxide (100 mg, 0.61 mmol), the title compound was obtained (38 mg, 36% yield) as a yellow oil. Following GPD, the title compound was obtained (29 mg, 28% yield). The spectroscopic data are consistent with previously reported [72]. TLC: *R_f_* = 0.39 (PE:EA = 5:1). ^1^H NMR (600 MHz, CDCl_3_) *δ* 8.91 (dd, *J* = 4.8, 1.2 Hz, 1H), 8.15 (dd, *J* = 8.4, 1.2 Hz, 1H), 7.73–7.71 (m, 1H).

5-Methoxypicolinonitrile (**13l**). Following GPC, using 3-methoxypyridine *N*-oxide (100 mg, 0.80 mmol), the title compound was obtained (72 mg, 67% yield) as a white solid. Following GPD, using 3-methoxypyridine *N*-oxide (100 mg, 0.80 mmol), the title compound was obtained (101 mg, 94% yield) as a white solid. The spectroscopic data are consistent with previously reported [73]. TLC: *R_f_* = 0.58 (PE:EA = 3:1). ^1^H NMR (600 MHz, CDCl_3_) *δ* 8.26 (dd, *J* = 4.2, 1.2 Hz, 1H), 7.49 (q, *J* = 4.2 Hz, 1H), 7.37 (dd, *J* = 8.4, 1.2 Hz, 1H), 3.96 (s, 3H).

6-Chloropicolinonitrile (**13m**). Following GPC, but with the modification that the reaction was conducted at 80 °C, the title compound was obtained. Using 2-chloropyridine *N*-oxide (100 mg, 0.78 mmol), the reaction afforded the product (20 mg, 19% yield) as a white solid. Following GPD, using 2-chloropyridine *N*-oxide (100 mg, 0.78 mmol), the title compound was obtained (60 mg, 56% yield) as a white solid. The spectroscopic data are consistent with previously reported [74]. TLC: *R_f_* = 0.46 (PE:EA = 5:1). ^1^H NMR (600 MHz, CDCl_3_) *δ* 7.83 (t, *J* = 7.8 Hz, 1H), 7.65 (dd, *J* = 7.8, 0.6 Hz, 1H), 7. 58 (dd, *J* = 7.8, 0.6 Hz, 1H).

6-Phenylpicolinonitrile (**13n**). Following GPC, but with the modification that the reaction was conducted at 80 °C, the title compound was obtained. Using 2-phenylpyridine *N*-oxide (100 mg, 0.59 mmol), the reaction afforded the product (80 mg, 76% yield) as a yellow solid. Following GPD, using 2-phenylpyridine *N*-oxide (100 mg, 0.59 mmol), the title compound was obtained (57 mg, 54% yield) as a yellow solid. The spectroscopic data are consistent with previously reported [24]. TLC: *R_f_* = 0.53 (PE:EA = 5:1). ^1^H NMR (600 MHz,CDCl_3_) *δ* 8.02 (d, *J* = 6.6 Hz, 2H), 7.94 (d, *J* = 7.8 Hz, 1H), 7.88 (t, *J* = 7.8 Hz, 1H), 7.62 (d, *J* = 7.8 Hz, 1H), 7.52–7.48 (m, 3H).

Diethyl 2-cyanopyridine-3,5-dicarboxylate (**13o**). Following GPC, using 3,5-bis(ethoxycarbonyl)pyridine *N*-oxide (100 mg, 0.42 mmol), the title compound was obtained (65 mg, 62% yield) as a yellow oil. Following GPD, using 3,5-bis(ethoxycarbonyl)pyridine *N*-oxide (100 mg, 0.42 mmol), the title compound was obtained (93 mg, 89% yield) as a yellow oil. TLC: *R_f_* = 0.52 (PE:EA = 5:1). ^1^H NMR (600 MHz, CDCl_3_) *δ* 9.36 (d, *J* = 2.4 Hz, 1H), 8.94 (d, *J* = 1.8 Hz, 1H), 4.52 (q, *J* = 7.2 Hz, 2H), 4.47 (q, *J* = 7.2 Hz, 2H), 1.47 (t, *J* = 7.2 Hz, 3H), 1.44 (t, *J* = 7.2 Hz, 3H). ^13^C {^1^H} NMR (150 MHz, CDCl_3_) *δ* 163.1, 162.3, 153.8, 139.7, 136.3, 130.0, 128.7, 115.6, 63.4, 62.8, 14.3, 14.1. HRMS (+ESI-TOF) *m*/*z*: [M + H]^+^ Calcd for C_12_H_13_N_2_O_4_ 249.0870; found 249.0871.

5-Bromo-3-methylpicolinonitrile (**13p**). Following GPC, using 3-bromo-5-methylpyridine *N*-oxide (100 mg, 0.53 mmol), 5-bromo-3-methylpicolinonitrile (**13p**) and 3-bromo-5-methylpicolinonitrile (**13p′**) were obtained. The title compound was obtained (39 mg, 37% yield) as a white solid. Following GPD, using 3-bromo-5-methylpyridine *N*-oxide (100 mg, 0.53 mmol), **13p** and **13p′** were obtained. The title compound was obtained (57 mg, 55% yield). The spectroscopic data are consistent with previously reported [75]. TLC: *R_f_* = 0.52 (PE:EA = 10:1). ^1^H NMR (600 MHz, CDCl_3_) *δ* 8.59 (d, *J* = 1.8 Hz, 1H), 7.85 (q, *J* = 0.6 Hz, 1H), 2.55 (s, 3H).

3-Bromo-5-methylpicolinonitrile (**13p′**). Following GPC, the title compound was obtained (15 mg, 14% yield) as a white solid. Following GPD, the title compound was obtained (28 mg, 27% yield). The spectroscopic data are consistent with previously reported [76]. TLC: *R_f_* = 0.31 (PE:EA = 10:1). ^1^H NMR (600 MHz, CDCl_3_) *δ* 8.46 (s, 1H), 7.84 (d, *J* = 0.6 Hz, 1H), 2.43 (s, 3H).

6-Bromo-3-chloropicolinonitrile (**13q**). Following GPC, using 2-bromo-5-chloropyridine *N*-oxide (100 mg, 0.48 mmol), the title compound was obtained (49 mg, 47% yield) as a white solid. Following GPD, using 2-bromo-5-chloropyridine *N*-oxide (100 mg, 0.48 mmol), the title compound was obtained (74 mg, 71% yield) as a white solid. TLC: *R_f_* = 0.43 (PE:EA = 5:1). Melting point: 99.6–100.4 °C. ^1^H NMR (600 MHz, CDCl_3_) *δ* 7.72 (d, *J* = 8.4 Hz, 1H), 7.66 (d, *J* = 8.4 Hz, 1H). ^13^C {^1^H} NMR (150 MHz, CDCl_3_) *δ* 140.3, 139.7, 135.8, 133.1, 132.8, 113.6. HRMS (+ESI-TOF) *m*/*z*: [M + H]^+^. Calcd for C_6_H_3_BrClN_2_ 216.9163; found 216.9163.

5,6-Dichloropicolinonitrile (**13r**). Following GPC, but with the modification that the reaction was conducted at 80 °C, the title compound was obtained. Using 2,3-dichloropyridine *N*-oxide (100 mg, 0.61 mmol), the reaction afforded the product (7 mg, 7% yield) as a white solid. Following GPD, using 2,3-dichloropyridine *N*-oxide (100 mg, 0.61 mmol), the title compound was obtained (12 mg, 11% yield) as a white solid. The spectroscopic data are consistent with previously reported [77]. TLC: *R_f_* = 0.59 (PE:EA = 5:1). ^1^H NMR (600 MHz, CDCl_3_) *δ* 7.94 (d, *J* = 8.4 Hz, 1H), 7.61 (d, *J* = 7.8 Hz, 1H).

3,5-Dibromopicolinonitrile (**13s**). Following GPC, using 3,5-dibromopyridine *N*-oxide (100 mg, 0.40 mmol), the title compound was obtained (94 mg, 90% yield) as a white solid. Following GPD, using 3,5-dibromopyridine *N*-oxide (100 mg, 0.40 mmol), the title compound was obtained (99 mg, 95% yield) as a white solid. The spectroscopic data are consistent with previously reported [78]. TLC: *R_f_* = 0.72 (PE:EA = 5:1). ^1^H NMR (600 MHz, CDCl_3_) *δ* 8.70 (d, *J* = 1.8 Hz, 1H), 8.21 (d, *J* = 1.8 Hz, 1H).

6-Cyano-*N*,*N*-diethylnicotinamide (**14**). Following GPD, but with the modification that the reaction was conducted at 80 °C, the title compound was obtained. Using 3-(diethylcarbamoyl)pyridine *N*-oxide (100 mg, 0.52 mmol), 6-cyano-*N*,*N*-diethylnicotinamide (**14**) and 2-cyano-N,N-diethylnicotinamide (**14′**) were obtained. The reaction afforded the product (44 mg, 42% yield) as a yellow oil. The spectroscopic data are consistent with previously reported [79]. TLC: *R_f_* = 0.26 (PE:EA = 3:1). ^1^H NMR (600 MHz, CDCl_3_) *δ* 8.70 (dd, *J* = 1.8, 0.6 Hz, 1H), 7.84 (dd, *J* = 7.8, 1.8 Hz, 1H), 7.75 (dd, *J* = 7.8, 0.6 Hz, 1H), 3.55 (d, *J* = 7.2 Hz, 2H), 3.22 (d, *J* = 6.6 Hz, 2H), 1.25 (t, *J* = 6.6 Hz, 3H), 1.14 (t, *J* = 6.6 Hz, 3H).

2-Cyano-*N*,*N*-diethylnicotinamide (**14′**). The reaction afforded the product (56 mg, 53% yield) as a yellow oil. The spectroscopic data are consistent with previously reported [79]. TLC: *R_f_* = 0.18 (PE:EA = 3:1). ^1^H NMR (600 MHz, CDCl_3_) *δ* 8.70 (dd, *J* = 4.8, 1.2 Hz, 1H), 7.76 (dd, *J* = 7.8, 1.8 Hz, 1H), 7.56 (dd, *J* = 8.4, 4.8 Hz, 1H), 3.58 (q, *J* = 7.2 Hz, 2H), 3.20 (q, *J* = 7.2 Hz, 2H), 1.27 (t, *J* = 7.2 Hz, 3H), 1.10 (t, *J* = 7.2 Hz, 3H).

*N*-((2-Cyanopyridin-4-yl)methyl)-*N*-ethyl-3-hydroxy-2-phenylpropanamide (**15**). To a solution of 4-((*N*-ethyl-3-hydroxy-2-phenylpropanamido)methyl)pyridine 1-oxide (100 mg, 0.33 mmol) in dry DCE (1.3 mL), TMSCN (99 mg, 1.0 mmol) and TMG (77 mg, 0.67 mmol) were added. The reaction mixture was stirred for 48 h and the progress was monitored by TLC. Upon completion, the reaction was quenched with a saturated sodium bicarbonate solution. The mixture was extracted with DCM (3 × 2 mL). The combined organic layers were dried over Na_2_SO_4_, filtered, and concentrated under reduced pressure to give the crude product. Purification by flash column chromatography (eluent: PE/EA from 5:1 to 3:1) afforded the desired product (52 mg, 51% yield) as a yellow oil, isolated as a 3.5:1 mixture of rotary isomers. TLC: *R_f_* = 0.42 (DCM:MeOH= 50:1). ^1^H NMR (600 MHz, CDCl_3_) *δ* 8.59 (d, *J* = 4.8 Hz, 1H), 8.55 (d, *J* = 4.8 Hz, 0.28H), 7.42–7.39 (m, 3H), 7.35–7.32 (m, 1H), 7.29–7.27 (m, 4H), 7.16–7.10 (m, 1H), 4.82 (d, *J* = 16.2 Hz, 1H), 4.45 (s, 0.58H), 4.39 (d, *J* = 16.2 Hz, 1H), 4.13 (dd, *J* = 10.8, 9.0 Hz, 1H), 4.05 (q, *J* = 4.2 Hz, 1H), 3.81–3.76 (m, 1H), 3.71 (dd, *J* = 11.4, 4.2 Hz, 0.33H), 3.45–3.35 (m, 2H), 3.17–3.11 (m, 1H), 2.88 (s, 1H), 1.11 (t, *J* = 7.2 Hz, 0.92H), 0.98 (t, *J* = 7.2 Hz, 3H). ^13^C {^1^H} NMR (100 MHz, CDCl_3_) *δ* 173.2, 172.7, 151.3, 151.2, 149.0, 147.9, 135.8, 135.4, 134.4, 134.3, 129.48, 129.45, 128.2, 128.13, 128.1, 126.8, 126.2, 125.3, 124.5, 117.2, 65.8, 52.4, 52.0, 49.0, 47.6, 42.9, 41.5, 29.8, 13.8, 12.4. HRMS (+ESI-TOF) *m*/*z*: [M + Na]^+^ Calcd for C_18_H_19_N_3_O_2_Na 332.1369; found 332.1370.

Heptan-2-yl 2-((5-chloro-2-cyanoquinolin-8-yl)oxy)acetate (**16**). Following GPA, using 5-chloro-8-(2-(heptan-2-yloxy)-2-oxoethoxy)quinoline 1-oxide (100 mg, 0.28 mmol), the title compound was obtained (73 mg, 72% yield) as a white solid. Following GPB, using 5-chloro-8-(2-(heptan-2-yloxy)-2-oxoethoxy)quinoline 1-oxide (100 mg, 0.28 mmol), the title compound was obtained (85 mg, 84% yield) as a white solid. The spectroscopic data are consistent with previously reported [58]. TLC: *R_f_* = 0.37 (PE:EA = 15:1). ^1^H NMR (600 MHz, CDCl_3_) *δ* 8.69 (d, *J* = 8.4 Hz, 1H), 7.84 (d, *J* = 8.4 Hz, 1H), 7.65 (d, *J* = 8.4 Hz, 1H), 7.01 (d, *J* = 9.0 Hz, 1H), 5.05–5.00 (m, 1H), 4.97 (d, *J* = 3.0 Hz, 1H), 1.59–1.55 (m, 1H), 1.49–1.44 (m, 1H), 1.24–1.22 (m, 9H), 0.86–0.83 (m, 3H).

5,5′-Dimethyl-[2,2′-bipyridine]-6-carbonitrile (**17**). Following GPD, using 5,5′-dimethyl-[2,2′-bipyridine] 1-oxide (100 mg, 0.50 mmol), the title compound was obtained (58 mg, 55% yield) as a white solid. TLC: *R_f_* = 0.32 (PE:EA = 5:1). ^1^H NMR (600 MHz, CDCl_3_) *δ* 8.51–8.48 (m, 2H), 8.32 (d, *J* = 7.8 Hz, 1H), 7.75 (d, *J* = 8.4 Hz, 1H), 7.64 (dd, *J* = 8.4, 1.8 Hz, 1H), 2.60 (s, 3H), 2.40 (s, 3H). ^13^C {^1^H} NMR (150 MHz, CDCl_3_) *δ* 155.5, 151.9, 149.8, 139.0, 138.0, 137.8, 134.5, 133.3, 123.8, 120.9, 116.8, 18.6. HRMS (+ESI-TOF) *m*/*z*: [M + H]^+^ Calcd for C_13_H_12_N_3_ 210.1026; found 210.1025.

4-(8-Chloro-2-cyano-5,6-dihydro-11H-benzo[5,6]cyclohepta[1,2-b]pyridin-11-ylidene)piperidine-1-carboxylate (**18**). Following GPD, but with the modification that the reaction was conducted at 80 °C, the title compound was obtained. Using 8-chloro-11-(1-(ethoxycarbonyl)piperidin-4-ylidene)-6,11-dihydro-5*H*-benzo[5,6]cyclohepta[1,2-b]pyridine 1-oxide (100 mg, 0.25 mmol), the title compound was obtained (56 mg, 55% yield) as a white solid. The spectroscopic data are consistent with previously reported [80]. TLC: *R_f_* = 0.31 (PE:EA = 1:1). ^1^H NMR (600 MHz, CDCl_3_) *δ* 7.57 (d, *J* = 7.8 Hz, 1H), 7.50 (d, *J* = 7.8 Hz, 1H), 7.17 (dd, *J* = 6.6, 2.4 Hz, 2H), 7.13 (dd, *J* = 7.2, 2.4 Hz, 1H), 4.15 (q, *J* = 7.2 Hz, 2H), 3.81–3.74 (m, 2H), 3.44–3.38 (m, 2H), 3.25–3.21 (m, 2H), 2.96–2.91 (m, 1H), 2.83–2.79 (m, 1H), 2.76 (s, 1H), 2.53–2.48 (m, 1H), 2.41–2.36 (m, 1H), 2.31–2.27 (m, 1H), 2.25–2.21 (m, 1H), 1.57 (s, 1H), 1.26 (t, *J* = 7.2 Hz, 3H).

4-((1*R*)-Hydroxy((1S,4S)-5-vinylquinuclidin-2-yl)methyl)-6-methoxyquinoline-2-carbonitrile (**19**). Following GPB, using 4-((1*R*)-hydroxy((1*S*,4*S*)-5-vinylquinuclidin-2-yl)methyl)-6-methoxyquinoline 1-oxide. (100 mg, 0.29 mmol), the title compound was obtained (65 mg, 64% yield) as a white solid. TLC: *R_f_* = 0.42 (DCM:MeOH: NH_3_·H_2_O= 20:1:0.5). ^1^H NMR (600 MHz, CDCl_3_) *δ* 8.00 (d, *J* = 3.6 Hz, 1H), 7.80 (s, 1H), 7.42 (dd, *J* = 9.0, 2.4 Hz, 1H), 7.24 (d, *J* = 2.4 Hz, 1H), 5.78–5.72 (m, 1H), 5.48 (d, *J* = 4.8 Hz, 1H), 4.99–4.94 (m, 2H), 3.92 (s, 3H), 3.44 (s, 1H), 3.33–3.29 (m, 1H), 3.10 (dd, *J* = 13.8, 9.0 Hz, 1H), 3.05 (dd, *J* = 13.8, 7.2 Hz, 1H), 2.66–2.61 (m, 2H), 2.29–2.27 (m, 1H), 1.84 (d, *J* = 3.0 Hz, 1H), 1.72–1.66 (m, 2H), 1.63–1.58 (m, 1H), 1.53–1.48 (m, 1H). ^13^C {^1^H} NMR (100 MHz, CDCl_3_) *δ* 160.0, 149.4, 144.6, 141.7, 132.4, 130.6, 127.9, 123.6, 121.3, 118.2, 114.7, 101.3, 71.9, 60.2, 57.0, 56.0, 43.3, 39.9, 27.8, 27.7, 22.2. HRMS (+ESI-TOF) *m*/*z*: [M + H]^+^ Calcd for C_21_H_24_N_3_O_2_ 350.1863; found 350.1857.

5-Chloro-6′-methyl-3-(4-(methylsulfonyl)phenyl)-[2,3′-bipyridine]-2′-carbonitrile (**20**). Following GPD, but with the modification that the reaction was conducted at 80 °C, the title compound was obtained. Using 5-chloro-6′-methyl-3-(4-(methylsulfonyl)phenyl)-[2,3′-bipyridine] 1′-oxide (100 mg, 0.28 mmol), the title compound was obtained (43 mg, 40% yield) as a yellow oil. The spectroscopic data are consistent with previously reported [22]. TLC: *R_f_* = 0.29 (PE:EA = 1:1). ^1^H NMR (600 MHz, CDCl_3_) *δ* 8.75 (d, *J* = 2.4 Hz, 1H), 7.88 (d, *J* = 8.4 Hz, 2H), 7.83 (d, *J* = 1.8 Hz, 1H), 7.55 (d, *J* = 8.4 Hz, 1H), 7.36 (d, *J* = 8.4 Hz, 2H), 7.30 (d, *J* = 8.4 Hz, 1H), 3.06 (s, 3H), 2.60 (s, 3H).

## 4. Conclusions

In summary, we have developed an eco-friendly method for the *ortho*-cyanation of *N*-heterocycles. This approach achieves highly regioselective *ortho*-cyanation of heteroaromatic *N*-oxides efficiently through reaction with TMSCN under basic conditions. Notably, this reaction proceeds under mild conditions without transition metals or additional activating agents. The methodology demonstrates broad applicability, proving effective not only for simple quinoline, isoquinoline, and pyridine derivatives, but also for a diverse range of other nitrogen-containing heteroarenes, including pyrimidine, quinoxaline, naphthyridine, and related heterocyclic compounds. The synthetic utility of this method was further substantiated through gram-scale reactions and the successful late-stage functionalization of various biologically active molecules. We believe this methodology holds significant potential for the widespread synthesis and structural modification of pharmaceutically relevant aza-heterocyclic compounds.

## 5. Patents

The authors have a patent application related to the research disclosed in this manuscript (Patent Application No.: [202511279538.5]).

## Figures and Tables

**Table 1:** Optimization of cyanation reaction for quinoline *N*-oxide *.

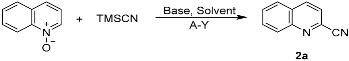				
Entry	Solvent (M)	A-Y (eq)	Base (eq)	Yield (%)
1 ^a,b^	DCE (0.25)	Ms_2_O (2)	TMG (4)	86
2 ^a^	DCE (0.25)	Ms_2_O (2)	TMG (4)	80
3	DCE (0.25)	Ts_2_O (2)	TMG (4)	62
4	DCE (0.25)	TsCl (2)	TMG (4)	70
5	DCE (0.25)	TsOH (2)	TMG (4)	<20
6 ^c^	DCE (0.25)	Ms_2_O (2)	N/A	86
7	DCE (0.25)	N/A	N/A	0
8	DCE (0.25)	N/A	TMG (4)	75
9	DCE (0.25)	N/A	DIEA (4)	96
10	DCE (0.25)	N/A	DMAP (4)	88
11	DCE (0.25)	N/A	DABCO (4)	66
12	DCE (0.25)	N/A	K_2_CO_3_ (4)	59
13	EA (0.25)	N/A	DIEA (4)	77
14	DCM (0.25)	N/A	DIEA (4)	94
15	THF (0.25)	N/A	DIEA (4)	89
16	DMF (0.25)	N/A	DIEA (4)	96
17	EtOH (0.25)	N/A	DIEA (4)	0
18	DCM (0.25)	N/A	DIEA (2)	92
19	DCM (0.5)	N/A	DIEA (2)	87
20	DCM (1)	N/A	DIEA (2)	91
21	DCM (2)	N/A	DIEA (2)	91
22	EA:DCM = 20:1 (2)	N/A	DIEA (2)	85
23	EA:DCM = 10:1 (2)	N/A	DIEA (2)	84
24	EA:DCM = 5:1 (2)	N/A	DIEA (2)	79
25 ^d^	EA (1)	N/A	DIEA (2)	93

* Unless otherwise noted, all reactions were conducted with quinoline *N*-oxide (100 mg, 1.0 equiv), TMSCN (3.0 equiv), base, and A-Y (activating agent) in dry solvent (0.25 M) at rt. ^a^ After reacting for 12 h, extra TMSCN (3.0 equiv) and Ms_2_O (2.0 equiv) were added to the reaction mixture. ^b^ lipoic acid (0.3 equiv) was utilized. ^c^ After reacting for 12 h, extra Ms_2_O (2.0 equiv) was added to the reaction mixture. ^d^ Reacting for 18 h.

**Table 2:** Green metrics for synthesis of compounds **2a**, **2d** and **2p**.

Cpd	Process ^a^	Yield (%)	AE (%)	AEf (%)	CE (%)	EcoScale ^b^
**2a**	A	93	63	59	45	77.5
	B	73	27	20	13	60.5
**2d**	A	94	65	61	47	78
	B	82	29	24	17	65
**2p**	A	92	72	66	58	77
	B	70	36	25	19	59

^a^ Process A is our developed method. Process B is the method reported in ref. [38], shown in Scheme 1D. ^b^ >75, excellent; >50, acceptable; <50, inadequate.

## Data Availability

The data underlying this study are available in the published article and its Supplementary Materials.

## Supplementary Materials

The following supporting information can be downloaded at https://www.mdpi.com/article/10.3390/molecules31020276/s1. Table S1: Optimization of cyanation reaction for pyridine *N*-oxides; NMR and Mass spectra.

## Abbreviations

The following abbreviations are used in this manuscript:

TMSCN	Trimethylsilyl cyanide
DCE	1,2-Dichloroethane
Ms_2_O	Methanesulfonic anhydride
Ts_2_O	Trifluoromethanesulfonic anhydride
TsCl	Tosyl chloride
TsOH	*p*-Toluenesulfonic acid
TMG	Tetramethylguanidine
DIPEA	*N*,*N*-Diisopropylethylamine
DCM	Dichloromethane
DMAP	4-Dimethylaminopyridine
DABCO	1,4-Diazabicyclo[2.2.2]octane
DMF	*N*,*N*-Dimethylformamide
EA	Ethyl acetate
THF	Tetrahydrofuran
