# Supplementary material for: Formal Synthesis of Ortho-Cyanated N-Heterocycles via Direct, Metal-Free Cyanation of N-Oxides Under Benign Conditions
Source: Molecules. 2026 Jan 13;31(2):276. doi: 10.3390/molecules31020276 (PMC12844355; doi:10.3390/molecules31020276)
Supplement: Supplementary file 1 [file molecules-31-00276-s001.zip › molecules-4062662-supplementary.pdf]

Supplementary Materials

Formal Synthesis of *ortho*-Cyanated *N*-Heterocycles via Direct, Metal-Free Cyanation of *N*-Oxides under Benign Conditions

Hua Xiao, Reziyamu Wufuer and Dong Wang*

chem_wd@hotmail.com

Table of Contents

1. Screening of the Reaction Conditions.. 2

2. Gram scale synthesis 5

3. Green Chemistry Metrics Analysis 6

4. References... 12

5. NMR Spectra Data 13

6. HRMS Spectra Data.. 58

1. Screening of the Reaction Conditions

Table S1 Optimization of cyanation reaction for pyridine *N*-oxides*

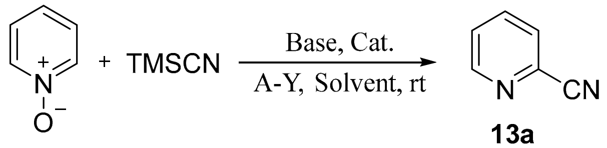

entry	NuH (eq)	Solvent	T (°C)	Cat. (eq)	A-Y (eq)	Base (eq)	Yield (%)
1[a,b]	TMSCN (3+3)	DCE	r.t.	lipoic acid (0.3)	Ms_2O (2+2)	TMG (4)	42
2[a]	TMSCN (3+3)	DCE	r.t.	lipoic acid (0.3)	N/A	TMG (4)	44
**Catalyst equivalent screening**							
3[a]	TMSCN (3+3)	DCE	r.t.	lipoic acid (1.0)	N/A	TMG (4)	25
**Solvent screening**							
4[a]	TMSCN (3+3)	EA	r.t.	lipoic acid (0.3)	N/A	TMG (4)	33
5[a]	TMSCN (3+3)	THF	r.t.	lipoic acid (0.3)	N/A	TMG (4)	35
6[a]	TMSCN (3+3)	DMF	r.t.	lipoic acid (0.3)	N/A	TMG (4)	20
**Without catalyst or base, or activating agent conditions**							
7[a,b]	TMSCN (3+3)	DCE	r.t.	N/A	Ms_2O (2+2)	TMG (4)	49
8[a,b]	TMSCN (3+3)	DCE	r.t.	lipoic acid (0.3)	Ms_2O (2+2)	N/A	56
9[a]	TMSCN (3+3)	DCE	r.t.	N/A	N/A	TMG (4)	66
**Base screening**							

10[a]	TMSCN (3+3)	DCE	r.t.	N/A	N/A	DMAP (4)	44
11[a]	TMSCN (3+3)	DCE	r.t.	N/A	N/A	DABCO (4)	14
12[a]	TMSCN (3+3)	DCE	r.t.	N/A	N/A	K_2CO_3 (4)	0
**Temperature screening**							
13[a]	TMSCN (3+3)	DCM	r.t.	N/A	N/A	TMG (4)	31
14[a]	TMSCN (3+3)	DCE	80	N/A	N/A	TMG (4)	65
**Base equivalent screening**							
**15[a]**	**TMSCN (3+3)**	**DCE**	**r.t.**	**N/A**	**N/A**	**TMG (2)**	**82**
16[a]	TMSCN (3+3)	DCE	r.t.	N/A	N/A	TMG (6)	35
**Solvent screening**							
17[a]	TMSCN (3+3)	CH_3CN	r.t.	N/A	N/A	TMG (2)	61
18[a]	TMSCN (3+3)	EtOH	r.t.	N/A	N/A	TMG (2)	0
19[a]	TMSCN (3+3)	DCM	r.t.	N/A	N/A	TMG (2)	36
**20[a]**	**TMSCN (3+3)**	**EA**	**r.t.**	**N/A**	**N/A**	**TMG (2)**	**72**
21[a]	TMSCN (3+3)	DMF	r.t.	N/A	N/A	TMG (2)	21
22[a]	TMSCN (3+3)	Anisole	r.t.	N/A	N/A	TMG (2)	0
23[a]	TMSCN (3+3)	Sulfolane	r.t.	N/A	N/A	TMG (2)	62
24[a]	TMSCN (3+3)	DMC	r.t.	N/A	N/A	TMG (2)	64
25[a]	TMSCN (3+3)	Anisole	80	N/A	N/A	TMG (2)	30
26[a]	TMSCN (3+3)	CPME	80	N/A	N/A	TMG (2)	39
27[a]	TMSCN (3+3)	Acetone	80	N/A	N/A	TMG (2)	0
28[a]	TMSCN (3+3)	EA:DCE=20:1	r.t.	N/A	N/A	TMG (2)	70
29[a]	TMSCN (3+3)	EA:DCE=10:1	r.t.	N/A	N/A	TMG (2)	84
30[a]	TMSCN (3+3)	EA:DCE=5:1	r.t.	N/A	N/A	TMG (2)	21
31[c]	TMSCN (6)	EA:DCE=10:1	r.t.	N/A	N/A	TMG (2)	41
**Temperature screening**							
32[a]	TMSCN (3+3)	EA	60	N/A	N/A	TMG (2)	37
33[a]	TMSCN (3+3)	EA:DCE=10:1	60	N/A	N/A	TMG (2)	27
**Base screening**							

34[a]	TMSCN (3+3)	DCE	r.t.	N/A	N/A	Sulfaguanidine (2)	0
35[a]	TMSCN (3+3)	DCE	r.t.	N/A	N/A	Metformin (2)	0
36	TMSCN (3)	DCM (2 M)	r.t.	N/A	N/A	DIEA (2)	0

* Unless otherwise noted, all reactions were conducted with pyridine *N*-oxide (100 mg, 1 equiv), TMSCN (3.0 equiv), base, and A-Y (activating agent) in dry solvent (0.25 M) at rt. [a] After reacting for 12 h, extra TMSCN (3.0 equiv) was added to the reaction mixture. [b] At the beginning of the reaction, 6.0 equivalents of TMSCN were added in one portion. After reacting for 12 h, extra Ms_2O (2.0 equiv) was added to the reaction mixture. [c] At the beginning of the reaction, 6 equivalents of TMSCN were added in one portion.

Although the reaction conditions in entry 29 afforded the highest yield, they proved ineffective for other substrates, such as methyl isonicotinate *N*-oxide (0% yield), 4-methylpyridine *N*-oxide (34% yield), and 4-(tert-butyl)pyridine *N*-oxide (23% yield).

2. Gram scale synthesis

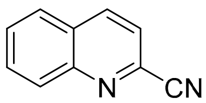

2a

Gram scale synthesis:

To a solution of quinoline *N*-oxide (1 g, 6.89 mmol) in dry EA (7.0 mL) is added TMSCN (2.05 g, 20.67 mmol) and DIEA (1.79 g, 13.78 mmol). The reaction mixture is stirred for 12 hours and the progress is monitored by TLC. Upon completion, the reaction is quenched with a saturated sodium bicarbonate solution. The mixture is extracted with EA (3 × 15 mL). The combined organic layers are dried over Na_2SO_4, filtered, and concentrated under reduced pressure to give the crude product. Purification by flash column chromatography (eluent: PE/EA= 20:1) affords the desired product (971 mg, 91%).

$$Yield = \frac{Actual\ yield}{Theoretical\ yield} \times 100\% = \frac{971}{1062} \times 100\% = 91\%$$

$$Yield\ (\mathbf{A}) = \frac{Actual\ yield}{Theoretical\ yield} \times 100\% = \frac{66}{103.9} \times 100\% = 64\%$$

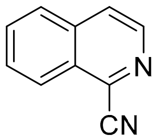

3a

Gram scale synthesis:

To a solution of isoquinoline *N*-oxide (1 g, 6.89 mmol) in dry EA (7.0 mL) is added TMSCN (2.05 g, 20.67 mmol) and DIEA (1.79 g, 13.78 mmol). The reaction mixture is stirred for 16 hours and the progress is monitored by TLC. Upon completion, the reaction is quenched with a saturated sodium bicarbonate solution. The mixture is extracted with EA (3 × 15 mL). The combined organic layers are dried over Na_2SO_4, filtered, and concentrated under reduced pressure to give the crude product. Purification by flash column chromatography (eluent: PE/EA = 20:1) furnishes the desired product (884 mg, 83%).

$$Yield = \frac{Actual\ yield}{Theoretical\ yield} \times 100\% = \frac{884}{1062} \times 100\% = 83\%$$

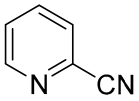

13a

Gram scale synthesis:

To a solution of pyridine N-oxide (1 g, 10.5 mmol) in dry EA (42.0 mL) is added TMSCN (3.13 g, 31.5 mmol) and TMG (2.42 g, 21.0 mmol). Following stirring at room temperature for 12 hours, additional TMSCN (2.42 g, 21.0 mmol) is added. After a further 36 hours (reaction completion by TLC), the mixture is quenched with saturated $NaHCO_3$ (aq). The mixture is extracted with EA (3 × 15 mL), and the combined organic phases are dried (Na_2SO_4), filtered, and concentrated in vacuo. The crude product is purified by flash chromatography (PE/EA = 5:1) to give the target compound. furnishes the desired pure product (632 mg, 58%).

$$Yield = \frac{Actual\ yield}{Theoretical\ yield} \times 100\% = \frac{632}{1093} \times 100\% = 58\%$$

3. Green Chemistry Metrics Analysis

The following formula were used for calculating Atom Economy (AE)

$$AE= \frac{\text{Molecular weight of product}}{\text{Total molecular weight of reactants}} \times 100\%$$

$$AEf = AE \times yield\%$$

$$CE= \frac{\text{Amount of carbon in the product}}{\text{Total carbon present in reactants}} \times 100\%$$

3.1 Compound 2a: Process A (our developed method)

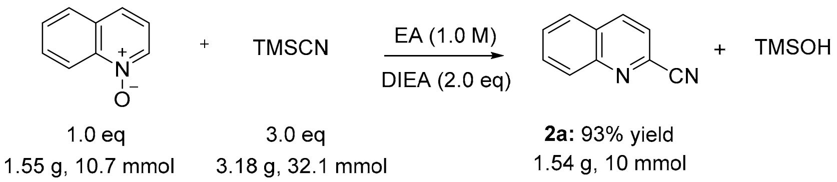

AE (**2a**)= $\frac{154.17}{145.16+99.21} \times 100= 63$

AEf (**2a**)= 63 × 93%= 59%

CE (**2a**)= $\frac{10\times10}{10.7\times9+32.1\times4} \times 100= 45$

Parameters	Penalty
1. Yield: 93%	3.5
2. Price of reaction components (to obtain 10 mmol of end product)	
quinoline *N*-oxide (1.55 g, 10.7 mmol)	0
TMSCN (3.18 g, 32.1 mmol)	0
DIEA (2.77 g, 21.4 mmol)	0
3. Safety	
EtOAc (F)	5
4. Technical setup	
Common setup	0
5. Temperature/time	
Room temperature, < 24 h	1
6. Workup and purification	
Extraction with EA	3
Classical chromatography	10
Penalty points total:	22.5

EcoScale =100-22.5=77.5

Compound 2a: Process B[1]

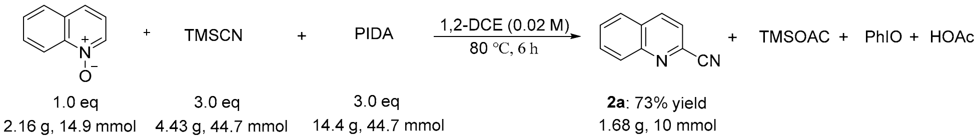

AE (**2a**)= $\frac{154.17}{145.16+99.21+322.1}$ ×100= 27

AEf (**2a**)= 27 × 73%= 20%

CE (**2a**)= $\frac{10\times10}{14.9\times9+44.7\times4+44.7\times10}$ ×100= 13

Parameters	Penalty
1. Yield: 73%	13.5
2. Price of reaction components (to obtain 10 mmol of end product)	
quinoline *N*-oxide (2.16 g, 14.9 mmol)	0
TMSCN (4.43 g, 44.7 mmol)	0
PIDA (14.4 g, 44.7 mmol)	0
3. Safety	
1,2-DCE (T, F)	10
4. Technical setup	
Common setup	0
5. Temperature/time	
80 °C, < 6 h	3
6. Workup and purification	
Extraction with DCM	3
Classical chromatography	10
Penalty points total:	39.5

EcoScale=100-39.5=60.5

3.2 Compound 2d: Process A (our developed method)

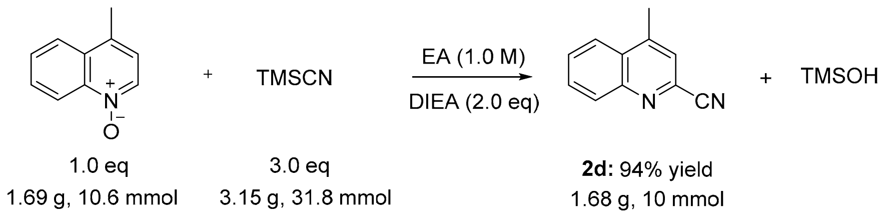

AE (**2d**)= $\frac{168.2}{159.19+99.21}$ ×100= 65

AEf (**2d**)= 65 × 94%= 61%

CE (**2d**)= $\frac{10\times11}{10.6\times10+31.8\times4}$ ×100= 47

Parameters	Penalty
1. Yield: 94%	3
2. Price of reaction components (to obtain 10 mmol of end product)	
4-methylquinoline *N*-oxide (1.69 g, 10.6 mmol)	0
TMSCN (3.15 g, 31.8 mmol)	0
DIEA (2.74 g, 21.2 mmol)	0
3. Safety	
EtOAc (F)	5
4. Technical setup	
Common setup	0
5. Temperature/time	
Room temperature, < 24 h	1
6. Workup and purification	
Extraction with EA	3
Classical chromatography	10
Penalty points total:	22

EcoScale =100-22=78

Compound 2d: Process B[1]

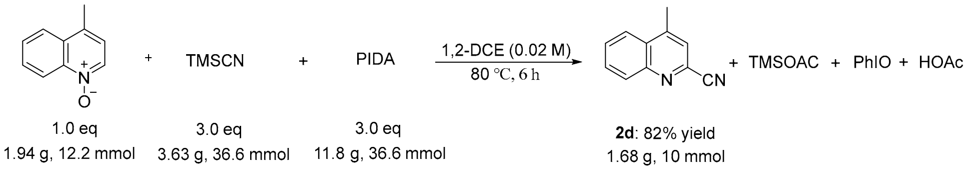

AE (**2d**)= $\frac{168.2}{159.19+99.21+322.1}$ ×100= 29

AEf (**2d**)= 29 × 82%= 24%

$$\text{CE } (\mathbf{2d}) = \frac{10\times11}{12.2\times10+36.6\times4+36.6\times10}\times100 = 17$$

Parameters	Penalty
1. Yield: 82%	9
2. Price of reaction components (to obtain 10 mmol of end product)	
4-methylquinoline *N*-oxide (1.94 g, 12.2 mmol)	0
TMSCN (3.63 g, 36.6 mmol)	0
PIDA (11.8 g, 36.6 mmol)	0
3. Safety	
1,2-DCE (T, F)	10
4. Technical setup	
Common setup	0
5. Temperature/time	
80 °C, < 6 h	3
6. Workup and purification	
Extraction with DCM	3
Classical chromatography	10
Penalty points total:	35

EcoScale=100-35=65

3.3 Compound 2p: Process A (our developed method)

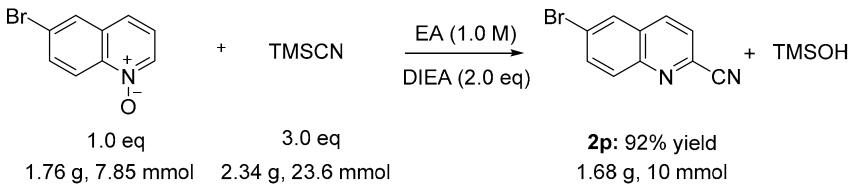

$$\text{AE } (\mathbf{2p}) = \frac{233.07}{224.06+99.21}\times100 = 72$$

AEf (**2p**)= 72 × 92%= 66%

CE (**2p**)= $\frac{10\times10}{7.85\times10+23.6\times4}$ ×100= 58

Parameters	Penalty
1. Yield: 92%	4
2. Price of reaction components (to obtain 10 mmol of end product)	
6-bromoquinoline *N*-oxide (1.76 g, 7.85 mmol)	0
TMSCN (2.34 g, 23.6 mmol)	0
DIEA (2.03 g, 15.7 mmol)	0
3. Safety	
EtOAc (F)	5
4. Technical setup	
Common setup	0
5. Temperature/time	
Room temperature, < 24 h	1
6. Workup and purification	
Extraction with EA	3
Classical chromatography	10
Penalty points total:	23

EcoScale =100-23=77

Compound 2p: Process B[1]

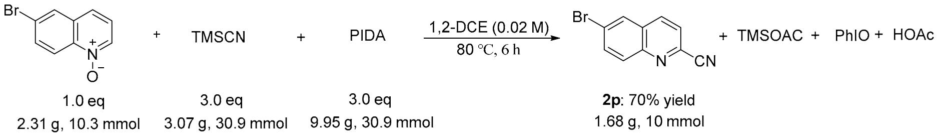

AE (**2p**)= $\frac{233.07}{224.06+99.21+322.1}$ ×100= 36

AEf (**2p**)= 36 × 70%= 25%

CE (**2p**)= $\frac{10\times10}{10.3\times10+30.9\times4+30.9\times10}$ ×100= 19

Parameters	Penalty
1. Yield: 70%	15
2. Price of reaction components (to obtain 10 mmol of end product)	
6-bromoquinoline *N*-oxide (2.31 g, 10.3 mmol)	0
TMSCN (3.07 g, 30.9 mmol)	0
PIDA (9.95 g, 30.9 mmol)	0
3. Safety	
1,2-DCE (T, F)	10
4. Technical setup	
Common setup	0
5. Temperature/time	
80 °C, < 6 h	3
6. Workup and purification	
Extraction with DCM	3
Classical chromatography	10
Penalty points total:	41

EcoScale=100-41=59

4. References

1. Xu, F.; Li, Y.; Huang, X.; Fang, X.; Li, Z.; Jiang, H.; Qiao, J.; Chu, W.; Sun, Z., Hypervalent Iodine(III)-Mediated Regioselective Cyanation of Quinoline N-Oxides with Trimethylsilyl Cyanide. *Advanced Synthesis & Catalysis* **2019,** *361* (3), 520-525.

5. NMR Spectra Data

Spectra data are shown from the next page.

^{1}H NMR (600 MHz, $CDCl_3$):

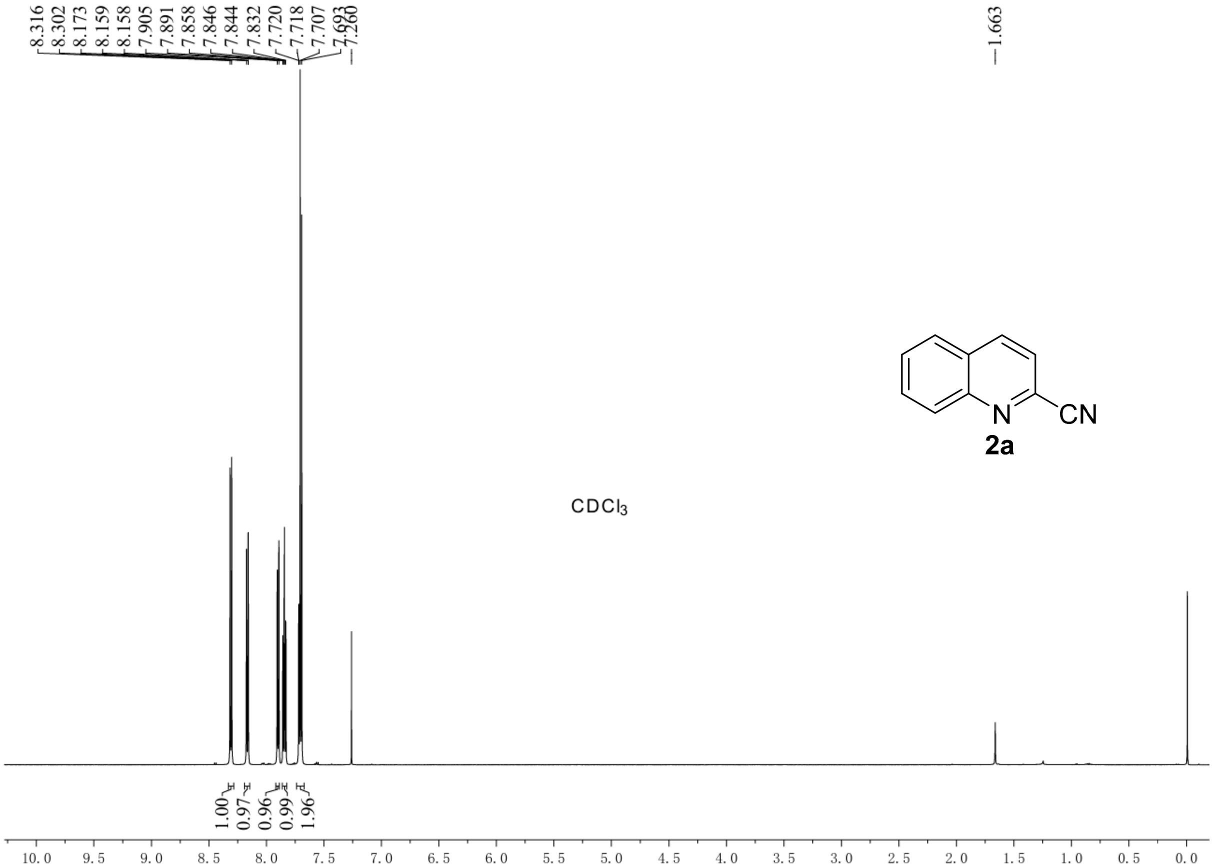

^{1}H NMR (600 MHz, $CDCl_3$):

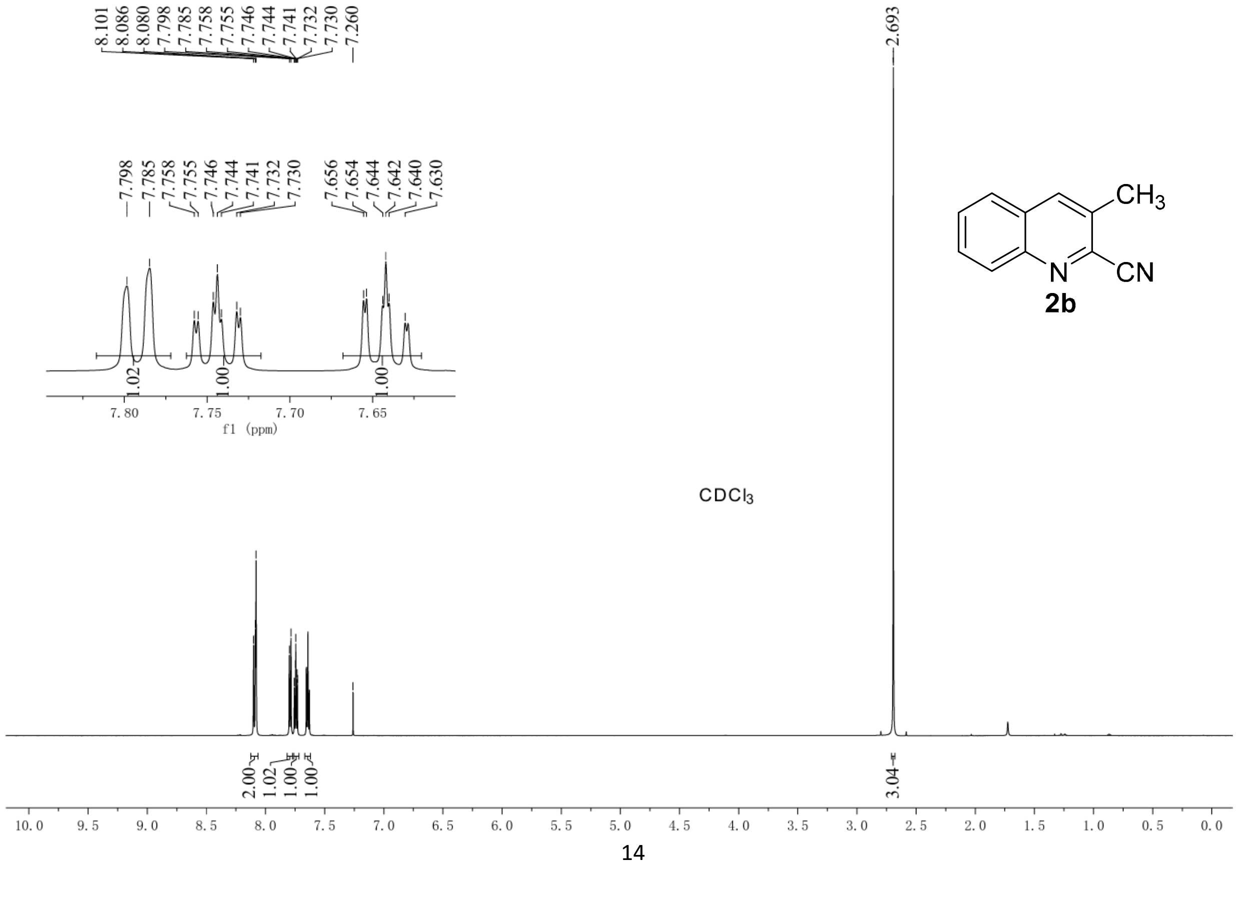

^{1}H NMR (600 MHz, CDCl$_3$):

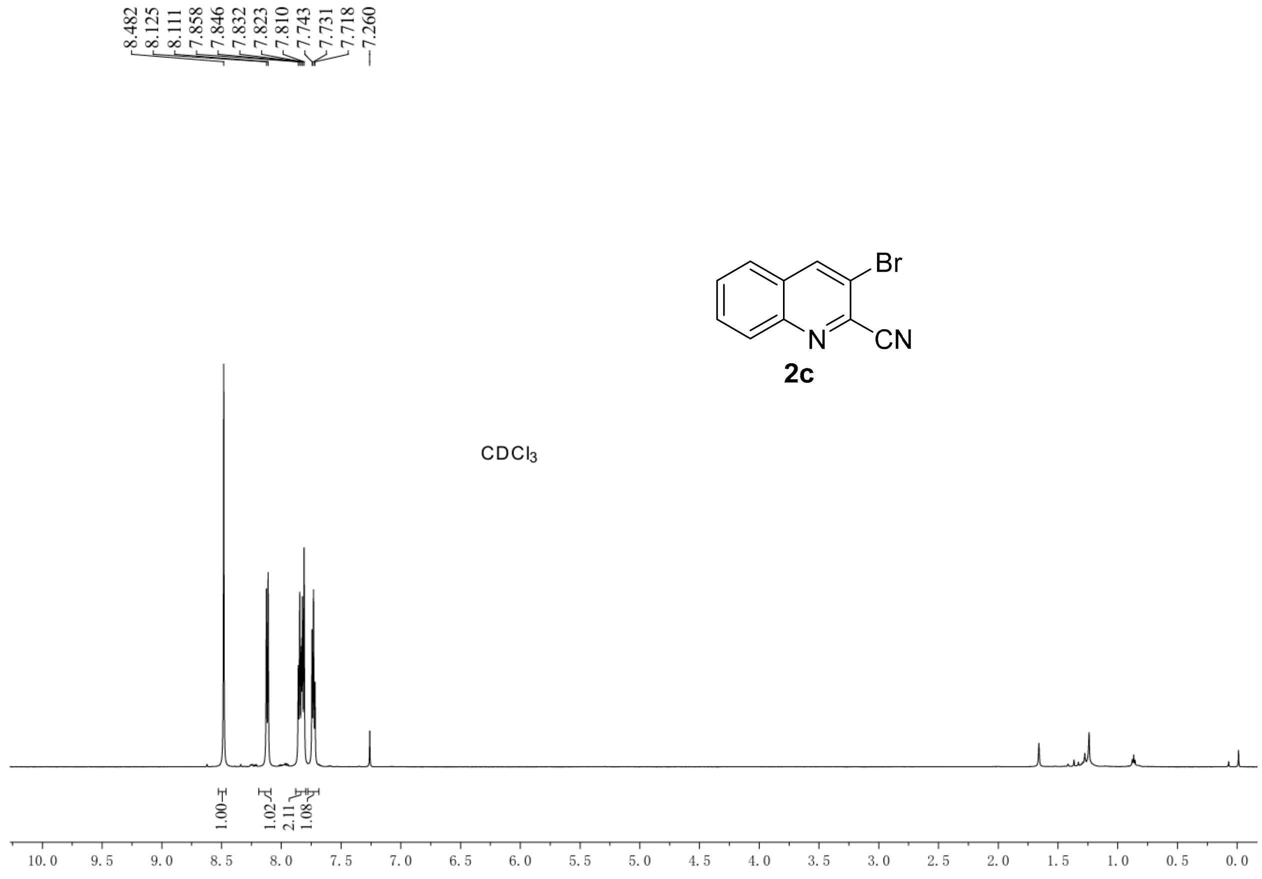

^{1}H NMR (600 MHz, CDCl$_3$):

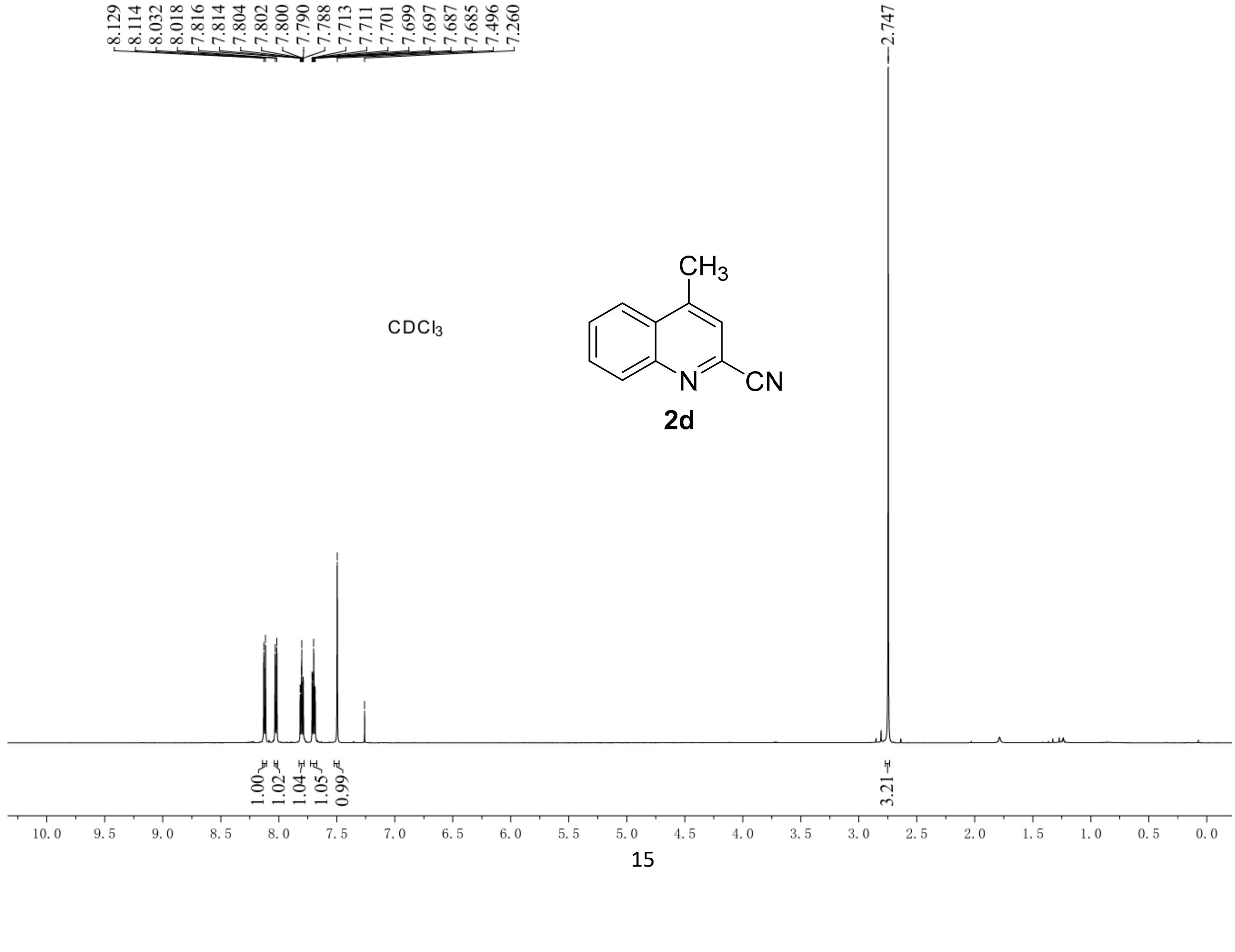

^{1}H NMR (600 MHz, $CDCl_3$):

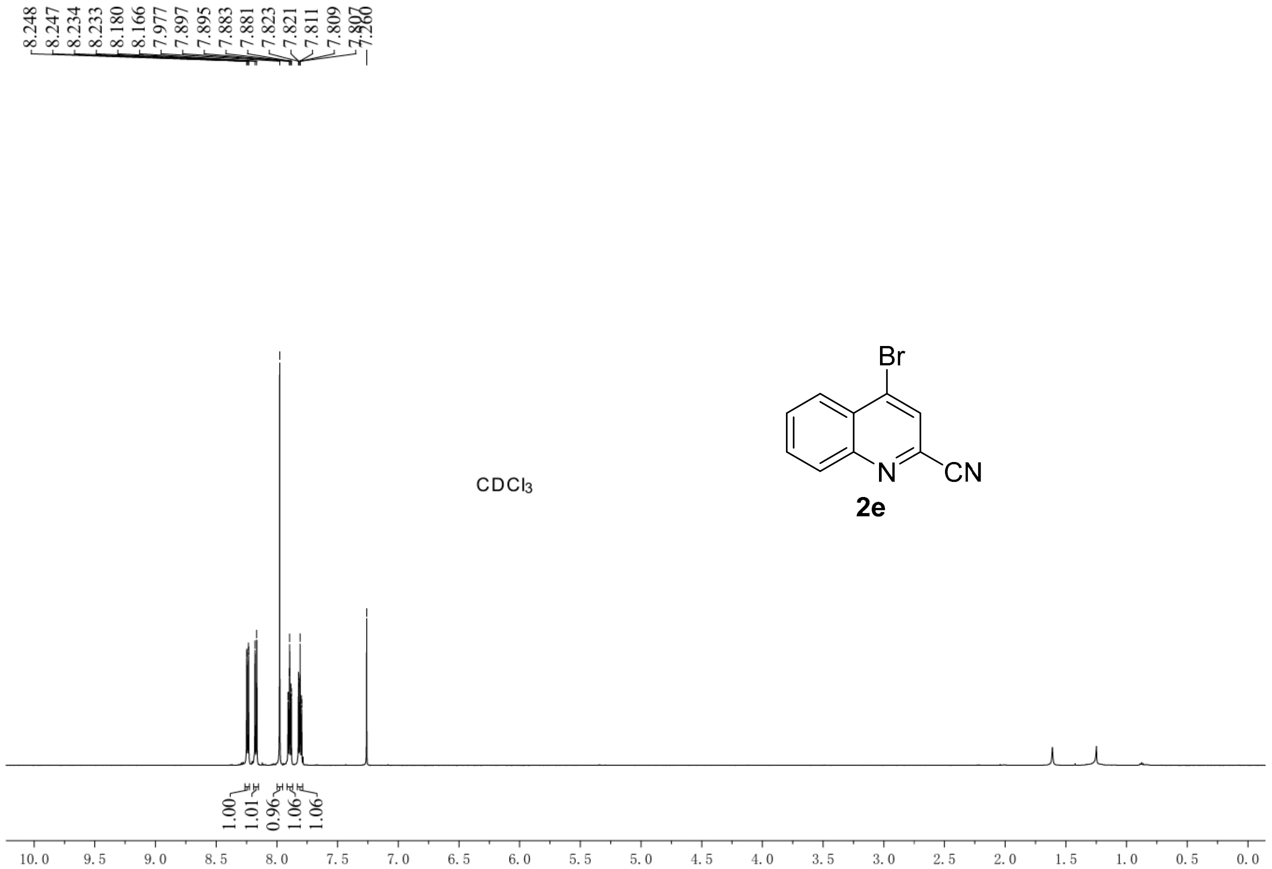

^{1}H NMR (600 MHz, $CDCl_3$):

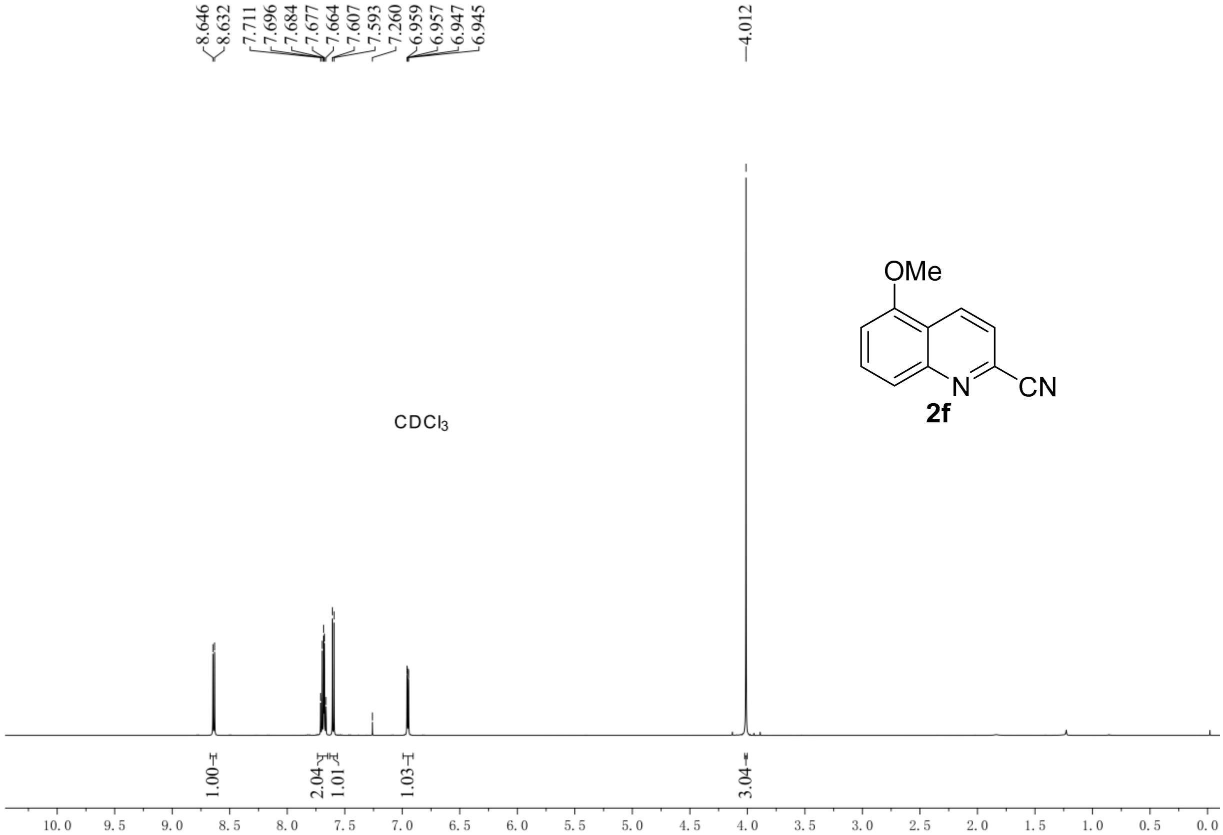

^{1}H NMR (600 MHz, $CDCl_3$):

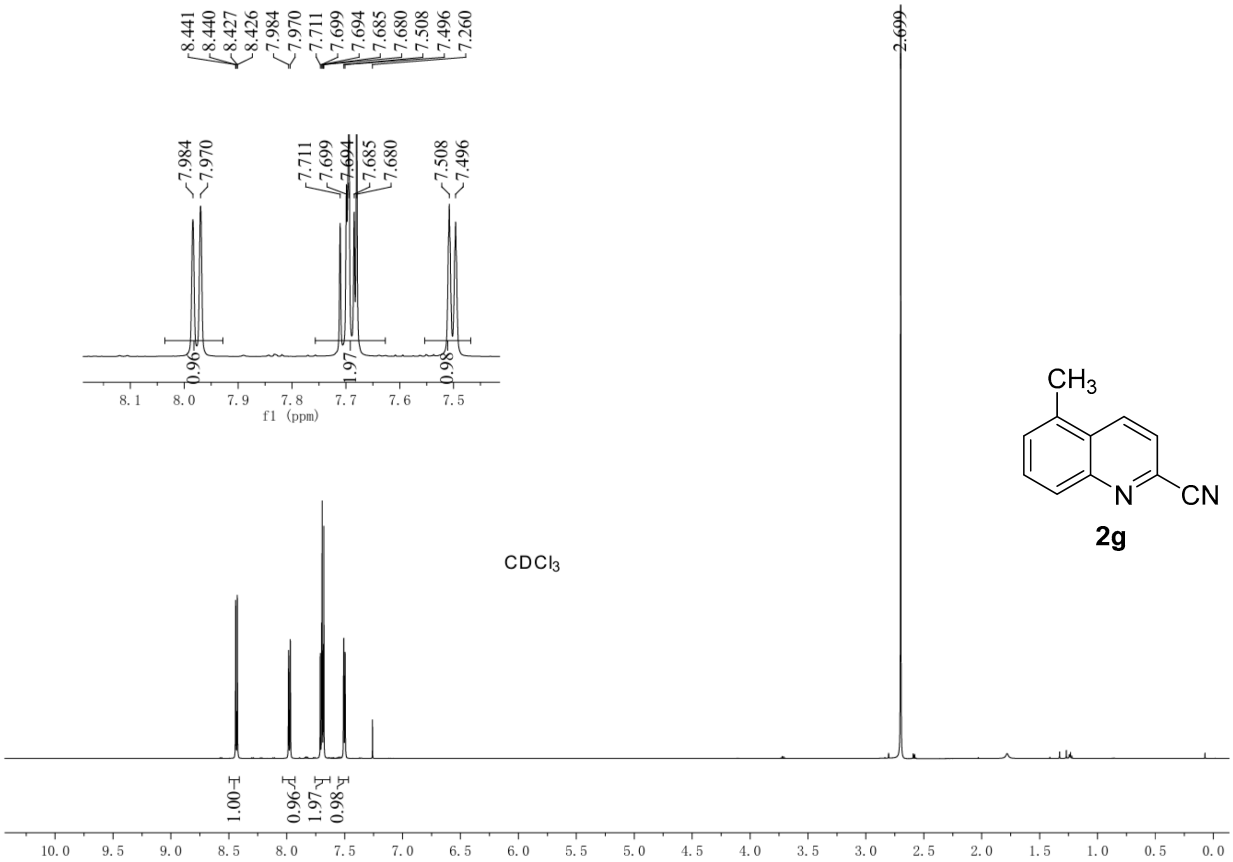

^{13}C NMR (150 MHz, $CDCl_3$):

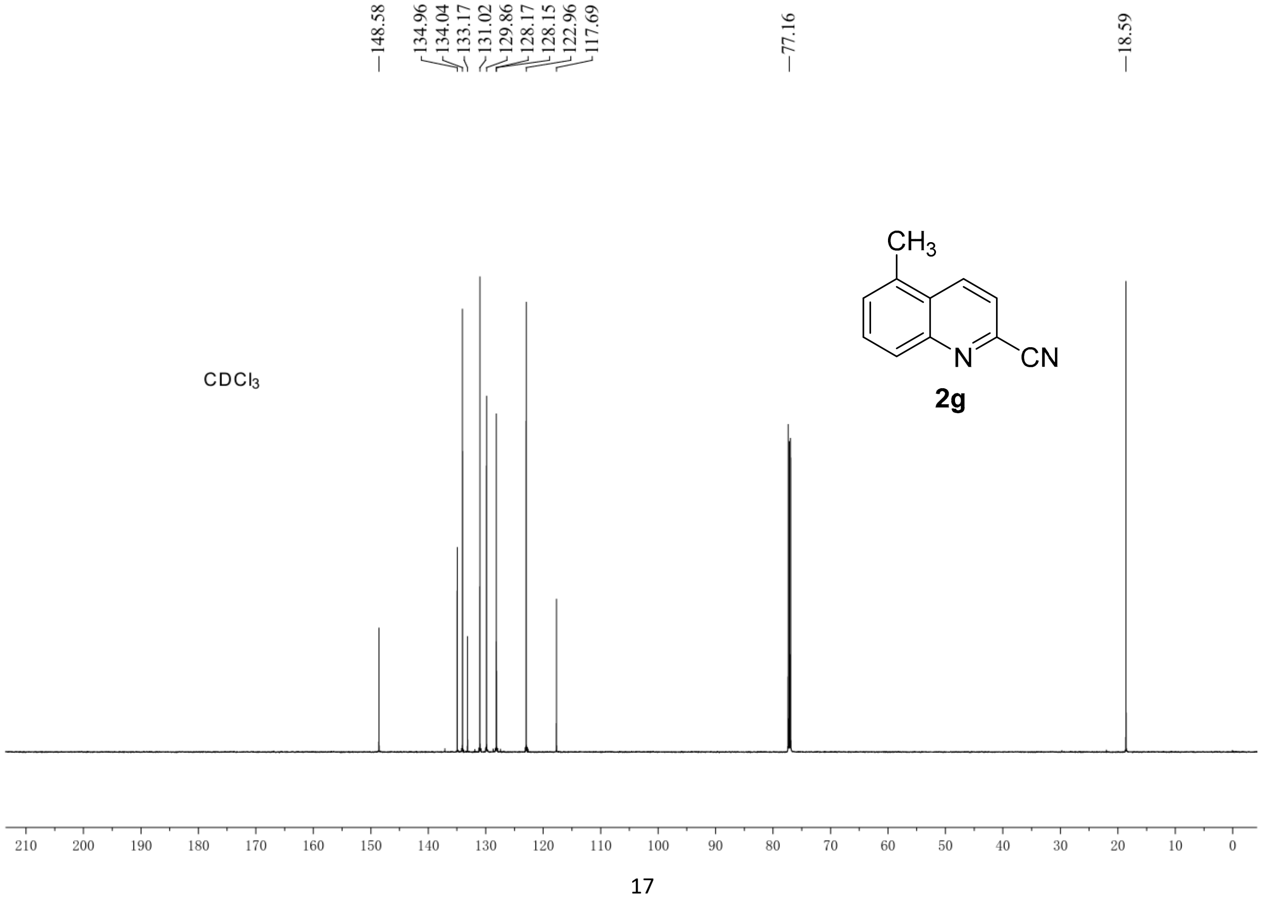

^{1}H NMR (600 MHz, $CDCl_3$):

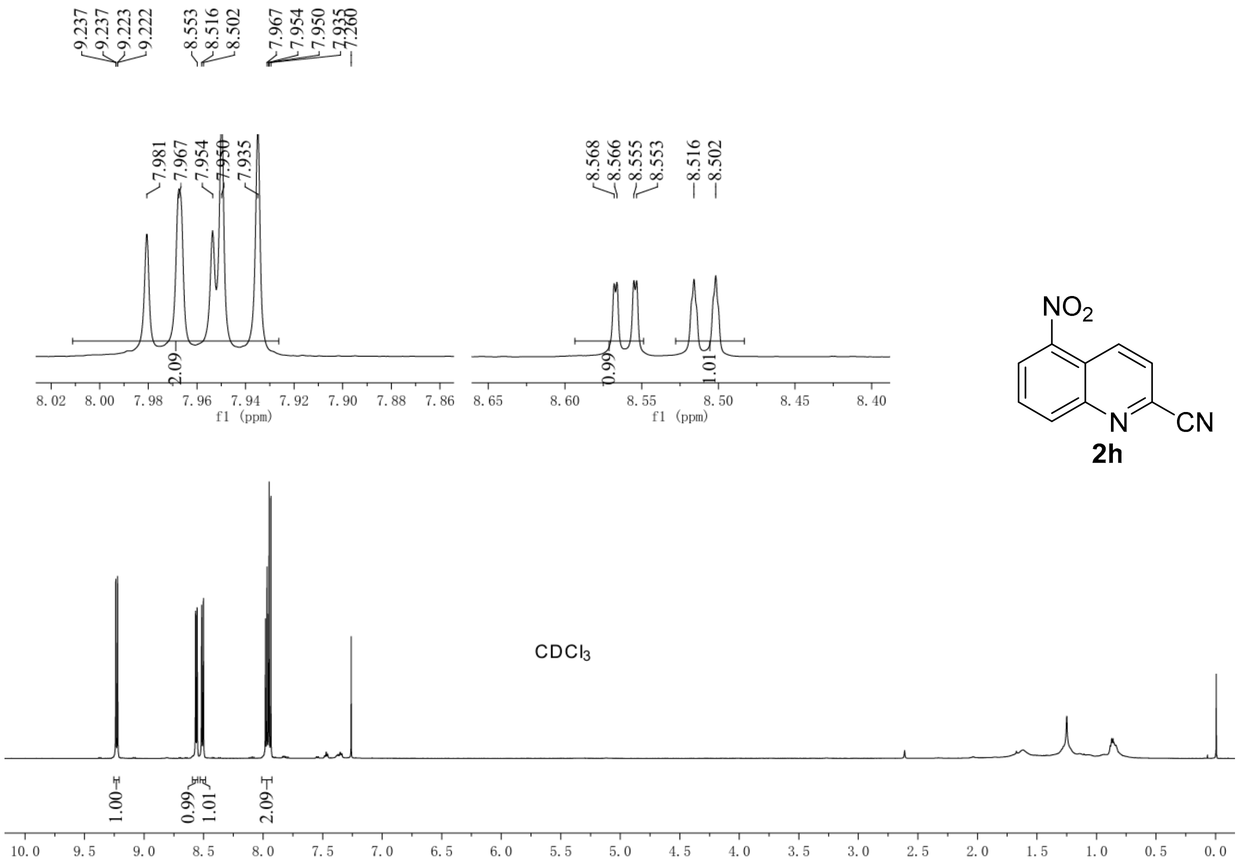

^{1}H NMR (600 MHz, $CDCl_3$):

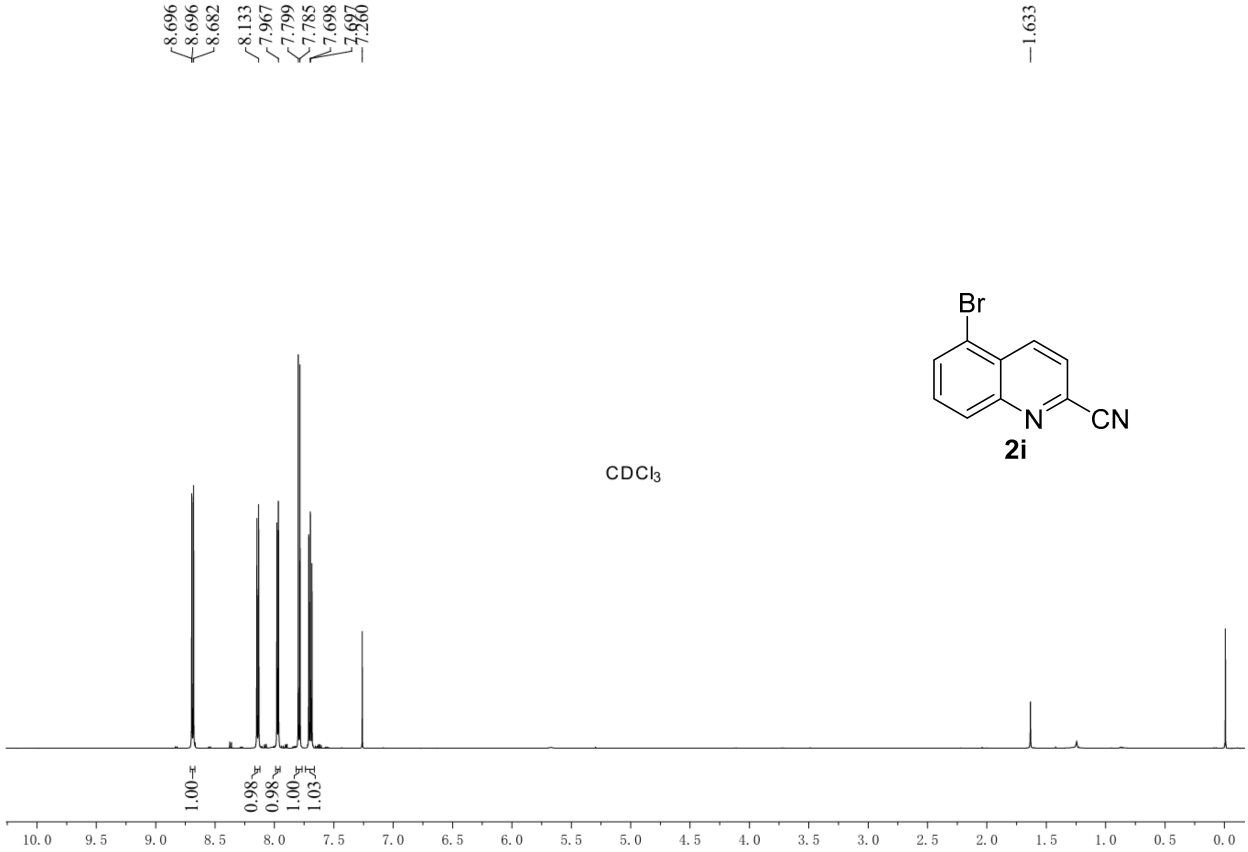

^{1}H NMR (600 MHz, $CDCl_3$):

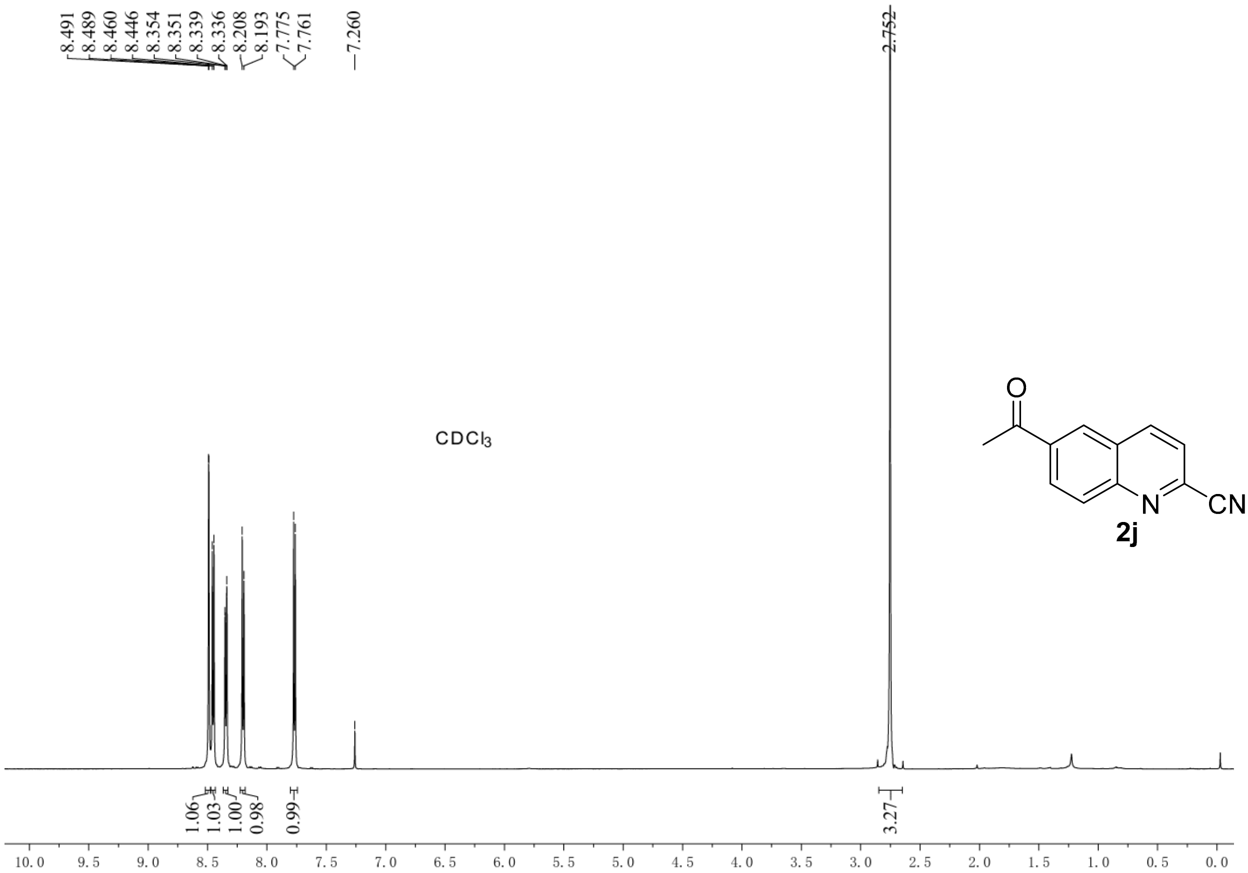

^{13}C NMR (150 MHz, $CDCl_3$):

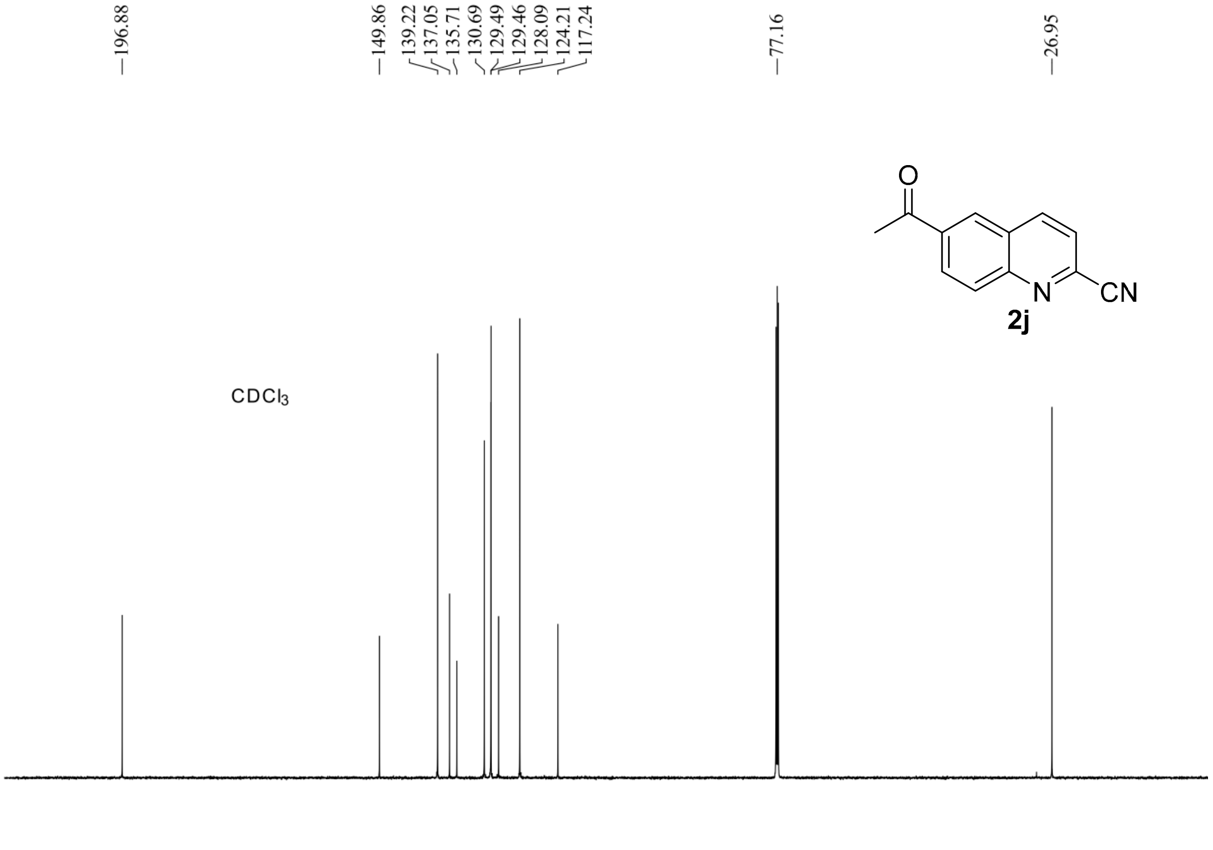

^{1}H NMR (600 MHz, $CDCl_3$):

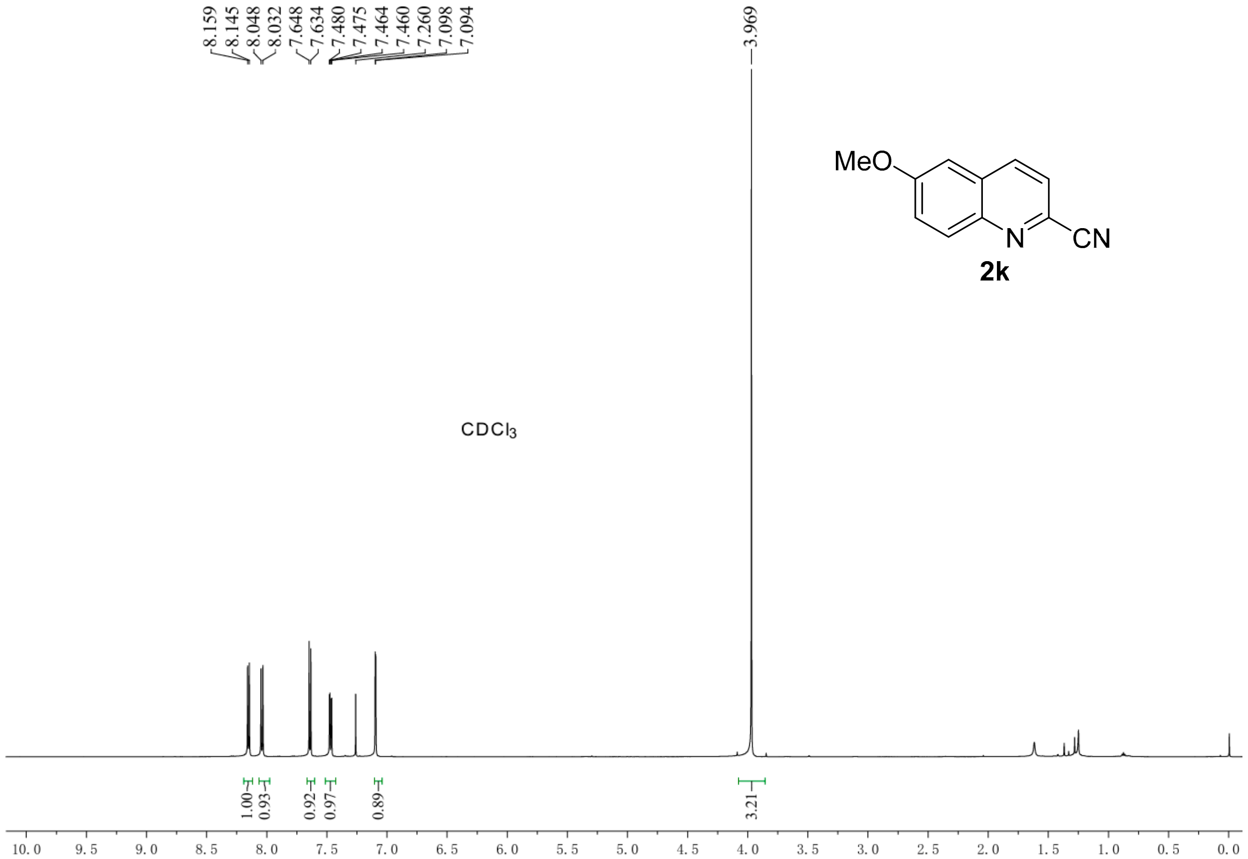

^{1}H NMR (600 MHz, $CDCl_3$):

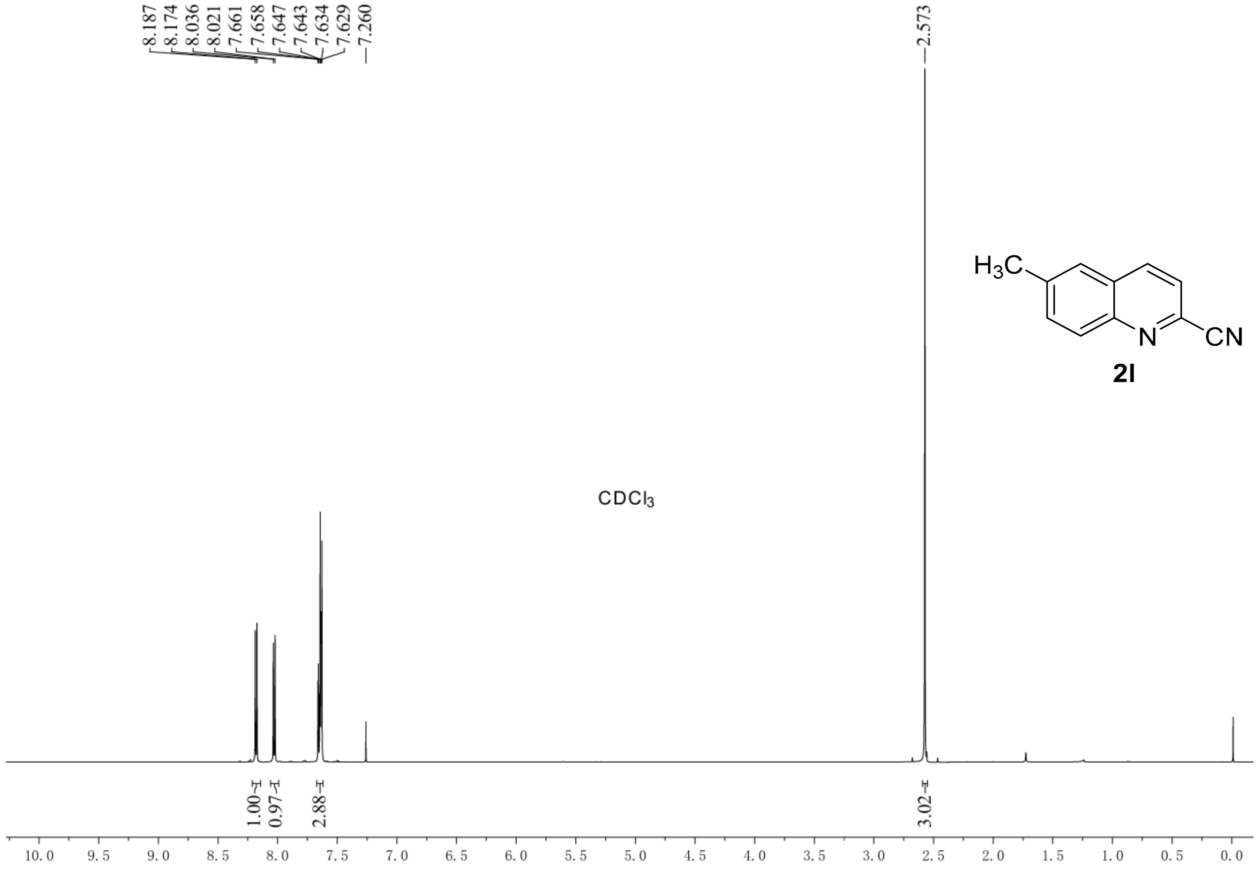

^{1}H NMR (600 MHz, $CDCl_3$):

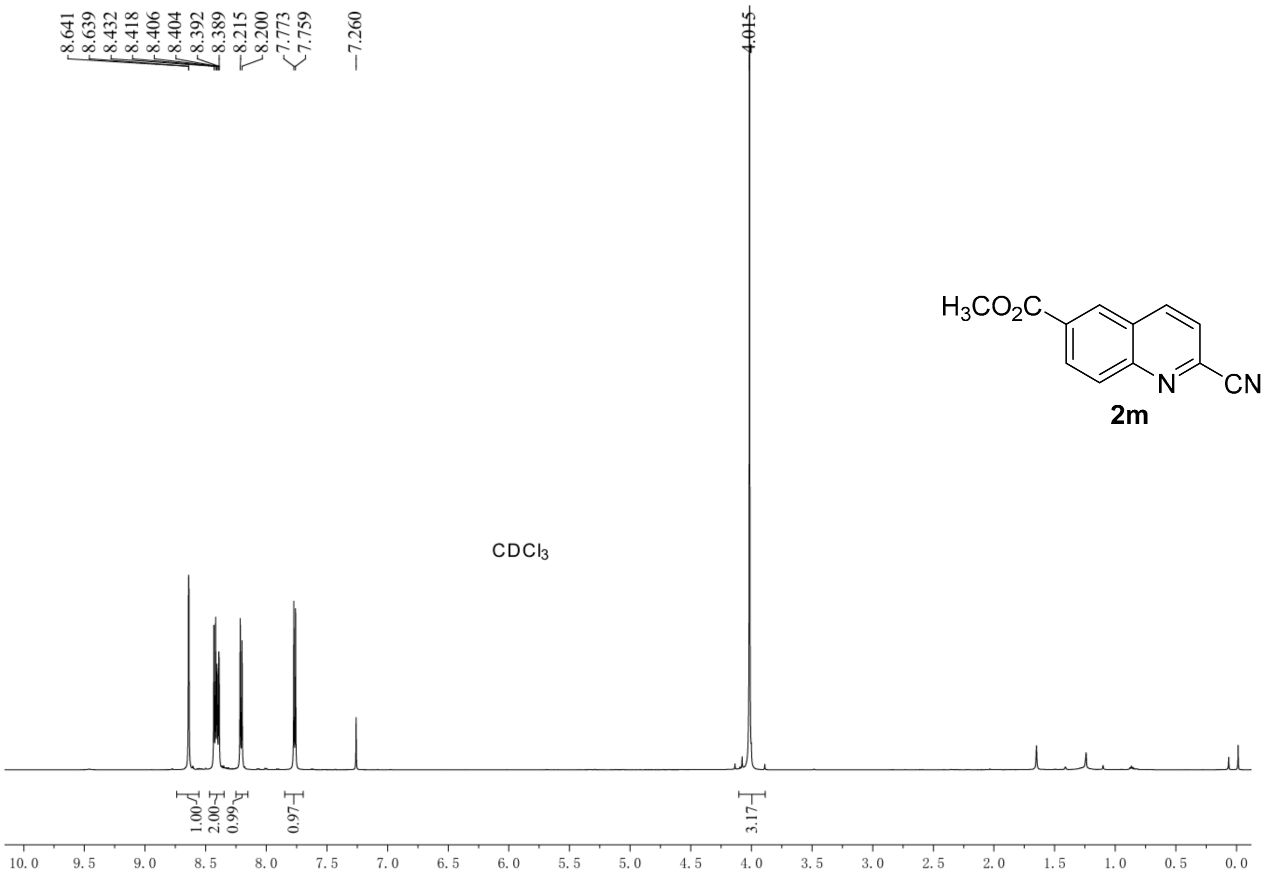

^{1}H NMR (600 MHz, $CDCl_3$):

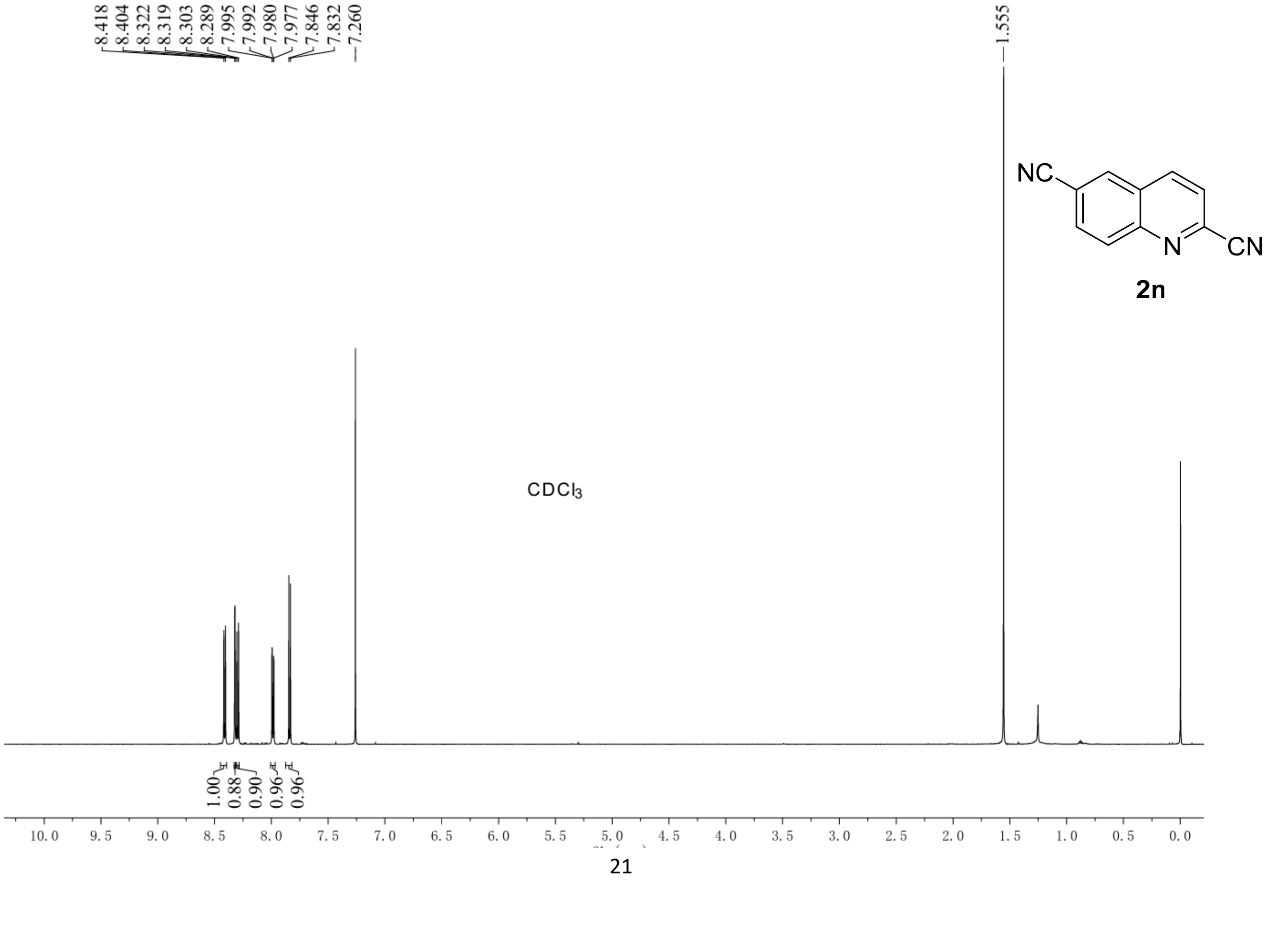

^{1}H NMR (600 MHz, $CDCl_3$):

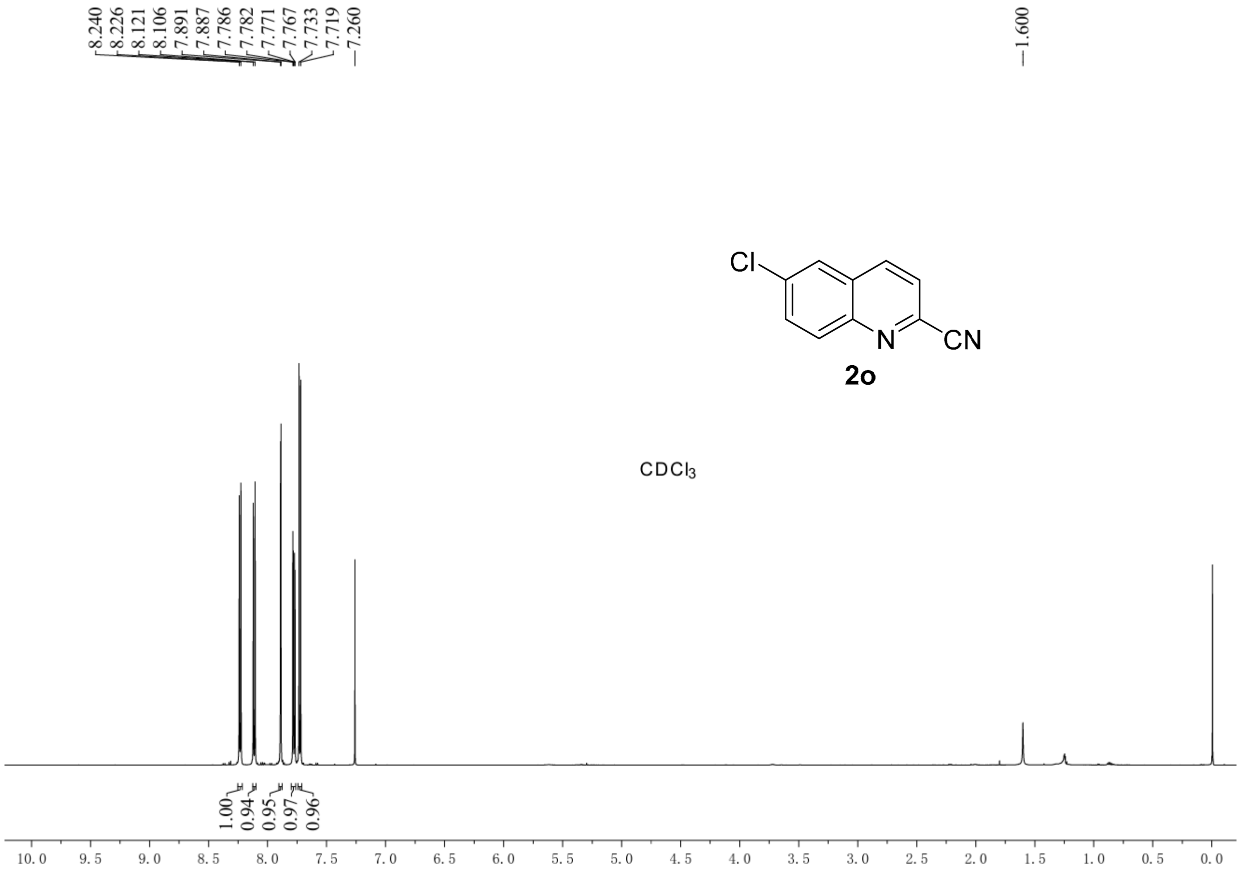

^{1}H NMR (600 MHz, $CDCl_3$):

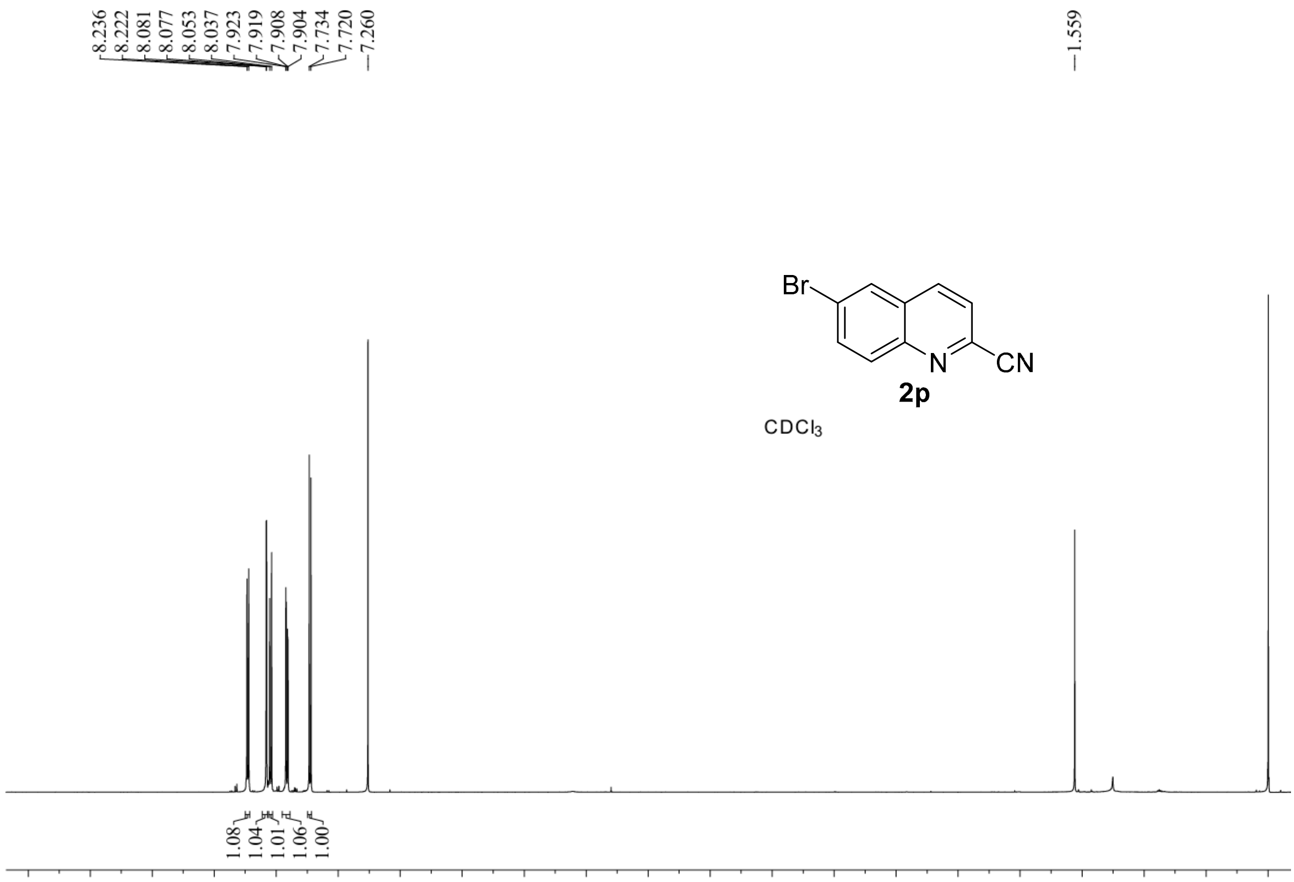

^{1}H NMR (600 MHz, CDCl$_3$):

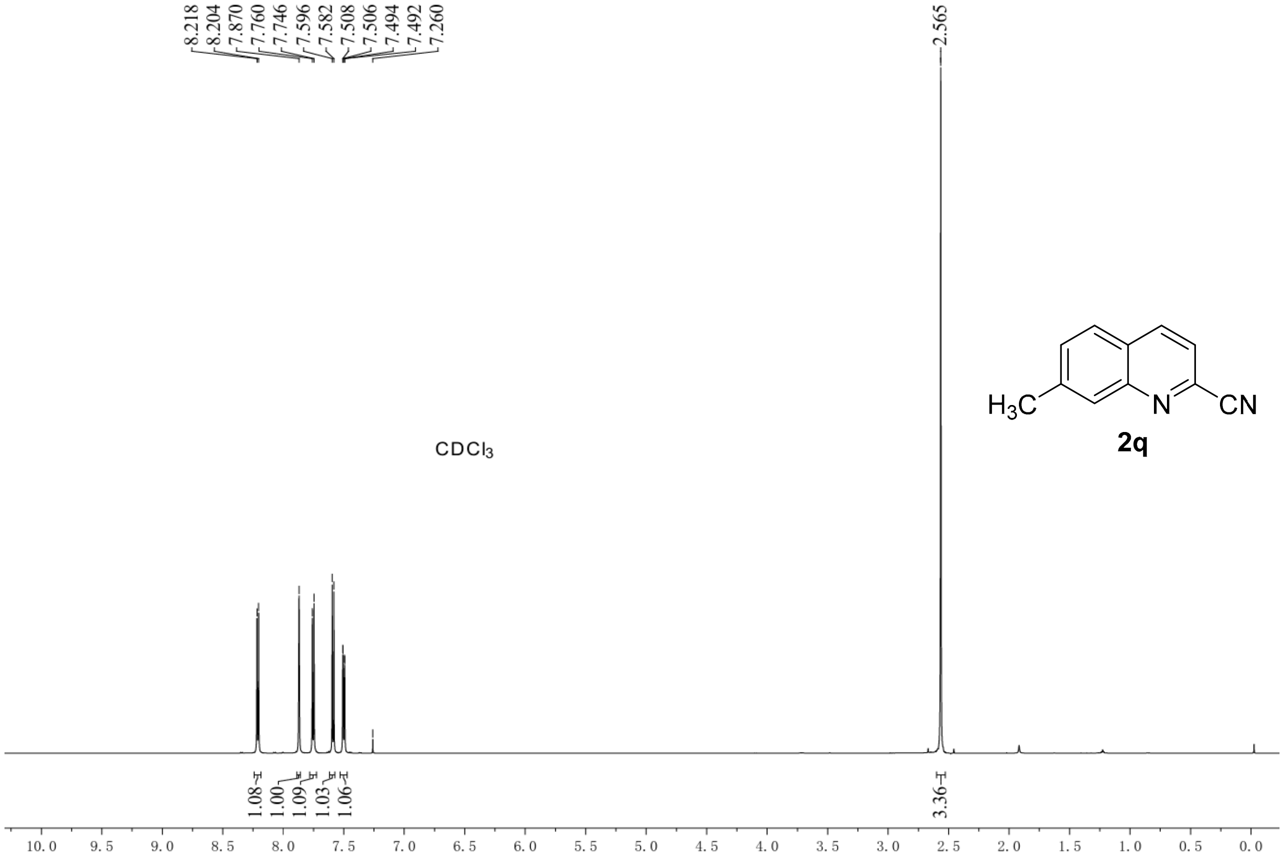

^{1}H NMR (600 MHz, CDCl$_3$):

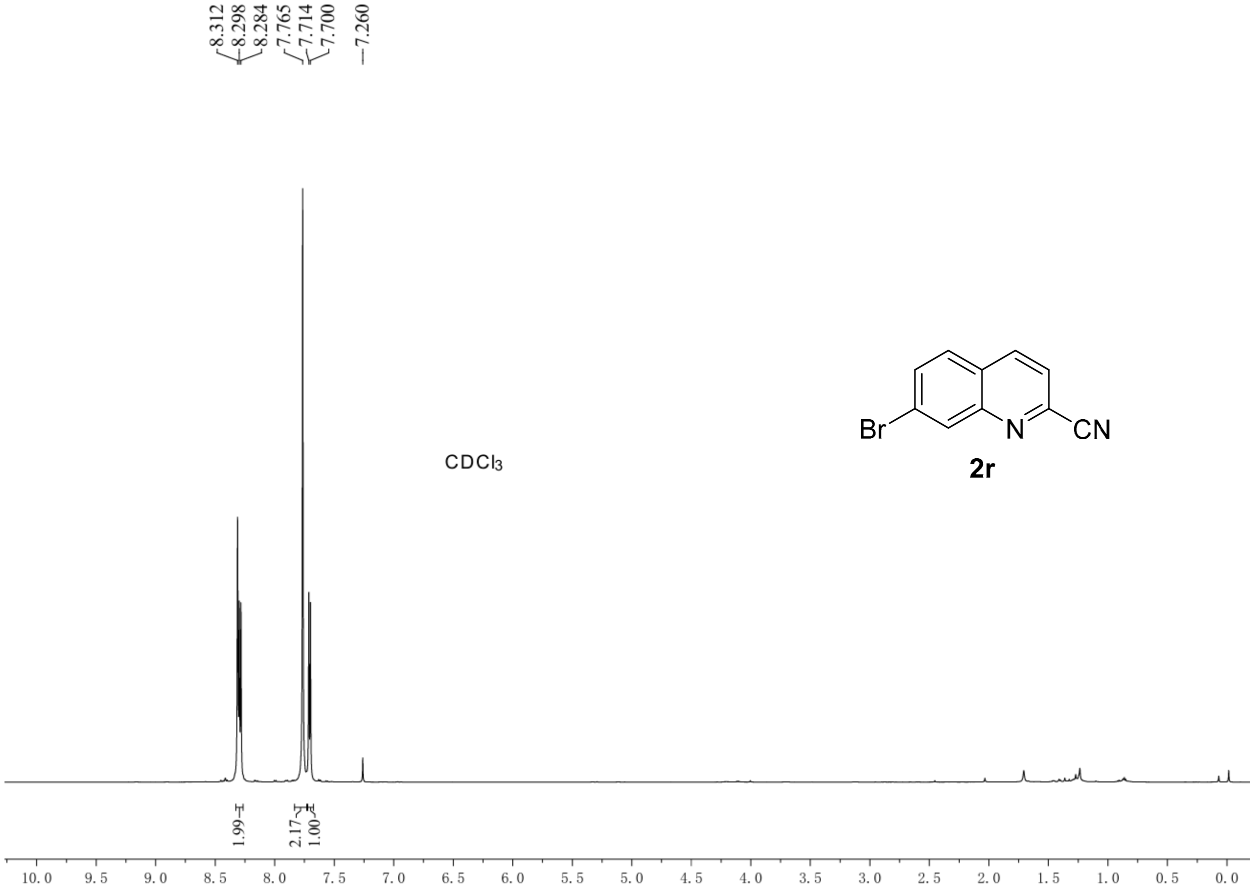

^{1}H NMR (600 MHz, $CDCl_3$):

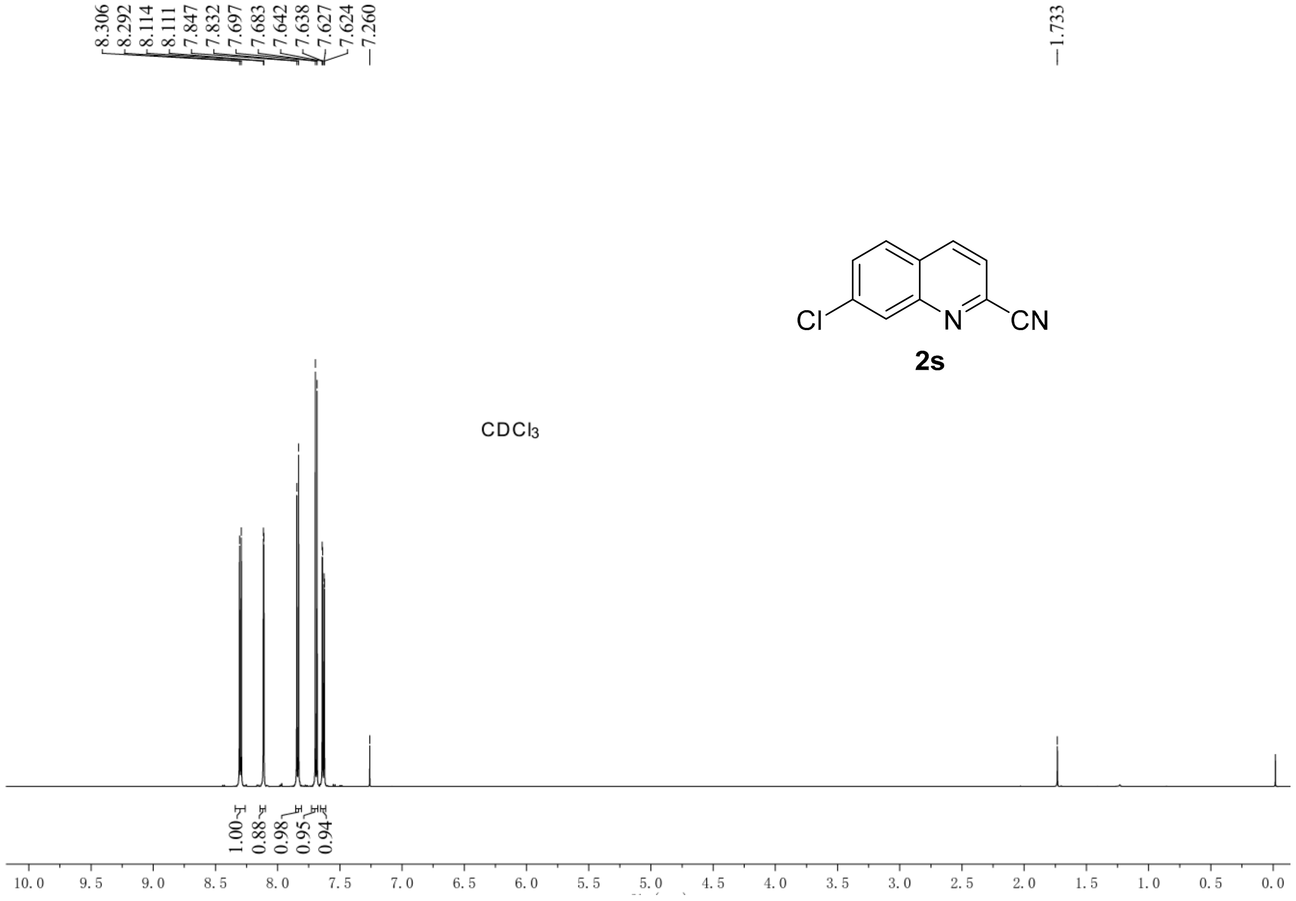

^{1}H NMR (600 MHz, $CDCl_3$):

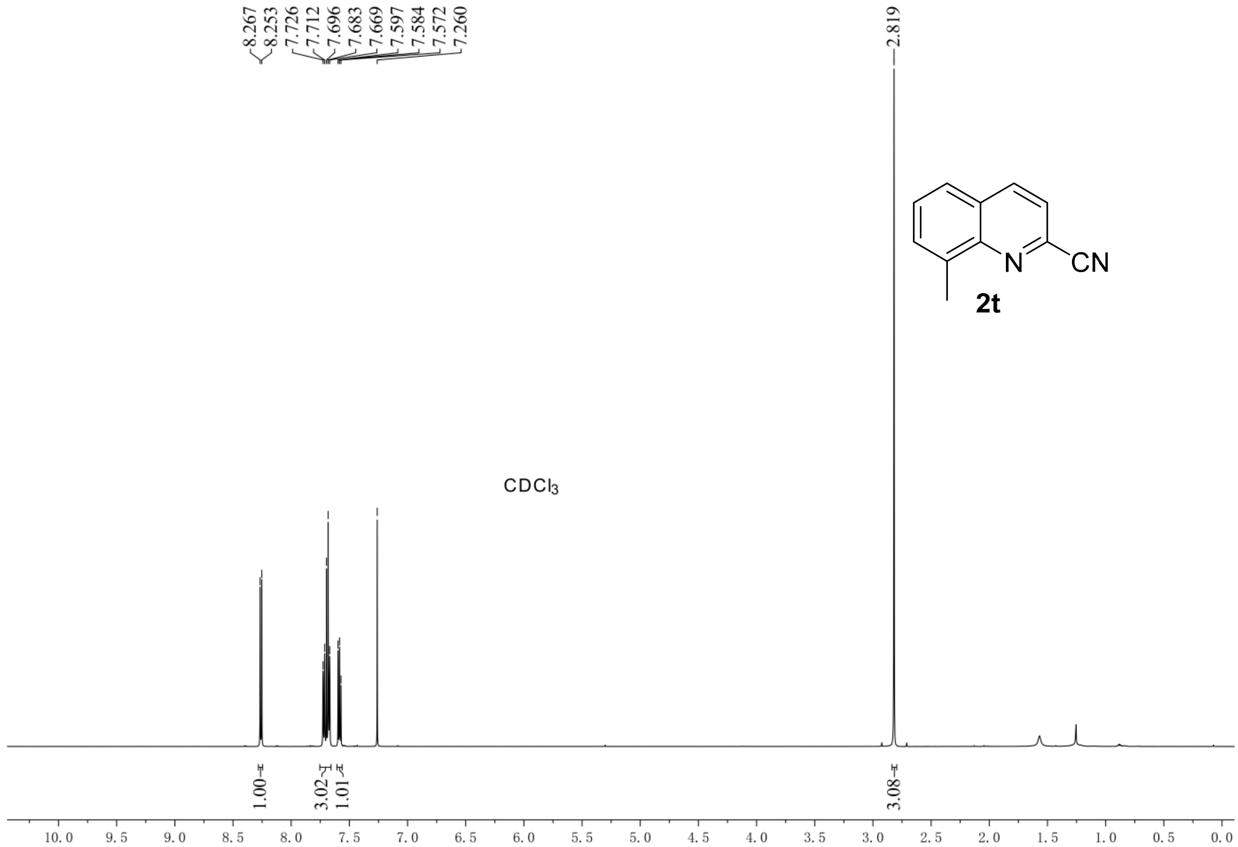

^{1}H NMR (600 MHz, $CDCl_3$):

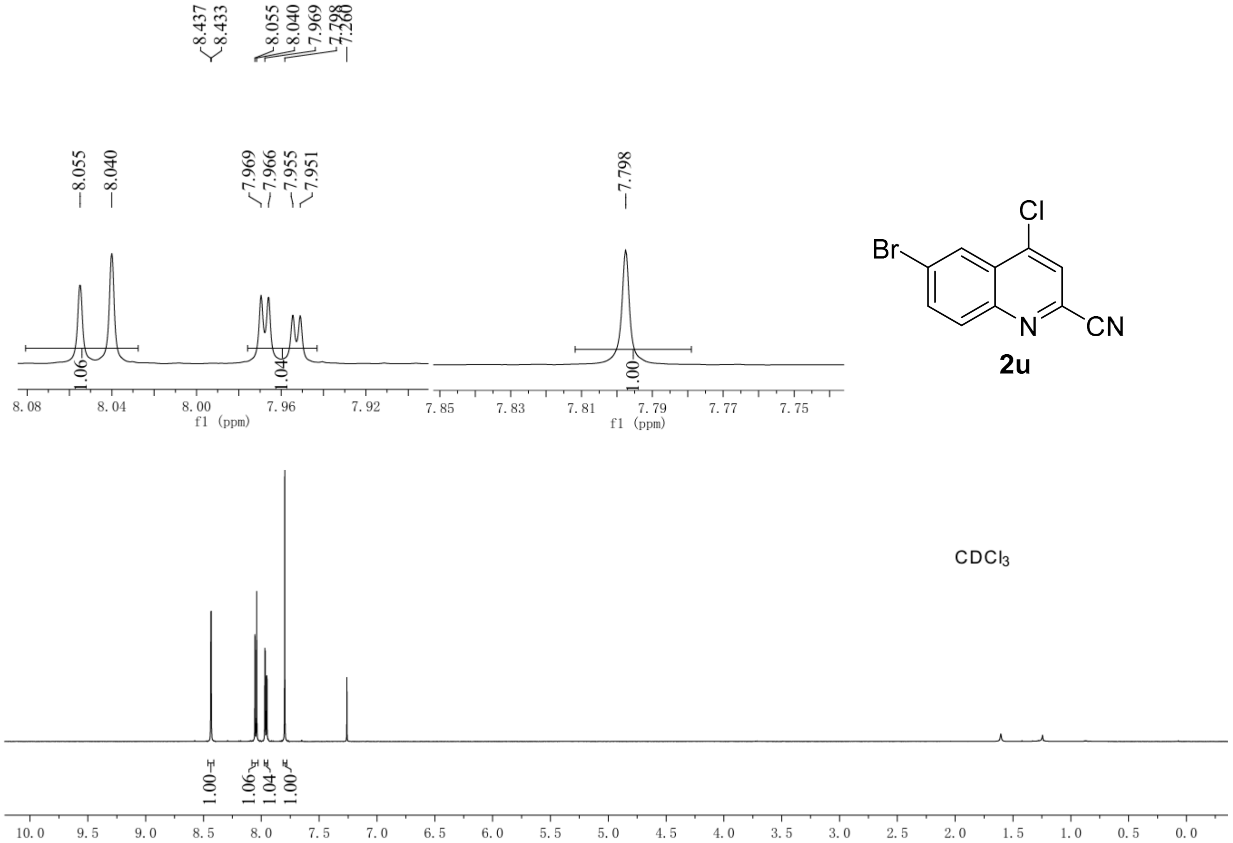

^{13}C NMR (150 MHz, $CDCl_3$):

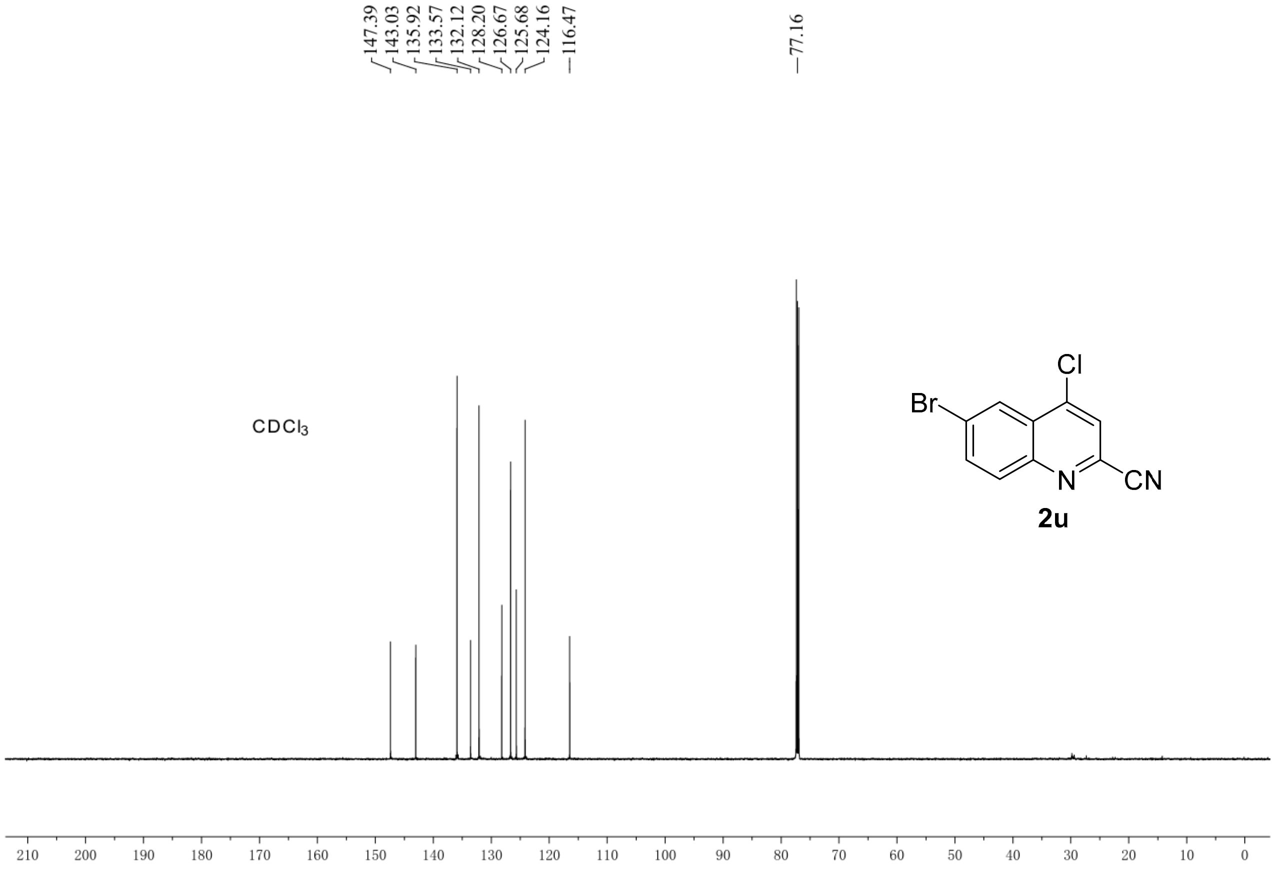

^{1}H NMR (600 MHz, $CDCl_3$):

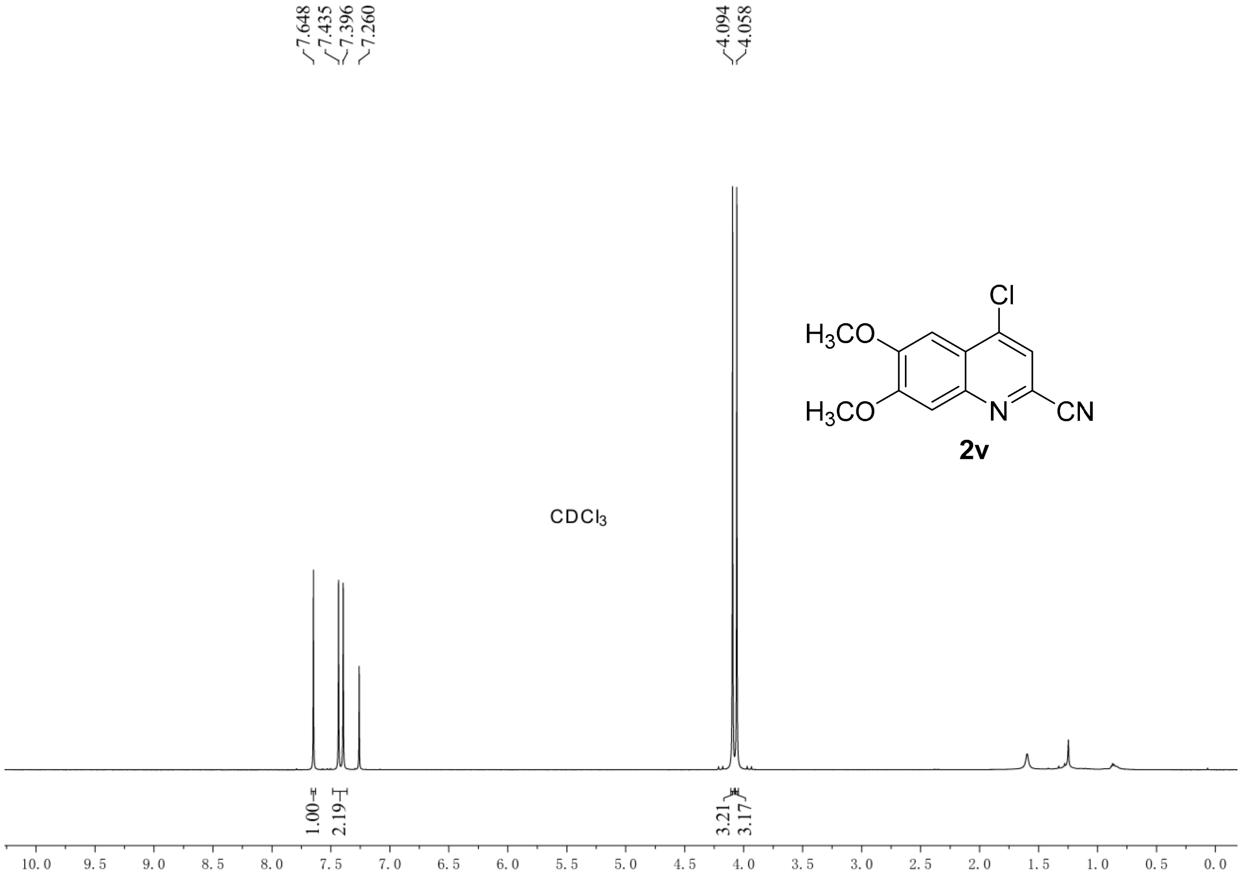

^{1}H NMR (600 MHz, $CDCl_3$):

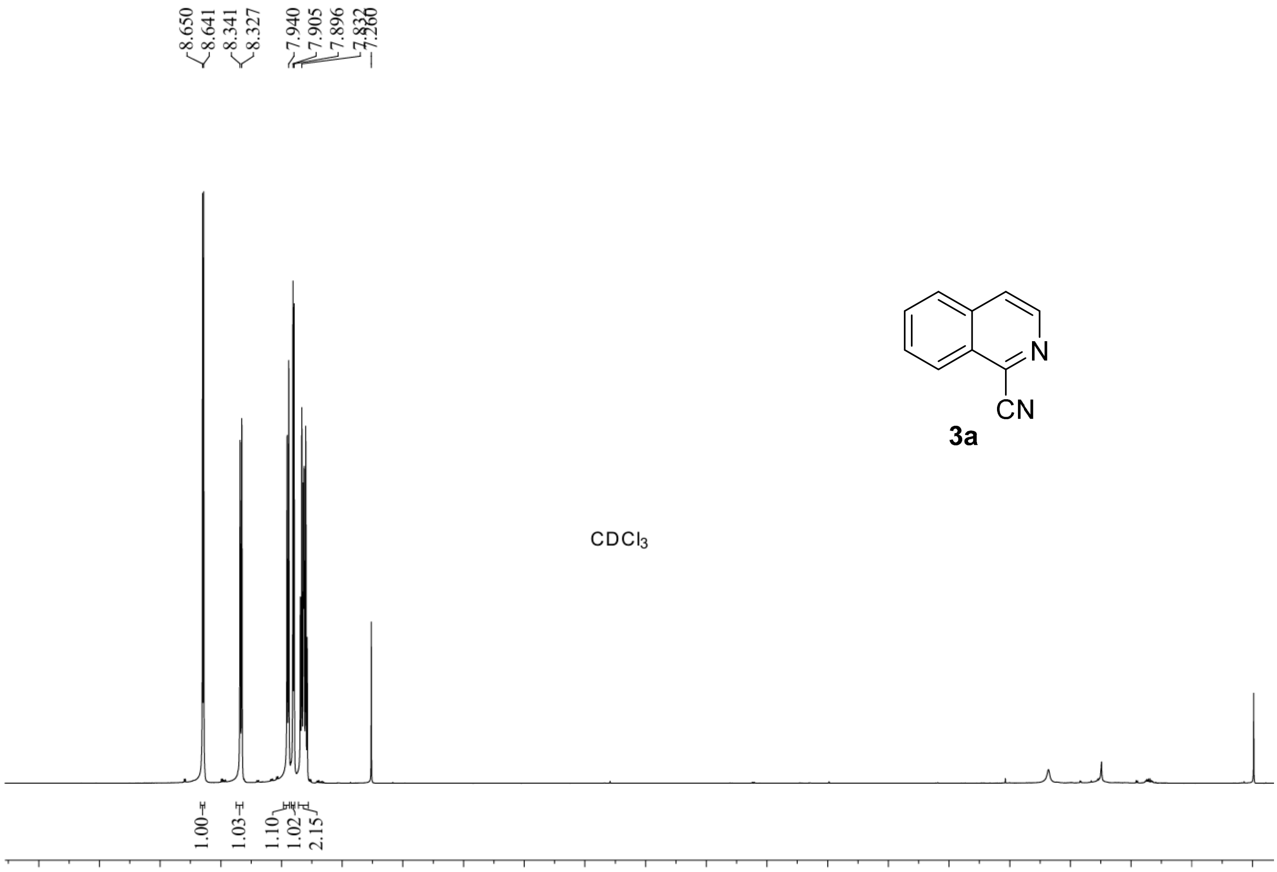

^{1}H NMR (600 MHz, CDCl$_3$):

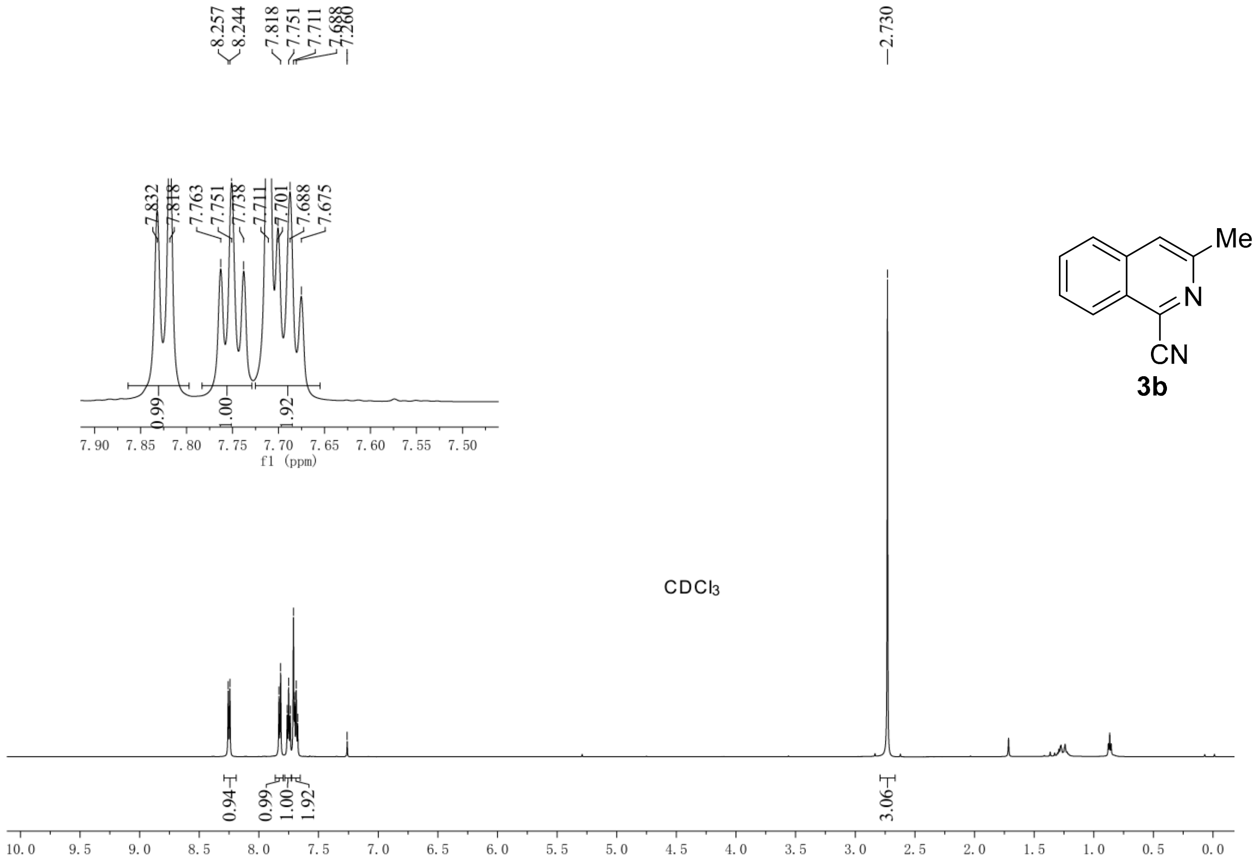

^{1}H NMR (600 MHz, CDCl$_3$):

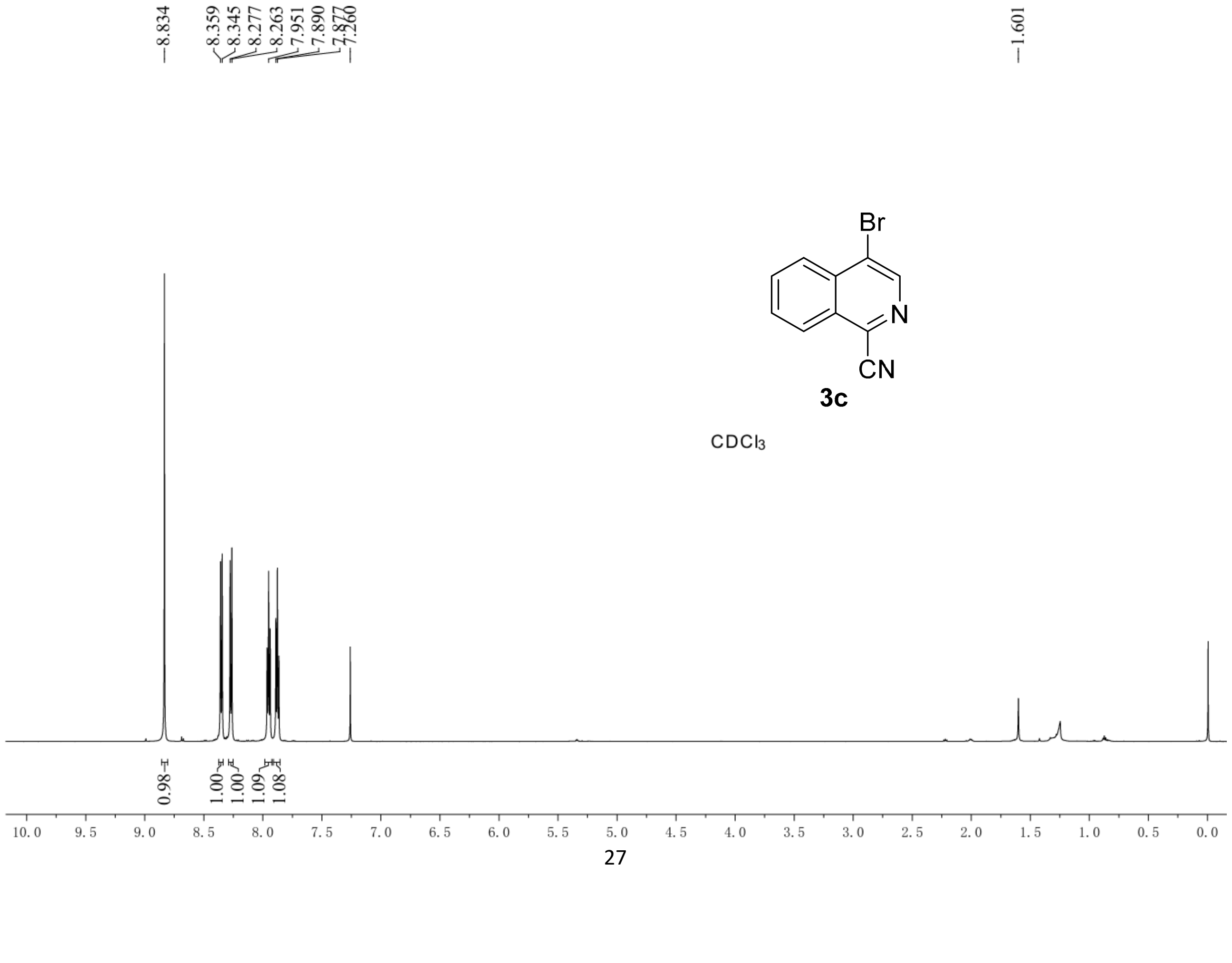

^{1}H NMR (600 MHz, $CDCl_3$):

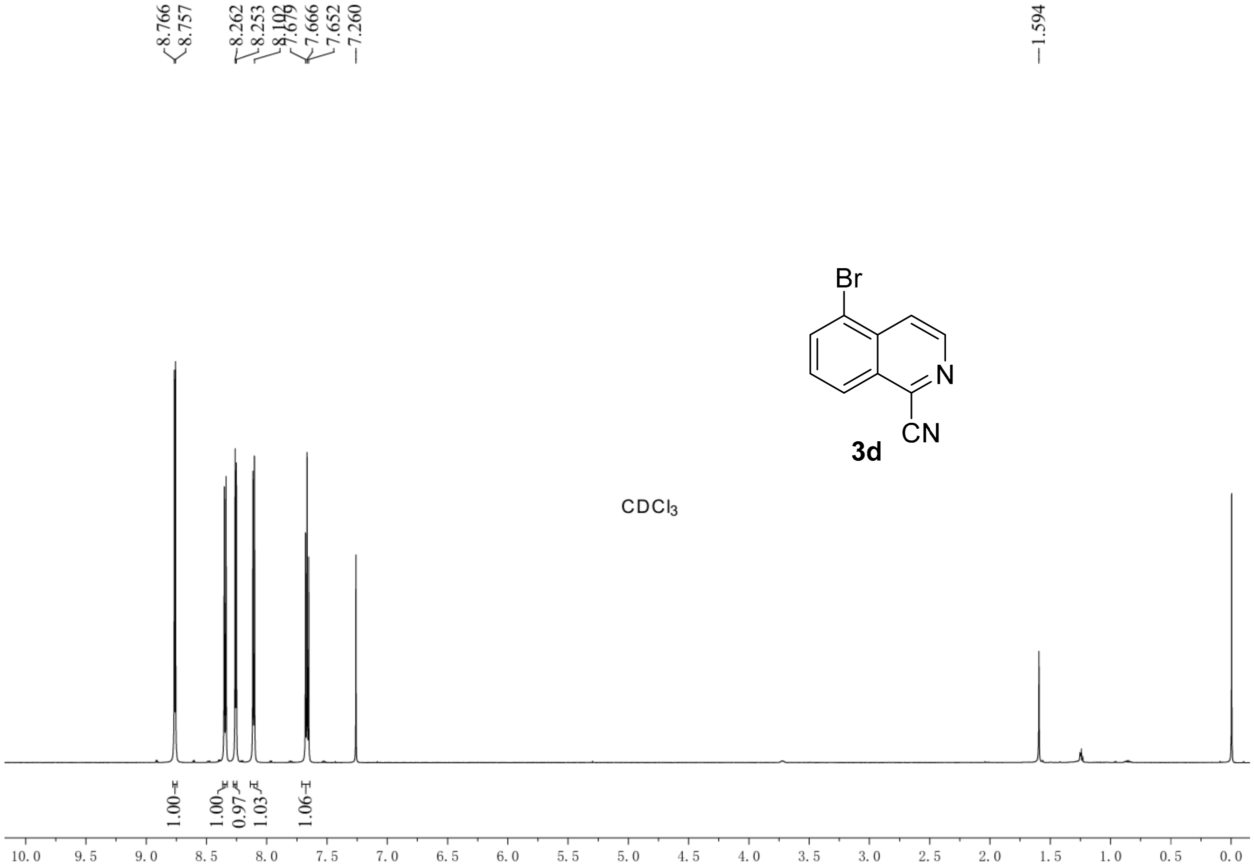

^{1}H NMR (600 MHz, $CDCl_3$):

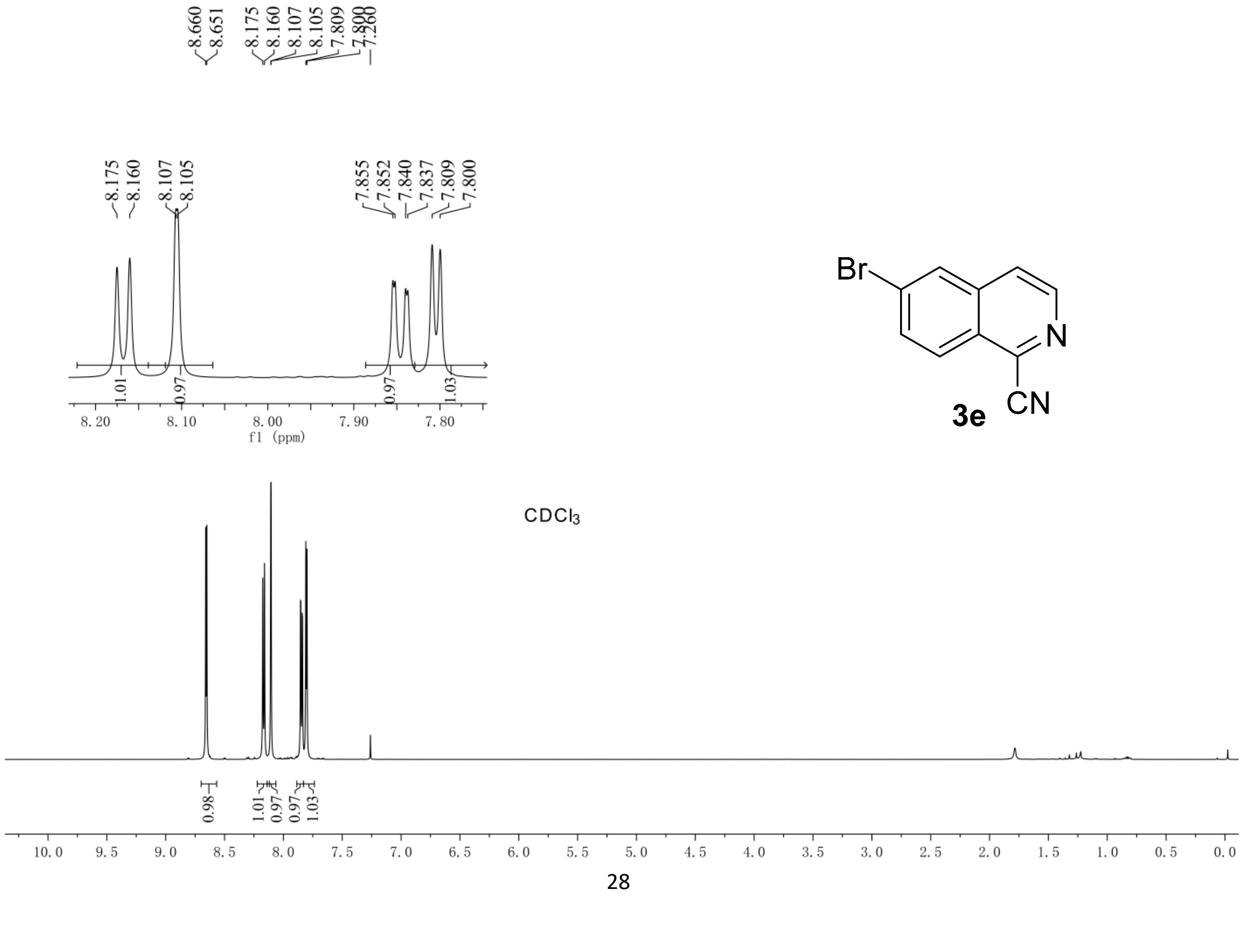

^{1}H NMR (600 MHz, $CDCl_3$):

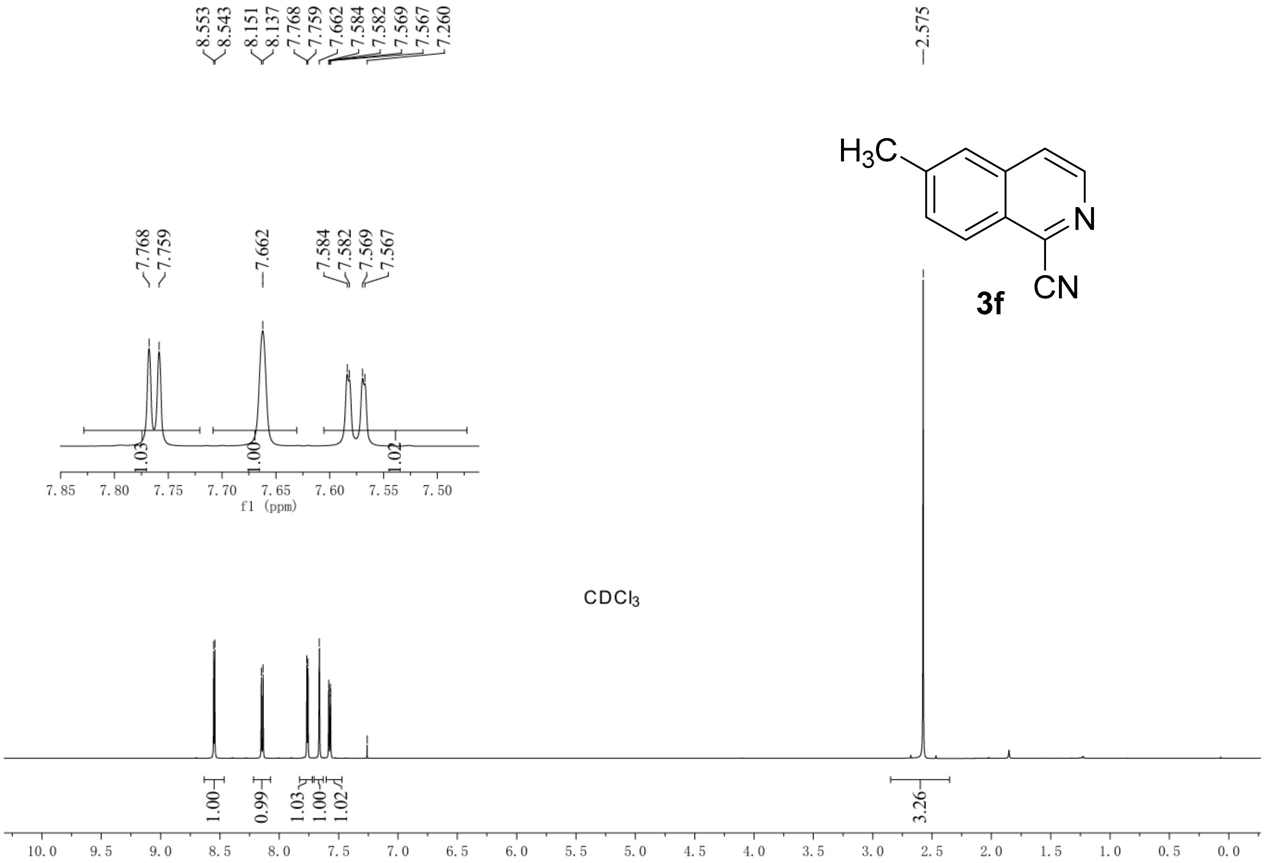

^{1}H NMR (600 MHz, $CDCl_3$):

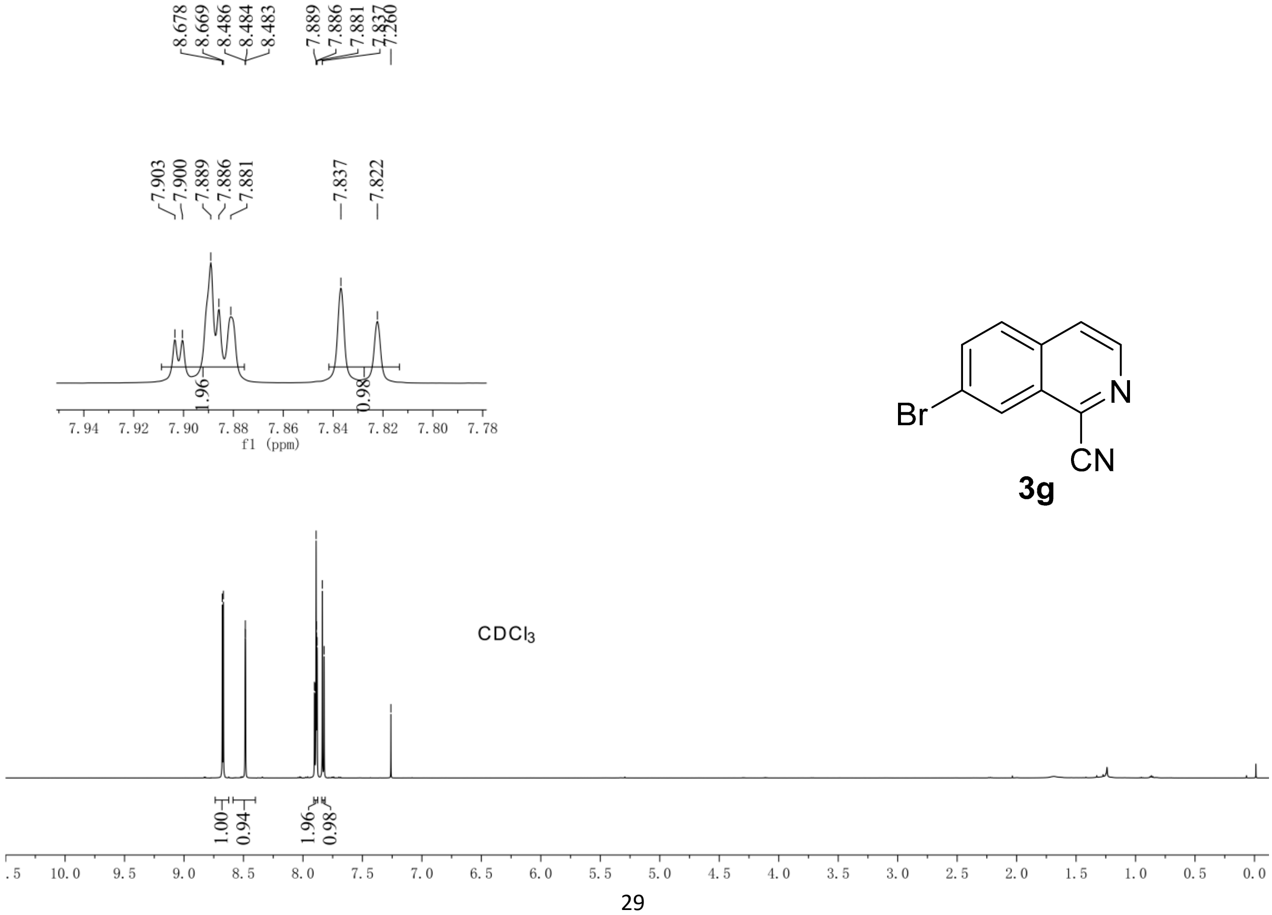

^{13}C NMR (150 MHz, $CDCl_3$):

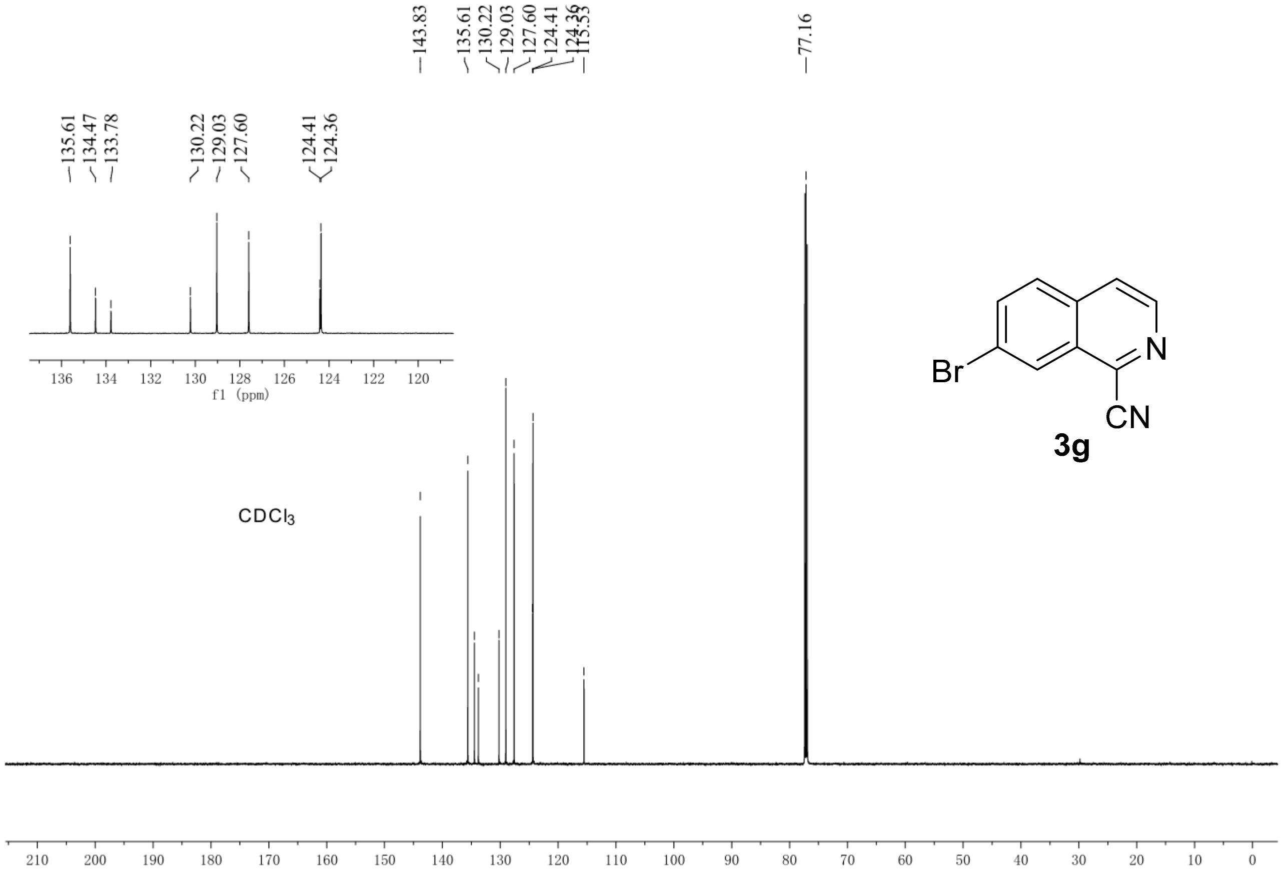

^{1}H NMR (600 MHz, $CDCl_3$):

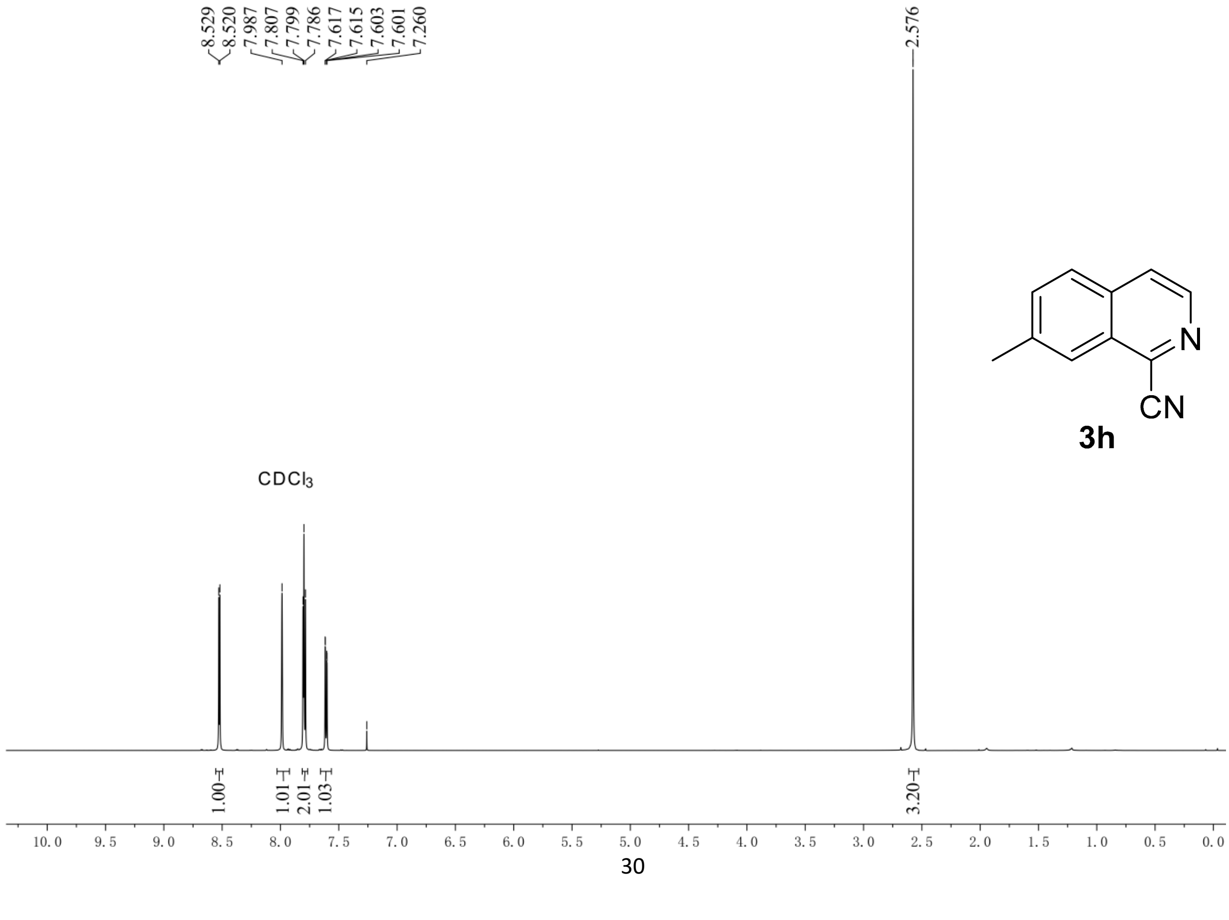

^{13}C NMR (150 MHz, $CDCl_3$):

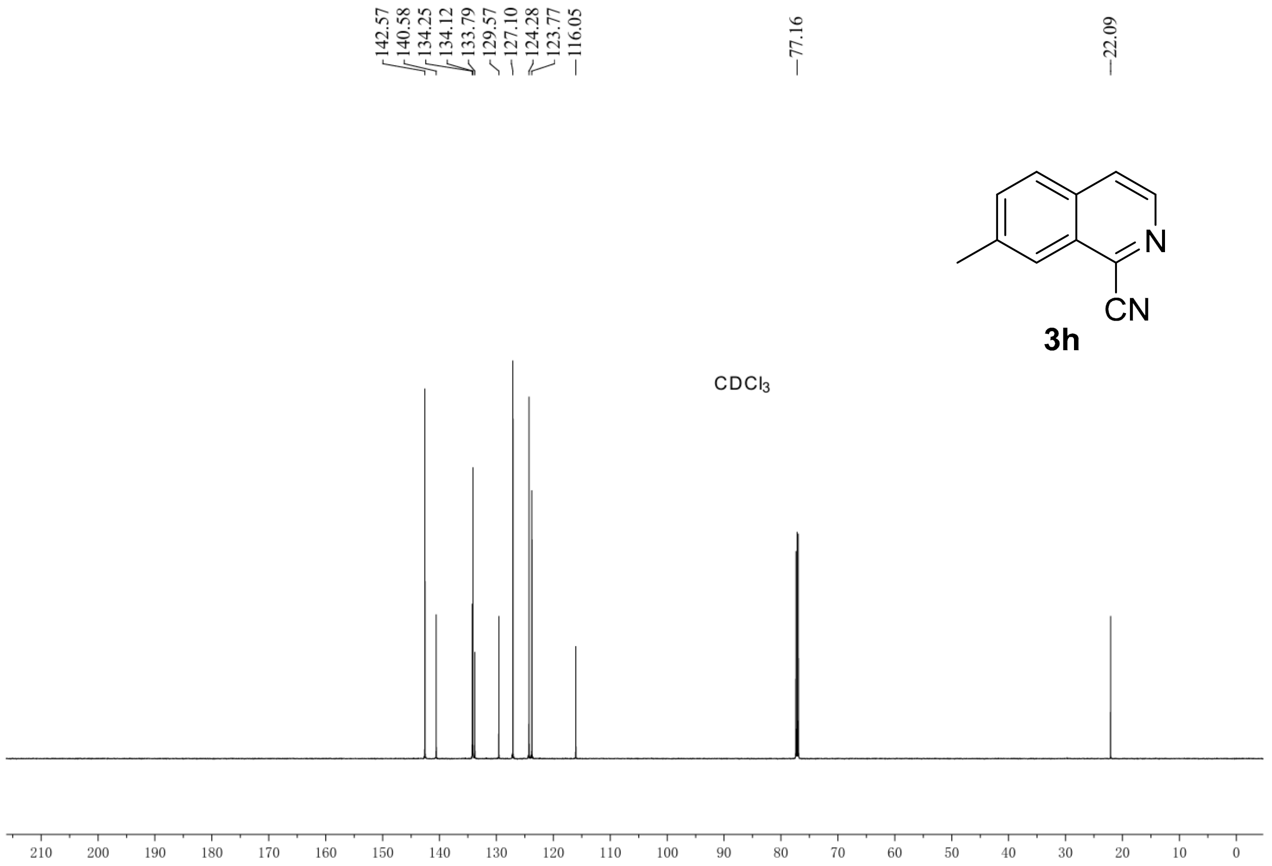

3h

^{1}H NMR (600 MHz, $CDCl_3$):

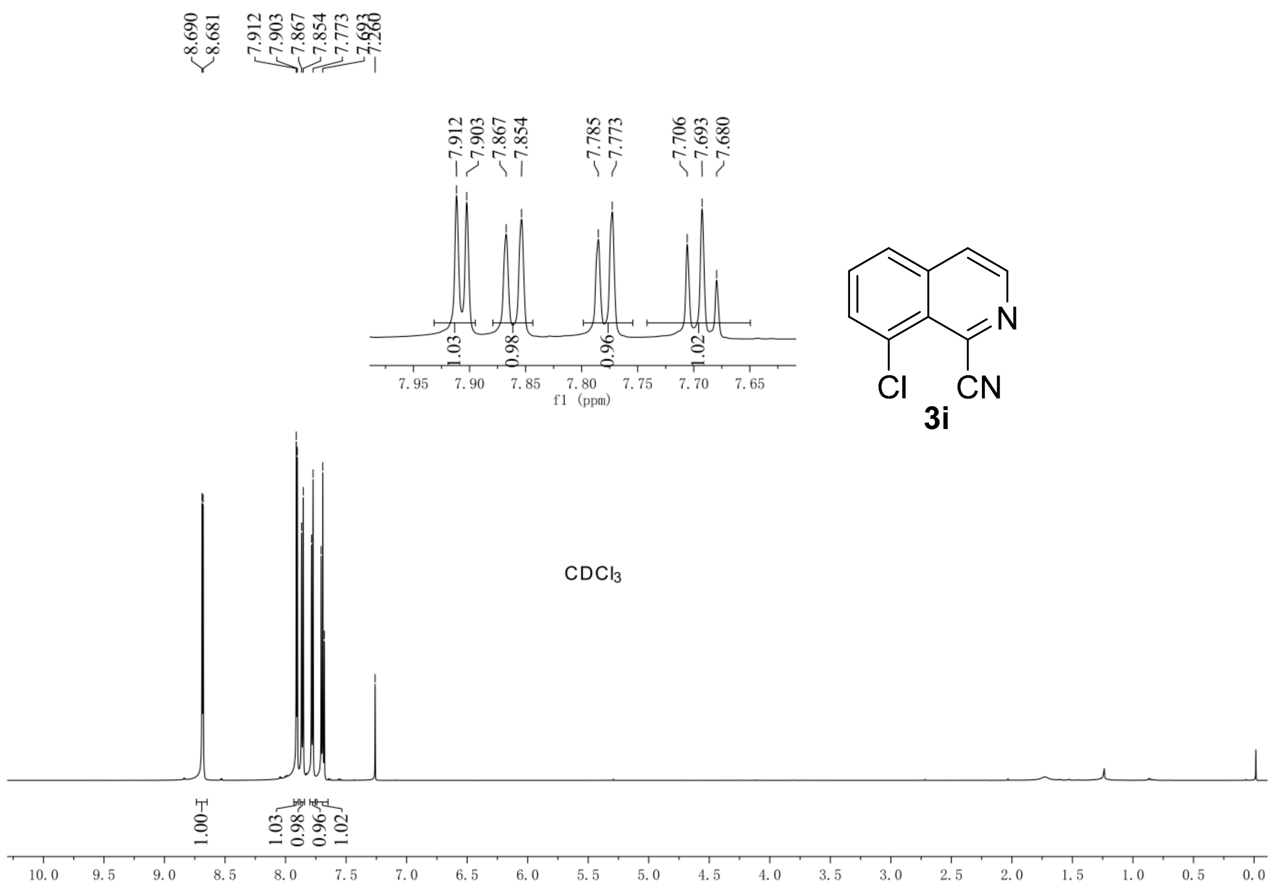

3i

^{13}C NMR (150 MHz, $CDCl_3$):

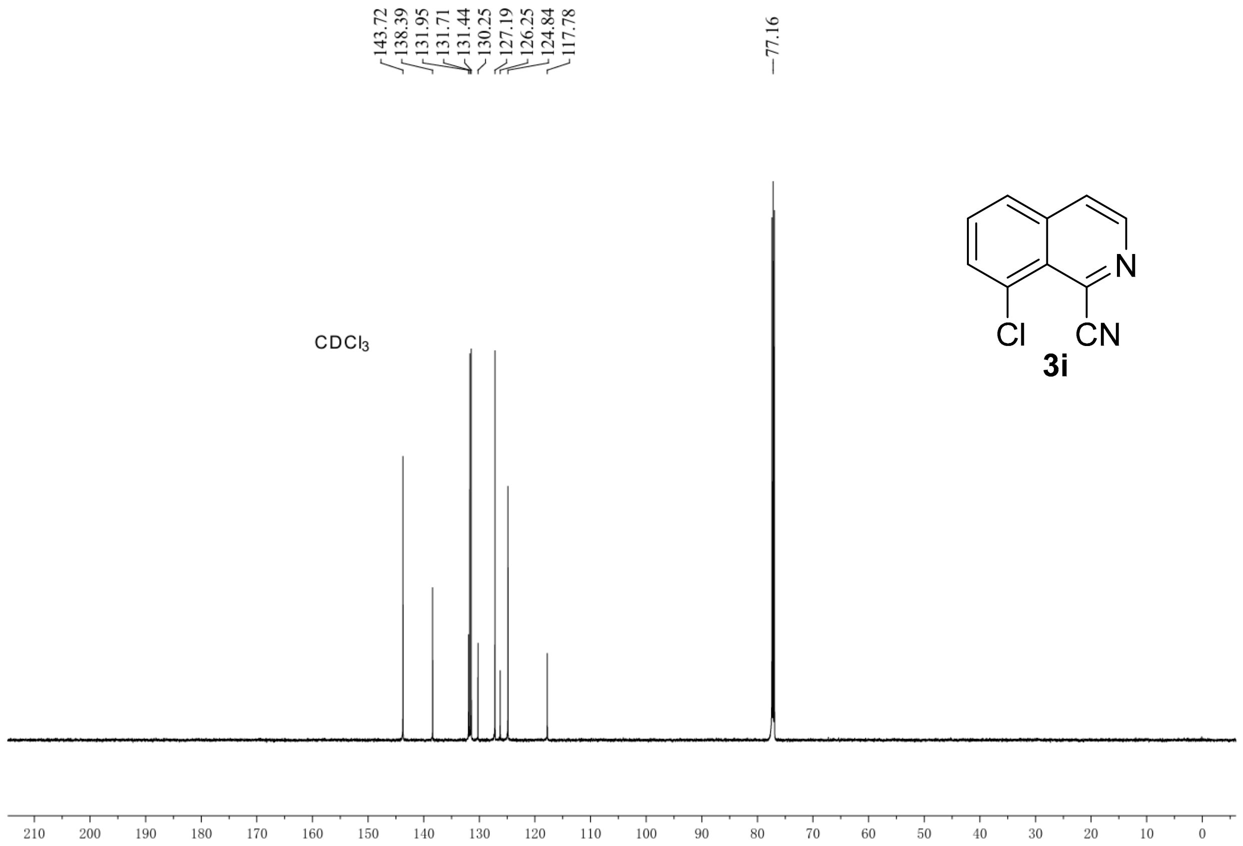

3i

^{1}H NMR (600 MHz, $CDCl_3$):

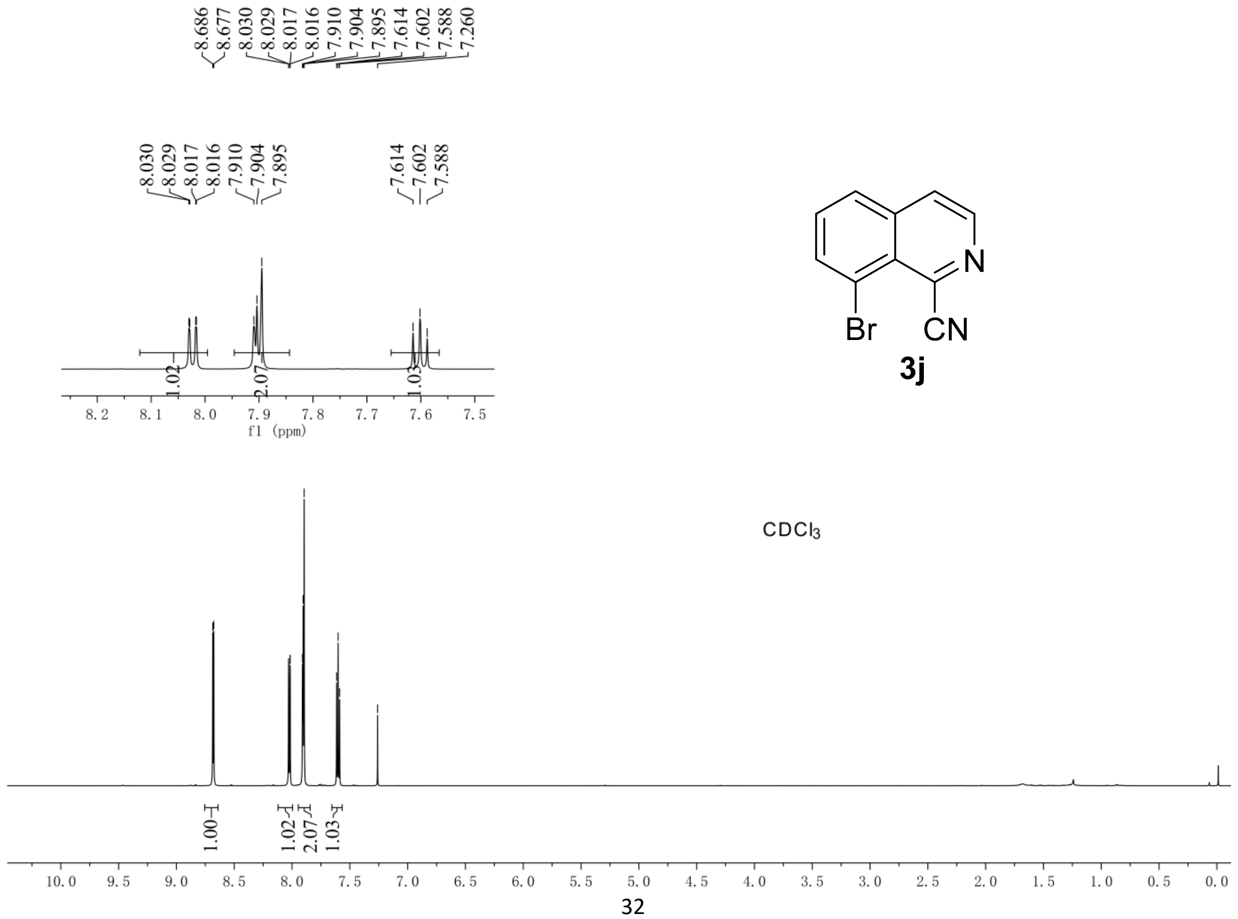

3j

^{13}C NMR (150 MHz, CDCl$_3$):

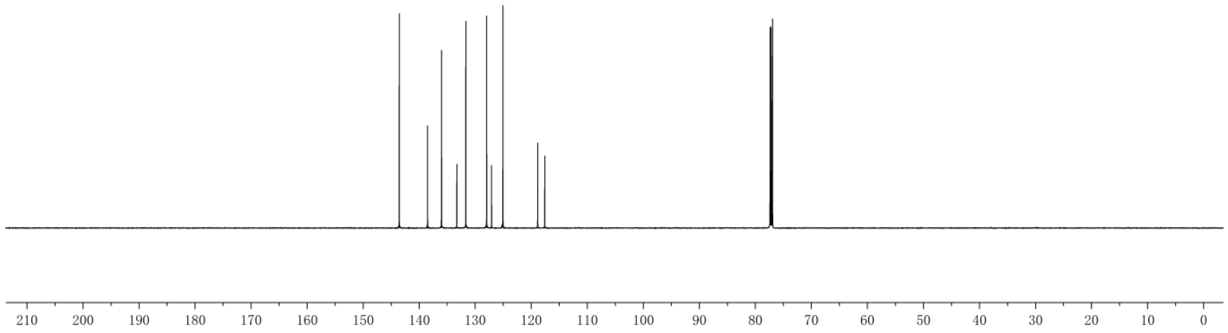

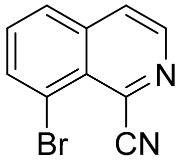

3j

^{1}H NMR (600 MHz, CDCl$_3$):

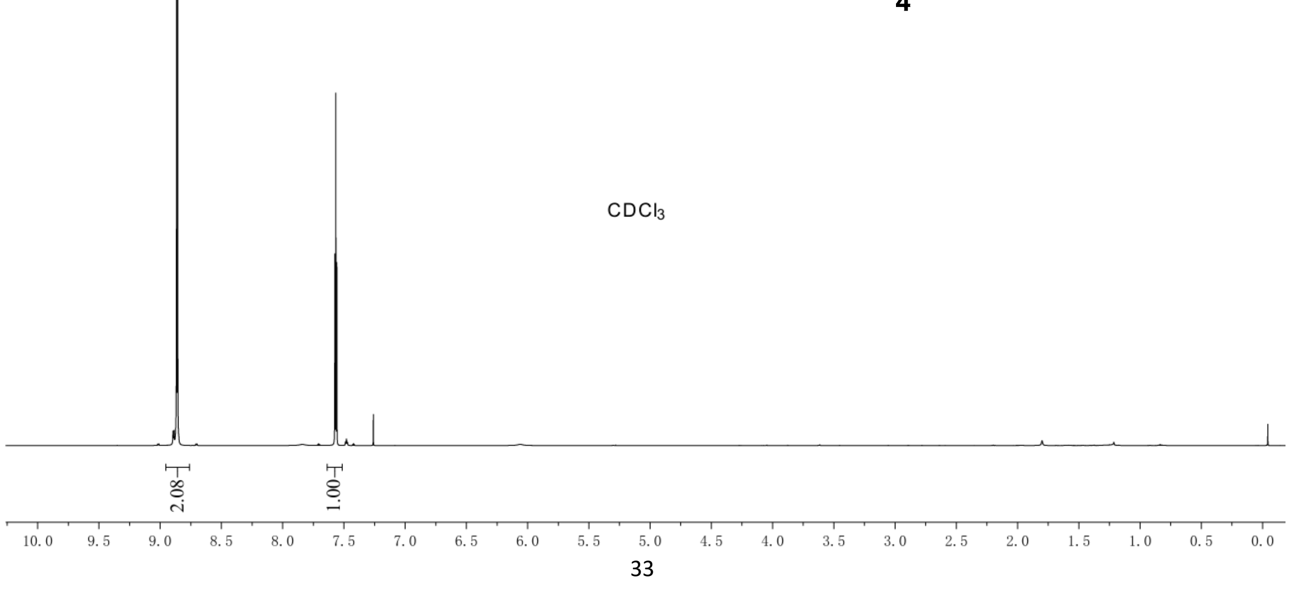

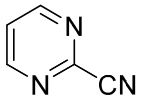

4

^{1}H NMR (600 MHz, $CDCl_3$):

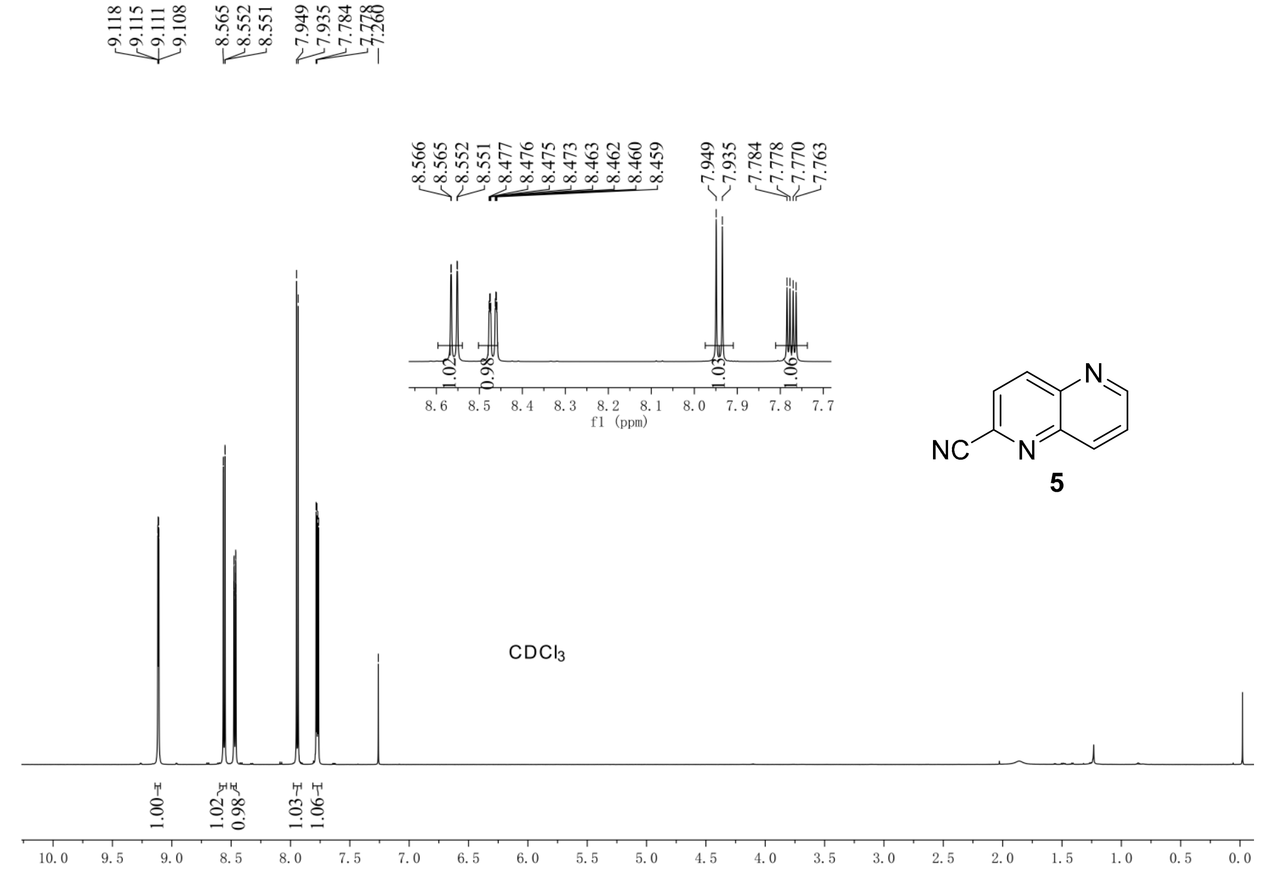

^{1}H NMR (600 MHz, $CDCl_3$):

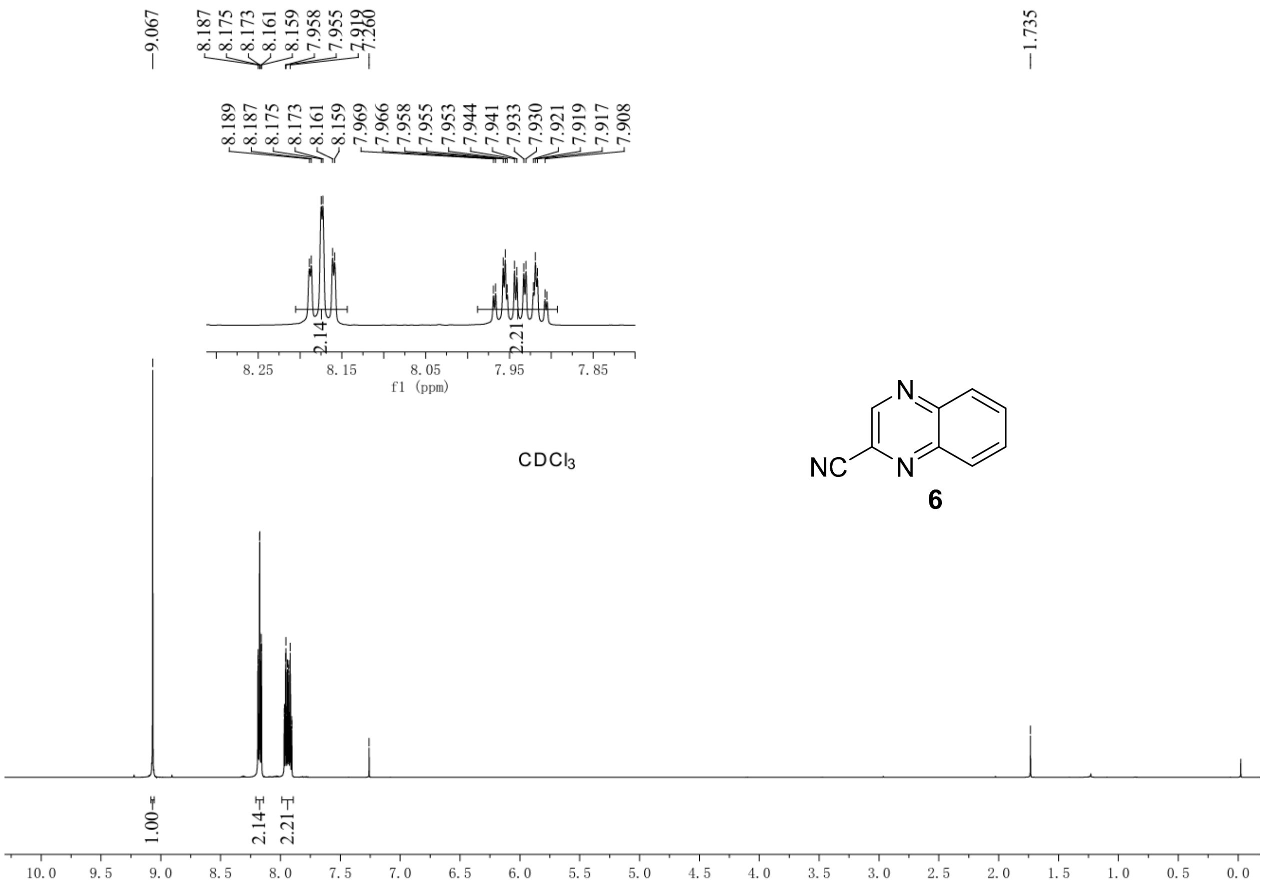

^{1}H NMR (600 MHz, CDCl$_3$):

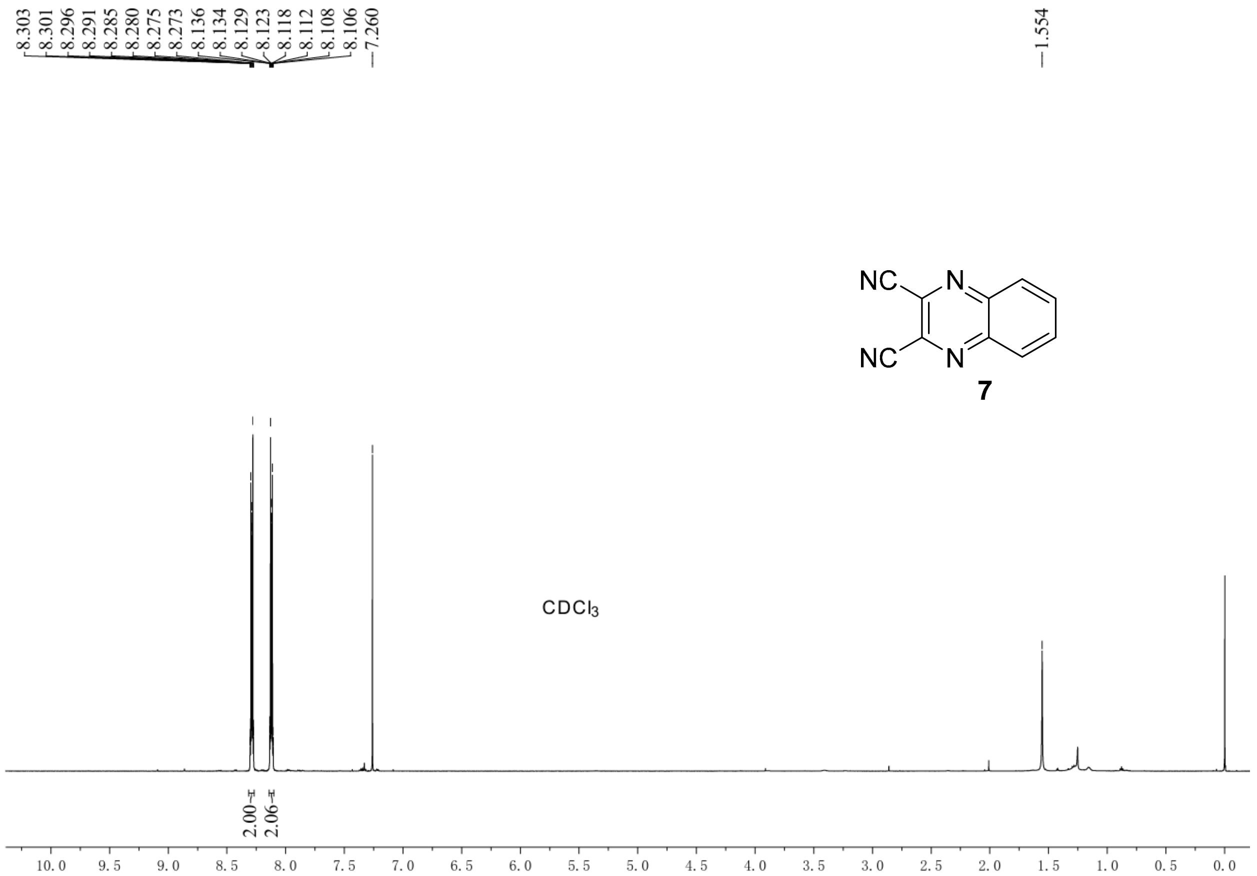

^{1}H NMR (600 MHz, CDCl$_3$):

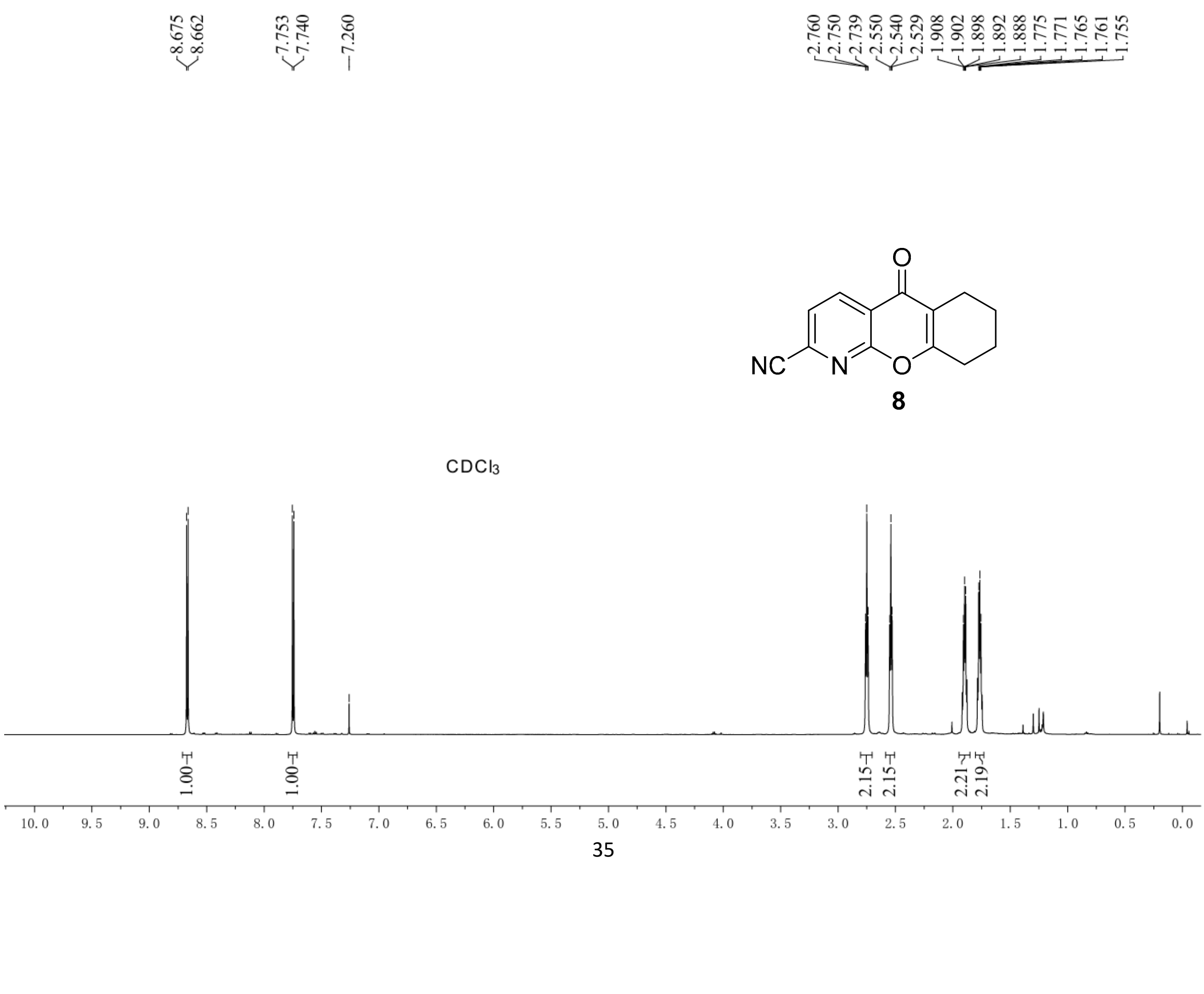

^{13}C NMR (150 MHz, $CDCl_3$):

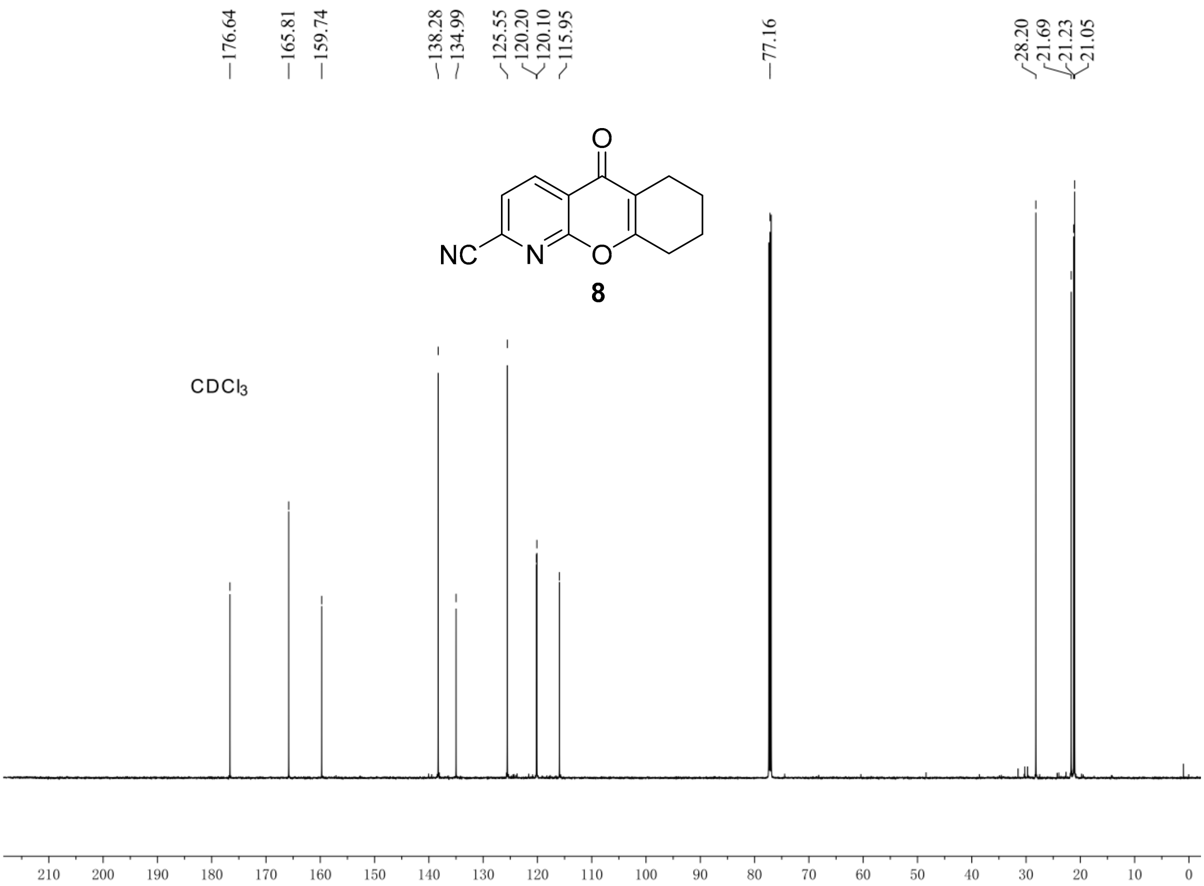

^{1}H NMR (600 MHz, $CDCl_3$):

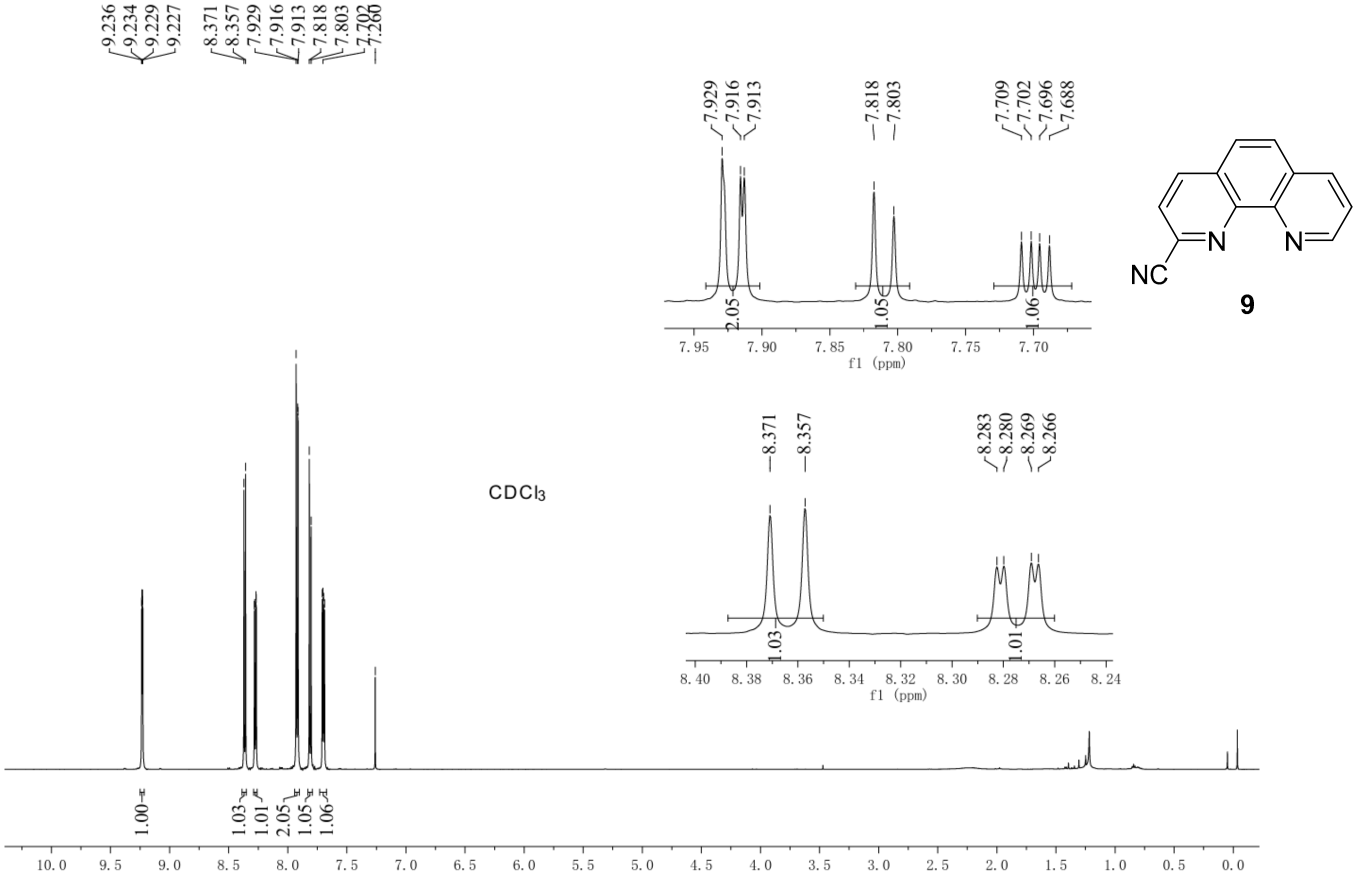

^{1}H NMR (600 MHz, $CDCl_3$):

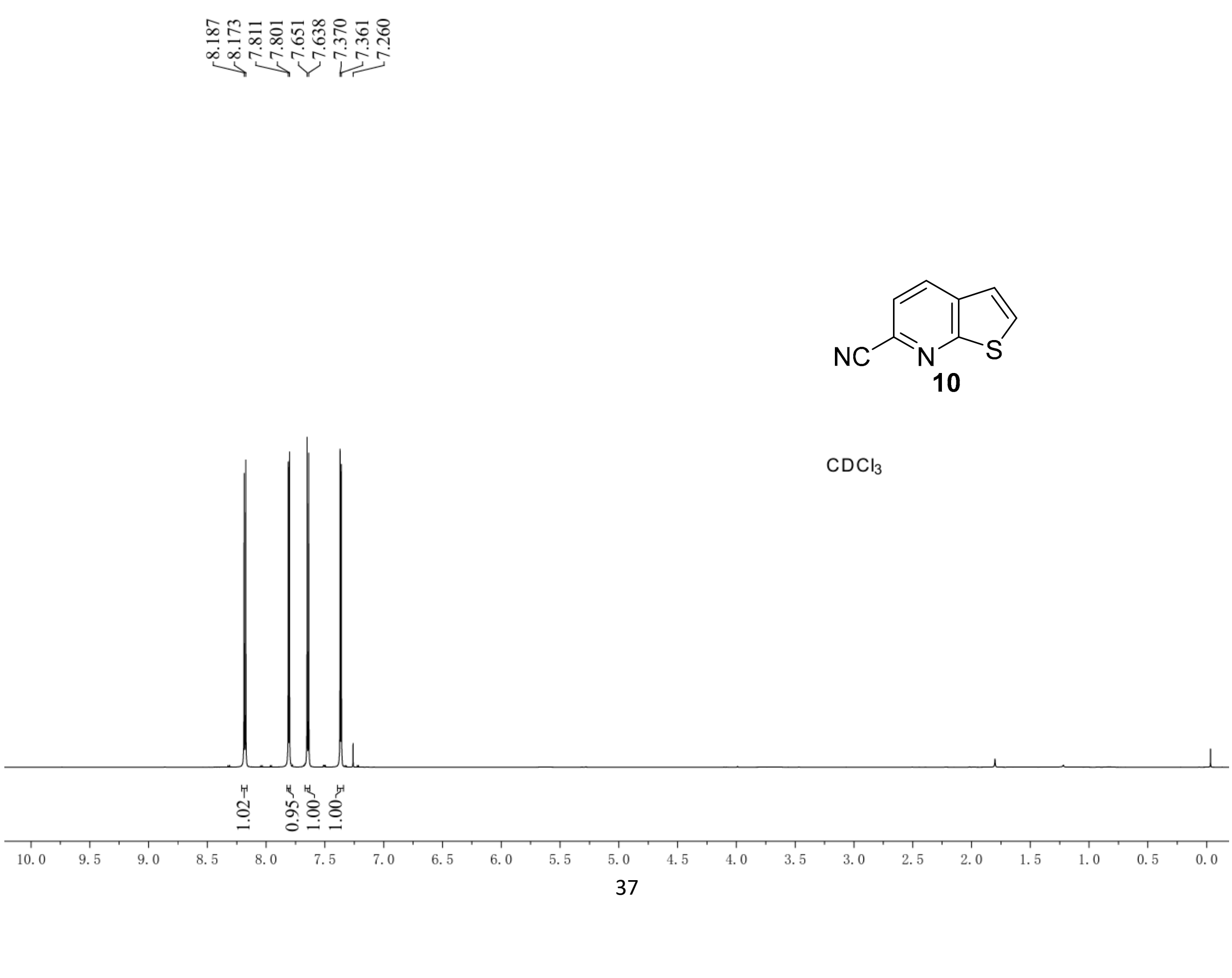

^{1}H NMR (600 MHz, DMSO-d_6):

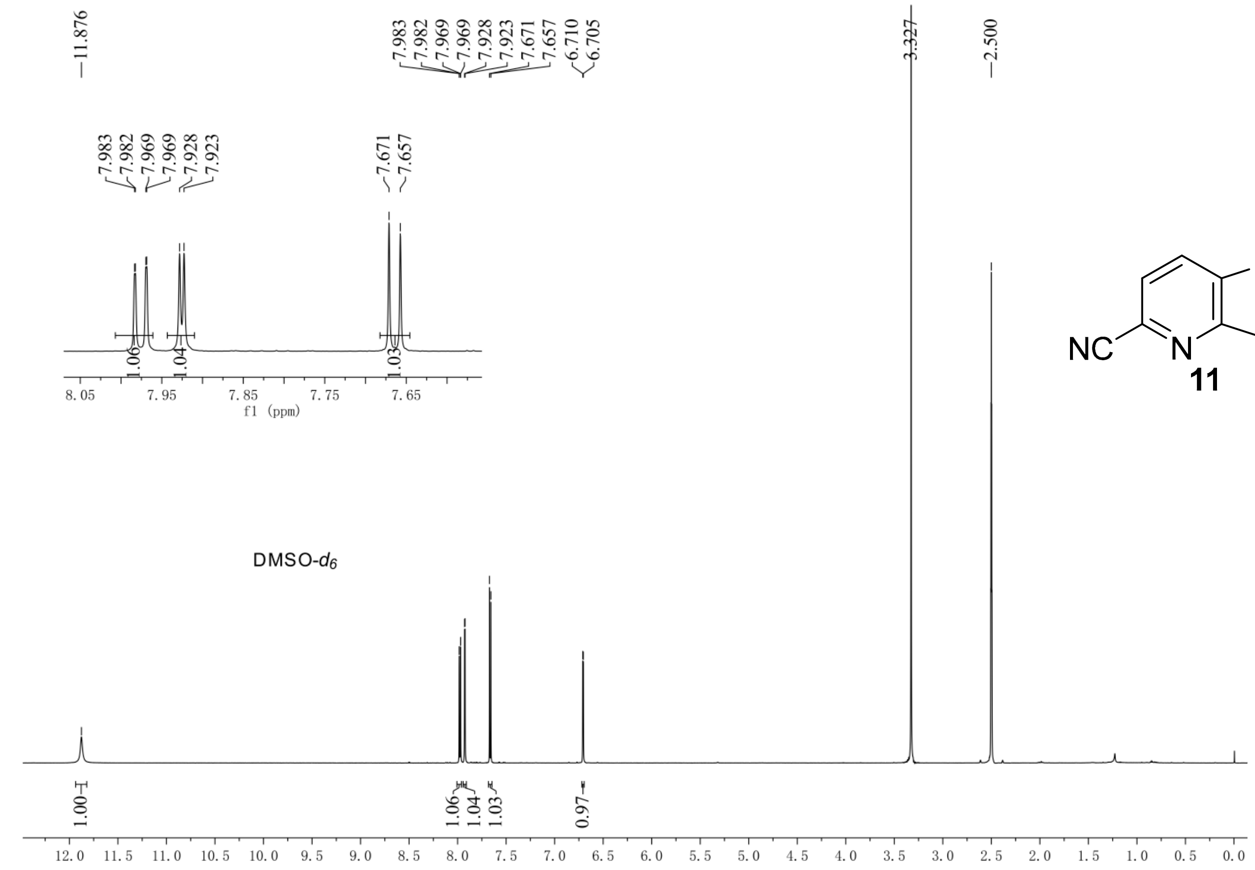

^{1}H NMR (600 MHz, $CDCl_3$):

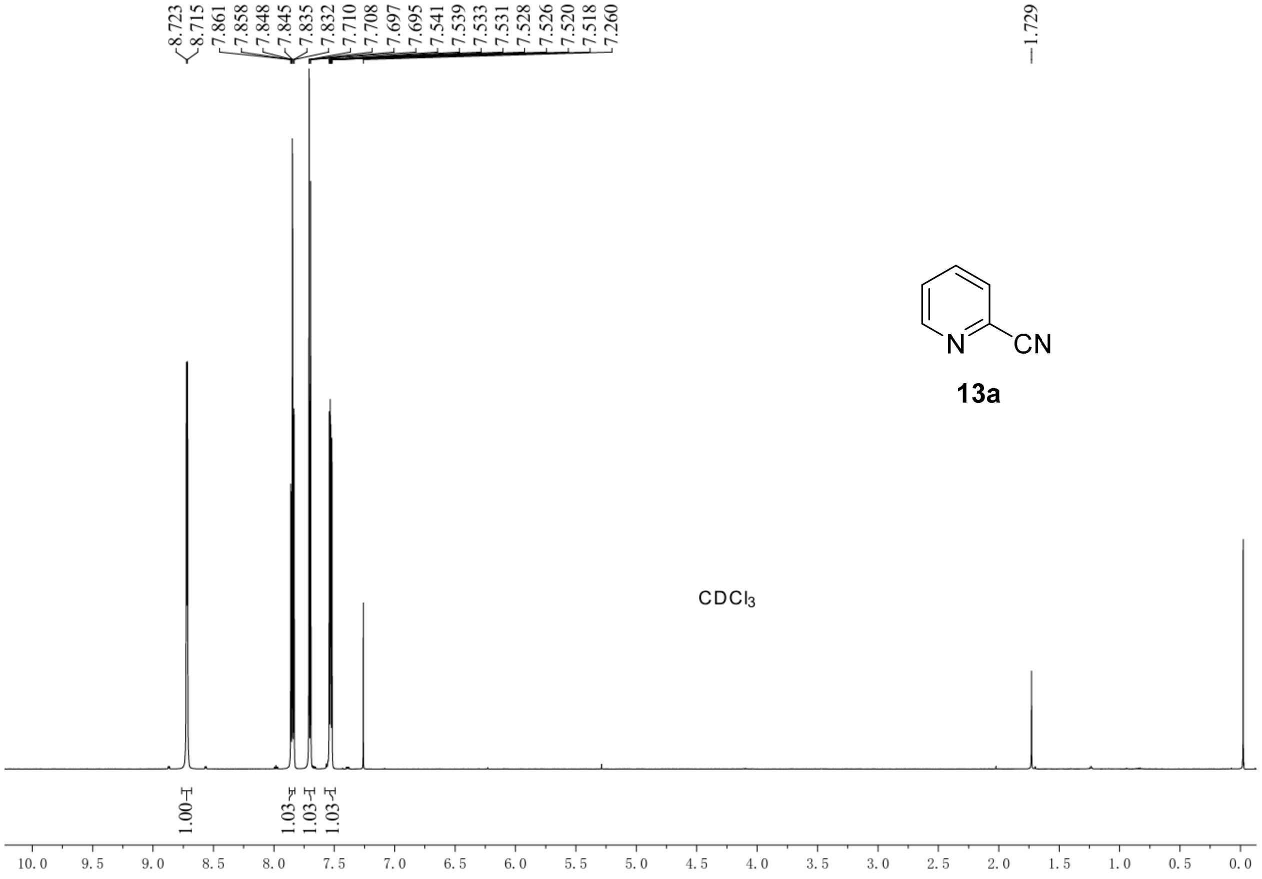

^{1}H NMR (600 MHz, $CDCl_3$):

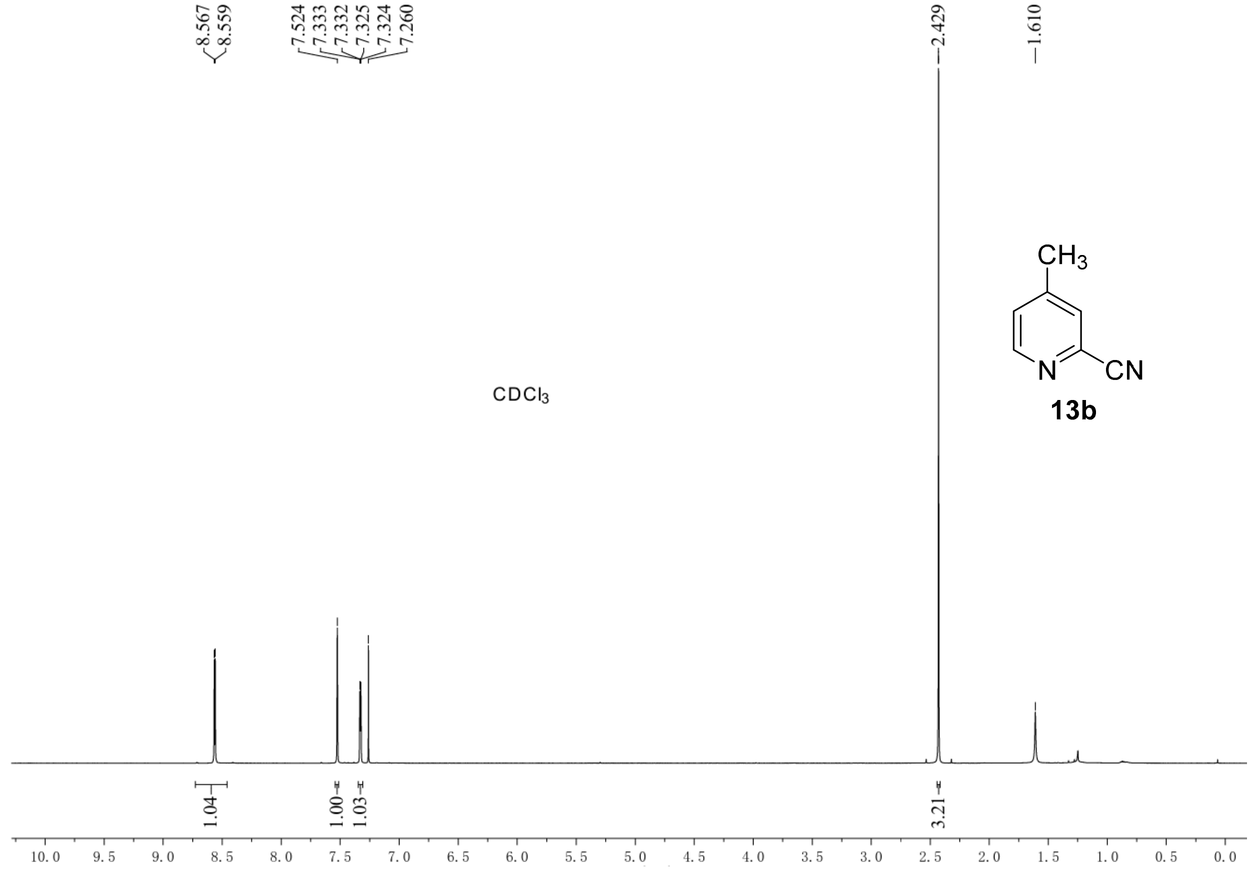

^{1}H NMR (600 MHz, $CDCl_3$):

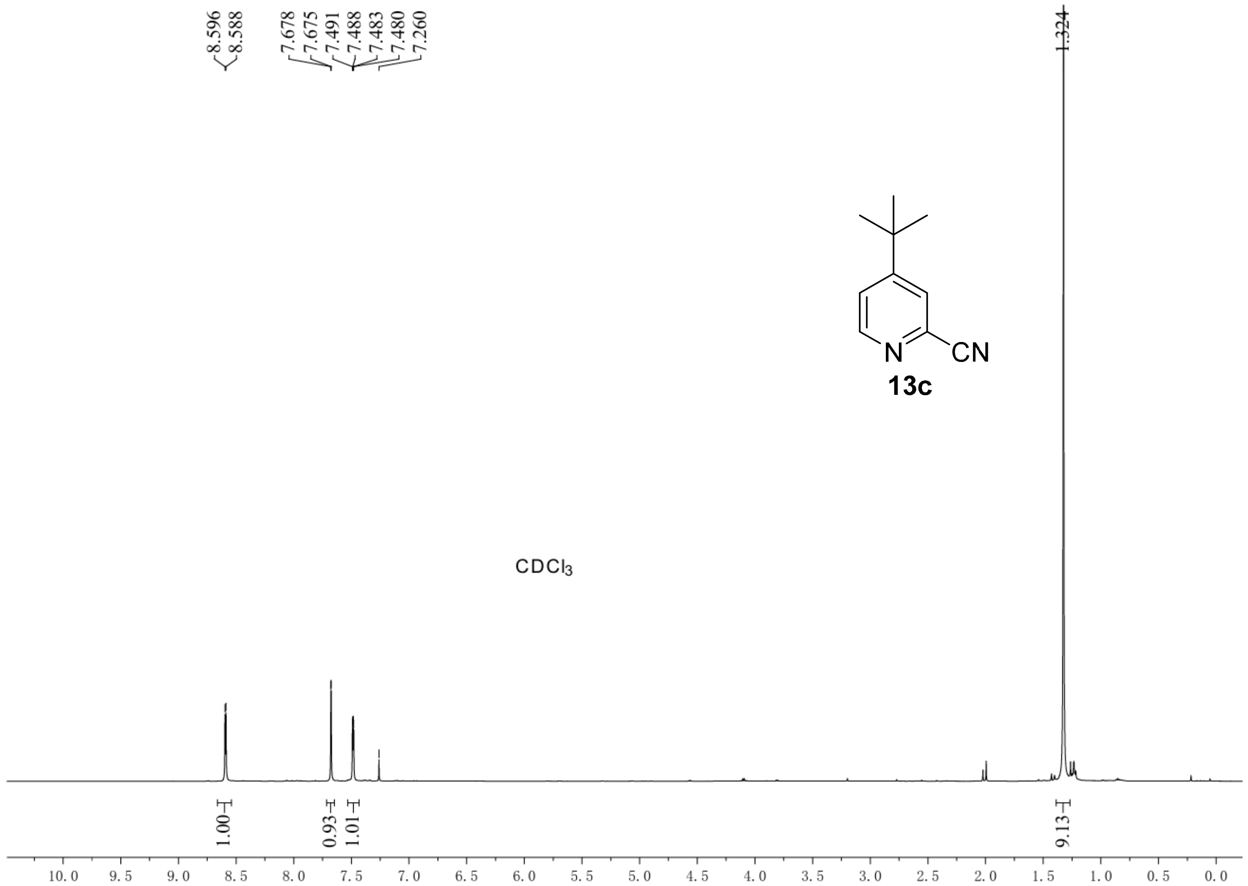

^{1}H NMR (600 MHz, CDCl$_3$):

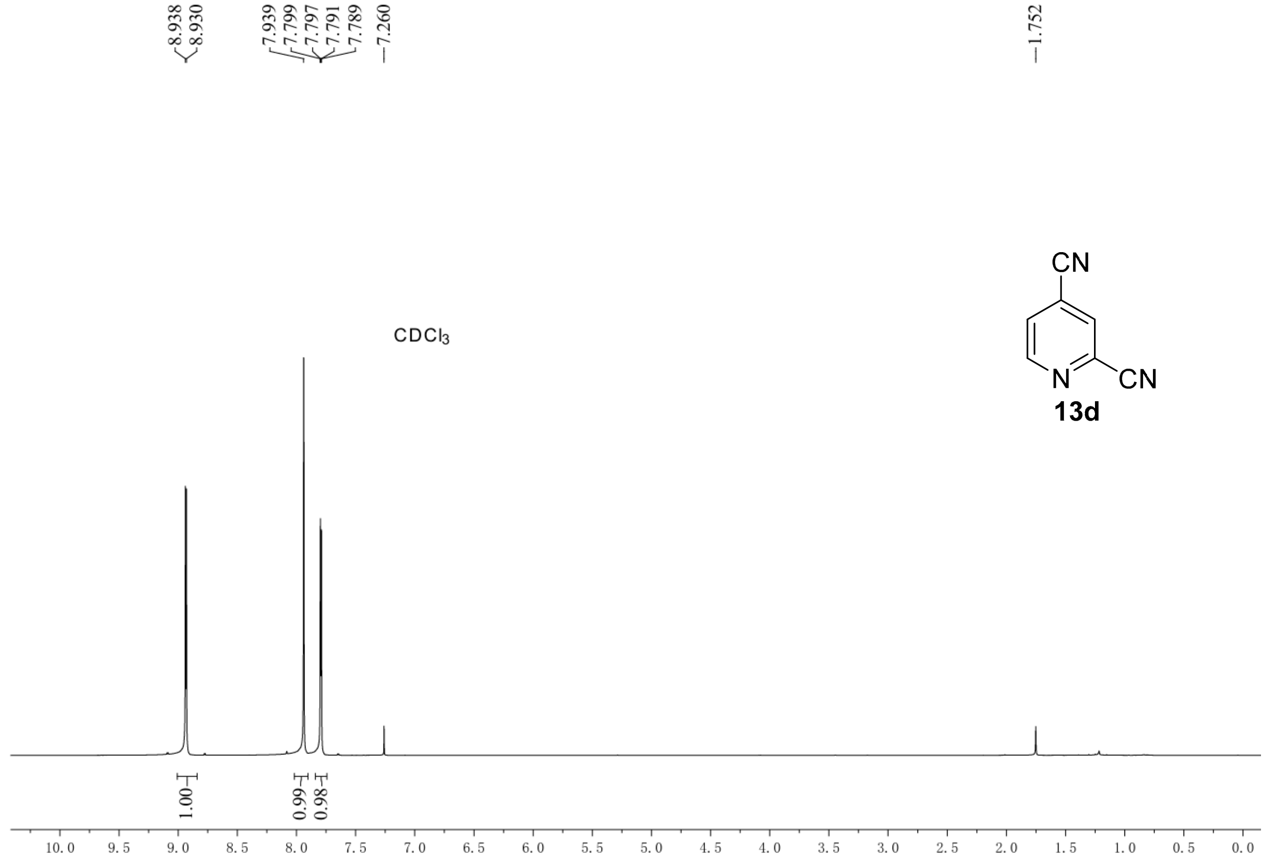

^{1}H NMR (600 MHz, CDCl$_3$):

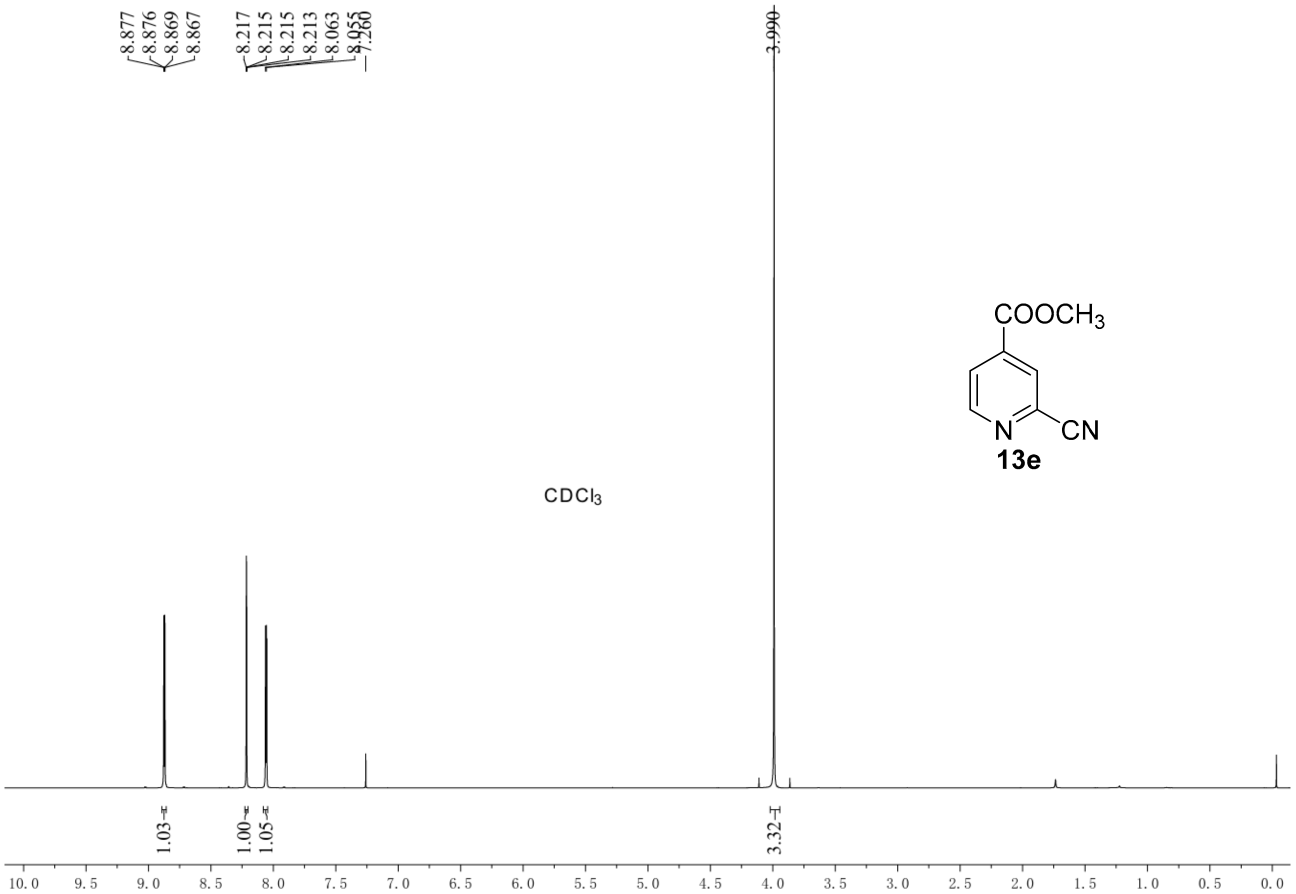

^{1}H NMR (600 MHz, $CDCl_3$):

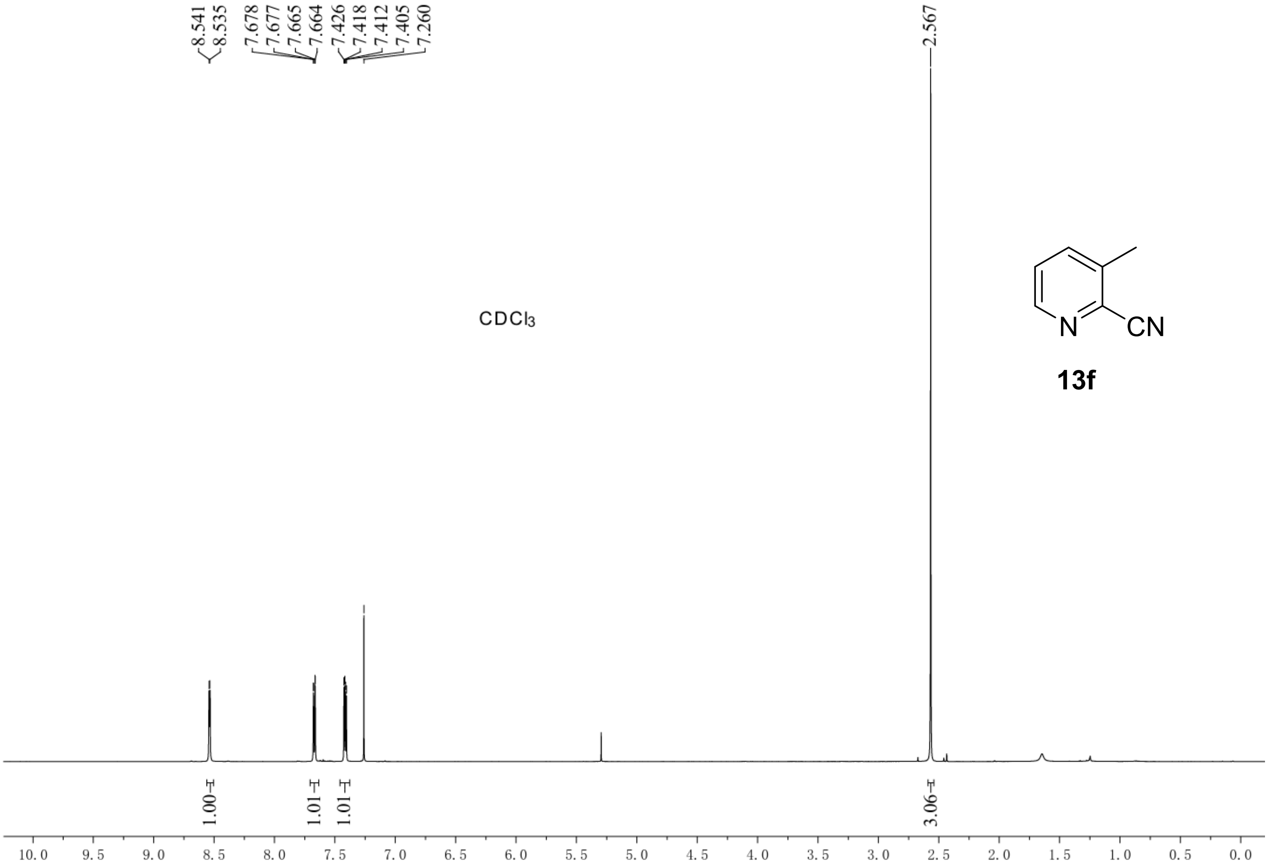

^{1}H NMR (600 MHz, $CDCl_3$):

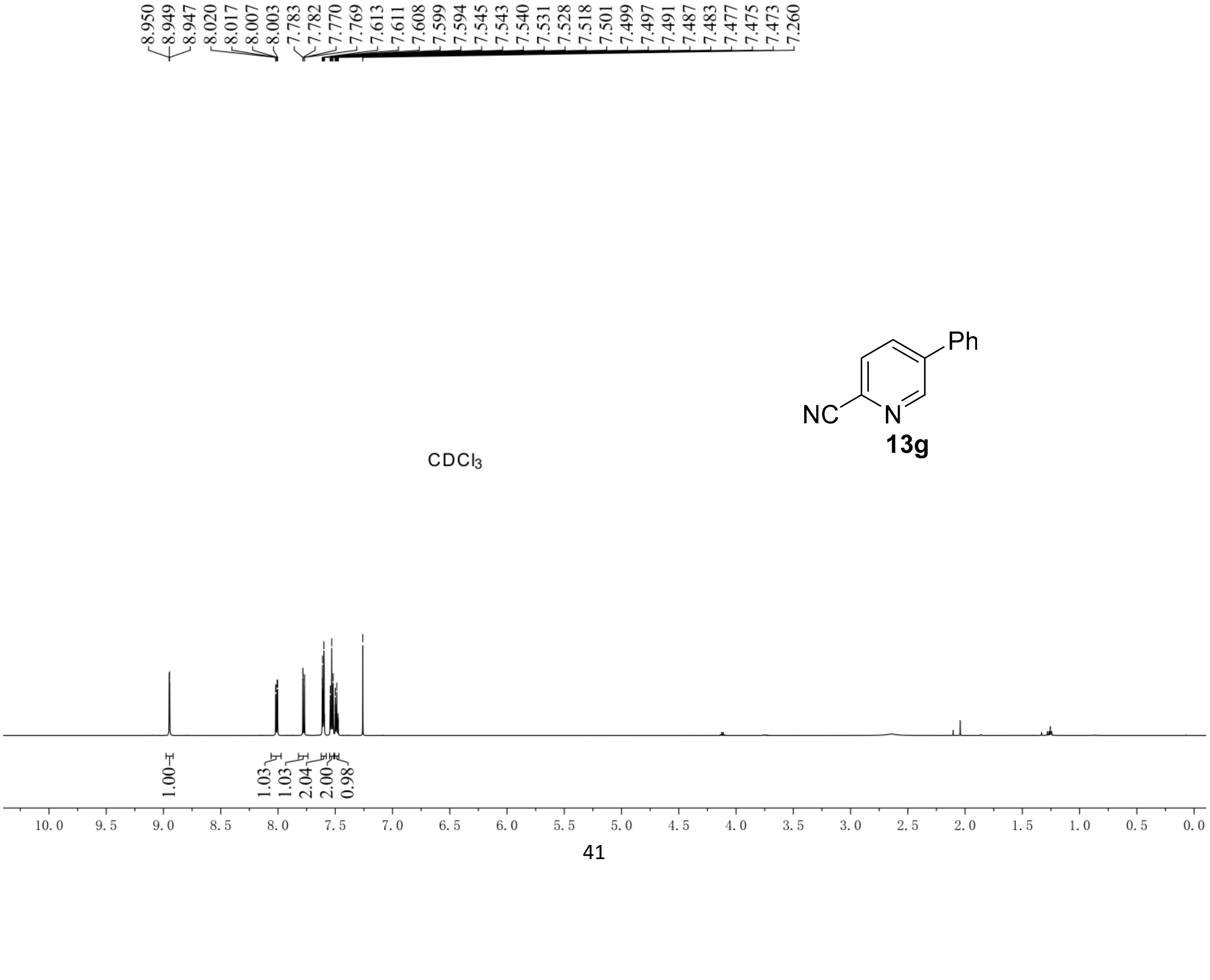

^{1}H NMR (600 MHz, $CDCl_3$):

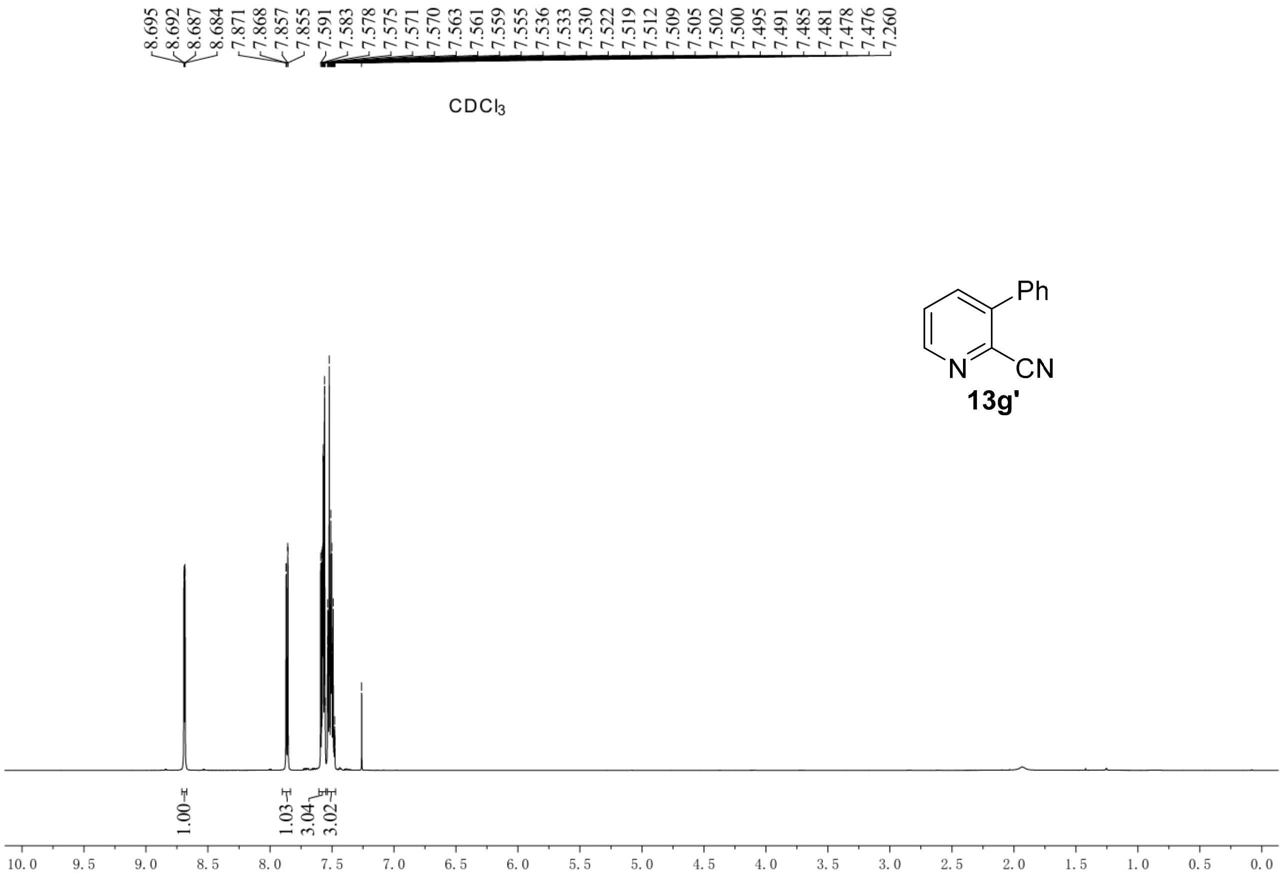

^{1}H NMR (600 MHz, $CDCl_3$):

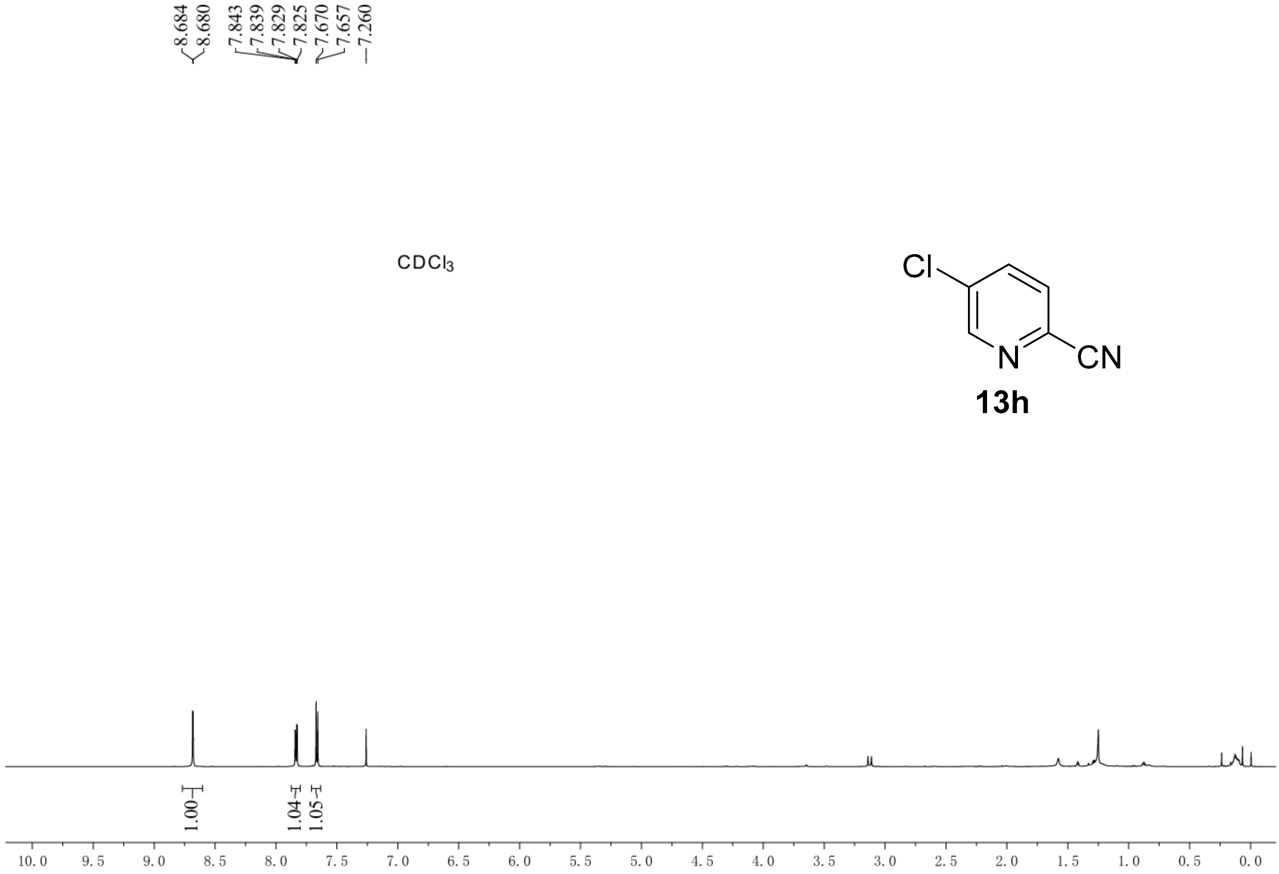

^{1}H NMR (600 MHz, $CDCl_3$):

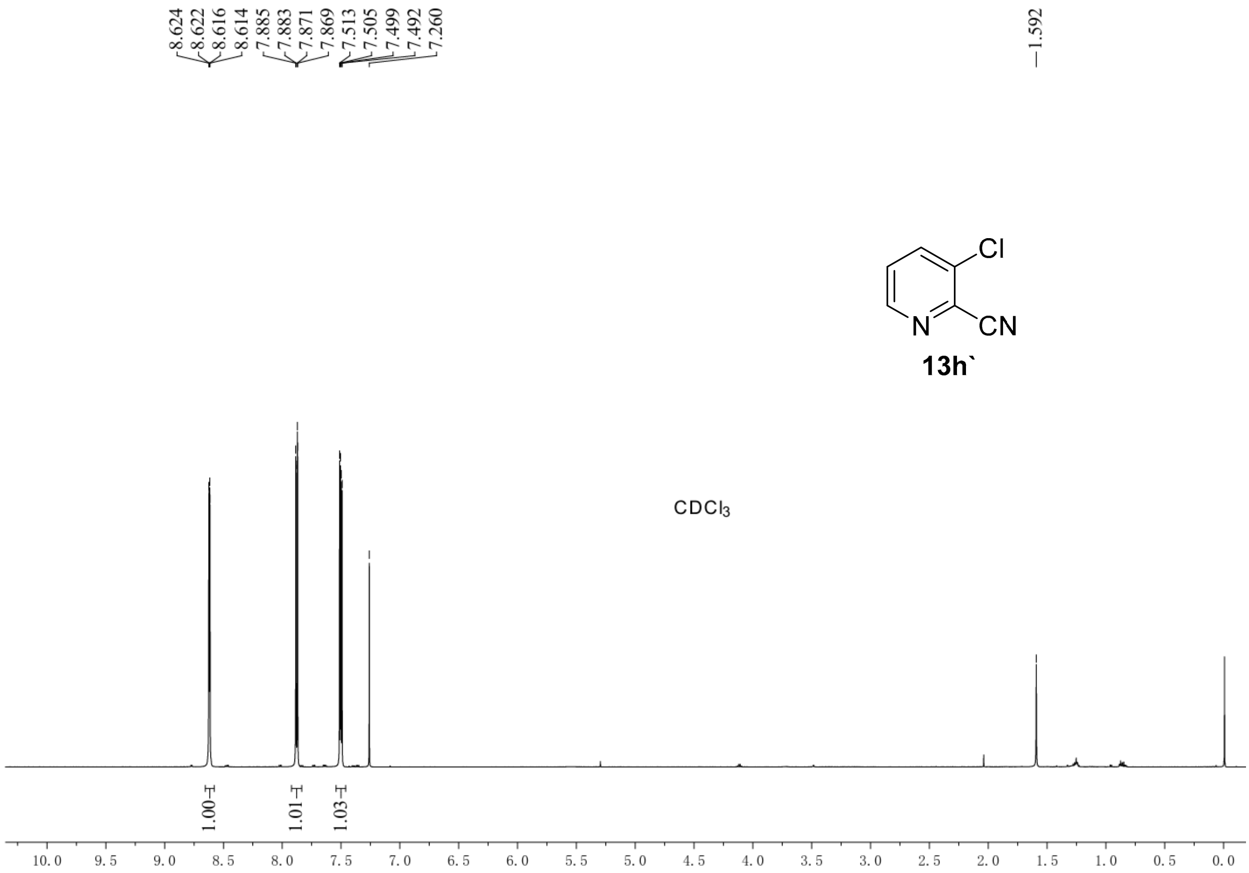

^{1}H NMR (600 MHz, $CDCl_3$):

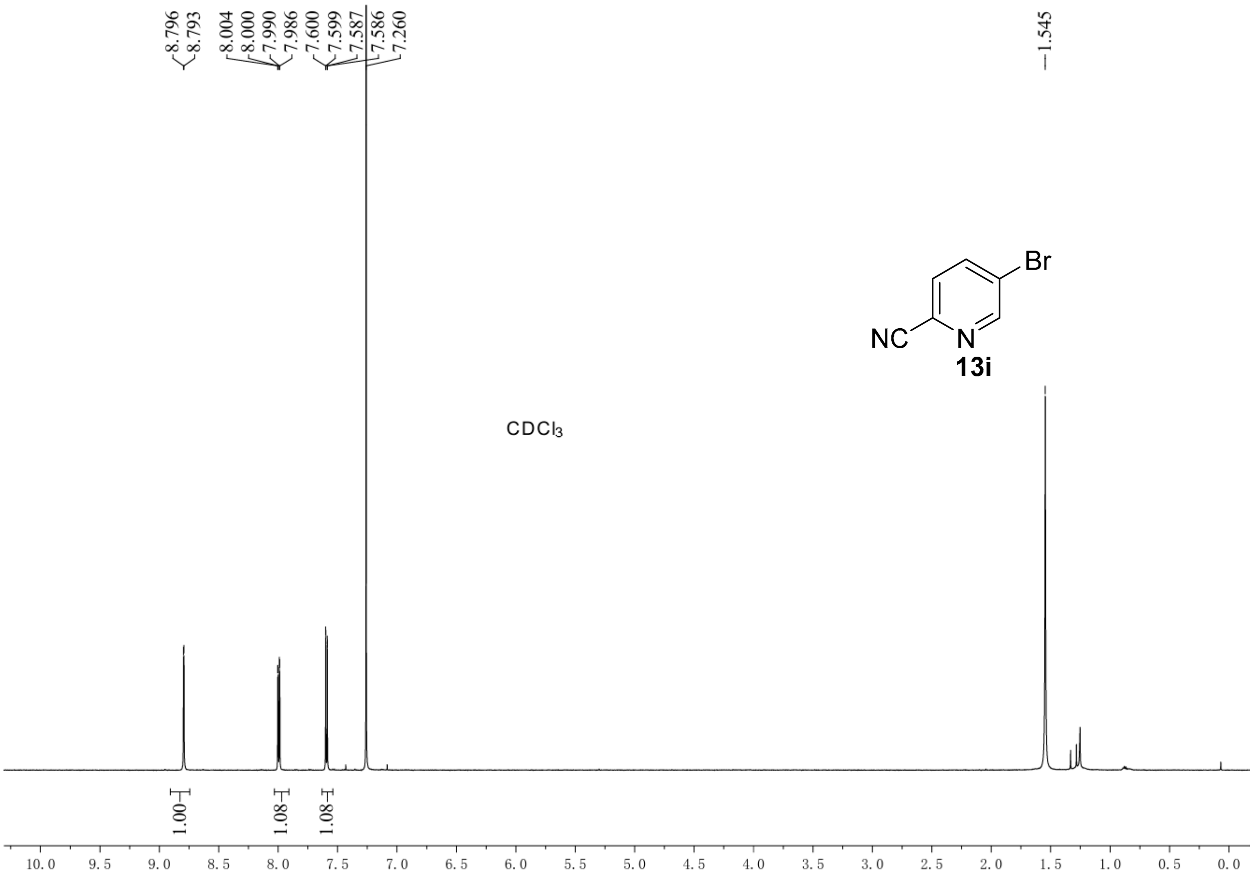

^{1}H NMR (600 MHz, $CDCl_3$):

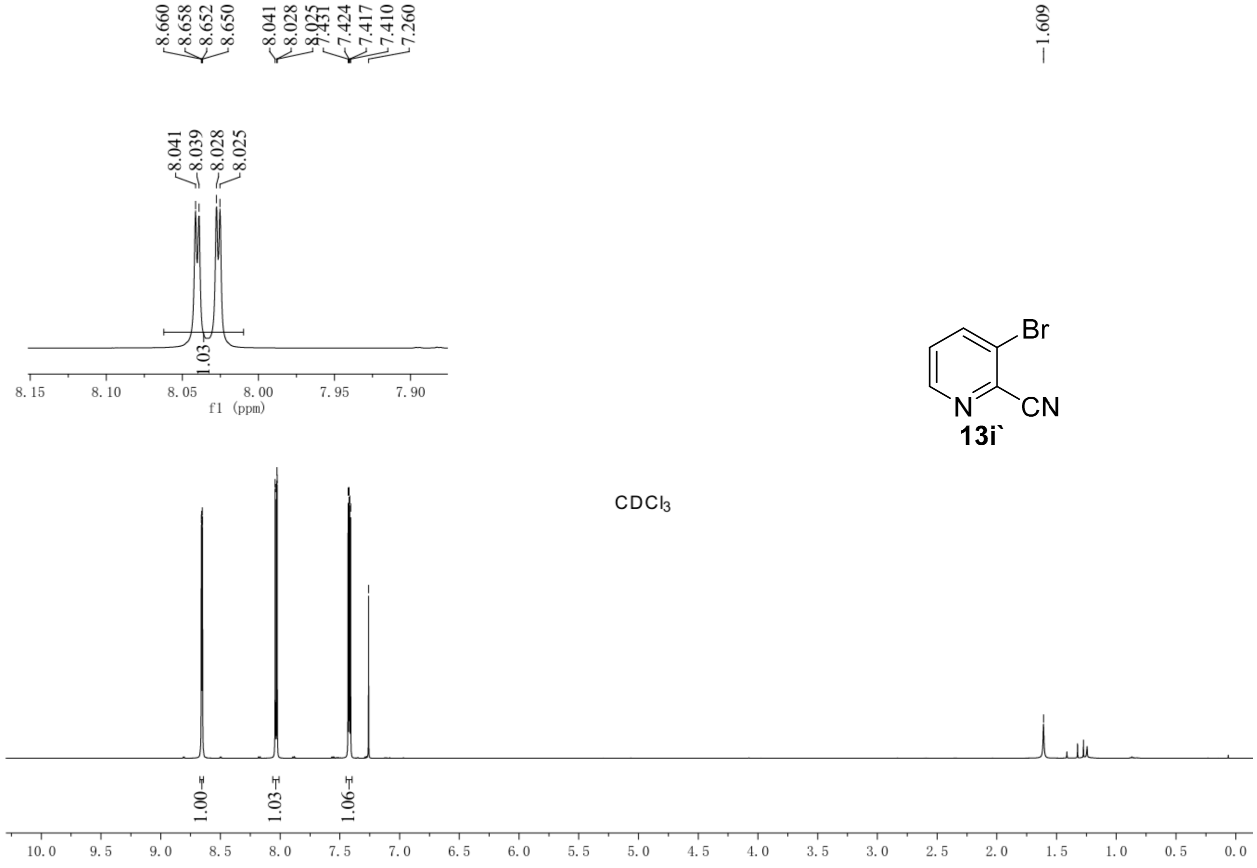

^{1}H NMR (600 MHz, $CDCl_3$):

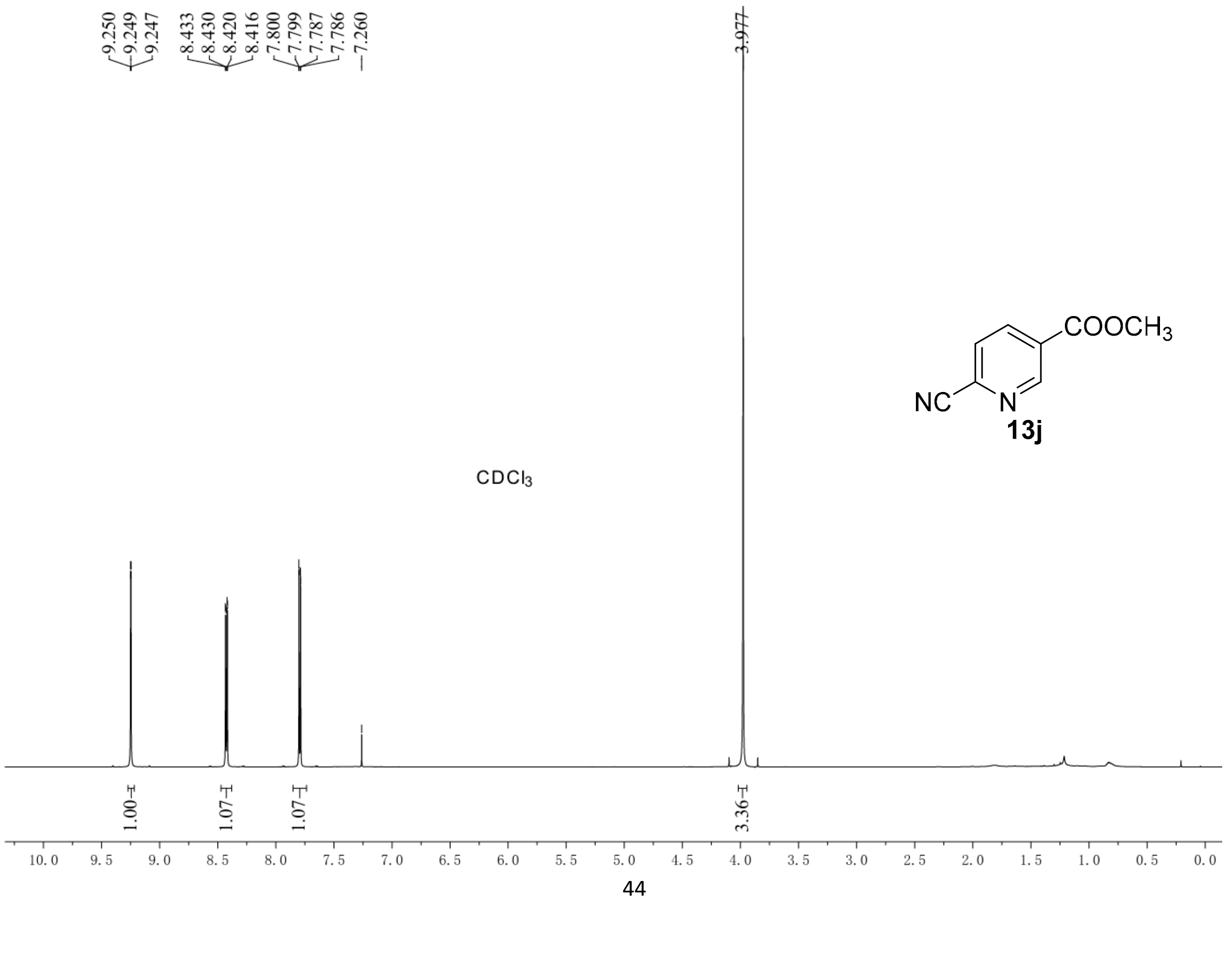

^{1}H NMR (600 MHz, $CDCl_3$):

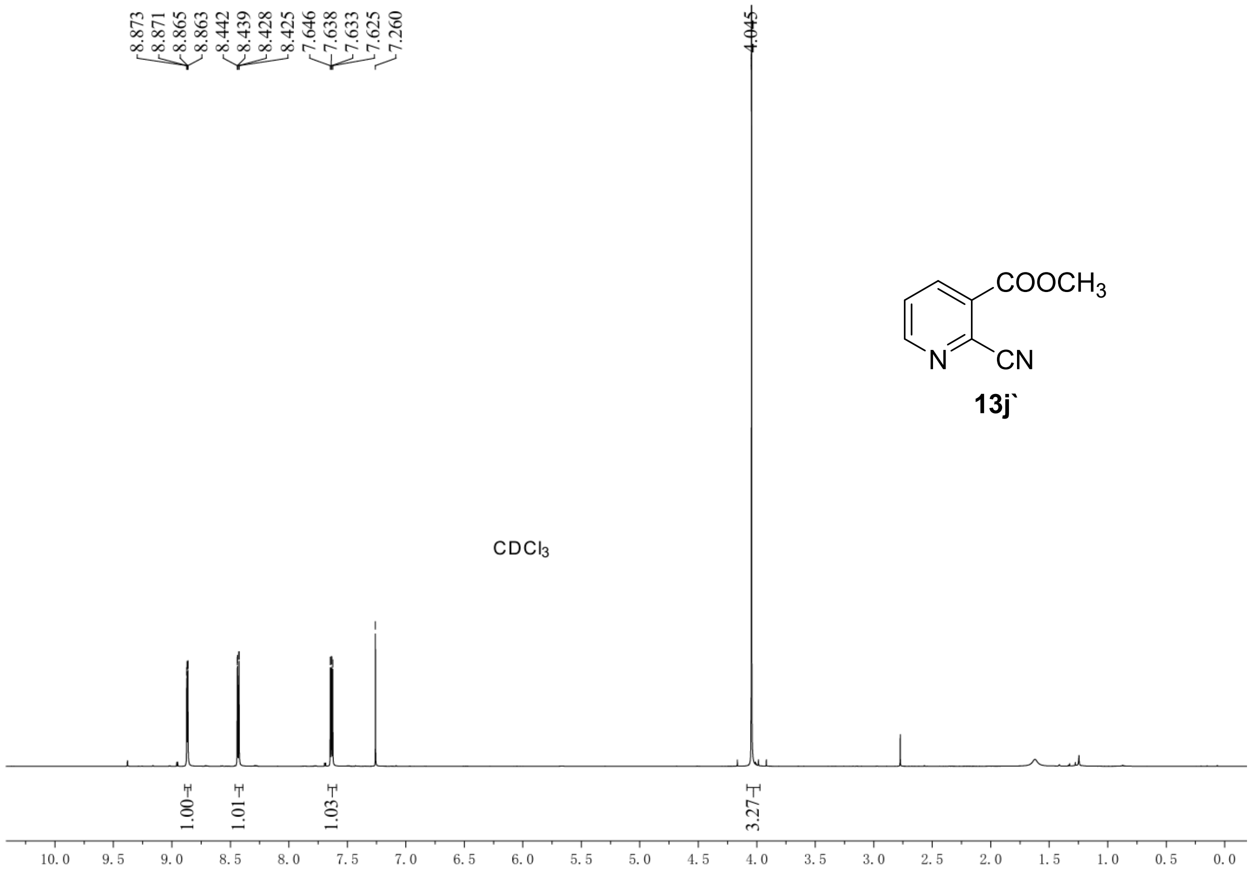

^{1}H NMR (600 MHz, $CDCl_3$):

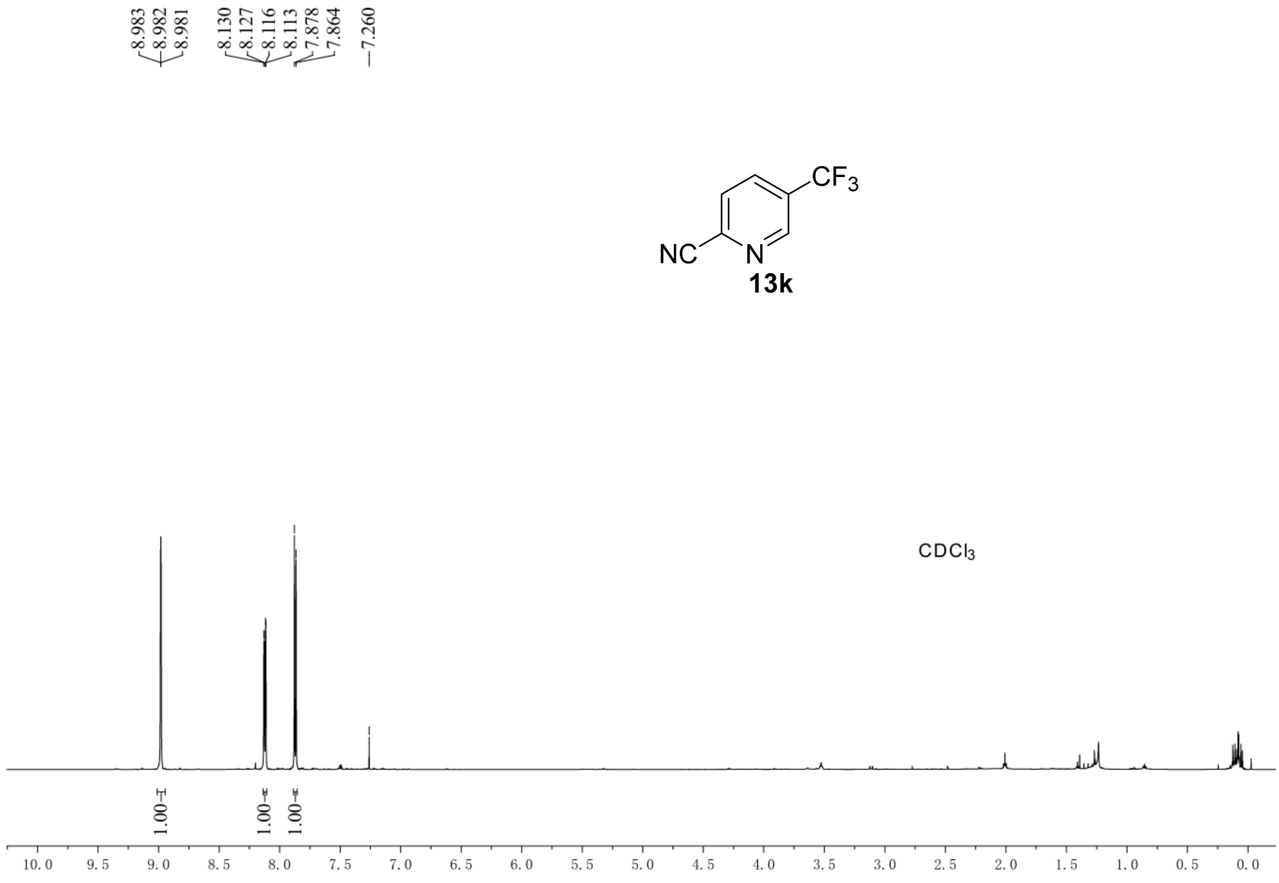

^{1}H NMR (600 MHz, $CDCl_3$):

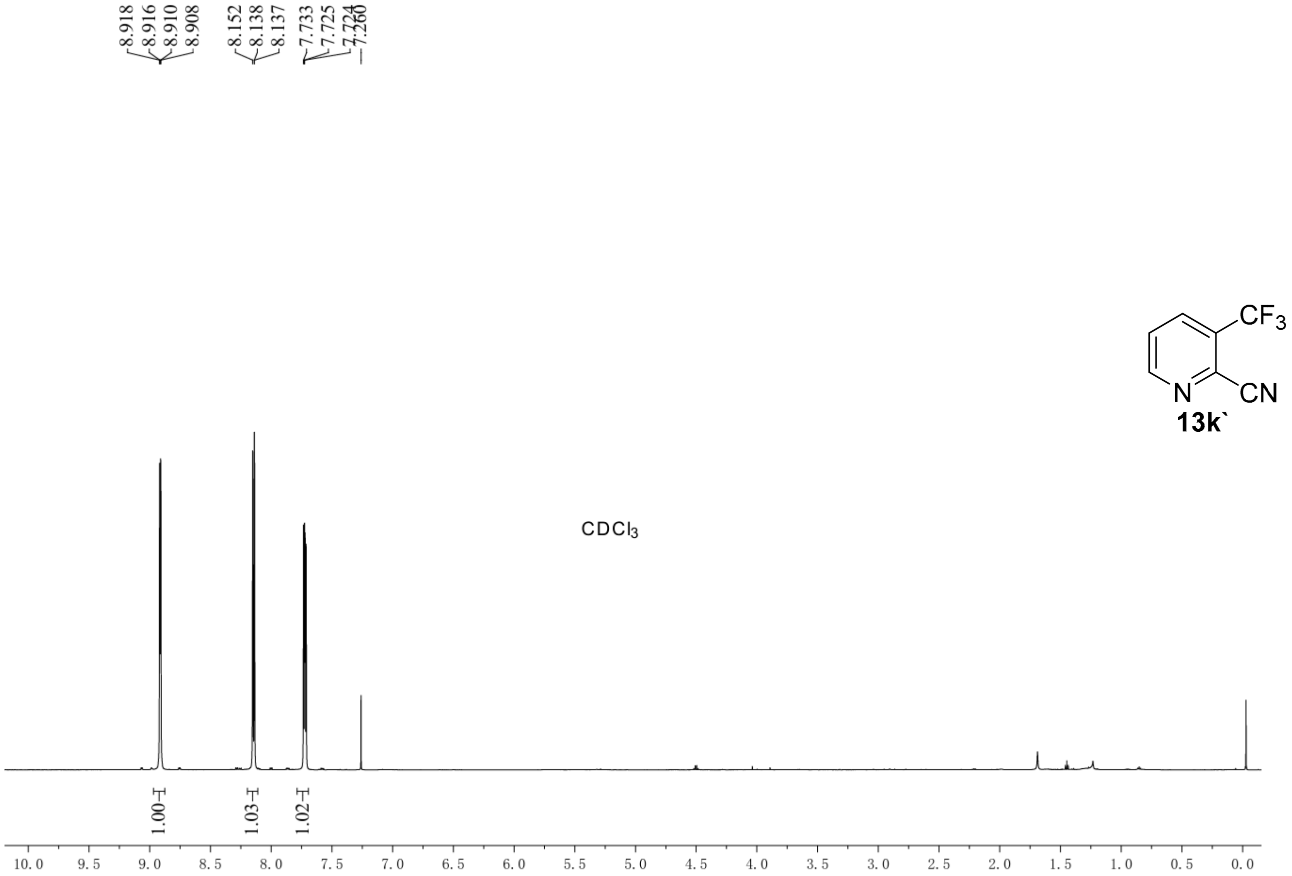

^{1}H NMR (600 MHz, $CDCl_3$):

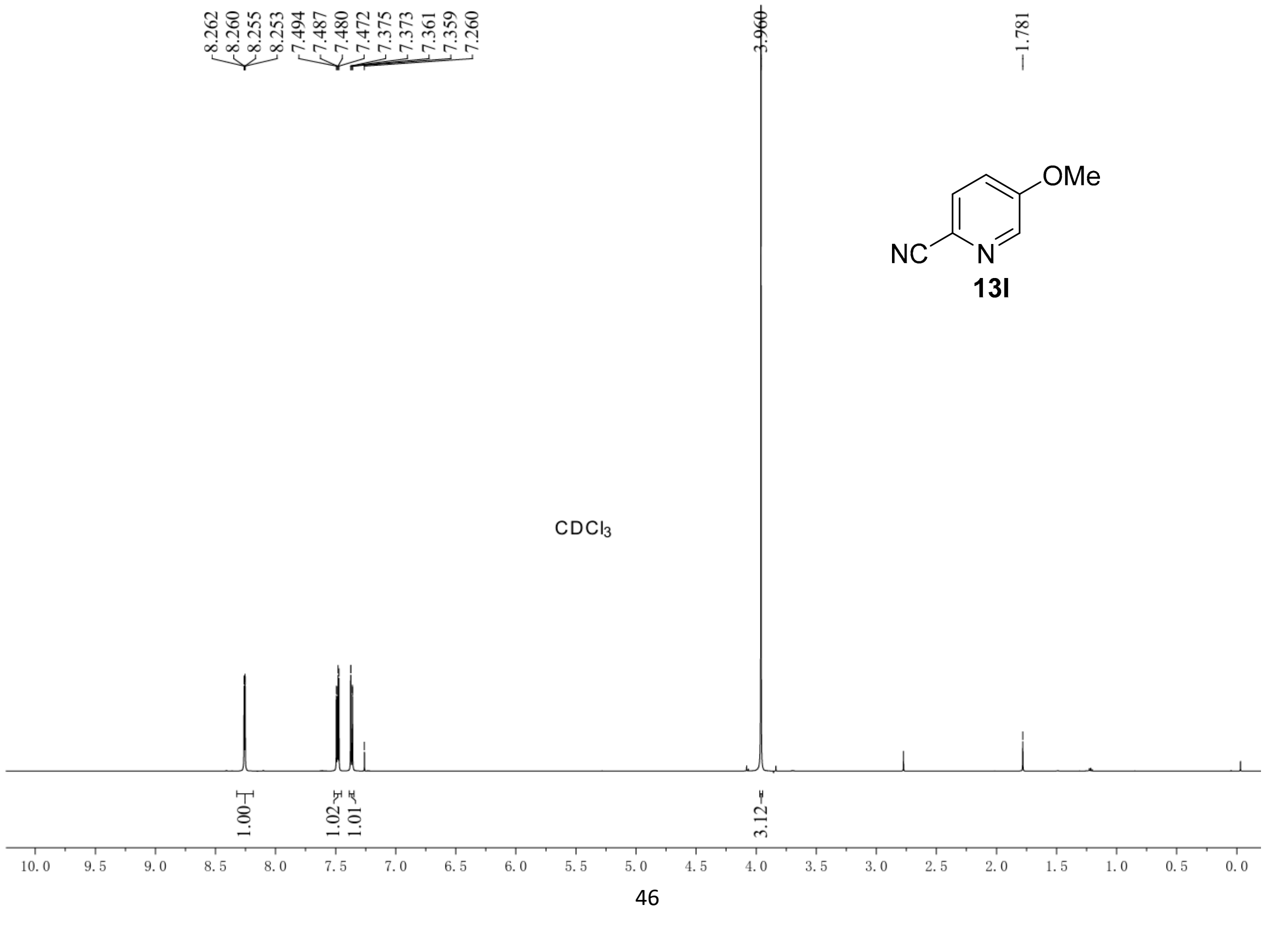

^{1}H NMR (600 MHz, CDCl$_3$):

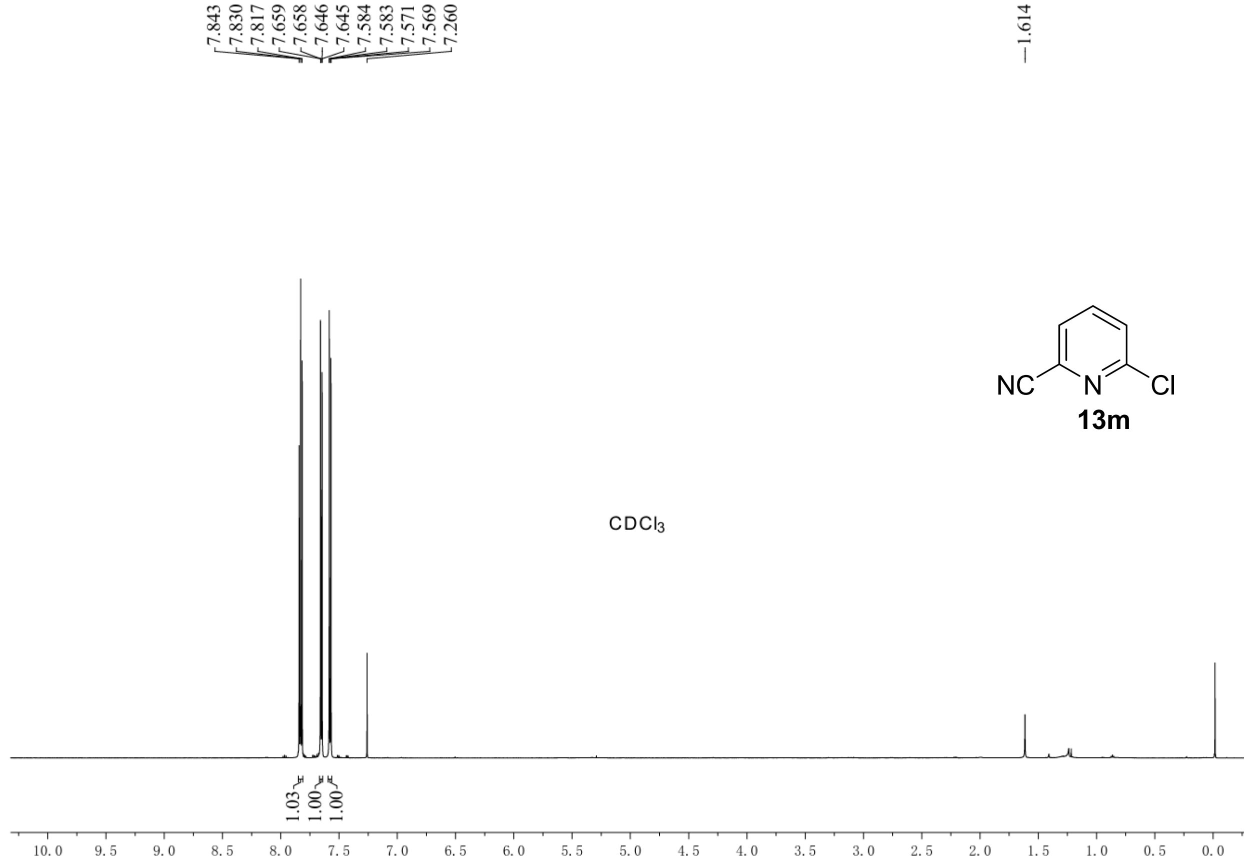

^{1}H NMR (600 MHz, CDCl$_3$):

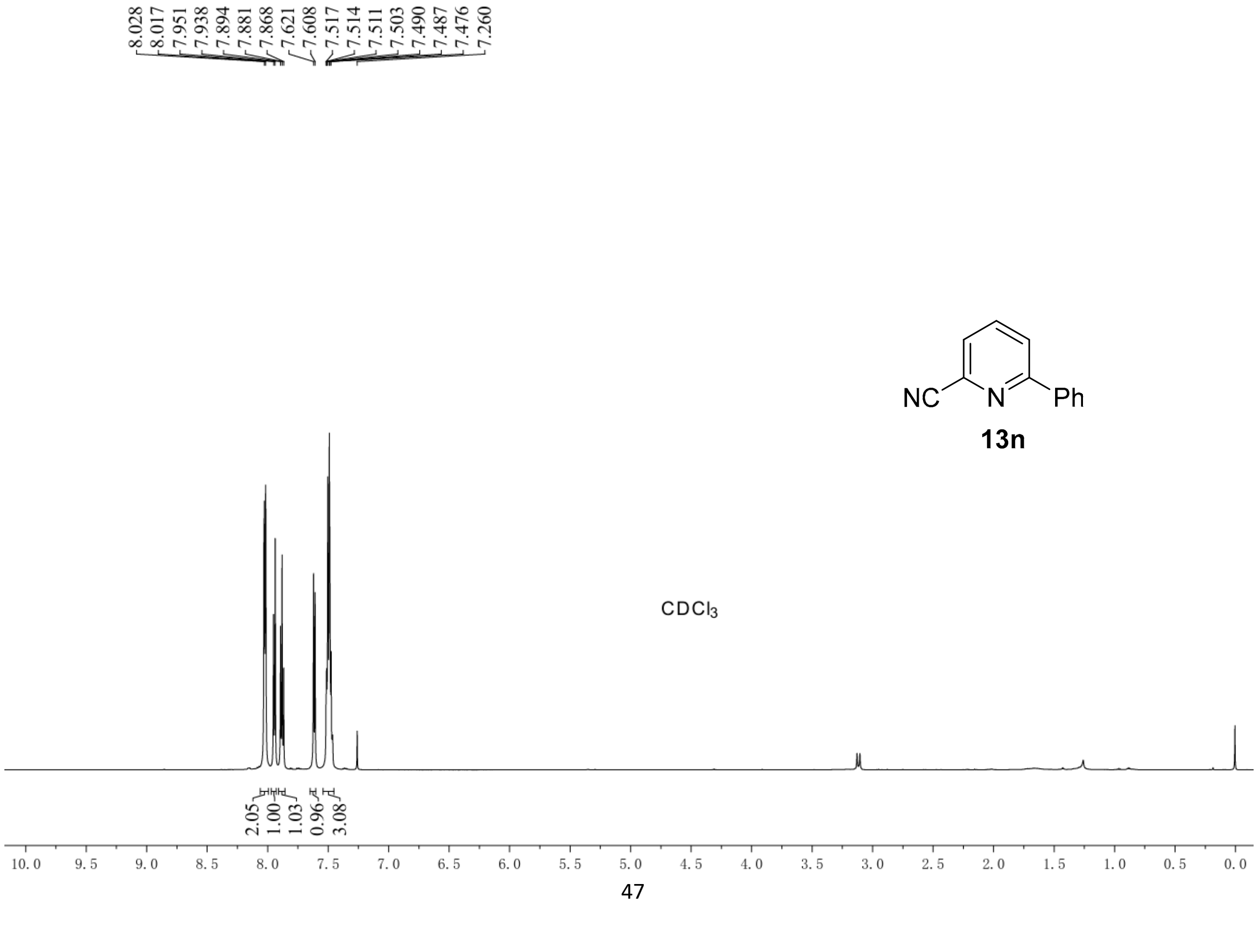

^{1}H NMR (600 MHz, $CDCl_3$):

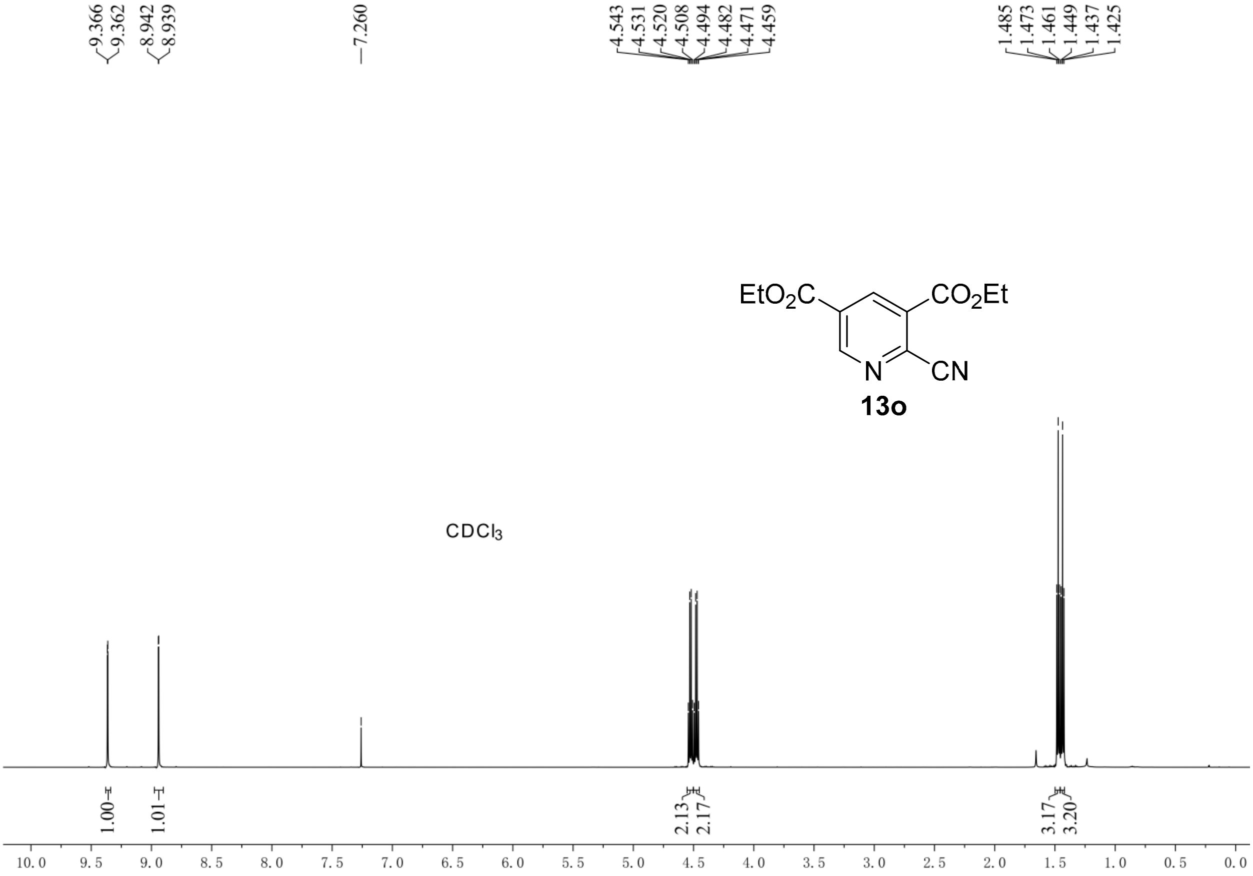

^{13}C NMR (150 MHz, $CDCl_3$):

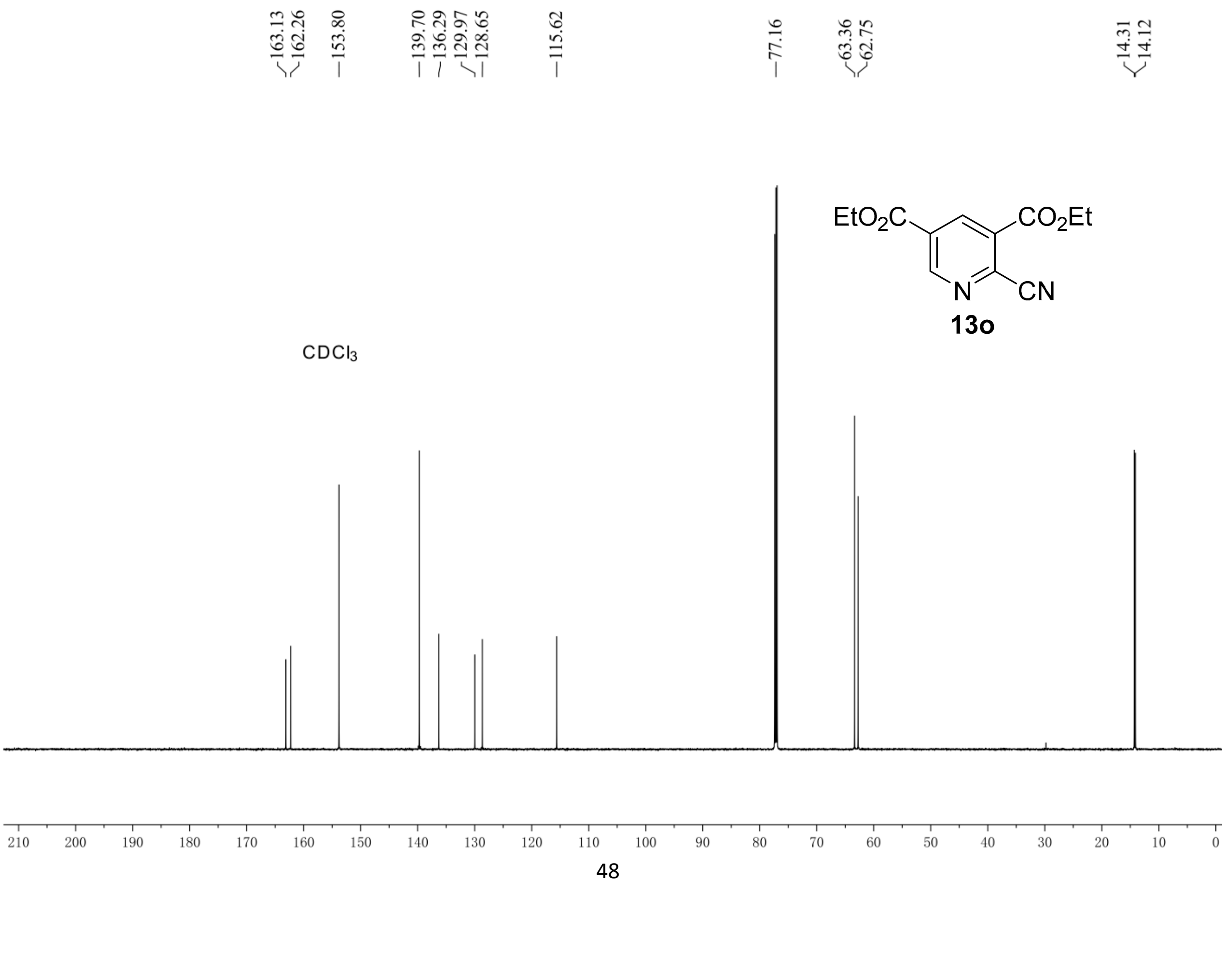

^{1}H NMR (600 MHz, $CDCl_3$):

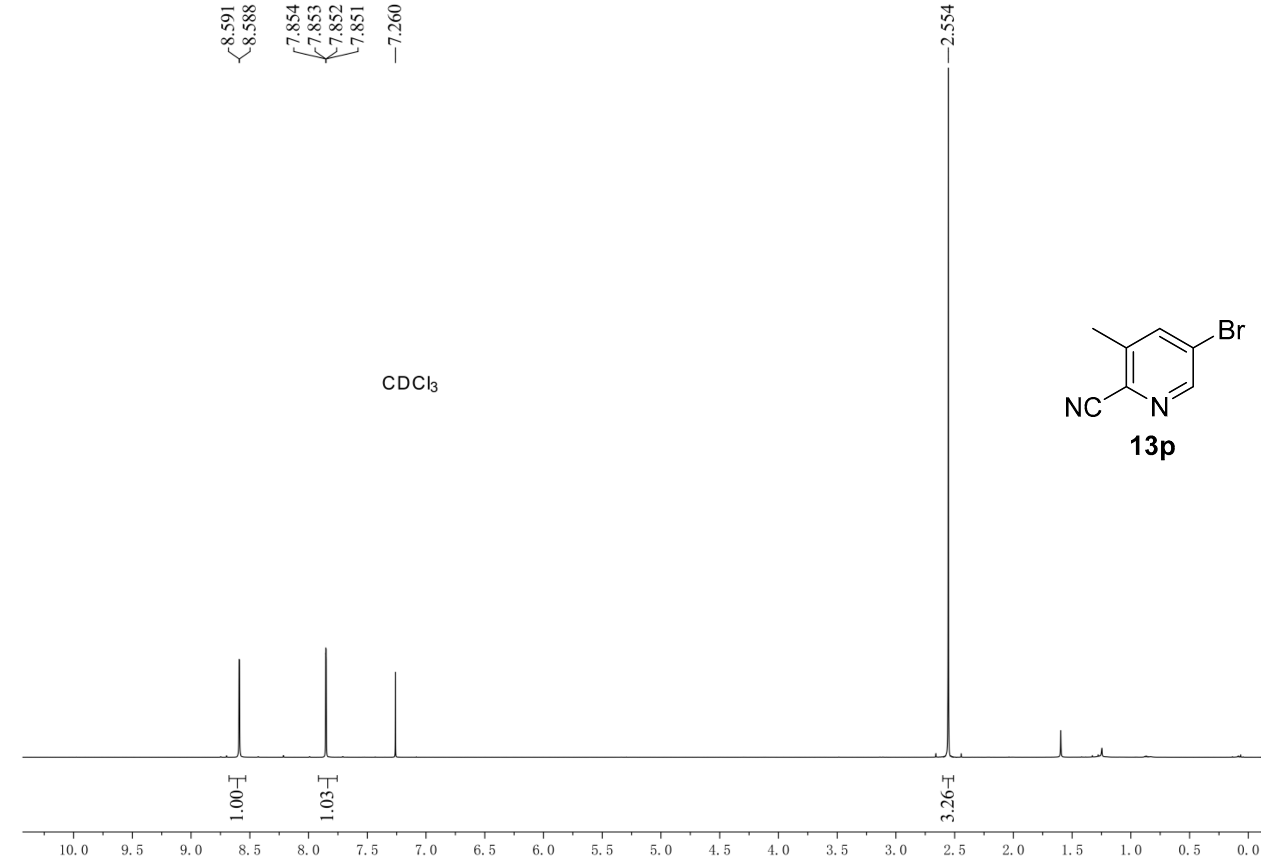

^{1}H NMR (600 MHz, $CDCl_3$):

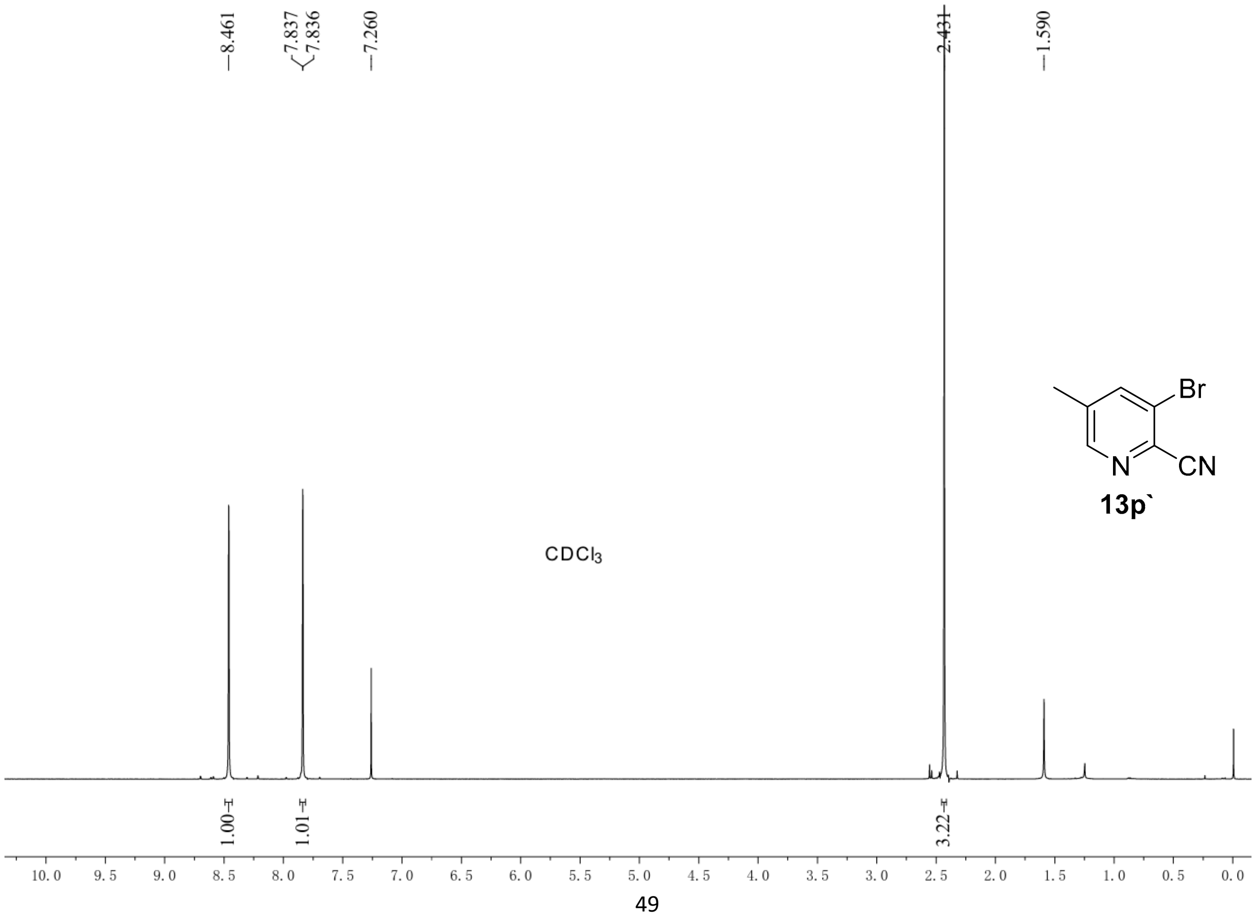

^{1}H NMR (600 MHz, $CDCl_3$):

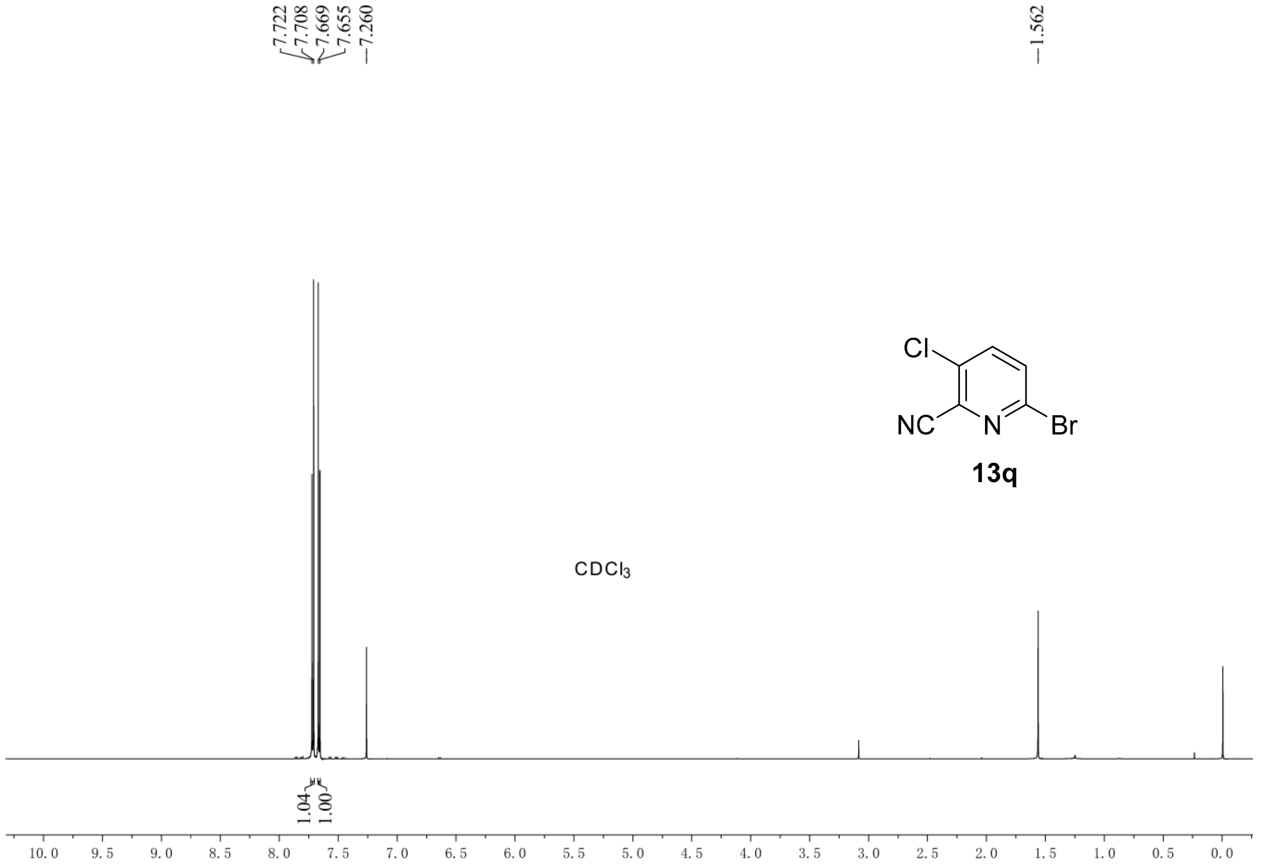

^{13}C NMR (150 MHz, $CDCl_3$):

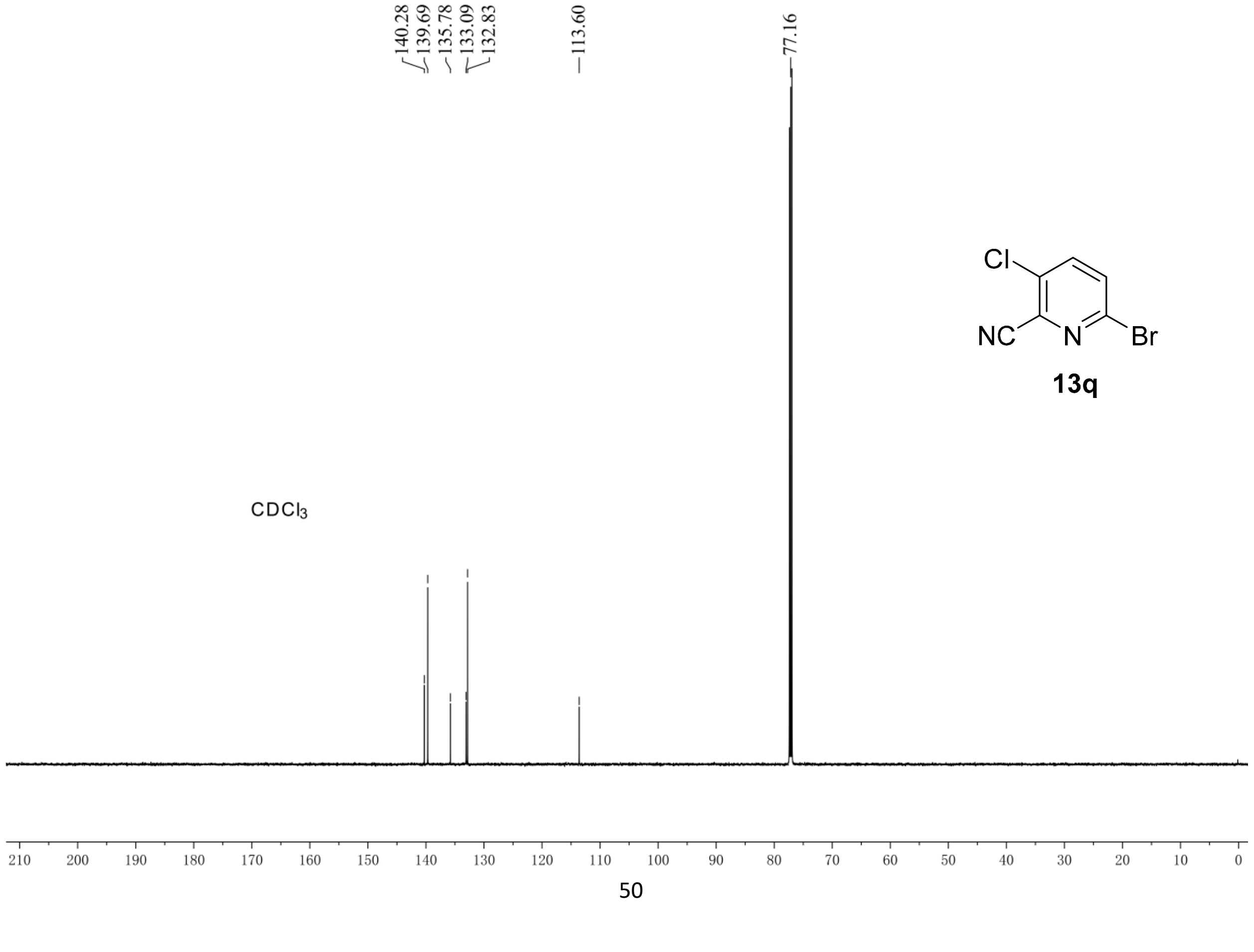

^{1}H NMR (600 MHz, CDCl$_3$):

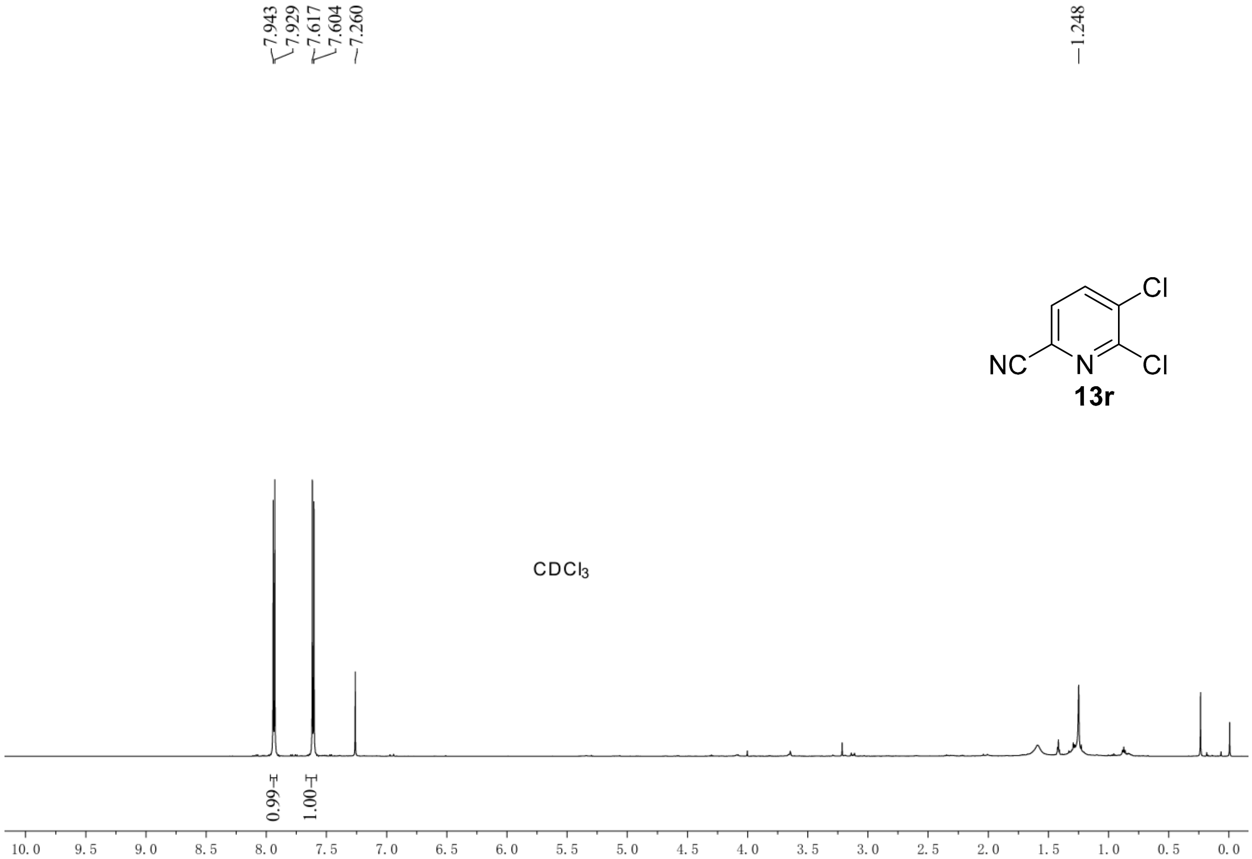

^{1}H NMR (600 MHz, CDCl$_3$):

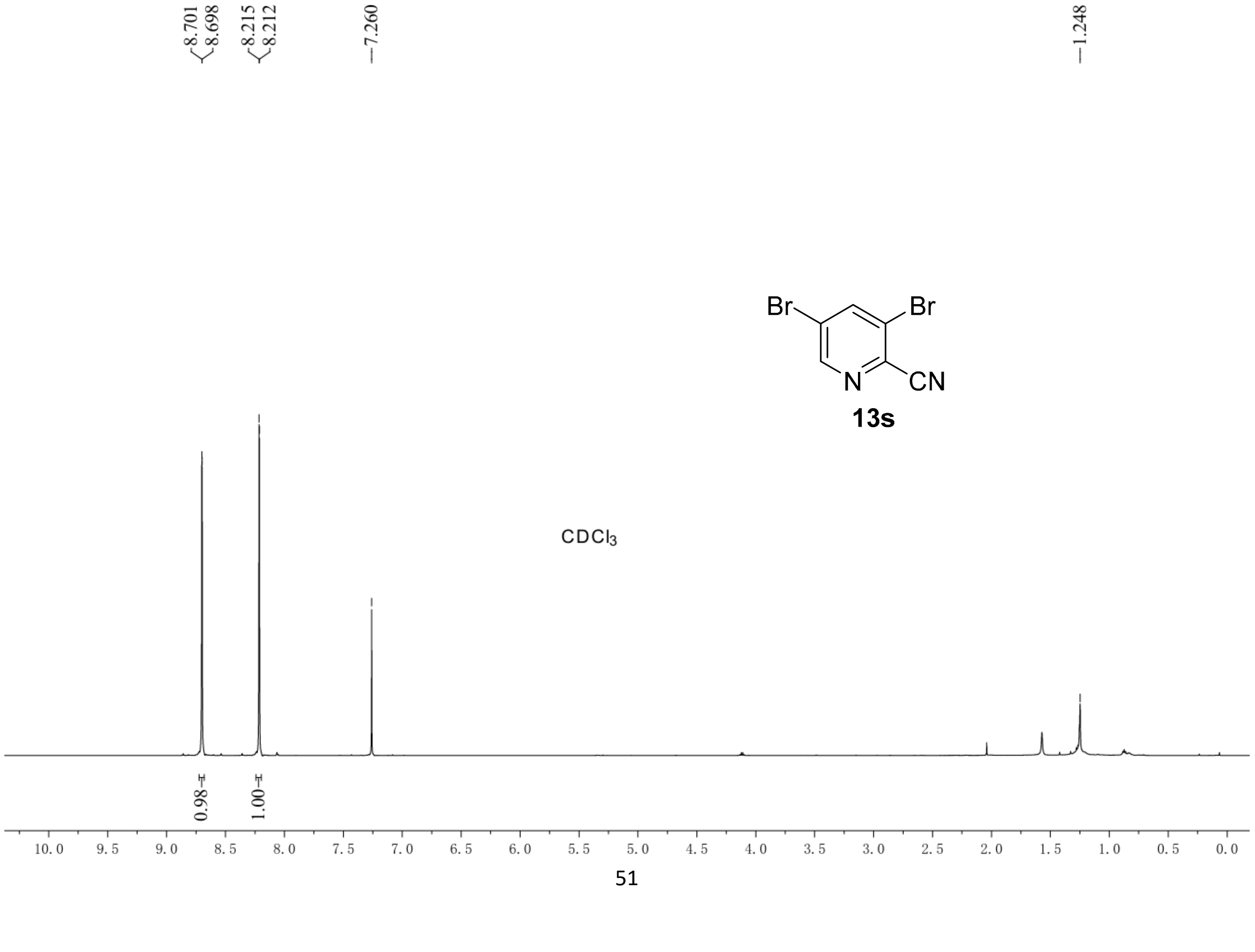

^{1}H NMR (600 MHz, $CDCl_3$):

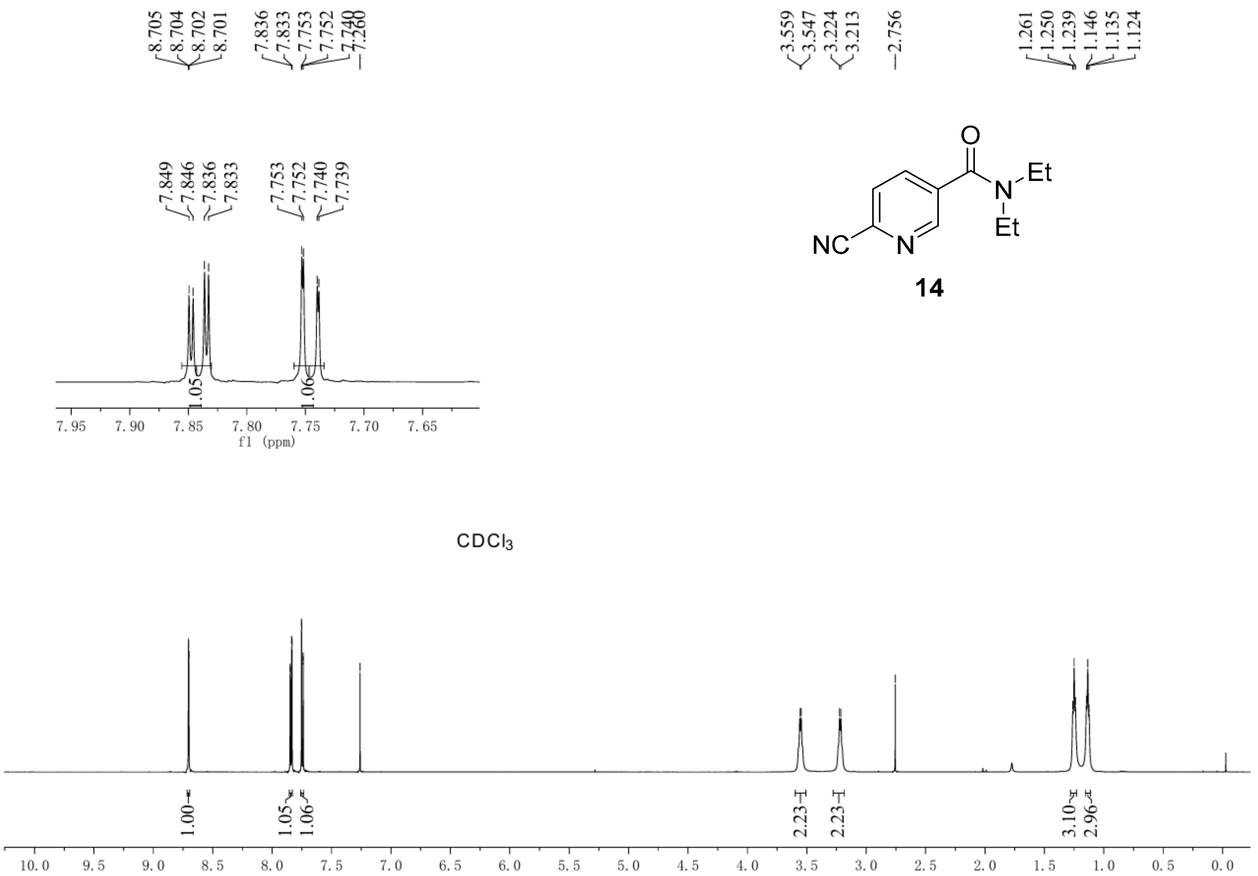

^{1}H NMR (600 MHz, $CDCl_3$):

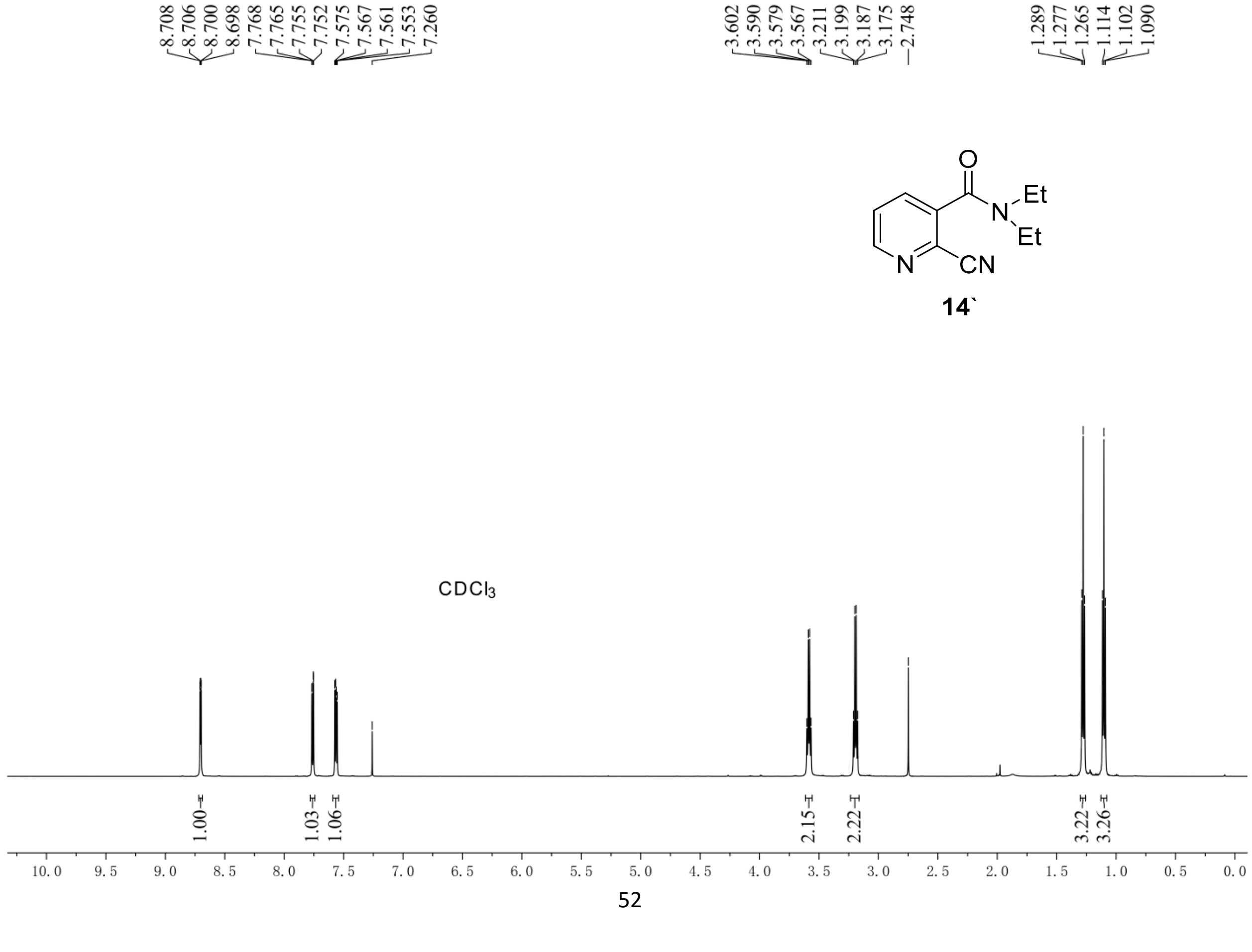

^{1}H NMR (600 MHz, CDCl$_3$):

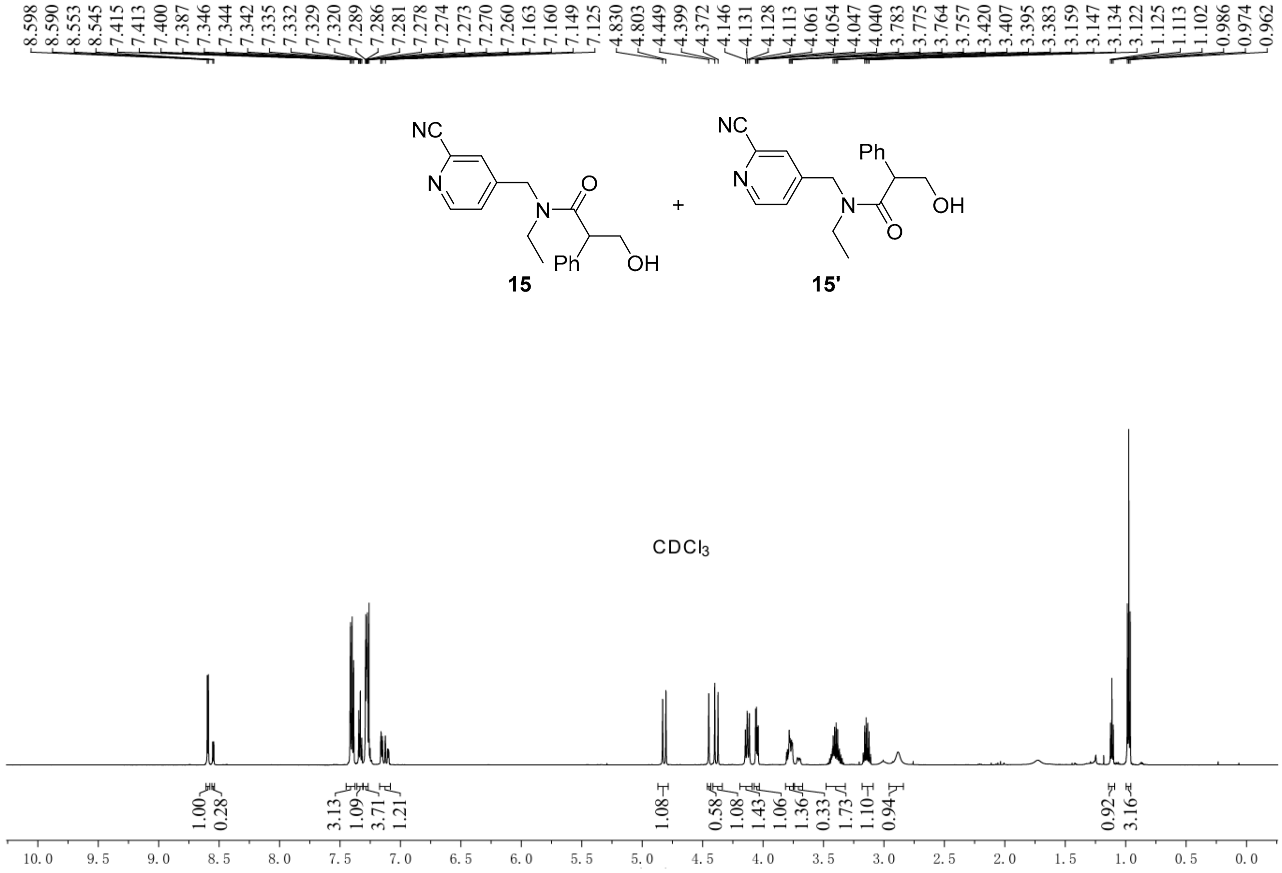

^{13}C NMR (100 MHz, CDCl$_3$):

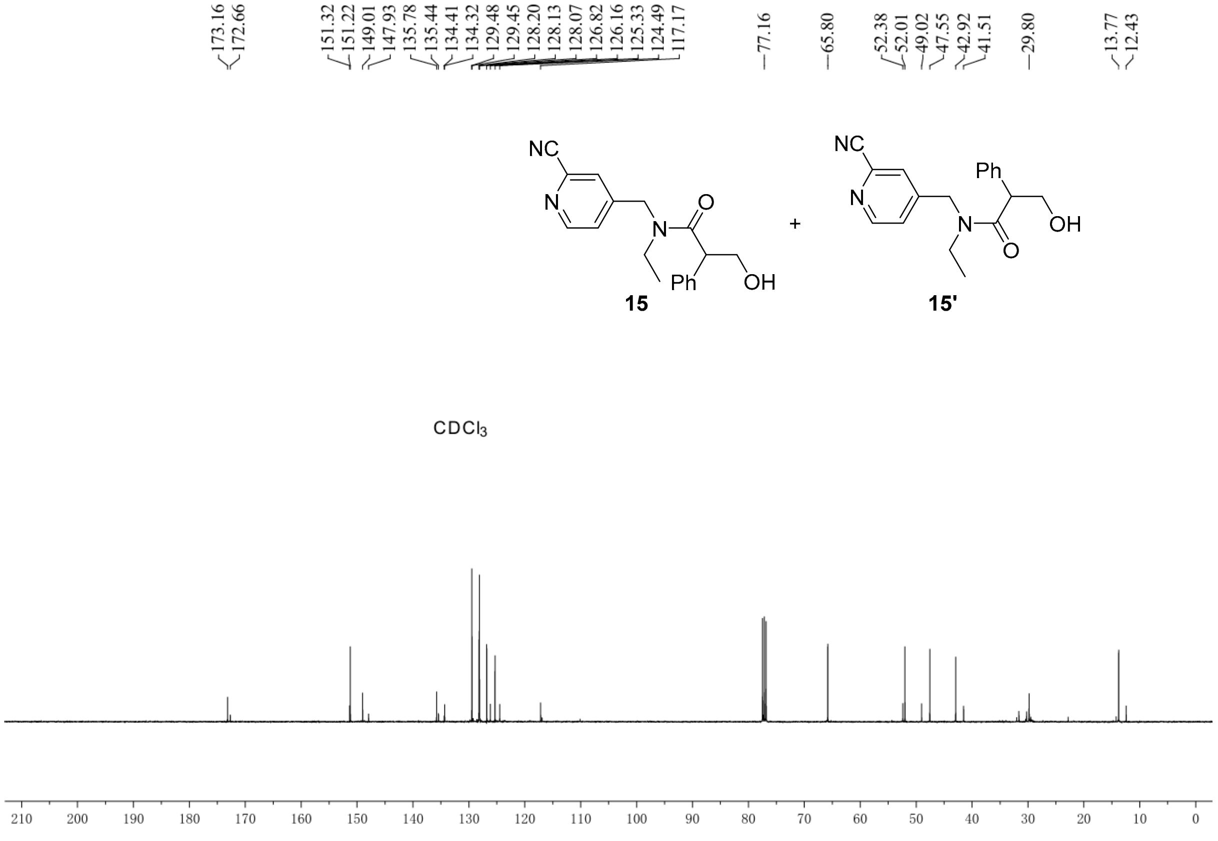

^{1}H NMR (600 MHz, $CDCl_3$):

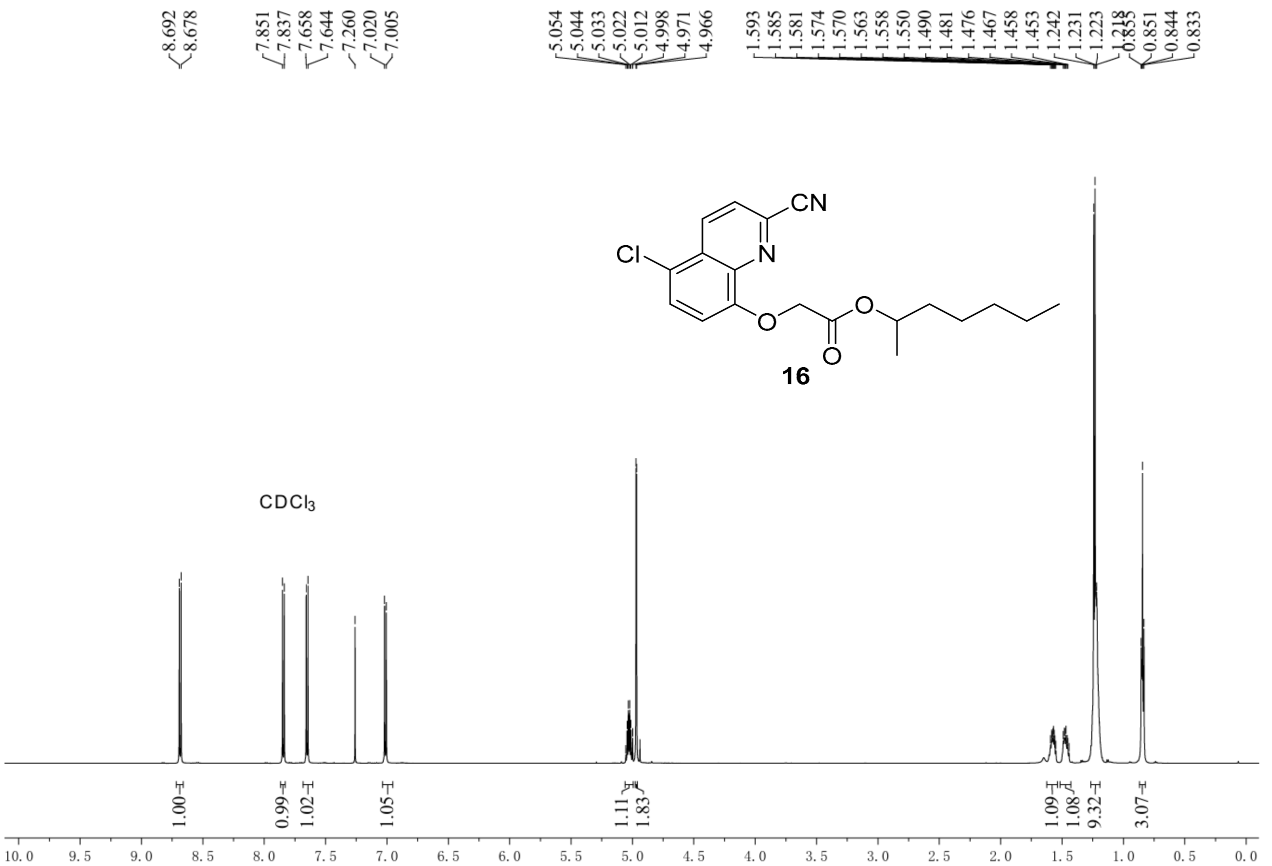

^{1}H NMR (600 MHz, $CDCl_3$):

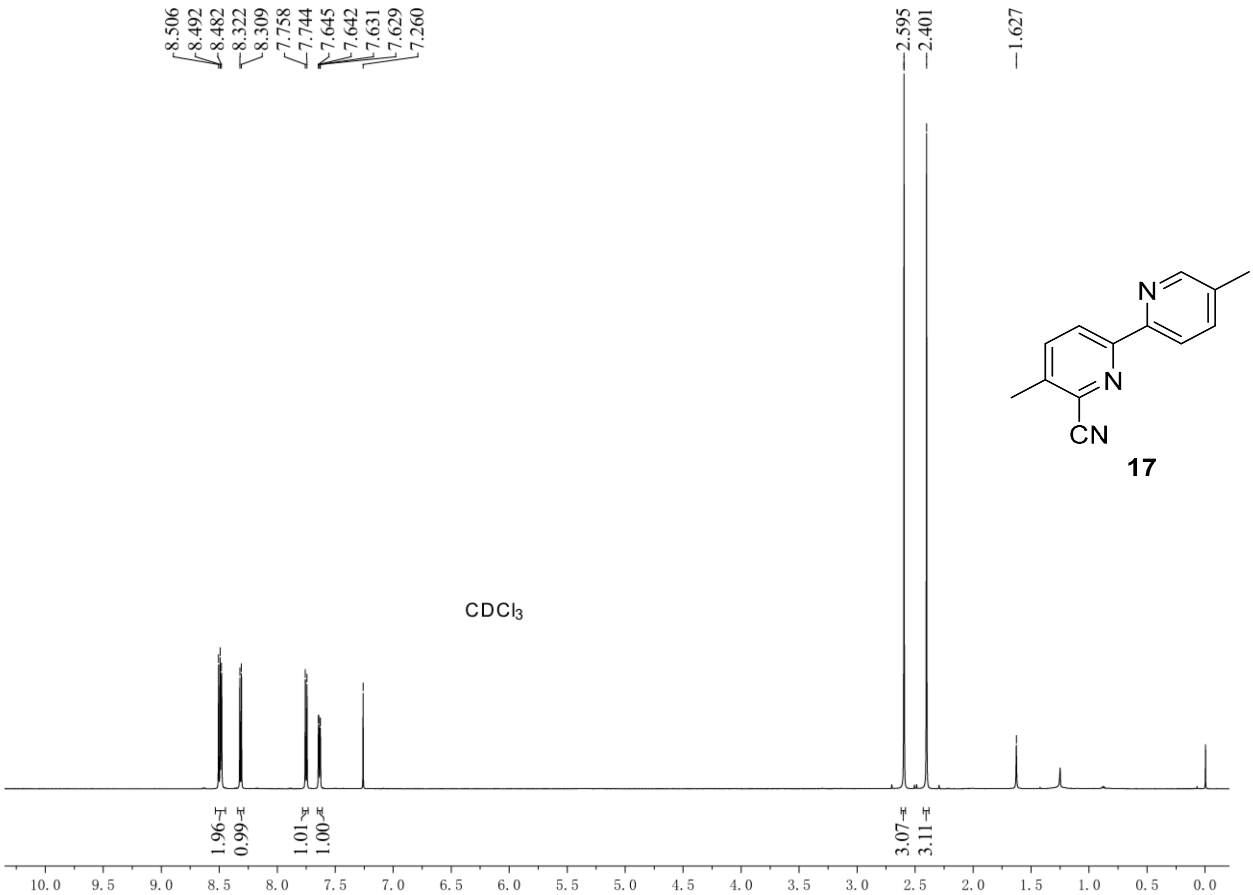

^{13}C NMR (150 MHz, $CDCl_3$):

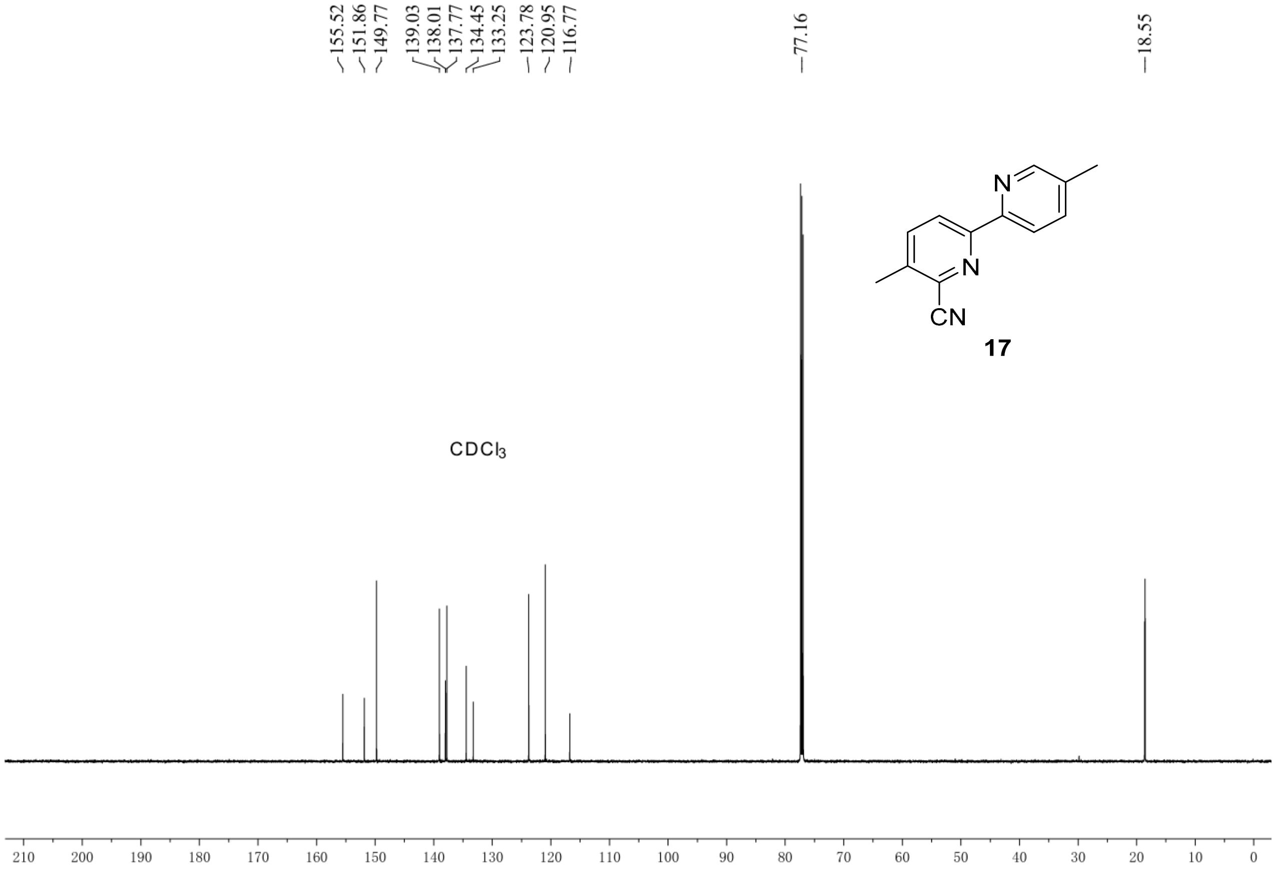

^{1}H NMR (600 MHz, $CDCl_3$):

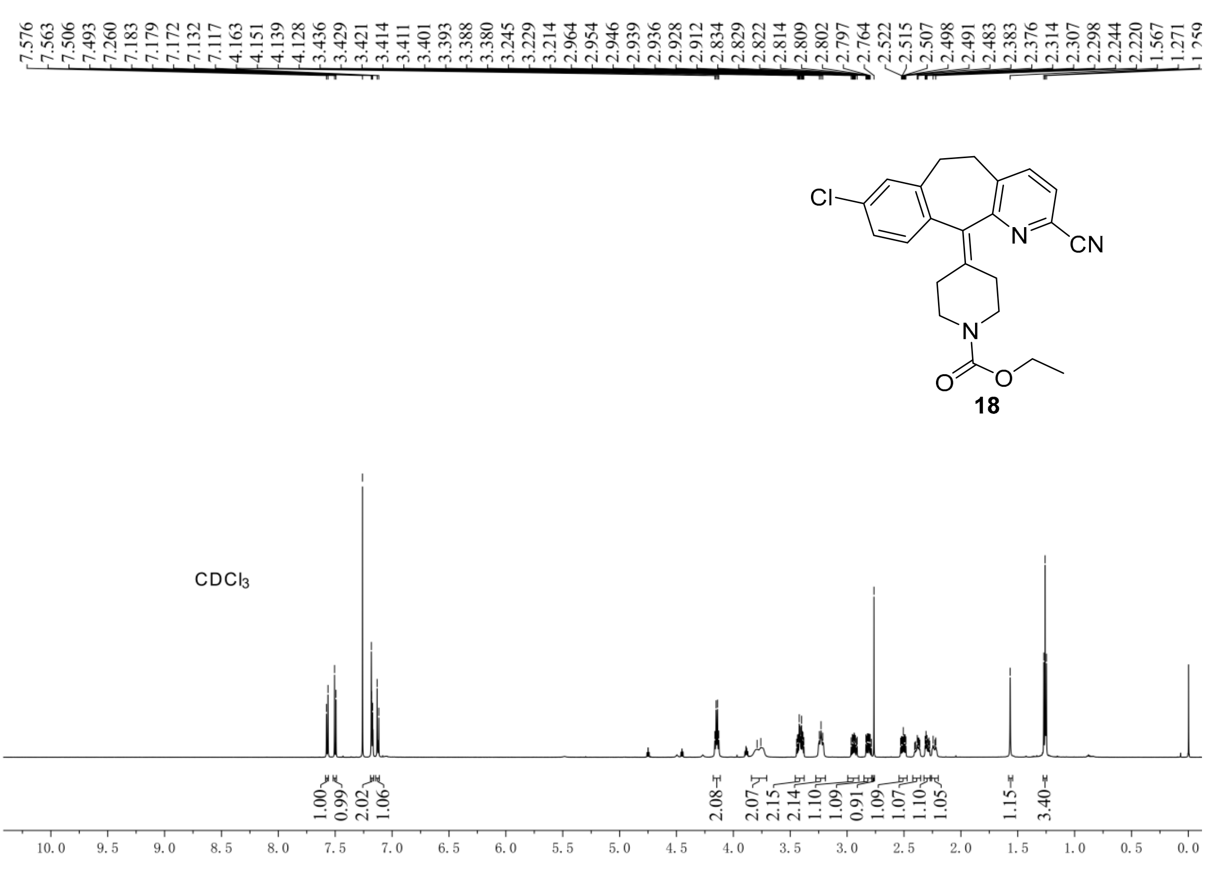

^{1}H NMR (600 MHz, CDCl$_3$):

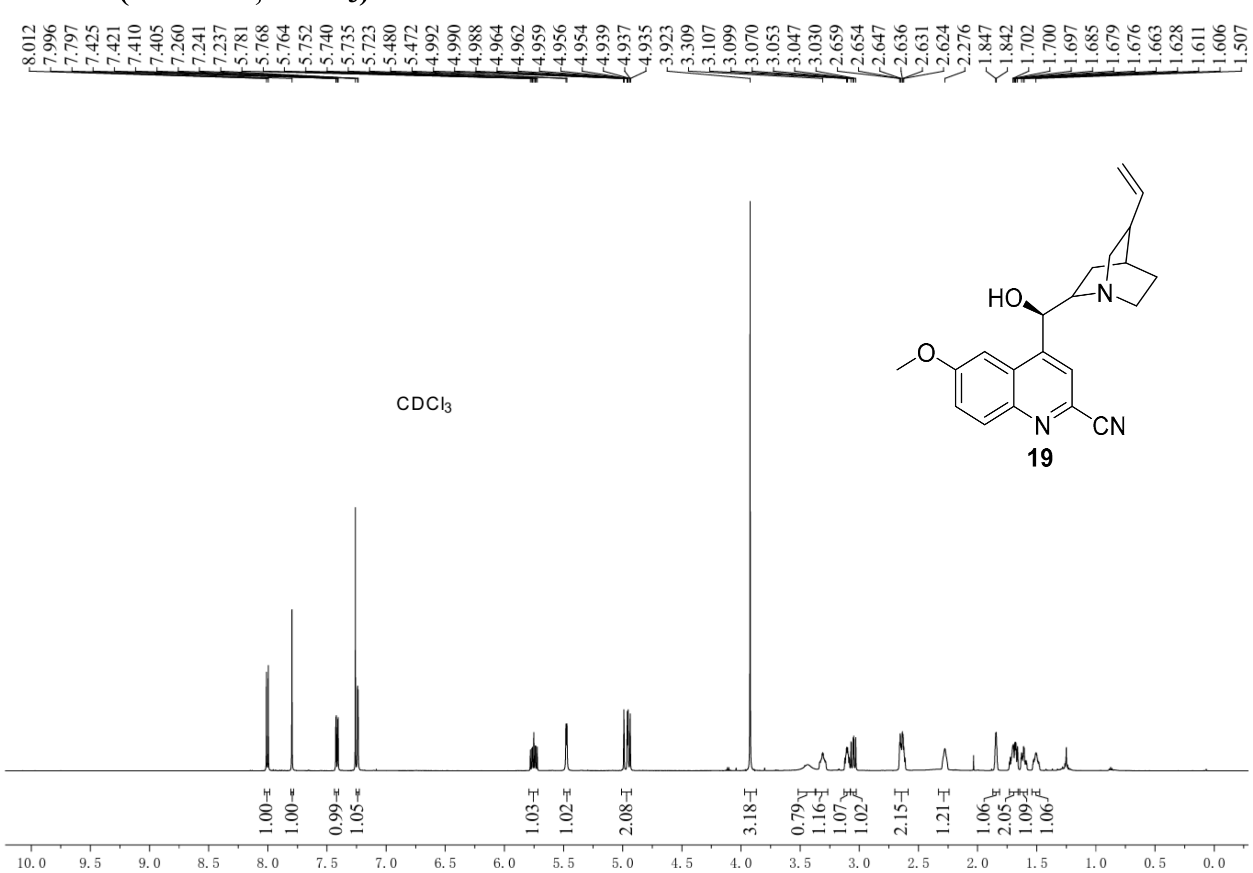

^{13}C NMR (100 MHz, CDCl$_3$):

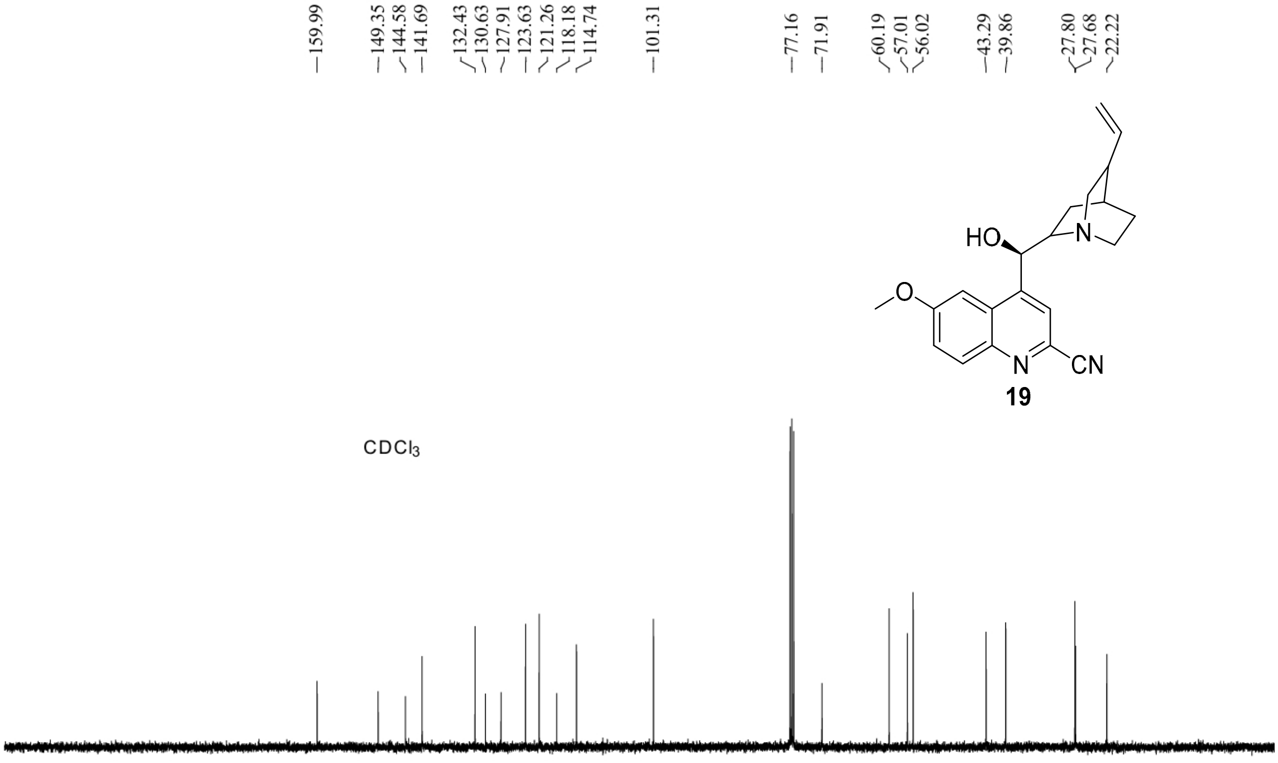

^{1}H NMR (600 MHz, CDCl$_3$):

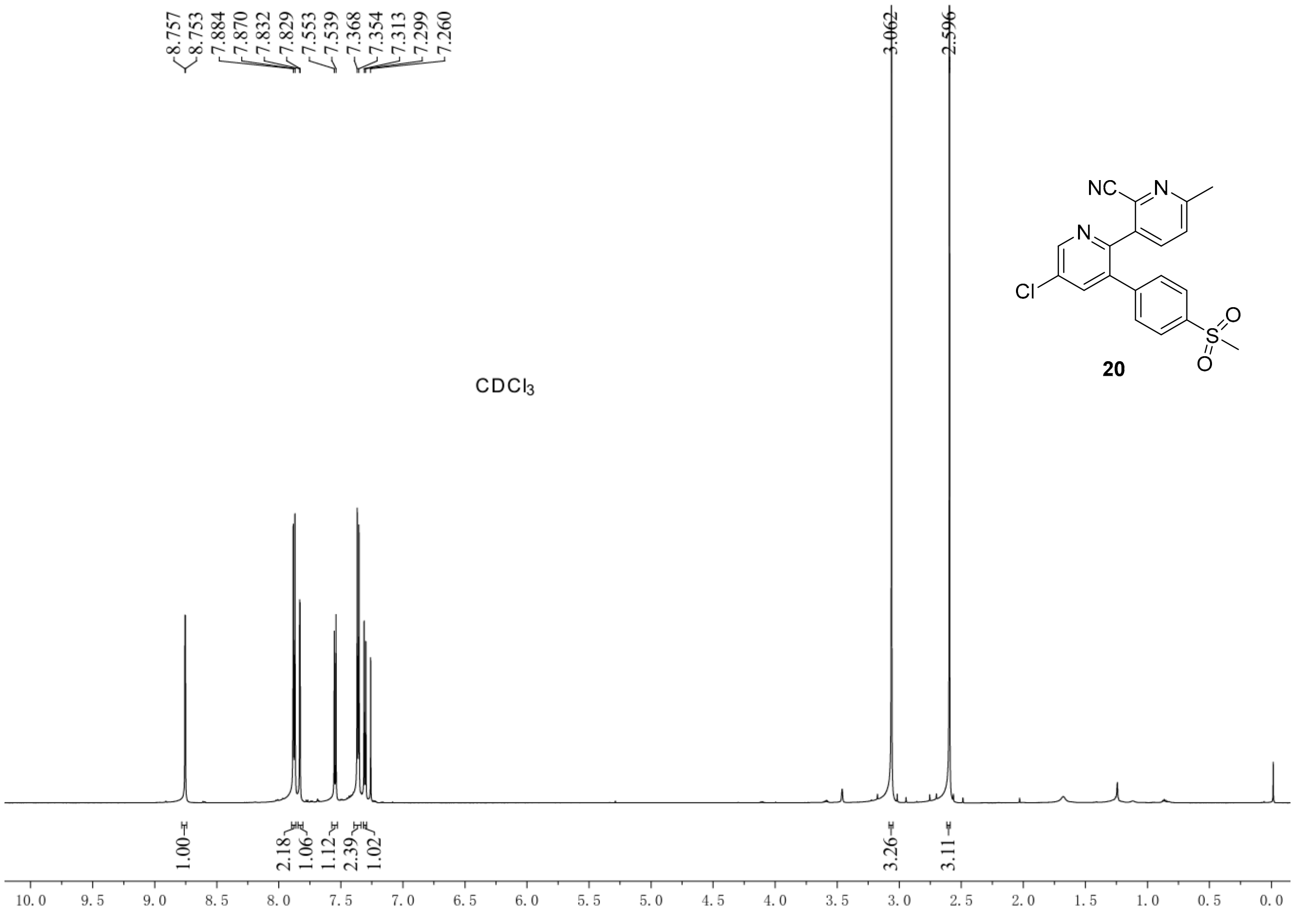

20

6. HRMS Spectra Data

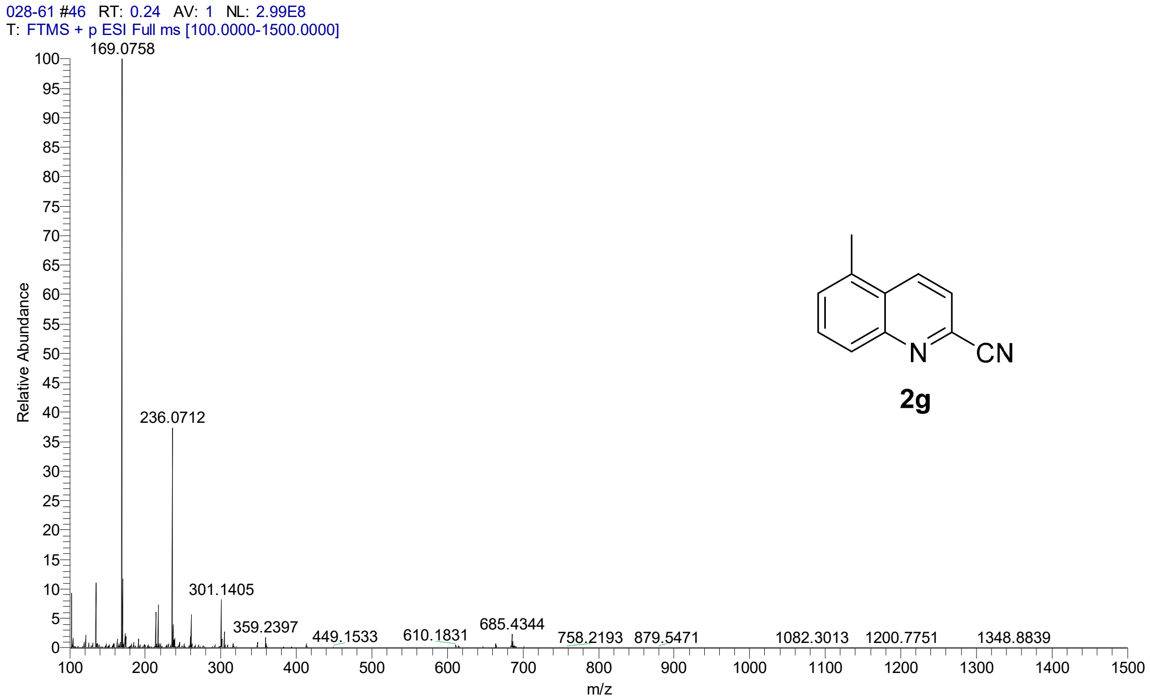

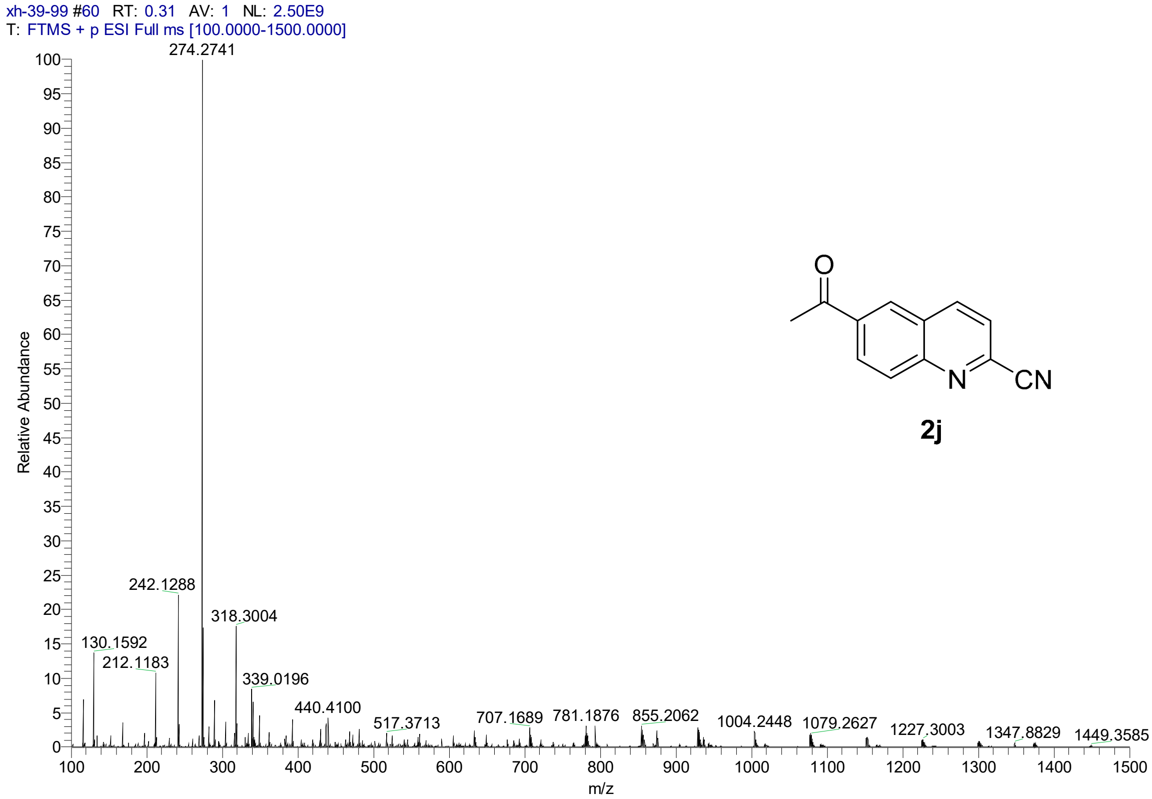

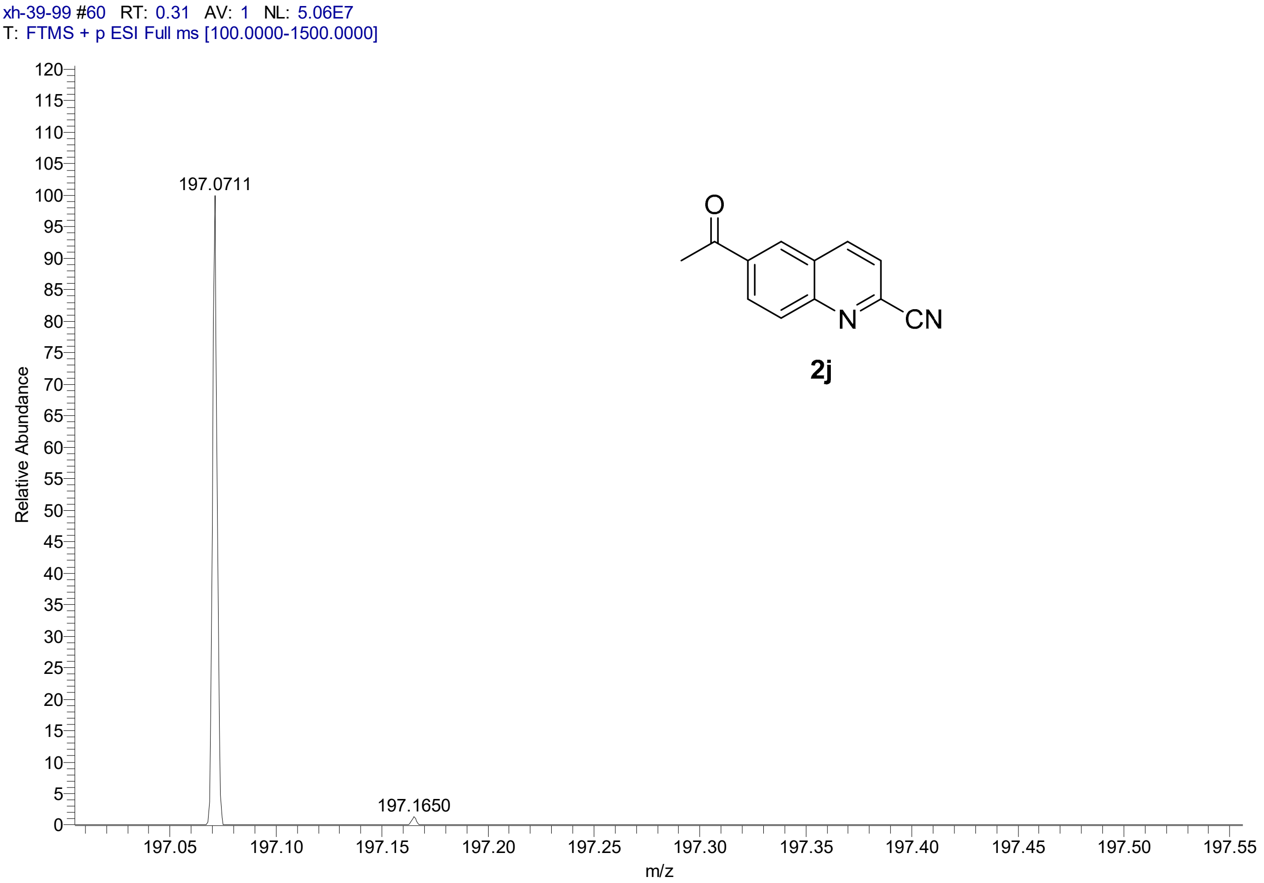
xh-39-99 #60 RT: 0.31 AV: 1 NL: 5.06E7
T: FTMS + p ESI Full ms [100.0000-1500.0000]
Relative Abundance
197.0711
197.1650
m/z
O
N
CN
2j

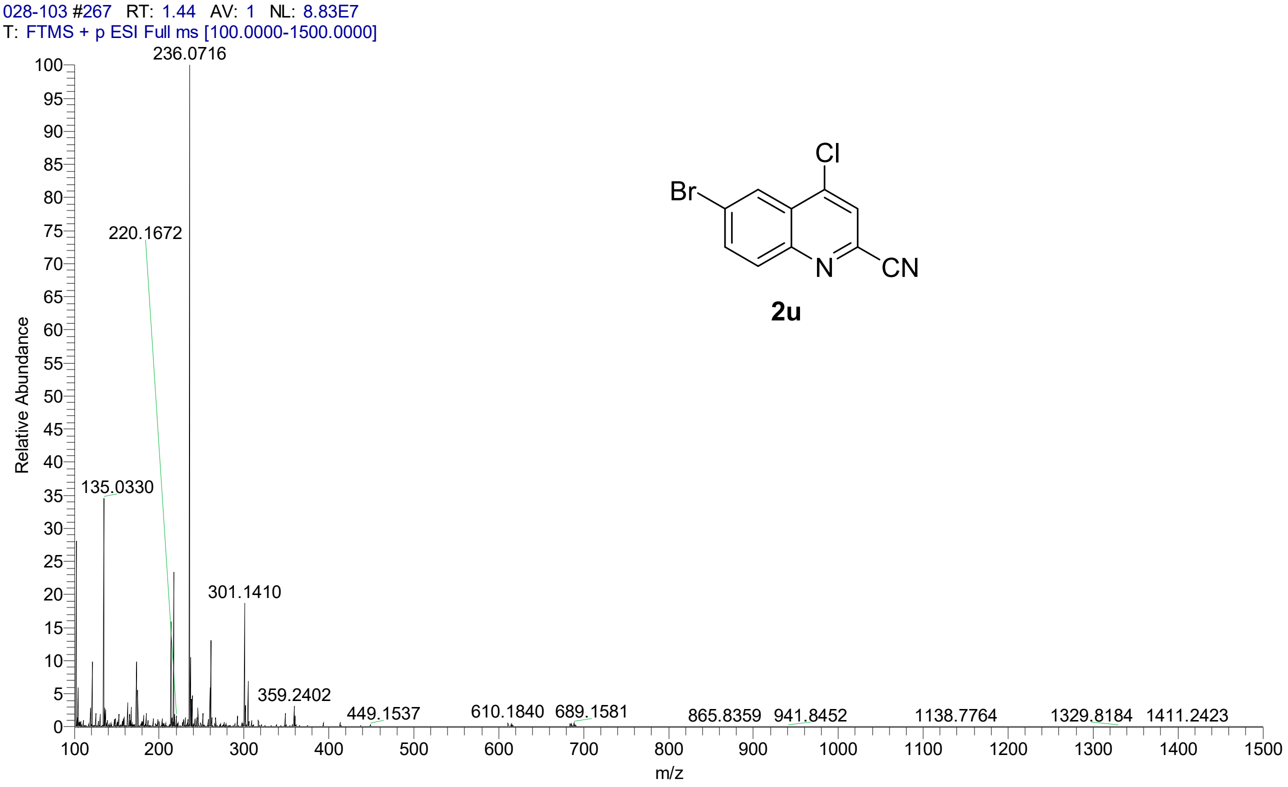
028-103 #267 RT: 1.44 AV: 1 NL: 8.83E7
T: FTMS + p ESI Full ms [100.0000-1500.0000]
Relative Abundance
236.0716
220.1672
135.0330
301.1410
359.2402
449.1537
610.1840
689.1581
865.8359
941.8452
1138.7764
1329.8184
1411.2423
m/z
Cl
Br
N
CN
2u

028-103 #267 RT: 1.44 AV: 1 NL: 1.16E5
T: FTMS + p ESI Full ms [100.0000-1500.0000]

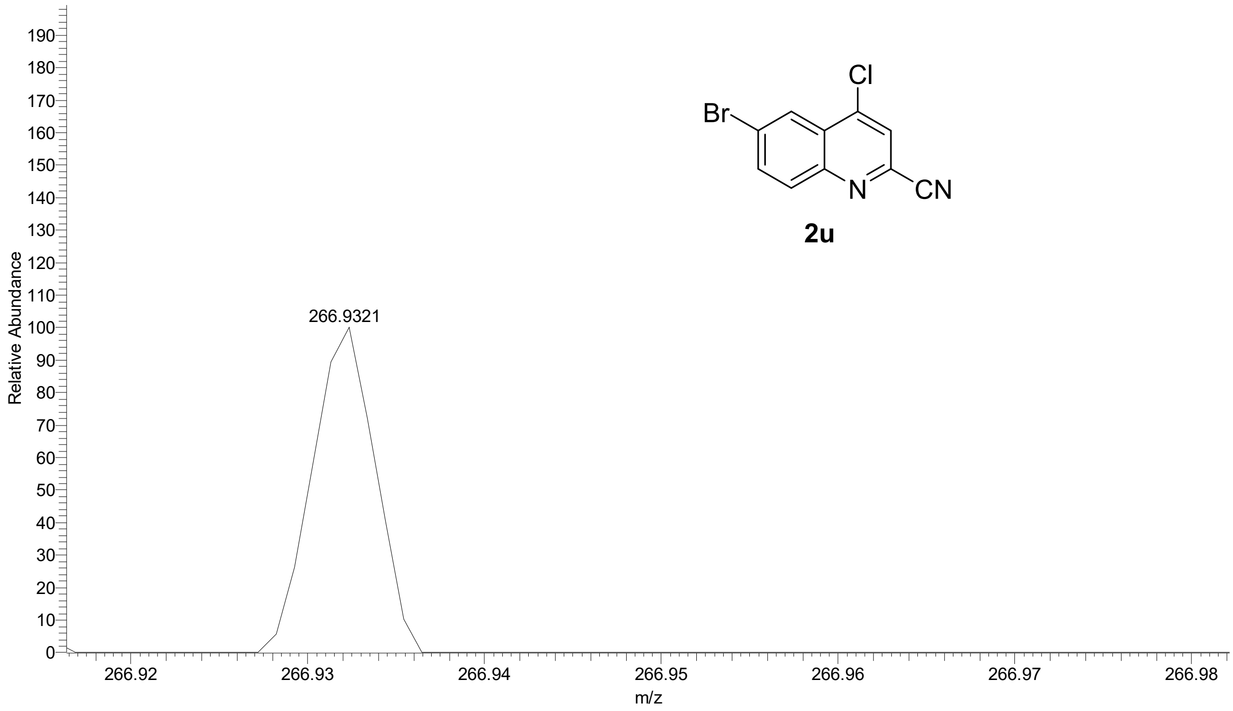

xh-39-55 #49 RT: 0.25 AV: 1 NL: 9.46E8
T: FTMS + p ESI Full ms [100.0000-1500.0000]

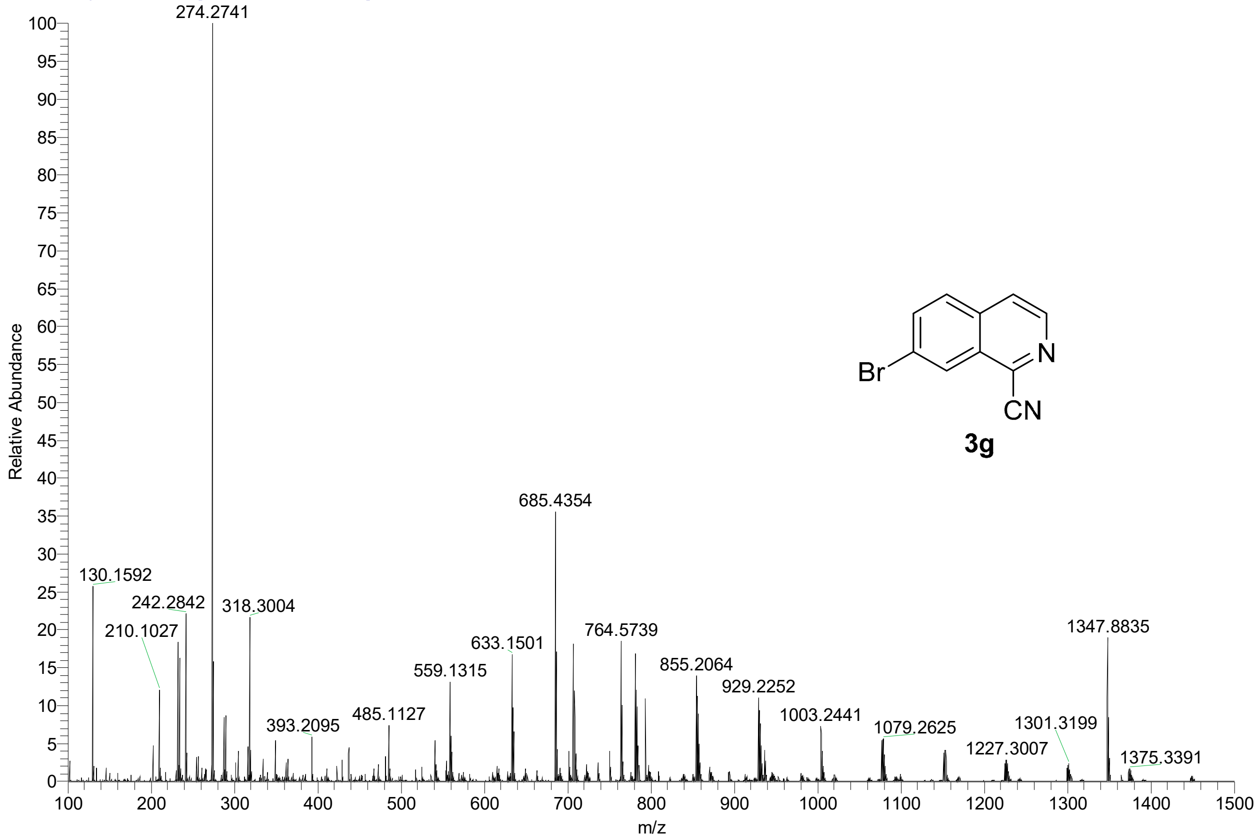

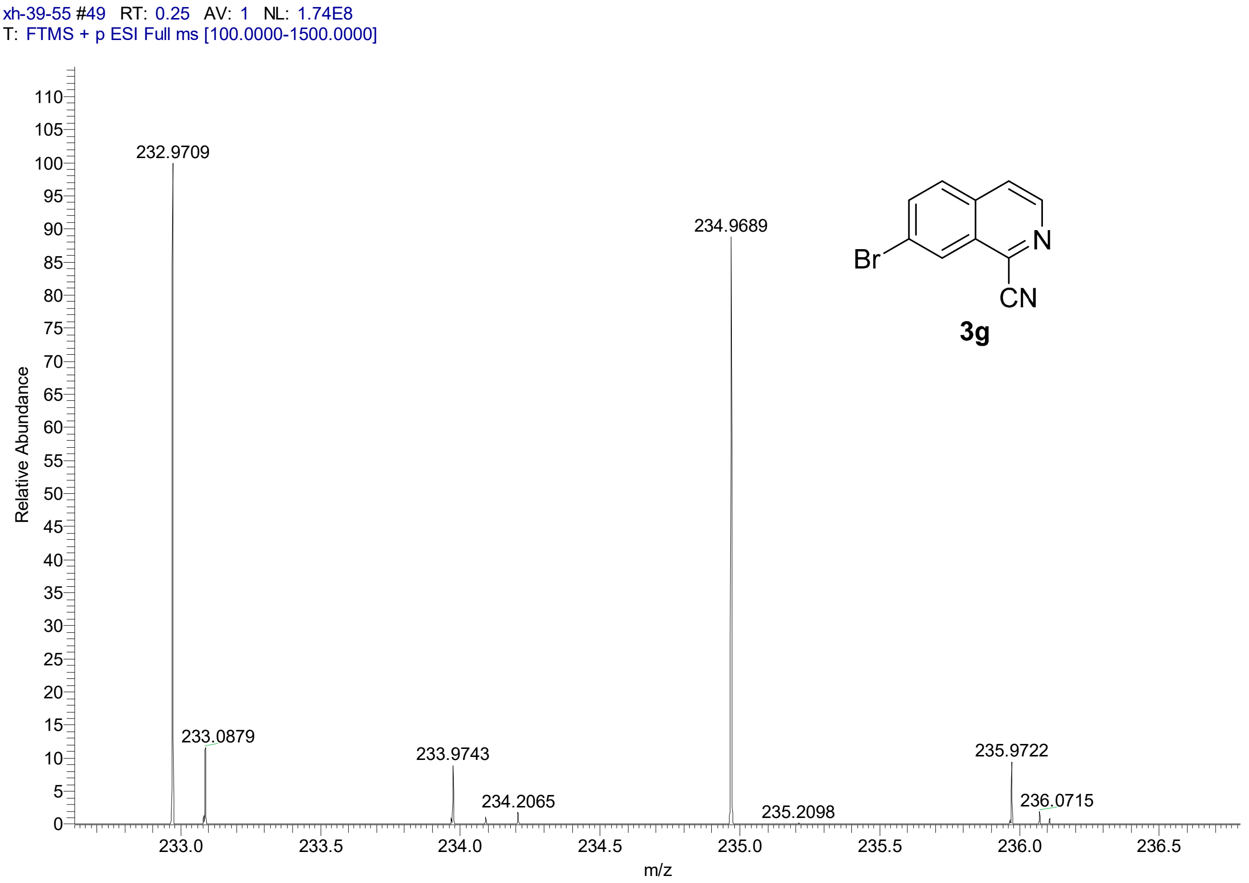
xh-39-55 #49 RT: 0.25 AV: 1 NL: 1.74E8
T: FTMS + p ESI Full ms [100.0000-1500.0000]
Relative Abundance
m/z
232.9709
234.9689
233.0879
233.9743
234.2065
235.2098
235.9722
236.0715
Br
N
CN
3g

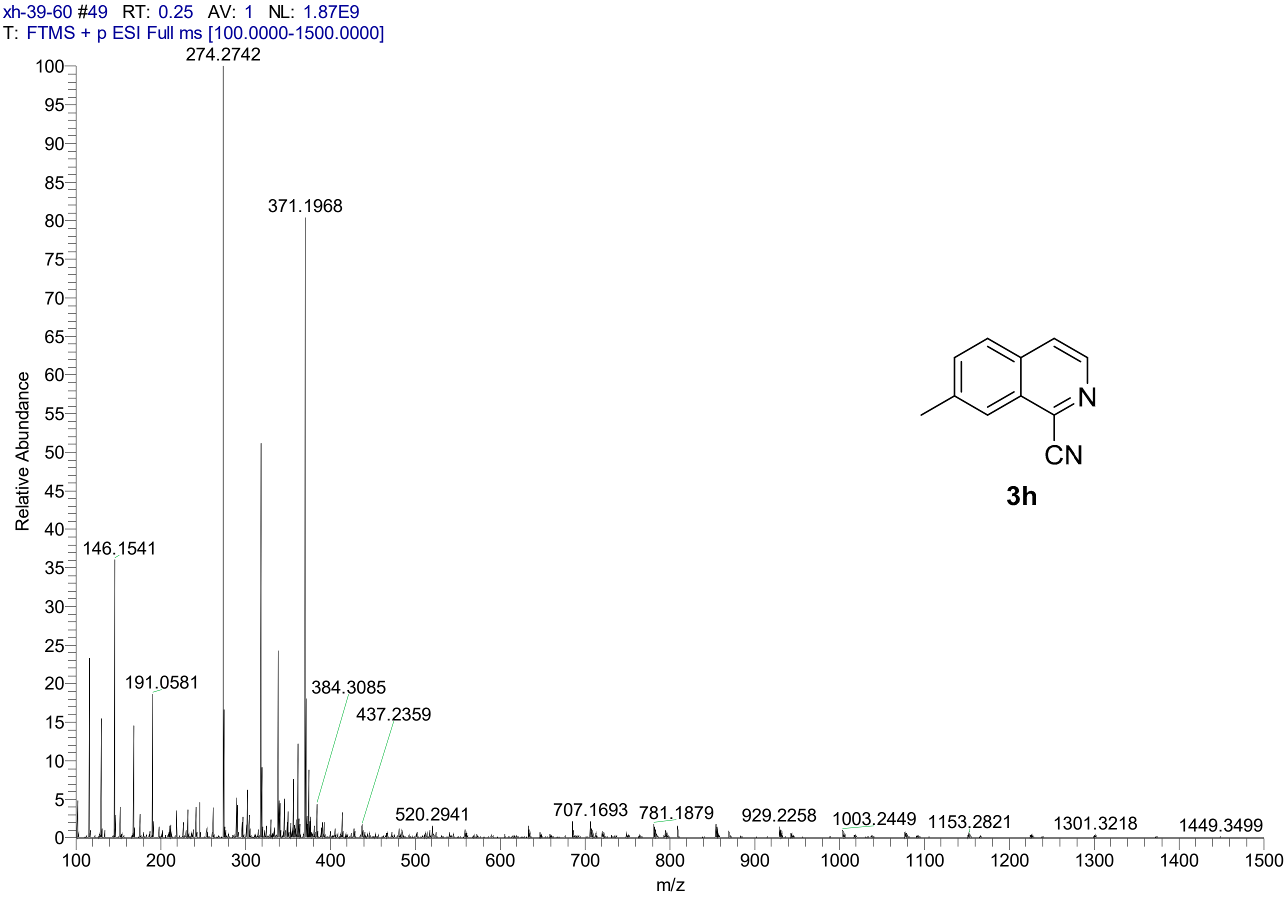
xh-39-60 #49 RT: 0.25 AV: 1 NL: 1.87E9
T: FTMS + p ESI Full ms [100.0000-1500.0000]
Relative Abundance
m/z
274.2742
371.1968
146.1541
191.0581
384.3085
437.2359
520.2941
707.1693
781.1879
929.2258
1003.2449
1153.2821
1301.3218
1449.3499
N
CN
3h

xh-39-57 #63 RT: 0.32 AV: 1 NL: 2.99E9
T: FTMS + p ESI Full ms [100.0000-1500.0000]

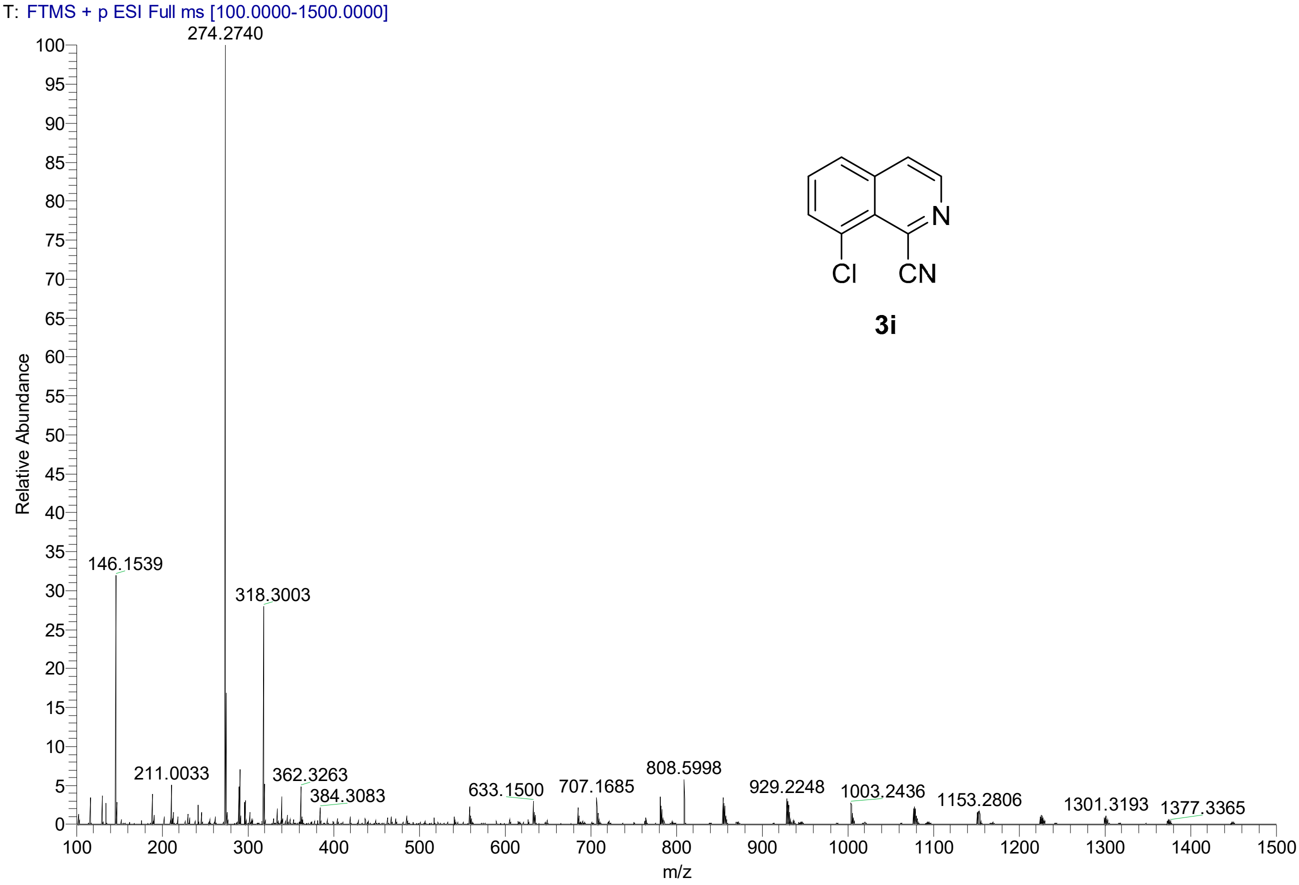

xh-39-57 #63 RT: 0.32 AV: 1 NL: 3.70E7
T: FTMS + p ESI Full ms [100.0000-1500.0000]

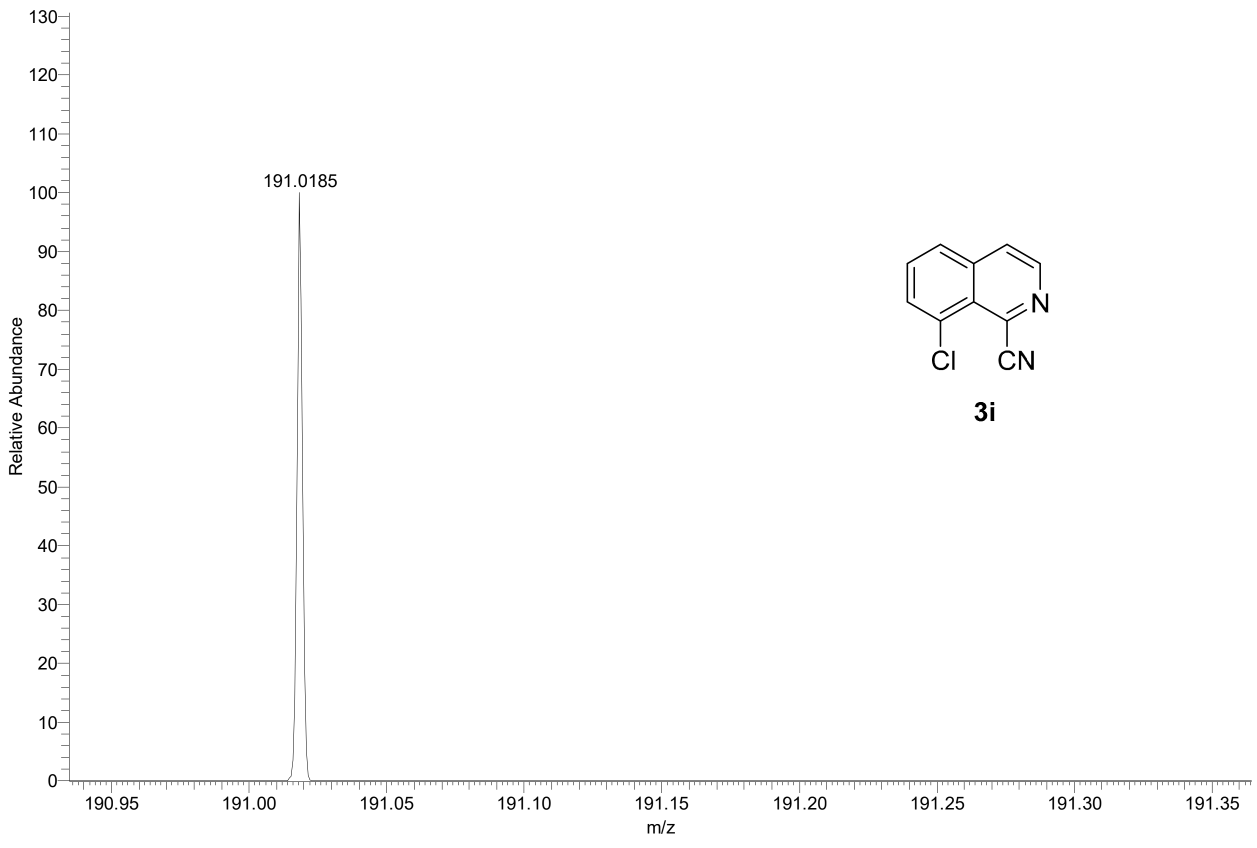

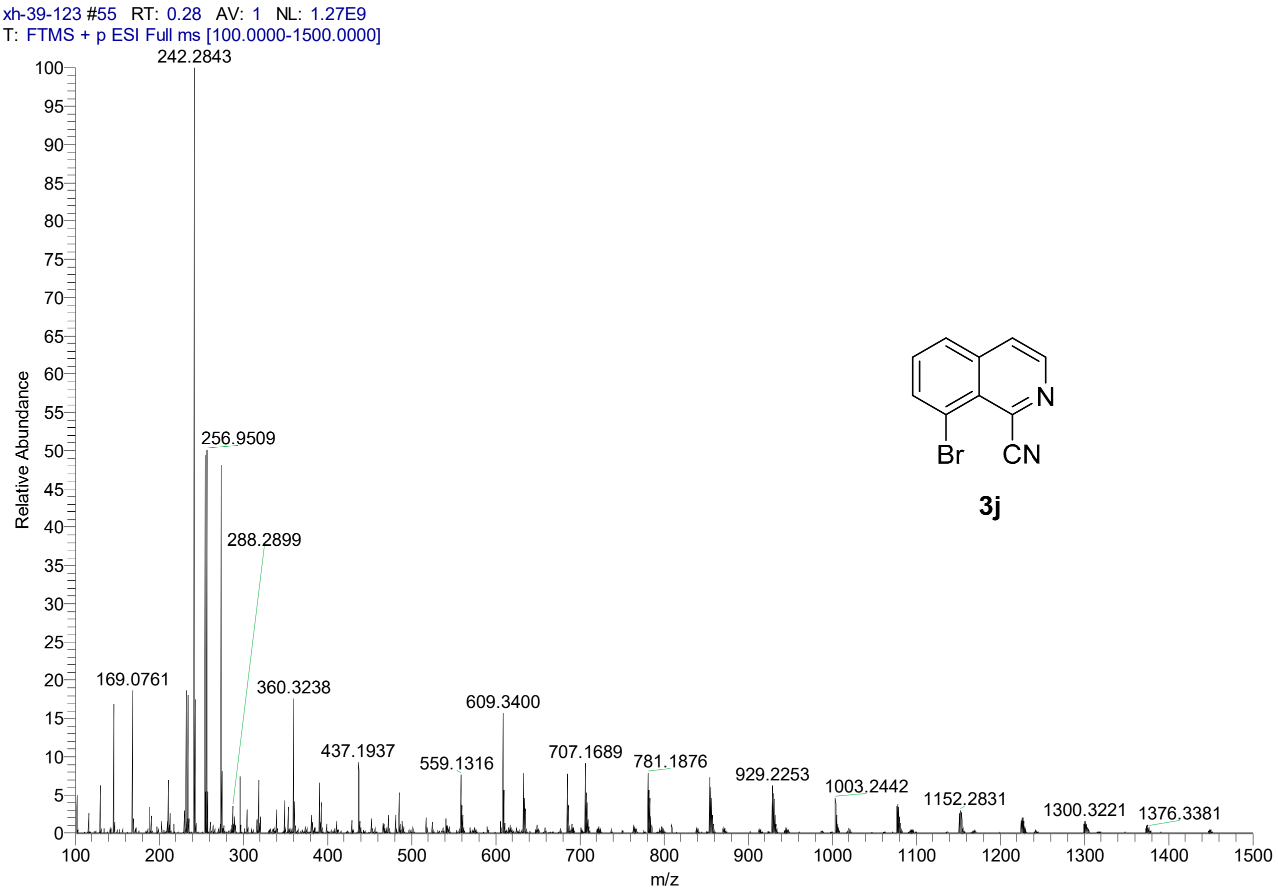
xh-39-123 #55 RT: 0.28 AV: 1 NL: 1.27E9
T: FTMS + p ESI Full ms [100.0000-1500.0000]
242.2843
256.9509
288.2899
169.0761
360.3238
609.3400
437.1937
559.1316
707.1689
781.1876
929.2253
1003.2442
1152.2831
1300.3221
1376.3381
Relative Abundance
m/z
Br
CN
N
3j

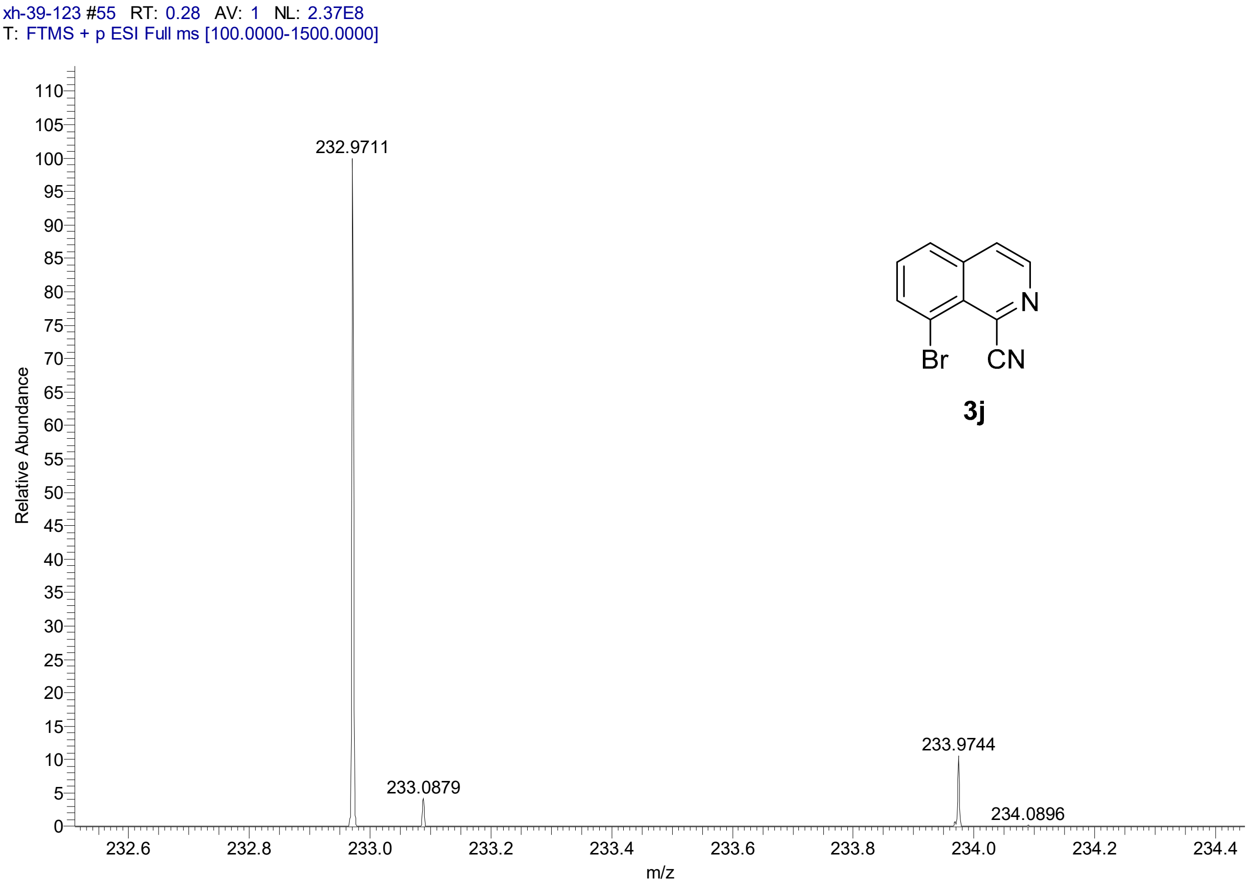
xh-39-123 #55 RT: 0.28 AV: 1 NL: 2.37E8
T: FTMS + p ESI Full ms [100.0000-1500.0000]
232.9711
233.0879
233.9744
234.0896
Relative Abundance
m/z
Br
CN
N
3j

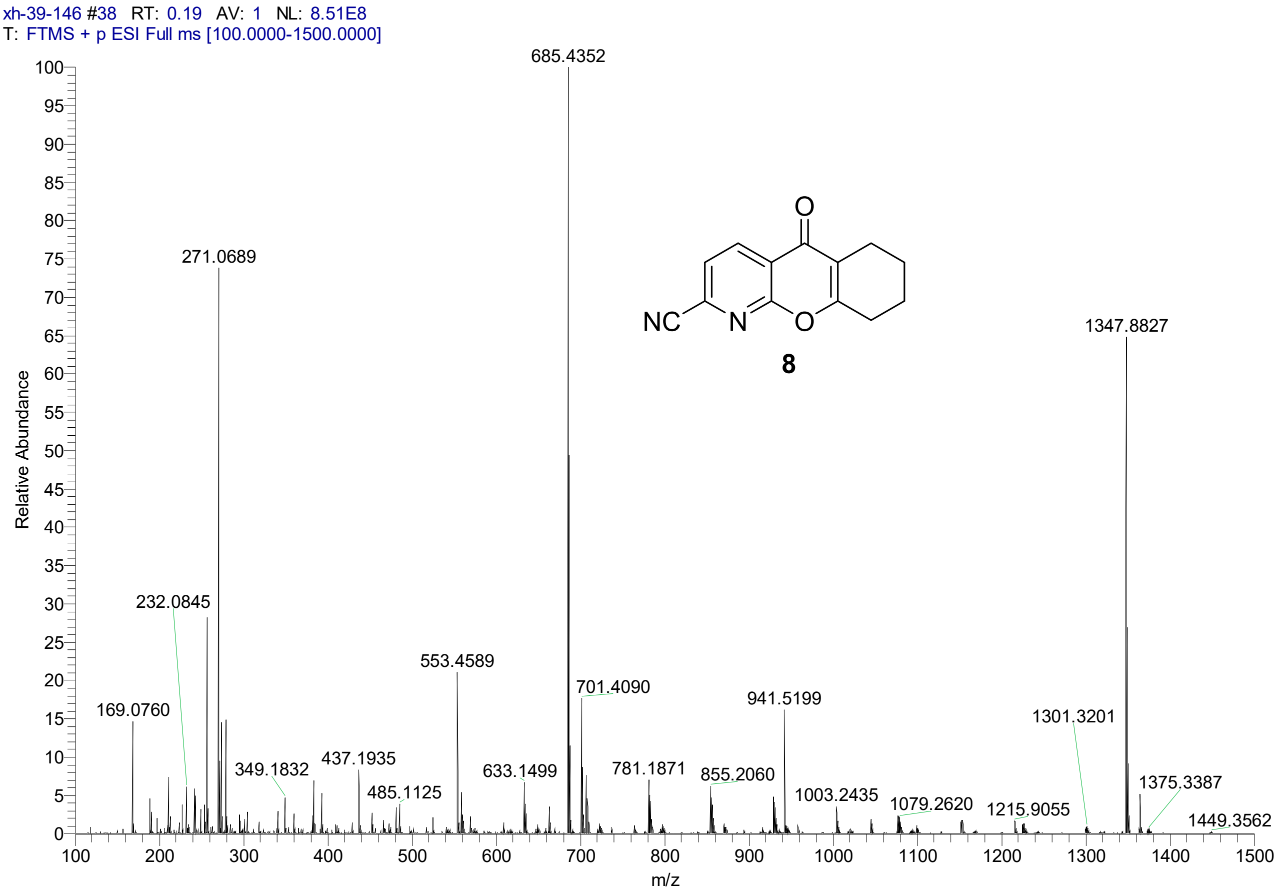
xh-39-146 #38 RT: 0.19 AV: 1 NL: 8.51E8
T: FTMS + p ESI Full ms [100.0000-1500.0000]
685.4352
271.0689
1347.8827
232.0845
553.4589
701.4090
941.5199
169.0760
1301.3201
349.1832
437.1935
485.1125
633.1499
781.1871
855.2060
1003.2435
1079.2620
1215.9055
1375.3387
1449.3562
Relative Abundance
m/z
NC
N
O
O
8

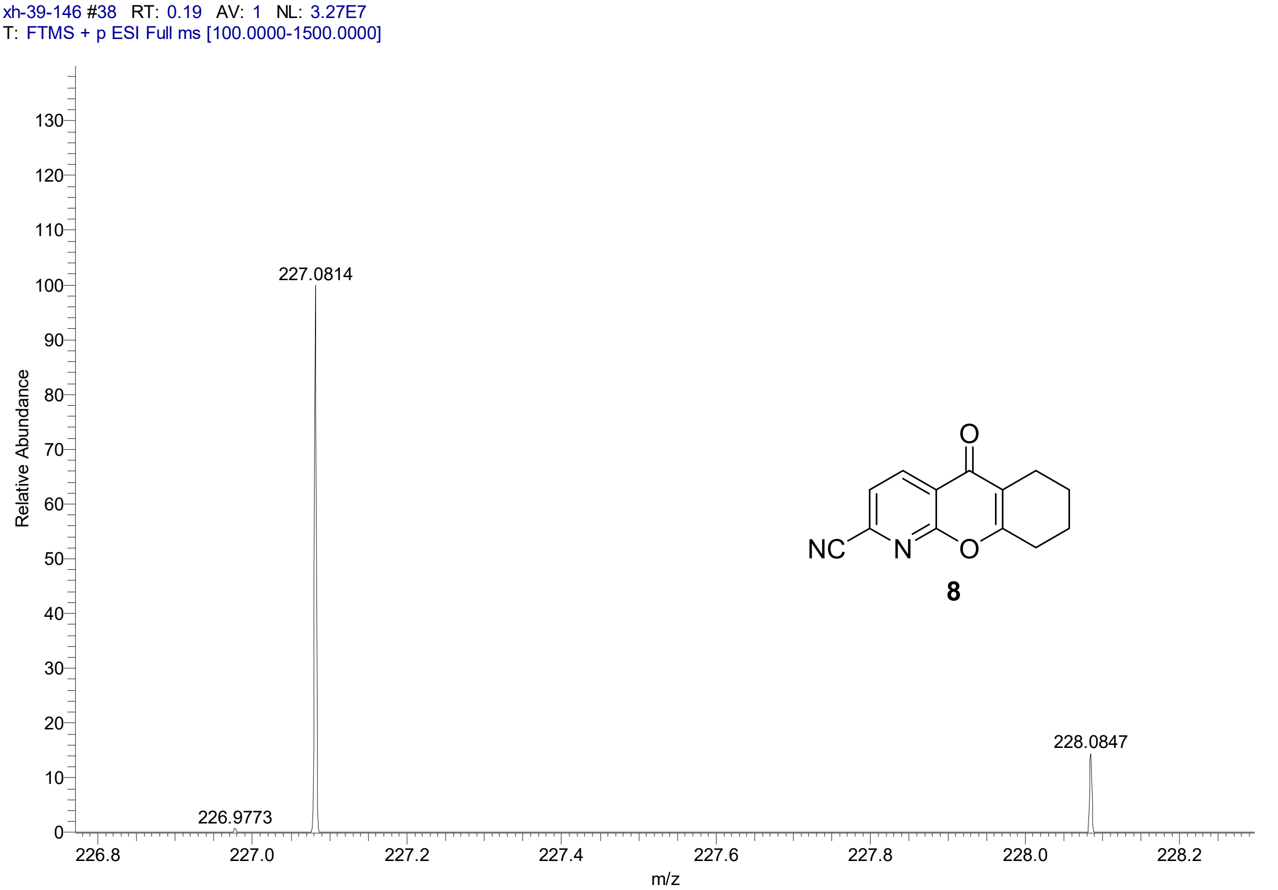
xh-39-146 #38 RT: 0.19 AV: 1 NL: 3.27E7
T: FTMS + p ESI Full ms [100.0000-1500.0000]
227.0814
228.0847
226.9773
Relative Abundance
m/z
NC
N
O
O
8

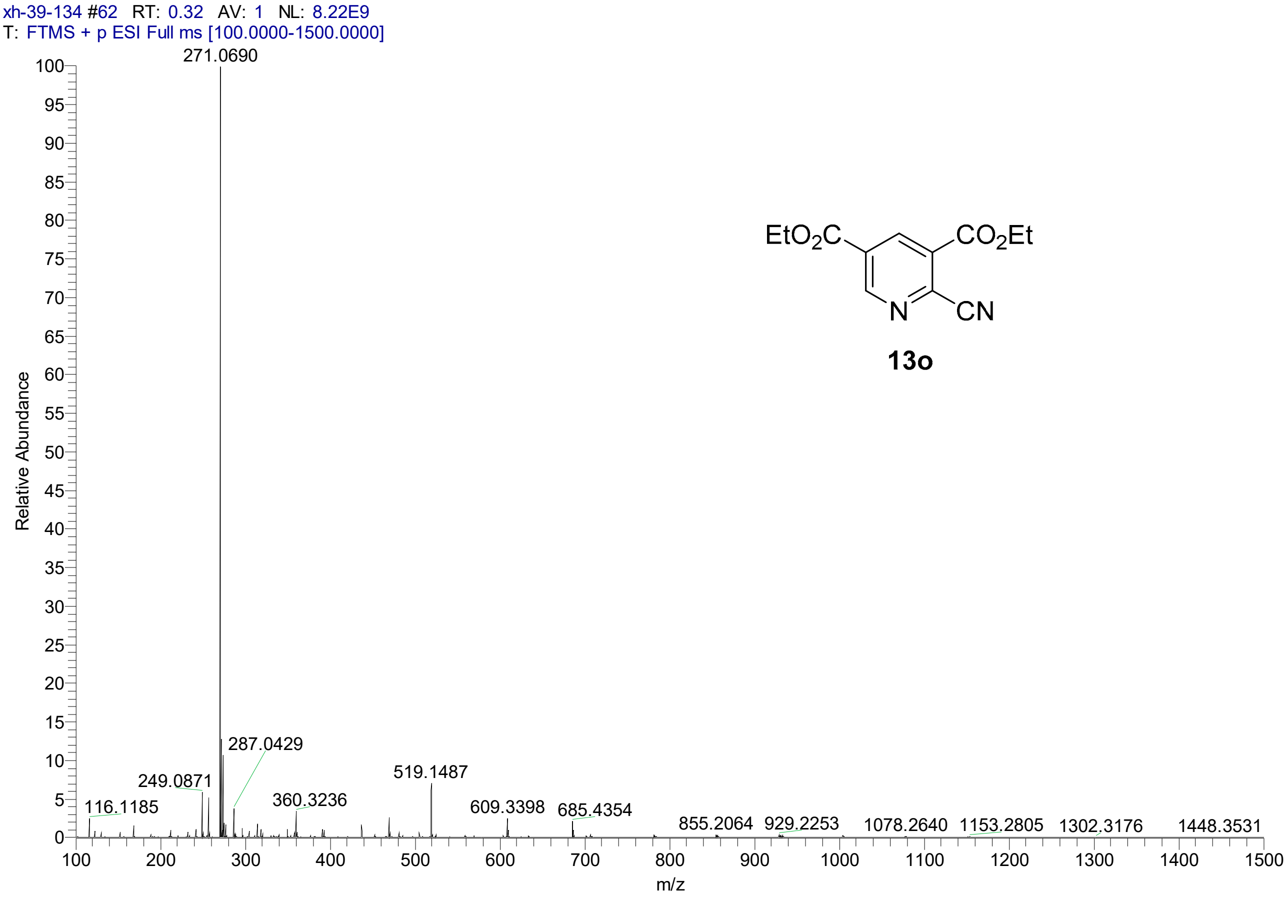
xh-39-134 #62 RT: 0.32 AV: 1 NL: 8.22E9
T: FTMS + p ESI Full ms [100.0000-1500.0000]
Relative Abundance
m/z
271.0690
287.0429
249.0871
519.1487
116.1185
360.3236
609.3398
685.4354
855.2064
929.2253
1078.2640
1153.2805
1302.3176
1448.3531
EtO_2C
CO_2Et
N
CN
13o

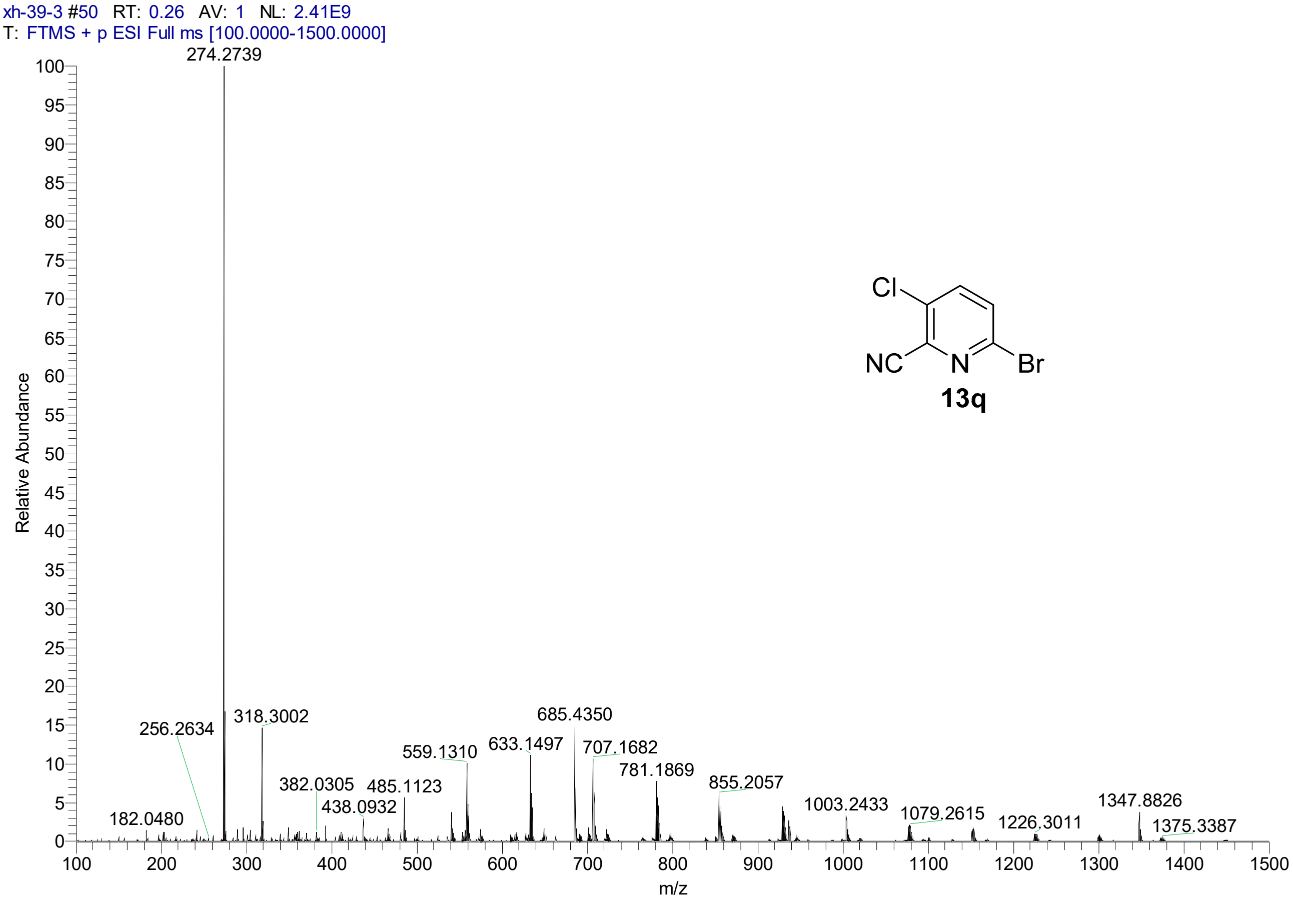
xh-39-3 #50 RT: 0.26 AV: 1 NL: 2.41E9
T: FTMS + p ESI Full ms [100.0000-1500.0000]
Relative Abundance
m/z
274.2739
256.2634
318.3002
685.4350
559.1310
633.1497
707.1682
781.1869
382.0305
485.1123
438.0932
855.2057
182.0480
1003.2433
1079.2615
1226.3011
1347.8826
1375.3387
Cl
NC
N
Br
13q

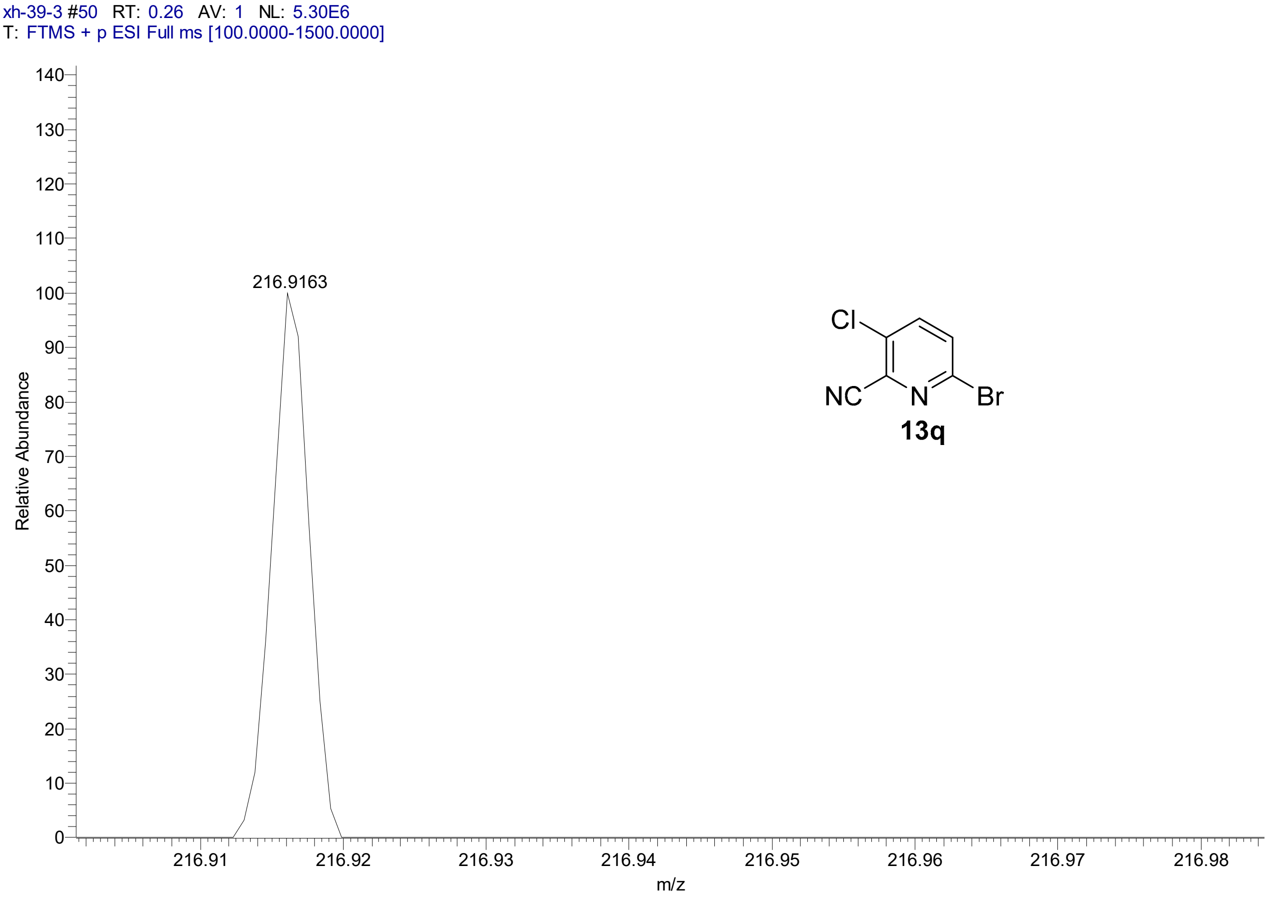
xh-39-3 #50 RT: 0.26 AV: 1 NL: 5.30E6
T: FTMS + p ESI Full ms [100.0000-1500.0000]
216.9163
Relative Abundance
m/z
Cl
NC
N
Br
13q

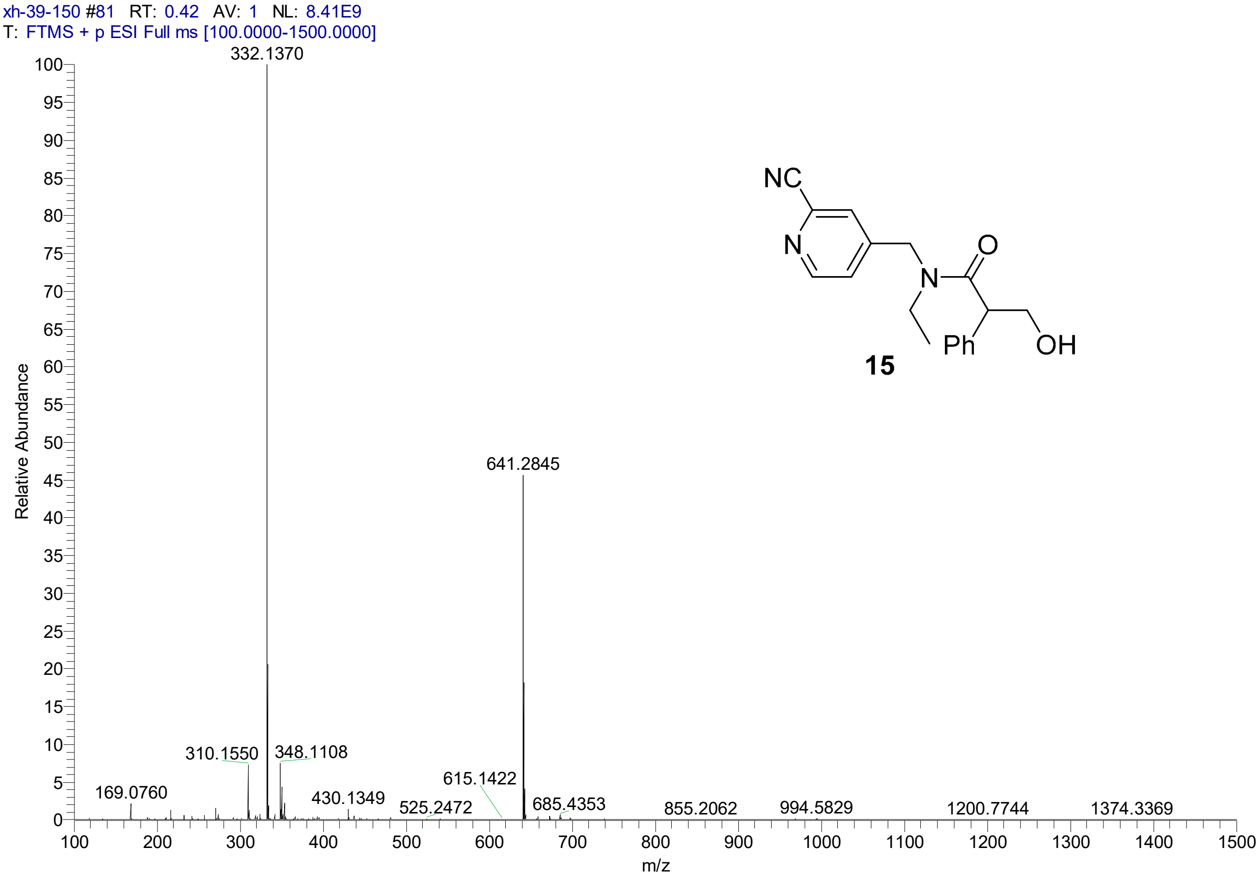
xh-39-150 #81 RT: 0.42 AV: 1 NL: 8.41E9
T: FTMS + p ESI Full ms [100.0000-1500.0000]
332.1370
641.2845
310.1550
348.1108
169.0760
430.1349
525.2472
615.1422
685.4353
855.2062
994.5829
1200.7744
1374.3369
Relative Abundance
m/z
NC
N
O
N
Ph
OH
15

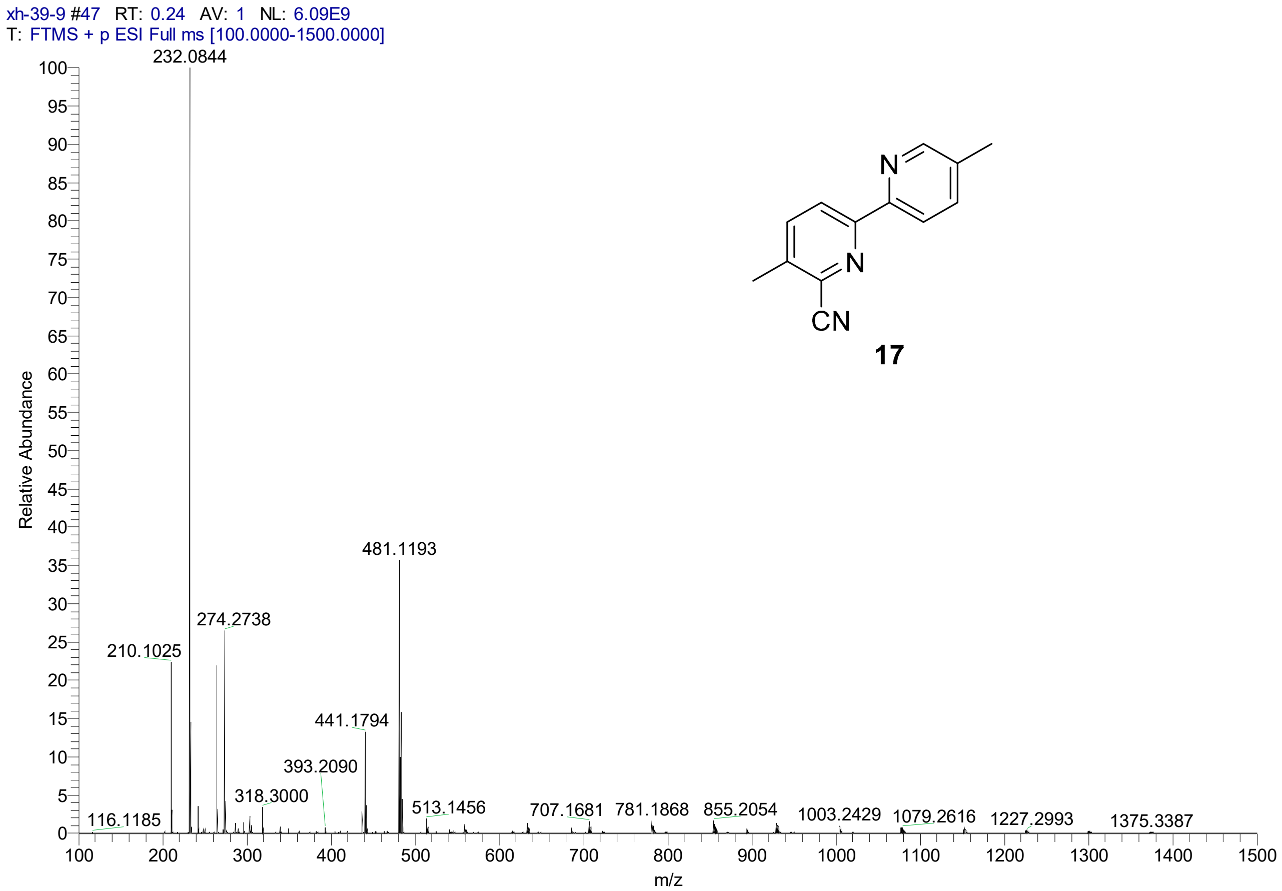
xh-39-9 #47 RT: 0.24 AV: 1 NL: 6.09E9
T: FTMS + p ESI Full ms [100.0000-1500.0000]
232.0844
481.1193
274.2738
210.1025
441.1794
393.2090
318.3000
116.1185
513.1456
707.1681
781.1868
855.2054
1003.2429
1079.2616
1227.2993
1375.3387
Relative Abundance
m/z
CN
N
17

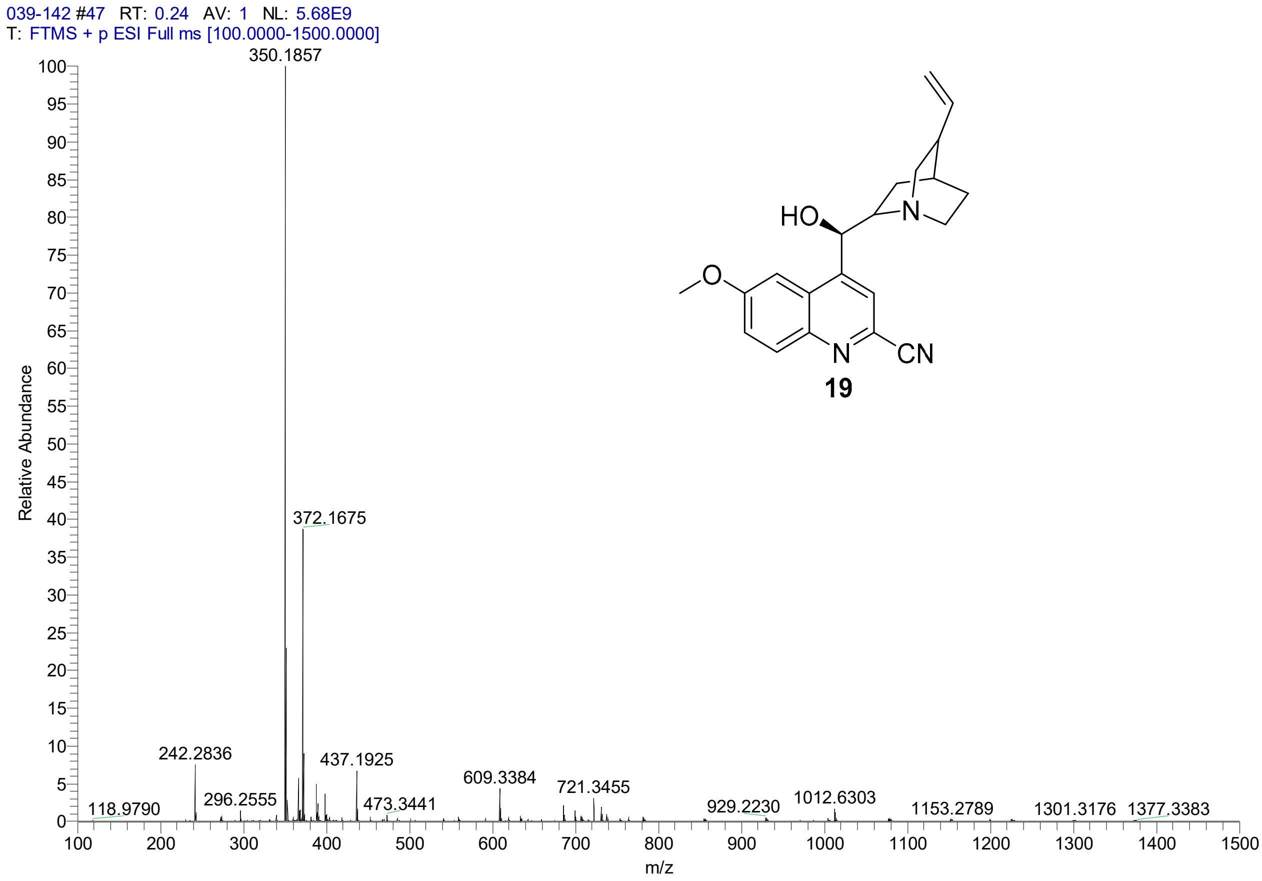
039-142 #47 RT: 0.24 AV: 1 NL: 5.68E9
T: FTMS + p ESI Full ms [100.0000-1500.0000]
350.1857
372.1675
242.2836
437.1925
609.3384
721.3455
296.2555
473.3441
118.9790
929.2230
1012.6303
1153.2789
1301.3176
1377.3383
Relative Abundance
m/z
HO
N
O
CN
19

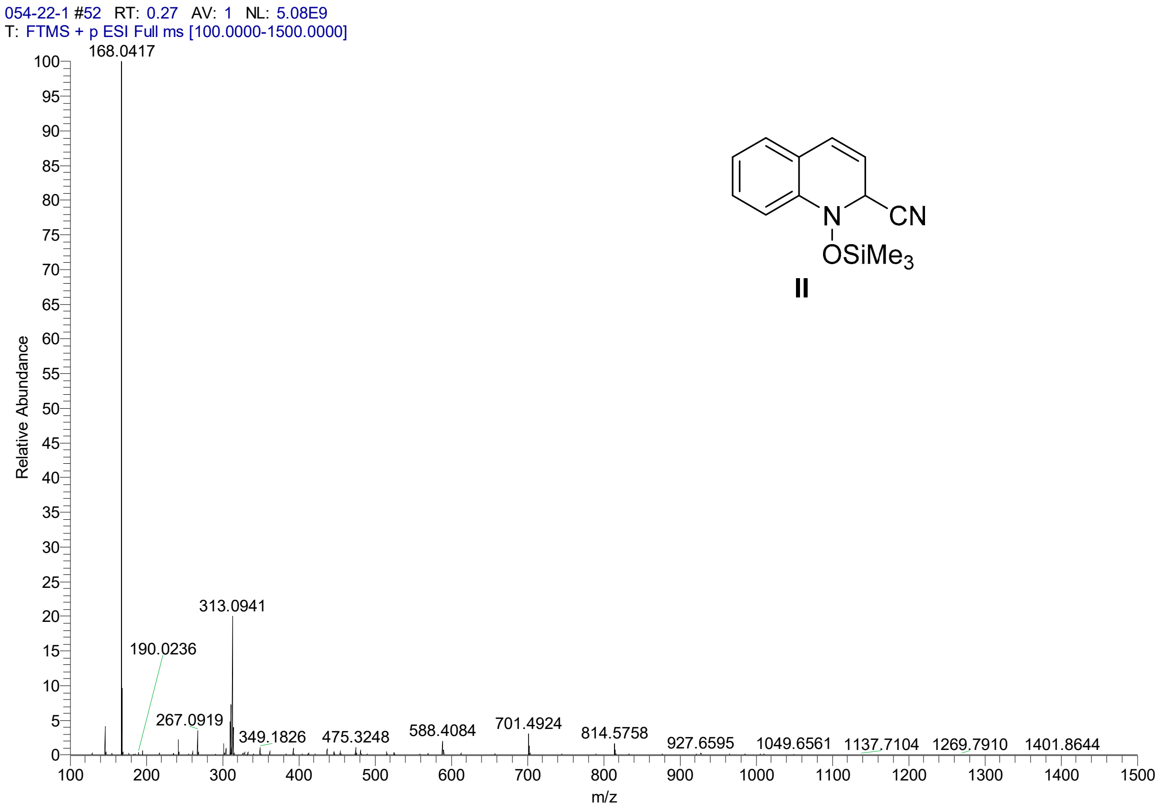
054-22-1 #52 RT: 0.27 AV: 1 NL: 5.08E9
T: FTMS + p ESI Full ms [100.0000-1500.0000]
168.0417
313.0941
190.0236
267.0919
349.1826
475.3248
588.4084
701.4924
814.5758
927.6595
1049.6561
1137.7104
1269.7910
1401.8644
Relative Abundance
m/z
CN
N
OSiMe3
II

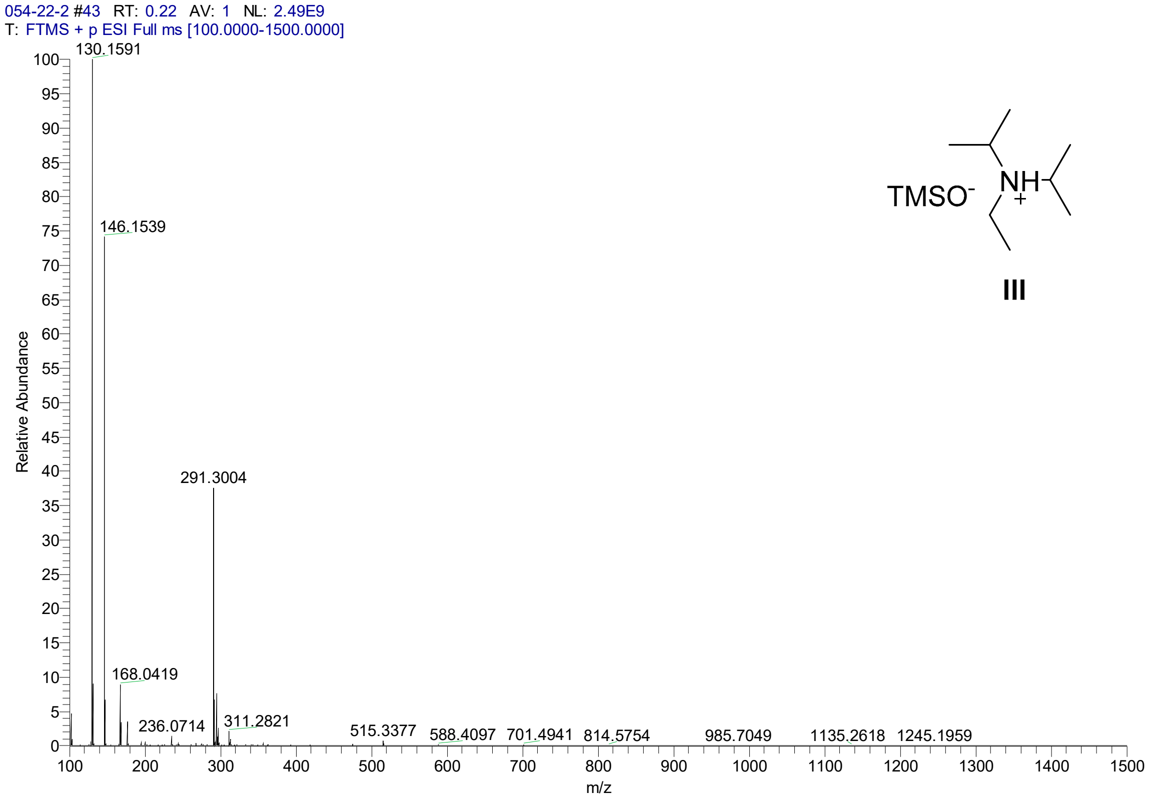
054-22-2 #43 RT: 0.22 AV: 1 NL: 2.49E9
T: FTMS + p ESI Full ms [100.0000-1500.0000]
130.1591
146.1539
291.3004
168.0419
236.0714
311.2821
515.3377
588.4097
701.4941
814.5754
985.7049
1135.2618
1245.1959
Relative Abundance
m/z
TMSO-
NH+
III
